# An annotated checklist of the coastal forests of Kenya, East Africa

**DOI:** 10.3897/phytokeys.147.49602

**Published:** 2020-05-12

**Authors:** Veronicah Mutele Ngumbau, Quentin Luke, Mwadime Nyange, Vincent Okelo Wanga, Benjamin Muema Watuma, Yuvenalis Morara Mbuni, Jacinta Ndunge Munyao, Millicent Akinyi Oulo, Elijah Mbandi Mkala, Solomon Kipkoech, Malombe Itambo, Guang-Wan Hu, Qing-Feng Wang

**Affiliations:** 1 CAS Key Laboratory of Plant Germplasm Enhancement and Specialty Agriculture, Wuhan Botanical Garden, Chinese Academy of Sciences, Wuhan 430074, Hubei, China; 2 Sino-Africa Joint Research Center (SAJOREC), Chinese Academy of Sciences, Wuhan 430074, Hubei, China; 3 University of Chinese Academy of Sciences, Beijing 100049, China; 4 East African Herbarium, National Museums of Kenya, P. O. Box 45166 00100, Nairobi, Kenya

**Keywords:** Biodiversity, checklist, coastal forests, endemic, threatened species

## Abstract

The inadequacy of information impedes society’s competence to find out the cause or degree of a problem or even to avoid further losses in an ecosystem. It becomes even harder to identify all the biological resources at risk because there is no exhaustive inventory of either fauna or flora of a particular region. Coastal forests of Kenya are located in the southeast part of Kenya and are distributed mainly in four counties: Kwale, Kilifi, Lamu, and Tana River County. They are a stretch of fragmented forests ca. 30−120 km away from the Indian Ocean, and they have existed for millions of years. Diversity of both fauna and flora is very high in these relicts and the coastal forests of Eastern Africa, extending along the coast from Somalia through Kenya and Tanzania to Mozambique, are ranked among the priority biodiversity hotspot in the world. In spite of the high plant species richness and their importance towards supporting the livelihoods of the communities that live around them, floristic studies in these forests have remained poorly investigated. Hence, based on numerous field investigations, plant lists from published monograph/literature, and data from BRAHMS (Botanical Records and Herbarium Management System) database at East African herbarium (EA), we present a detailed checklist of vascular plants recorded in this region. Our results show that Kenyan coastal forests play an essential role in the flora of Kenya and the plant diversity of the coastal forests of East Africa. The checklist represents 176 families, 981 genera, 2489 species, 100 infraspecific taxa, 90 endemic plants species, 72 exotic species, and 120 species that are included in the current IUCN Red List of Threatened Species as species of major concern. We also discovered three new species to the world from these relicts. Thus, Kenyan coastal forests present a remarkable and significant center of plant diversity.

## Introduction

Africa is among the world’s major centers of endemism and species-rich biodiversity regions (Burgess and Clarke 2000; Linder 2001). It hosts several centers of diversity with the eastern moist forests, which include coastal forests of East Africa and Eastern Arc forests as one of the significant centers of diversity (Lovett 1998). Another important center is the Congo Basin rainforest, which forms the second largest extent of continuous rainforest in the world (Linder 2014). Hamilton (1974, 1982) presented four centers of endemism in Africa, which include Kivu, the East African coast, Cameroon Gabon area, and possibly small refugia in West Africa. Brenan (1978), working with data arranged according to country, identified the same regions as being rich in endemism.

Coastal forests of Eastern Africa are ranked among the 35 identified world’s biodiversity hotspots due to the concentration of many endemic species and habitat loss (Mittermeier et al. 1998, 2004; Olson and Dinerstein 1998). Together with the Eastern Arc mountains, they contain approximately 2000 endemic plant species (WWF–US 2003b) . These forests have lived for millions of years, of which they existed as a continuous belt of forest between the East and West coast referred to as the ancient Pan-African forest. However, fluctuation of climate, during the last 2 million years, caused their fragmentation, leading to loss of some lowland dry forests in Africa (Hawthorne et al. 1981). Today, these forests have lost considerable amounts of their wilderness due to anthropogenic pressures leading to small fragments up to less than 5 km^2^, with the most extensive patches recorded in the Kenya coastal forests (Wass 1995; Habel et al. 2017).

Loss of habitat due to human activities is the most severe threat to biodiversity and has become a major global environmental problem (Collinge 1996), hence, plant diversity protection has attracted more attention. Coastal forests of East Africa are ranked among the world’s ten most threatened forest hotspots because they have lost more than 90% of their original habitat. Similarly, Brooks et al. (2002) and Azeria et al. (2007) pointed out that Northern Kenya and Southern Tanzania coastal forests should be highly prioritized because they are in danger of losing most of their biodiversity in coming times.

Floristic studies, however, provide a basic outline for plant conservation. Based on these studies, it is possible to determine the condition of an ecosystem, the primary relationships of species with each other or with the environment, and the identity of rare species or widespread species (Ivanauskas et al. 2007). Therefore, they must be carried out not only in a particular area but also over time (Mota et al. 2017). In spite the coastal forests of Kenya acting as significant reservoirs of carbon and biodiversity, and supporting the livelihoods of rural people, plant species diversity remains poorly studied. Despite a substantial amount of floristic research having been performed over the last decades on some of the Kenyan coastal relicts, (Robertson and Luke 1993; Lehmann and Kioko 2005; Luke 2005), a comprehensive study of the whole coastal region of Kenya is still extremely urgent due to its vast area and large number of threatened and endemic taxa. In addition, these forests are vital in providing ecological services at local, national, and global levels; it is therefore, crucial to understand their composition. Here we aim at presenting a detailed checklist of vascular plants recorded in the whole coastal region of Kenya. We achieved this through numerous field investigations carried out from 2015 to 2018, checking of plant species data from published monographs or literature (Beentje 1994; Luke 2005; Flora of Tropical East Africa (FTEA) 1952–2012), and obtaining data documented in the BRAHMS (Botanical Research and Herbarium Management System) database at the East African Herbarium (EA). We also document endemic and threatened species found in this region.

## Material and methods

### Study area

**Figure 1.**
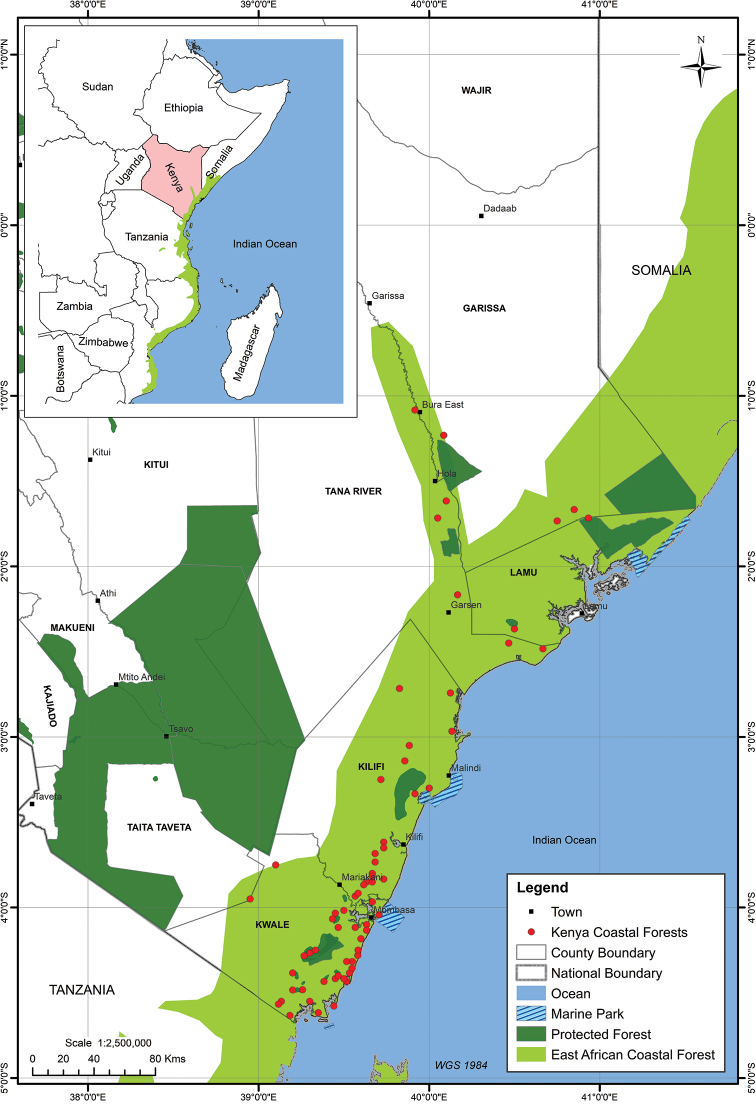
A map showing the study area and distribution of forest patches along the Kenyan coastline.

The study was carried out in the Kenyan coastal region located in the southeast part of Kenya (Fig. 1). Coastal forests are mainly distributed in four counties in the coastal province of Kenya, lying within the latitude of 1°40' to about 4°40' south and between 0–ca. 840 m a.s.l. especially at around Kilibasi (Moomaw 1960; Robertson and Luke 1993). They are part of the larger coastal forests of East Africa which cover an area of approximately 3170 km^2^ (Azeria et al. 2007; Wegner et al. 2009) of which, Kenyan coastal forests cover about 787 km^2^ (Younge et al. 2002; Burgess et al. 2003). They contain various forest patches with Arabuko Sokoke, and Shimba hills National Reserves forests being the largest remaining forests patches (Burgess et al. 1998, 2003; Younge et al. 2002; Matiku et al. 2013). Younge et al. (2002), estimated a total area covered by the coastal woodland/bushland to be 120 000 ha. Of this, 114 460 ha are in national reserves, 50 790 ha is in forest reserves, 16 000 ha remains ungazetted, and 10–200 ha are in the sacred Kaya Forests. The coastal region of Kenya supports a mosaic of different vegetation types. Much of the area supports bushland or thicket habitats, transition woodland, swampy forests, edaphic grasslands habitats, moist forests, and dry forests. The littoral vegetation includes mangrove vegetation along some parts of the coast and shoreline vegetation (Githitho 2004).

### Plant collection and nomenclature

Plant species data for the checklist of Kenya coastal forests were obtained from three primary sources. Firstly, collection records collected during floristic surveys carried out between 2015 and 2018. Different forest patches were visited and surveyed at different seasons to mainly cover the area where there had been few or no previous collections. The investigations included random walks while collecting fertile plant specimens. Details of collected specimens were recorded in a notebook. Information such as habit, habitat, location, and collector details were recorded as well. All collected materials were then processed (pressed and dried) and later deposited at the East Africa herbarium (EA). Duplicates were stored at Wuhan Botanical Garden (HIB). Secondly, plant species data were obtained from the BRAHMS (Botanical Research and Herbarium Management System) database in the East Africa herbarium (EA). Lastly, plant species data for coastal forests of Kenya were assembled from monographs and published literature (Beentje 1994; Luke 2005; FTEA 1952–2012).

Further information about the species recorded at the coastal forests was checked on specimens found in the Global Biodiversity Information Facility (GBIF) (www.gbif.org). Identification of collected specimens was made using taxonomic monographs/floras (FTEA) also through checking and comparing with identified specimens at the East African Herbarium (EA). Family and species circumscription, as well as spelling, authorities, and synonyms of scientific names, were updated based on online databases such as African plant database (www.villege.ch/musinfo/bd/cjb/africa), Catalogue of life (Roskov et al. 2019), and Tropicos (www.tropicos.org). Finally, we identified threatened and near threatened species based on the International Union for Conservation of Nature (IUCN) Red List of Threatened Species (IUCN 2019).

## Results and conclusion

### Species diversity

A total of 2489 vascular plant species and 100 infraspecific taxa (subspecies and varieties) belonging to 981 genera and 176 families are comprehensively listed and presented here. This represents 39.55%, 63.78%, and 78.67%, respectively, of the total number of taxa found in Kenya flora (FTEA 1952–2012). Also, it represents more than half the total number of plant species found at the coastal forests of East Africa (WWF–US 2003b). 120, 89, and 72 entries represent threatened and near threatened, endemic, and exotic species of the coastal forests of Kenya, respectively (Tables 5, 7, 9). Three new species were discovered and described from these relicts; *Adenia angulosa*Passifloraceae (Ngumbau et al. 2017) recorded from the coastal forests of Kenya and Tanzania, *Zehneria monocarpa*, Curcubitaceae (Ngumbau et al. unpublished) from Kenyan coastal relicts, and *Croton kinondoensis*, Euphorbiaceae, (Ngumbau et al. in press). Morphology and ITS sequence of *Croton kinondoensis* placed it under the Adenophorus group, which is known to be endemic to Madagascar and Comoros Islands. This new entity represented an independent dispersal of *Croton* from Madagascar to Africa mainland (Ngumbau et al. in press).

The top 5 most species-rich families for coastal forests of Kenya are Fabaceae (226 species), Poaceae (207 species), Rubiaceae (137 species), Malvaceae (117 species), and Cyperaceae (111 species). The top 5 most species-rich genera are *Cyperus* (48 species, Cyperaceae), *Euphorbia* (28 species, Euphorbiaceae), *Justicia* (27 species, Acanthaceae), *Ipomoea* (25 species, Convolvulaceae), and *Crotalaria* (23 species, Fabaceae) (Table 1).

**Table 1.** The 10 largest families and genera of the vascular plants of Kenya coastal region.

Family	Genus	Species	Genus	Species
Fabaceae	80	226	* Cyperus *	48
Poaceae	72	207	* Euphorbia *	28
Rubiaceae	63	137	* Justicia *	27
Malvaceae	30	117	* Ipomoea *	25
Cyperaceae	18	111	* Indigofera *	24
Acanthaceae	30	108	* Crotalaria *	23
Apocynaceae	53	101	* Berlaria *	19
Euphorbiaceae	26	87	* Ficus *	19
Asteraceae	49	82	* Combretum *	19
Lamiaceae	18	67	* Hibiscus *	18
Orchidaceae	23	60	* Grewia *	17

### Growth forms

The growth forms (trees, shrubs, lianas, and herbs) have widely different mechanical architectures, which can also vary phenotypically with the environment (Rowe and Speck 2005). In the coastal forests of Kenya, herbs represent the highest percentage of life form with 48.09%, followed by shrubs 19.96%, trees 15.79%, climbers 8.28%, lianas, 6.07%, and epiphytes 1.81% (Table 2).

**Table 2.** Growth habit of the plants of Kenya coastal region.

Habit	No. of species	Percentage
Trees	393	15.79%
Shrubs	497	19.96%
Lianas	151	6.07%
Climbers	206	8.28%
Herbs	1197	48.09%
Epiphytes	45	1.81%
**Total**	**2489**	**100**%

### Species in need of conservation attention

The Red List and Red Data species system is an approach developed by the IUCN for evaluating the conservation status of species, and in particular for identifying and documenting species in need of conservation attention (IUCN 2019). According to this system, 120 species from the coastal forests of Kenya (Table 5), belonging to 39 families and 98 genera were categorized as Extinct in the wild (EW), Critically Endangered (CR), Endangered (EN), Vulnerable (VU), and Near Threatened (NT). Of 120 species recorded, one taxon was recorded as extinct in the wild, 11 were critically endangered, 20 endangered, 52 vulnerable, and 26 near threatened (Table 3). The top three families which had most of its members threatened and near threatened were Acanthaceae 21 species (17.5%), followed by Fabaceae 18 species (14.17%), and Annonaceae 14 species (11.67%) (Table 4).

**Table 3.** IUCN category and number of species representing in each category.

IUCN category	No. of Species	Percentage
Extinct in wild (EW)	1	0.83%
Critically endangered (CR)	11	9.17%
Endangered (EN)	30	25%
Vulnerable (VU)	52	43.33%
Near threatened (NT)	26	21.67%
**Total**	**120**	**100**%

**Table 4.** Families with the largest to the smallest number of threatened and near threatened taxa and their percentage representatives.

**Family**	**No. of species**	**Percentage (%)**
Acanthaceae	21	17.5
Fabaceae	17	14.17
Annonaceae	14	11.67
Rubiaceae	9	7.5
Moraceae	5	4.17
Asphodelaceae	4	3.33
Loranthaceae	4	3.33
Araceae	3	2.5
Asteraceae	3	2.5
Malvaceae	3	2.5
Orchidaceae	3	2.5
Burseraceae	2	1.67
Combretaceae	2	1.67
Euphorbiaceae	2	1.67
Melastomataceae	2	1.67
Meliaceae	2	1.67
Putranjivaceae	2	1.67
Sapotaceae	2	1.67
Anacardiaceae	1	0.83
Apocynaceae	1	0.83
Burmanniaceae	1	0.83
Buxaceae	1	0.83
Canellaceae	1	0.83
Connaraceae	1	0.83
Crassulaceae	1	0.83
Ebenaceae	1	0.83
Gesneriaceae	1	0.83
Lamiaceae	1	0.83
Lythraceae	1	0.83
Menispermaceae	1	0.83
Montiniaceae	1	0.83
Myristicaceae	1	0.83
Picrodendraceae	1	0.83
Rhamnaceae	1	0.83
Rutaceae	1	0.83
Salicaceae	1	0.83
Sapindaceae	1	0.83
Zosteraceae	1	0.83
**Totals**	**120**	**100**

**Table 5.** List of threatened and near threatened plant taxa found in Kenya coastal region.

Family	Species	Life form	IUCN	Version
Crassulaceae	*Kalanchoe fadeniorum* Raadts	Herb	Extinct in the wild	3.1
Acanthaceae	*Justicia drummondii* Vollesen	Herb	Critically Endangered	3.1
	*Megalochlamys tanaensis* Vollesen	Herb	Critically Endangered	3.1
Annonaceae	*Uvaria puguensis* D.M. Johnson	Liana	Critically Endangered	3.1
Burmanniaceae	*Afrothismia baerae* Cheek	Herb	Critically Endangered	3.1
Combretaceae	*Combretum tenuipetiolatum* Wickens	Tree	Critically Endangered	2.3
Euphorbiaceae	*Euphorbia tanaensis* P.R.O. Bally	Tree	Critically Endangered	3.1
Gesneriaceae	Streptocarpus ionanthus subsp. rupicola (B. L. Burtt) Christenh.	Herb	Critically Endangered	3.1
Lamiaceae	*Karomia gigas* (Faden) Verdc.	Tree	Critically Endangered	2.3
Loranthaceae	*Taxillus wiensii* Polhill	Shrub	Critically Endangered	3.1
Malvaceae	*Cola porphyrantha* Brenan	Tree	Critically Endangered	3.1
Rubiaceae	*Vangueriopsis shimbaensis* A.P. Davis & Q. Luke	Tree	Critically Endangered	3.1
Acanthaceae	*Asystasia linearis* S. Moore	Herb	Endangered	3.1
	*Barleria lukei* I. Darbysh.	Herb	Endangered	3.1
	*Barleria maculata* S. Moore	Herb	Endangered	3.1
	*Blepharis kenyensis* Vollesen	Herb	Endangered	3.1
	*Cephalophis lukei* Vollesen	Herb	Endangered	3.1
	*Barleria whytei* S. Moore	Herb	Endangered	3.1
	*Justicia faulknerae* Vollesen	Shrub	Endangered	3.1
	*Justicia breviracemosa* Vollesen	Shrub	Endangered	3.1
	*Monanthotaxis faulknerae* Verdc.	Liana	Endangered	3.1
	*Toussaintia orientalis* Verdc.	Tree	Endangered	3.1
	*Uvariodendron gorgonis* Verdc.	Tree	Endangered	3.1
	*Uvaria faulknerae* Verdc.	Liana	Endangered	3.1
Asteraceae	*Aspilia macrorrhiza* Chiov.	Herb	Endangered	3.1
Asphodelaceae	*Aloe kilifiensis* Christian	Herb	Endangered	3.1
Araceae	*Stylochaeton bogneri* Mayo	Herb	Endangered	3.1
Ebenaceae	*Diospyros shimbaensis* F. White	Tree	Endangered	2.3
Fabaceae	*Newtonia erlangeri* (Harms) Brenan	Tree	Endangered	3.1
	Crotalaria laburnoides var. nudicarpa Polhill	Herb	Endangered	3.1
	*Cynometra lukei* Beentje	Tree	Endangered	3.1
	*Gigasiphon macrosiphon* (Harms) Brenan	Tree	Endangered	2.3
	*Bauhinia mombassae* Vatke	Shrub	Endangered	3.1
	*Dalbergia gloveri* Q. Luke, ined.	Liana	Endangered	3.1
Loranthaceae	*Englerina ramulosa* (Sprague) Polhill & Wiens	Shrub	Endangered	3.1
	*Agelanthus microphyllus* Polhill & Wiens	Shrub	Endangered	3.1
Malvaceae	*Cola octoloboides* Brenan	Tree	Endangered	3.1
Montiniaceae	Grevea eggelingii var. keniensis (Verdc.) Verdc.	Shrub	Endangered	3.1
Orchidaceae	*Habenaria stylites* Rchb.f. & S. Moore	Herb	Endangered	3.1
	*Polystachya teitensis* P.J. Cribb	Epiphytic herb	Endangered	3.1
Rhamnaceae	*Ziziphus robertsoniana* Beentje	Tree	Endangered	3.1
Rubiaceae	*Multidentia sclerocarpa* (K. Schum.) Bridson	Shrub	Endangered	3.1
Acanthaceae	*Barleria usambarica* Lindau	Herb	Vulnerable	3.1
	*Justicia galeata* Hedrén	shrub	Vulnerable	3.1
	*Asystasia minutiflora* Ensermu & Vollesen	Herb	Vulnerable	3.1
	*Barleria maritima* I. Darbysh.	Herb	Vulnerable	3.1
	*Blepharis pratensis* S. Moore	Herb	Vulnerable	3.1
	*Dicliptera inconspicua* I. Darbysh.	Herb	Vulnerable	3.1
	*Anisotes galanae* (Baden) Vollesen	Shrub	Vulnerable	3.1
	*Justicia brevipila* Hedrén	Herb	Vulnerable	3.1
	*Justicia heterotricha* Mildbr.	Herb	Vulnerable	3.1
Annonaceae	*Isolona cauliflora* Verdc.	Tree	Vulnerable	3.1
	*Uvariodendron kirkii* Verdc.	Shrub	Vulnerable	3.1
Annonaceae	*Xylopia arenaria* Engl.	Shrub	Vulnerable	3.1
	*Mkilua fragrans* Verdc.	Shrub	Vulnerable	3.1
Apocynaceae	*Pleioceras orientale* Vollesen	Tree	Vulnerable	3.1
Asteraceae	*Emilia bellioides* (Chiov.) C. Jeffrey	Herb	Vulnerable	3.1
Araceae	*Gonatopus petiolulatus* (Peter) Bogner	Herb	Vulnerable	3.1
Asphodelaceae	*Aloe massawana* Reynolds	Herb	Vulnerable	3.1
	*Aloe ukambensis* Reynolds	Herb	Vulnerable	3.1
Buxaceae	*Buxus obtusifolia* (Mildbr.) Hutch.	Shrub	Vulnerable	3.1
Canellaceae	*Warburgia stuhlmannii* Engl.	Tree	Vulnerable	3.1
Euphorbiaceae	*Pycnocoma littoralis* Pax	Shrub	Vulnerable	2.3
Fabaceae	*Newtonia paucijuga* (Harms) Brenan	Tree	Vulnerable	2.3
	*Sesbania speciosa* Taub.	Herb	Vulnerable	3.1
	*Angylocalyx braunii* Harms	Tree	Vulnerable	3.1
	*Cynometra webberi* Baker f.	Tree	Vulnerable	2.3
	*Dalbergia vacciniifolia* Vatke	Liana	Vulnerable	2.3
	*Diospyros greenwayi* F. White	Tree	Vulnerable	2.3
	*Prioria msoo* (Harms) Breteler	Tree	Vulnerable	2.3
Loranthaceae	*Erianthemum alveatum* (Sprague) Danser	Shrub	Vulnerable	3.1
Lythraceae	Ammannia parkeri var. longifolia (Verdc.) S.A. Graham & Gandhi	Herb	Vulnerable	3.1
Malvaceae	*Hibiscus holstii* Mwachala	Shrub	Vulnerable	3.1
Melastomataceae	*Memecylon verruculosum* Brenan	Shrub	Vulnerable	3.1
	*Warneckea mouririifolia* (Brenan) Borhidi	Shrub	Vulnerable	3.1
Menispermaceae	Cissampelos nigrescens Diels var. nigrescens	Climber	Vulnerable	3.1
Moraceae	Dorstenia tayloriana Rendle var. tayloriana	Herb	Vulnerable	3.1
	*Ficus faulkneriana* C.C. Berg	Tree	Vulnerable	3.1
Myristicaceae	*Cephalosphaera usambarensis* (Warb.) Warb.	Tree	Vulnerable	2.3
Orchidaceae	*Ansellia africana* Lindl.	Epiphytic herb	Vulnerable	3.1
Picrodendraceae	*Aristogeitonia monophylla* Airy shaw	Shrub	Vulnerable	2.3
Putranjivaceae	Drypetes natalensis var. leiogyna Brenan	Shrub	Vulnerable	2.3
	Drypetes usambarica var. trichogyna Radcl.-Sm.	Tree	Vulnerable	2.3
Rubiaceae	*Pavetta linearifolia* Bremek.	Shrub	Vulnerable	3.1
	*Tarenna drummondii* Bridson	Shrub	Vulnerable	2.3
	*Vangueria pallidiflora* (Bullock) Lantz	Shrub	Vulnerable	2.3
	*Afrocanthium kilifiense* (Bridson) Lantz	Shrub	Vulnerable	2.3
	*Pavetta tarennoides* S. Moore	Shrub	Vulnerable	2.3
	*Rothmannia macrosiphon* (K. Schum. ex Engl.) Bridson	Tree	Vulnerable	2.3
Rutaceae	*Vepris sansibarensis* (Engl.) Mziray	Shrub	Vulnerable	2.3
Sapindaceae	*Chytranthus obliquinervis* Radlk. ex Engl.	Tree	Vulnerable	2.3
Sapotaceae	*Mimusops riparia* Engl.	Tree	Vulnerable	3.1
	*Pouteria pseudoracemosa* (J.H. Hemsl.) L.Gaut.	Tree	Vulnerable	2.3
Zosteraceae	*Zostera capensis* Setch.	Herb	Vulnerable	3.1
Acanthaceae	*Chlamydacanthus lindavianus* H. Winkl.	Shrub	Near Threatened	3.1
	*Justicia anisophylla* (Mildbr.) Brummitt	Herb	Near Threatened	3.1
Anacardiaceae	Lannea schweinfurthii var. acutifoliolata (Engl.) Kokwaro	Tree	Near Threatened	2.3
Annonaceae	*Uvaria kirkii* Oliv. ex Hook. f.	Shrub	Near Threatened	3.1
	*Uvaria denhardtiana* Engl. & Diels	Shrub	Near Threatened	3.1
	*Asteranthe asterias* (S. Moore) Engl. & Diels	Shrub	Near Threatened	3.1
	*Huberantha stuhlmannii* (Engl.) Chaowasku	Shrub	Near Threatened	3.1
	*Ophrypetalum odoratum* Diels	Shrub	Near Threatened	3.1
Araceae	*Callopsis volkensii* Engl.	Herb	Near Threatened	3.1
Asteraceae	*Brachylaena huillensis* O. Hoffm.	Tree	Near Threatened	2.3
Asphodelaceae	*Aloe deserti* A. Berger	Shrub	Near Threatened	3.1
Burseraceae	*Commiphora obovata* Chiov.	Shrub	Near Threatened	2.3
Burseraceae	*Commiphora pseudopaolii* J.B. Gillett	Tree	Near Threatened	2.3
Combretaceae	*Conocarpus lancifolius* Engl. & Diels	Tree	Near Threatened	2.3
Connaraceae	*Ellipanthus madagascariensis* (G. Schellenb.) Capuron ex Keraudren	Tree	Near Threatened	2.3
Fabaceae	*Dialium orientale* Baker f.	Shrub	Near Threatened	2.3
	*Dalbergia eremicola* Polhill	Shrub	Near Threatened	3.1
	*Dalbergia melanoxylon* Guill. & Perr.	Shrub	Near Threatened	2.3
	*Erythrina sacleuxii* Hua	Tree	Near Threatened	3.1
Meliaceae	*Lovoa swynnertonii* Baker f.	Tree	Near Threatened	3.1
	*Pseudobersama mossambicensis* (Sim) Verdc.	Tree	Near Threatened	3.1
Moraceae	Dorstenia hildebrandtii Engl. var. hildebrandtii	Herb	Near Threatened	3.1
	*Dorstenia goetzei* Engl.	Herb	Near Threatened	3.1
	*Dorstenia warneckei* Engl.	Herb	Near Threatened	3.1
Rubiaceae	*Coffea pseudozanguebariae* Bridson	Shrub	Near Threatened	3.1
Salicaceae	*Bivinia jalbertii* Tul.	Tree	Near Threatened	2.3

### Endemic species of Kenyan coastal forests

From our results, 551 species were endemic to coastal forests of East Africa. 185 plant species are endemic to Kenya and Tanzania coastal forests and 89 species are only found in Kenya coastal forests (Tables 6, 7). In terms of life forms herbs (44 species, 49.44%) had the highest number of endemic species recorded at the coastal region followed by shrubs (24 species, 26.97%), trees (13 species, 14.6%), climbers and lianas recorded the least numbers of endemic species. (Table 8).

**Table 6.** Number of endemic species in coastal of East Africa forests found in Kenya coastal forests.

**Region**	**No. of endemic species**
Kenyan coastal forests	90
Kenyan and Tanzanian coastal forests	185
East Africa coastal endemic	551

**Table 7.** The endemic plants of Kenya coastal forest.

Family	Species	Life form
Acanthaceae	*Blepharis kenyensis* Vollesen	Herb
	*Dicliptera maculata* subsp. A	Herb
	*Justicia drummondii* Vollesen	Herb
	Justicia sp. aff. fittonioides S. Moore	Herb
	*Justicia* sp. F	Herb
	*Megalochlamys tanaensis* Vollesen	Herb
Amaranthaceae	*Hermbstaedtia gregoryi* C.B. Clarke	Herb
Ancistrocladaceae	*Ancistrocladus robertsoniorum* J.Léonard	Liana
Annonaceae	*Polyceratocarpus* sp. ?nov.	Tree
	*Uvariodendron schmidtii* Q. Luke ined.	Tree
	*Xylopia keniensis* D. M. Johnson	Tree
Apocynaceae	*Echidnopsis ericiflora* Lavranos	Herb
	*Huernia andreaeana* (Rauh) L.C. Leach	Herb
	*Huernia archeri* L.C.Leach	Herb
	Raphionacme sp. cf. jurensis N.E. Br.	Climber
	Ceropegia racemosa var. voiensis Masinde	Herb
Asparagaceae	*Sansevieria ballyi* L.E. Newton	Herb
	*Sansevieria francisii* Chahinian	Herb
	*Sansevieria nitida* Chahinian	Herb
Asteraceae	*Athroisma pusillum* T. Erikss.	Herb
Burmanniaceae	*Afrothismia baerae* Cheek	Herb
Capparaceae	*Maerua mungaii* Beentje	Shrub
	*Thilachium roseomaculatum* Y.B. Harv. & Vollesen	Herb
Caryophyllaceae	*Polycarpaea grahamii* Turrill	Herb
	*Polycarpaea tenuistyla* Turrill	Herb
Commelinaceae	*Aneilema succulentum* Faden	Herb
	*Aneilema tanaense* Faden	Herb
Convolvulaceae	*Merremia* sp. C of FTEA	Herb
Crassulaceae	*Kalanchoe ballyi* Raym.-Hamet ex Cufod.	Herb
	*Kalanchoe fadeniorum* Raadts	Herb
Cucurbitaceae	*Cucumis* sp. (*Oreosyce* sp. A of FTEA)	Climber
	*Zehneria monocarpa* G.W. Hu, V.M. Ngumbau & Q.F. Wang, sp. nov.	Climber
Cyperaceae	*Bulbostylis densecaespitosa* (Lye) R. W. Haines	Herb
	Bulbostylis hispidula subsp. intermedia (Lye) R.W. Haines	Herb
	*Cyperus boreobellus* Lye	Herb
	*Cyperus kwaleensis* Lye	Herb
	*Cyperus microumbellatus* Lye	Herb
Dichapetalaceae	*Dichapetalum fadenii* Breteler	Liana
	*Dichapetalum* sp. ?nov.	Shrub
Euphorbiaceae	*Croton kinondoensis* G.W. Hu, V.M. Ngumbau & Q.F. Wang, sp. nov.	Shrub
	*Euphorbia fluminis* S. Carter	Shrub
	*Euphorbia tanaensis* P.R.O. Bally	Tree
	*Euphorbia taruensis* S. Carter	Herb
	*Erythrococca pentagyna* Radcl.-Sm.	Shrub
Fabaceae	Dichrostachys cinerea subsp. keniensis Brenan & Brummitt	Shrub
	*Aeschynomene* sp. B	Shrub
	*Bauhinia mombassae* Vatke	Shrub
	*Cynometra greenwayi* Brenan	Tree
	*Dalbergia gloveri* Q. Luke, ined.	Liana
	Vigna membranacea subsp. hapalantha (Harms) Verdc.	Herb
	*Crotalaria grata* Polhill	Shrub
	*Abrus* sp. A of FTEA	Herb
Lamiaceae	*Endostemon wakefieldii* (Baker) M. Ashby	Herb
Lamiaceae	*Plectranthus auriglandulosus* A.J.Paton	Herb
Lamiaceae	Leucas tsavoensis var. kilifiensis Sebald	Herb
Loranthaceae	*Taxillus wiensii* Polhill	Shrub
Lythraceae	Ammannia parkeri var. longifolia (Verdc.) S.A. Graham & Gandhi	Herb
Malvaceae	*Cola octoloboides* Brenan	Tree
Montiniaceae	Grevea eggelingii var. keniensis (Verdc.) Verdc.	Shrub
Nyctaginaceae	*Boerhavia* sp A of FTEA	Herb
Ochnaceae	*Ochna* sp. 17 of KTSL	Shrub
Olacaceae	*Strombosiopsis pentamera* Q. Luke, ined.	Tree
Orchidaceae	*Eulophia serrata* P.J. Cribb	Herb
Passifloraceae	*Turnera thomasii* (Urb.) Story	Shrub
Plumbaginaceae	*Plumbago stenophylla* Wilmot-Dear	Herb
Poaceae	*Eleusine semisterilis* S.M.Phillips	Herb
	*Eragrostis* sp. A	Herb
Portulacaceae	*Portulaca coralloides* S.M.Phillips	Herb
Rubiaceae	Tricalysia bridsoniana Robbr. var. bridsoniana	Shrub
	*Keetia lukei* Bridson	Shrub
	*Mitriostigma greenwayi* Bridson	Shrub
	*Pavetta tarennoides* S.Moore	Shrub
	*Psydrax robertsoniae* Bridson	Shrub
	*Psydrax* sp. A of FTEA	Shrub
	*Canthium mrimaense* (Verdc.) Lantz	Shrub
	*Rytigynia* sp. I of FTEA	Shrub
	*Rytigynia* sp. L of FTEA	Tree
	*Spermacoce* sp. B of FTEA	Herb
	*Vangueriopsis shimbaensis* A.P. Davis & Q. Luke	Tree
Rutaceae	*Vepris* sp. (*Diphasia* sp. A of FTEA)	Tree
	*Vepris robertsoniae* Q. Luke, ined.	Tree
	Vepris sp. nr. stolzii I. Verd.	Tree
Salicaceae	*Dovyalis keniensis* E. V. Williams	Shrub
Sapotaceae	*Synsepalum subverticillatum* (E.A. Bruce) T.D.Penn.	Shrub
	Synsepalum sp. cf. subcordatum De Wild.	Tree
Solanaceae	*Solanum malindiense* Voronts.	Shrub
Urticaceae	*Pouzolzia fadenii* Friis & Jellis	Herb
Vitaceae	*Cyphostemma* sp. I of FTEA	Climber
	*Cyphostemma* sp. G	Climber
	*Cyphostemma* sp. L of FTEA	Climber

**Table 8.** Life form representative for endemic species.

Life form	No. of species	Percentage presentation
Climbers	6	5.62%
Herbs	44	49.44%
Lianas	3	3.37%
Shrubs	24	26.97%
Trees	13	14.6%
**Total**	**90**	**100**

### New records

Several new species have been discovered recently from the coastal forests of Kenya, *Adenia angulosa* (Ngumbau et al. 2017), *Dovyalis keniensis* (Williams 2017), *Zehneria monocarpa* (Ngumbau et al. unpublished) and *Croton kinondoensis* (Ngumbau et al. in press) belonging to family Passifloraceae, Salicaceae, Cucurbitaceae and Euphorbiaceae respectively. In addition, one species from family Poaceae, *Panicum peteri*, was collected and recorded for the first time in Kenya.

### Exotic species

A total of 72 introduced, cultivated or naturalized species are recorded in this study belonging to 67 genera and 32 families, for Kenya coastal region (Table 9). The top-rich exotic plant families include: Fabaceae (13 species), Asteraceae (7 species) and Solanaceae (6 species). Out of the 72 plant species, herbs had the highest number of exotic species (35) 48.61%, followed by trees (21) 29.17%, shrubs (12) 16.67% and climbers (4) 5.55%.

**Table 9.** Exotic plants recorded at the coastal region of Kenya.

Family	Species	Growth form
Acanthaceae	*Indoneesiella echioides* (L.) Sreem.	Herb
	*Ruellia tuberosa* L.	Herb
	*Ruspolia hypocrateriformis* (Vahl) Milne-Redh.	Herb
	*Justicia gendarussa* Burm. f.	Herb
Amaranthaceae	*Gomphrena globosa* L.	Herb
	*Amaranthus dubius* Mart. ex Thell.	Herb
	*Atriplex semibaccata* R.Br.	Herb
Anacardiaceae	*Mangifera indica* L.	Tree
	*Anacardium occidentale* L.	Tree
Annonaceae	*Cananga odorata* (Lam.) Hook. f. & Thomson	Tree
Apiaceae	*Foeniculum vulgare* Mill.	Herb
Apocynaceae	*Asclepias curassavica* L.	Herb
Araucariaceae	*Araucaria cunninghamii* Aiton ex D. Don	Tree
Arecaceae	*Areca catechu* L	Herb
	*Cocos nucifera* L.	Tree
Asteraceae	*Synedrella nodiflora* (L.) Gaertn.	Herb
	*Tridax procumbens* L.	Herb
	*Ageratum conyzoides* L.	Herb
	*Acanthospermum hispidum* DC.	Herb
	*Flaveria trinervia* (Spreng.) C. Mohr	Herb
	*Bidens pilosa* L.	Herb
	*Galinsoga parviflora* Cav.	Herb
Bixaceae	*Bixa orellana* L.	Shrub
Cactaceae	*Opuntia ficus-indica* (L.) Mill.	Shrub
Cassuarinaceae	*Casuarina equisetifolia* L.	Tree
Chrysobalanaceae	*Dactyladenia barteri* (Hook. f. ex Oliv.) Prance & F. White	Tree
Combretaceae	*Terminalia catappa* L.	Tree
	*Conocarpus lancifolius* Engl. ex Engl. & Diels	Tree
Cucurbitaceae	*Luffa cylindrica* M. Roem.	Climber
	*Citrullus lanatus* (Thunb.) Matsum. & Nakai	Climber
Euphorbiaceae	*Euphorbia heterophylla* L.	Herb
	*Croton blanchetianus* Baill.	Shrub
Fabaceae	*Centrosema pubescens* Benth.	Climber
	Desmodium adscendens (Sw.) DC. var. adscendens	Herb
	*Senna timoriensis* (DC.) H.S.Irwin & Barneby	Shrub
	*Caesalpinia pulcherrima* (L.) Sw.	Shrub
	*Leucaena leucocephala* (Lam.) de Wit	Shrub
	*Prosopis juliflora* (Sw.) DC.	Shrub
	*Lysiloma latisiliquum* (L.) Benth.	Tree
	*Tamarindus indica* L.	Tree
	*Samanea saman* (Jacq.) Merr.	Tree
	*Senna siamea* (Lam.) H. S. Irwin & Barneby	Tree
	*Parkinsonia aculeata* L.	Tree
	*Pithecellobium dulce* (Roxb.) Benth.	Tree
	Mimosa pudica var. unijuga (Duchass. & Walp.) Griseb.	Herb
Lamiaceae	*Ocimum basilicum* L.	Herb
Lamiaceae	*Mesosphaerum suaveolens* (L.) Kuntze	Herb
Malvaceae	*Corchorus parviflorus* (Benth.) Domin	Herb
	*Gossypium barbadense* L.	Shrub
	*Gossypium hirsutum* L.	Shrub
Melastomataceae	*Clidemia hirta* (L.) D. Don	Shrub
Meliaceae	*Melia azedarach* L.	Tree
	*Toona ciliata* M. Roem.	Tree
Meliaceae	*Azadirachta indica* A.Juss.	Tree
Moringaceae	*Moringa oleifera* Lam.	Tree
Myrtaceae	*Syzygium cumini* (L.) Skeels	Tree
Oxalidaceae	*Oxalis latifolia* Kunth	Herb
	*Oxalis barrelieri* L.	Herb
	*Oxalis corniculata* L.	Herb
Papaveraceae	*Argemone mexicana* L.	Herb
Passifloraceae	*Passiflora foetida* L.	Climber
Piperaceae	*Piper betle* L.	Herb
Sapindaceae	*Sapindus trifoliatus* L.	Tree
Solanaceae	*Lycopersicon esculentum* Mill.	Herb
	*Datura metel* L.	Herb
	*Lycopersicon esculentum* Mill.	Herb
	*Physalis angulata* L.	Herb
	*Physalis minima* L.	Herb
	*Solanum americanum* Mill.	Herb
Talinaceae	*Talinum paniculatum* (Jacq.) Gaertn.	Herb
Verbenaceae	*Lantana camara* L.	Shrub
	*Stachytarpheta urticifolia* Sims	Shrub

### Conclusion

Kenya coastal forests host a large number of species and provide a unique habitat for many species of special concern and endemic plant species. It harbors hundreds of plant species that are only found in coastal forests of East Africa. In addition, they are home to almost half of plant species found in Kenya flora (39.55%), and more than half of species found in the coastal forests of East Africa (65%). Despite this high plant diversity relevance, they have undergone a history of anthropogenic disturbance since the colonial era (Moomaw 1960). Today the rapidly increasing human population along the coast of Kenya demands more agricultural land, more wood products for fuel, house construction, and industry, consequentially threatening the small areas of intact forest and woodland. This therefore, has resulted in large number of threatened and near threatened plants in this region. High conservation measures should be highly taken into action to ensure the protection of large numbers of endemic and threatened species that are at the brink of extinction. Besides, conservation of these relicts will not only provide the protection of species rich diversity of the flora but also of the fauna, including humans which depend on it. Importantly, it will prevent soil erosion and the drying up of moist microhabitats as a result protecting catchment areas and wetlands. This study hence provides not only a comprehensive list of the plant species that have been recorded in this region but also endemic species and species of special concern to guide conservationists and policymakers towards conserving the remaining patches. It also provides a prerequisite to ecology and other thorough work towards understanding plant diversity and conservation of the region.

## Checklist

This checklist is designed for use by anybody interested in the plants of the Kenya coastal region. However, it will be most useful to professionals and students who have some botanical knowledge. Information such as voucher number, habit, habitat, altitude is recorded for each species. Species without voucher numbers were not included in the checklist. Species preceded by “?” means that there is doubt over the correct identification, those preceded with “letter” means that they have not been described and finally vouchers documented as “sr” means that they were sight recorded. EA means the East African Herbarium, Nairobi, Kenya; HIB means the Herbarium of Wuhan Botanical Garden, Wuhan, China, and SAJIT means Sino Africa Joint Investigation Team. The families are divided into four groups, namely lycophytes, monilophytes, gymnosperms, and angiosperms. Families of lycophytes and monilophytes are organized based on the PPG I system (PPG I 2016), while those of gymnosperms are organized based on Christenhusz et al. (2011) and families of angiosperms are organized based on the APG IV system (APG IV 2016).

### Part 1 Lycophytes

**F1. Selaginellaceae**

1 Genus, 3 Species

***Selaginella eublepharis* A. Braun** Habit: Herb. Habitat: Coastal forest and bushland, 0–300 m. Vouchers: Robertson & Luke 5840, Hildebrandt 1961, Drummond & Hemsley 4258, Wamukoya G K7–x936 (EA).

***Selaginella mittenii* Baker** Habit: Herb. Habitat: Wet rocks of forest streams, waterfalls in evergreen forest, ca. 20 m. Voucher: Luke et al. 4521 (EA).

***Selaginella perpusilla* Baker** Habit: Herb. Habitat: In *Hyphaene* grassland in shade of grasses, 7–400 m. Vouchers: Drummond & Hemsley 4034, Faden & Evans 70/441, Rawlins 38, Faden RB 70/441 (EA).

### Part 2 Monilophytes

**F2. Aspleniaceae**

1 Genus, 5 Species

**Asplenium affine var. mettenii (Kuhn) Tardieu** Habit: Herb. Habitat: Forest, ca. 50 m. Voucher: Kresten 29 (EA).

***Asplenium buettneri* Hieron.** Habit: Epiphytic herb. Habitat: Forest, ca. 280 m. Vouchers: Ngumbau V & Mwadime N V016 (EA, HIB), Luke WRQ & Robertson SA 2743B (EA).

***Asplenium erectum* Bory ex Willd.** Habit: Epiphytic herb. Habitat: Forest, ca. 400 m. Voucher: HD van Someren 116 (EA).

***Asplenium nidus* L.** Habit: Epiphytic herb. Habitat: Moist forest, 0–275 m. Vouchers: Ngumbau V & Mwadime N V067 (EA, HIB), Luke et al. 4529 & 4512, Luke & Robertson 2693 (EA).

***Asplenium emarginatum* P. Beauv.** Habit: Herb. Habitat: Moist forest, ca. 40 m. Vouchers: Luke WRQ 1645, Faden & Faden 72/224 (EA).

**F3. Azollaceae**

1 Genus, 1 Species

***Azolla nilotica* Decne. ex Mett.** Habit: Herb. Habitat: River, lake edges, and temporary pools, ca. 10 m. Voucher: Festo L, Luke Q & P 2751 (EA).

**F4. Blechnaceae**

1 Genus, 1 Species

***Stenochlaena tenuifolia* (Desv.) Moore** Habit: Epiphytic herb. Habitat: Swamp forest, ca. 0–140 m. Vouchers: Ngumbau V & Mwadime N V0229 (EA, HIB), Luke WRQ & Mbinda J 6278 (EA).

**F5. Dryopteridaceae**

1 Genus, 1 Species

***Bolbitis auriculata* (Lam.) Alston** Habit: Herb. Habitat: Moist forest, ca. 300 m. Vouchers: Luke WRQ & Robertson SA 2750, Luke 1648 (EA).

**F6. Davalliaceae**

1 Genus, 1 Species

***Davallia denticulata* (Burm. f.) Mett. ex Kuhn** Habit: Epiphyte. Habitat: Bushland on rocks, ca. 2–410 m. Vouchers: Ngumbau V & Mwadime N V0305 (EA, HIB), Robertson SA & Luke WRQ 6046 (EA).

**F7. Dennstaedtiaceae**

2 Genera, 2 Species

***Microlepia speluncae* (L.) T. Moore** Habit: Herb. Habitat: Forests edge, 350–700 m. Voucher: Luke WRQ & PA 8199 (EA).

***Pteridium aquilinum* (L.) Kuhn** Habit: Herb. Habitat: Seasonally wet grassland, 0–150 m. Vouchers: Magogo FC & Glover PE 25, Bally & Smith 14340 (EA).

**F8. Hymenophyllaceae**

2 Genera, 2 Species

***Crepidomanes chevalieri* (Christ) Ebihara & Dubuisson** Habit: Epiphytic herb. Habitat: Moist forest, 150–500 m. Voucher: Faden et al. 70/812 (EA).

***Didymoglossum erosum* (Willd.) J.P. Roux** Habit: Low epiphytic or lithophytic herb. Habitat: Shaded rocks or trunk of trees, ca. 230 m. Voucher: Luke WRQ & Robertson SA 2760 (EA).

**F9. Nephrolepidaceae**

1 Genus, 2 Species

***Nephrolepis acutifolia* (Desv.) Christ** Habit: Herb. Habitat: Rocky cliff or *Acacia*-*Commiphora* bushland, ca. 230 m. Voucher: Luke & Robertson 2530 (EA).

***Nephrolepis biserrata* (Sw.) Schott** Habit: Herb. Habitat: Evergreen forest or streamside forest, ca. 417 m. Vouchers: Ngumbau V & Mwadime N V0322 (EA, HIB), Luke WRQ & Robertson SA 2750, Magogo & Glover 518, Drummond & Hemsley 1221 (EA).

**F10. Ophioglossaceae**

1 Genus, 5 Species

***Ophioglossum costatum* R. Br.** Habit: Herb. Habitat: Dry deciduous woodland, ca. 10 m. Vouchers: Faden & Faden 74/1119, Festo 2731 (EA).

***Ophioglossum latifolium* (Prantl) J. E. Burrows** Habit: Herb. Habitat: In seasonally wet shallow sandy soils and deciduous woodland, ca. 10 m. Vouchers: Faden & Faden 74/1121, Faden RB 70/439B (EA).

***Ophioglossum lusoafricanum* Welw. ex Prantl** Habit: Herb. Habitat: In marshy areas among tall grasses, grassland, and woodland, ca. 150 m. Voucher: Faden 70/440 (EA).

**Ophioglossum nudicaule var. robustum J. S. Licht.** Habit: Herb. Habitat: In shallow sandy soils, dry tropical woodland, and savannah, ca. 10 m. Voucher: Faden & Faden 74/1120 (EA).

***Ophioglossum reticulatum* L.** Habit: Herb. Habitat: Grassland, moist woodland, montane grassland among rocks, and forest margins, ca. 0–167 m. Vouchers: Magogo & Glover 896, Schippers RR 196 (EA).

**F11. Pteridaceae**

8 Genera, 13 Species

***Acrostichum aureum* L.** Habit: Herb. Habitat: Woodland, ca. 253 m. Voucher: SAJIT–005915 (EA, HIB).

***Adiantum capillus-veneris* L.** Habit: Herb. Habitat: Road banks and riverbanks, ca. 200 m. Voucher: Luke WRQ 3969 (EA).

***Adiantum comorense* (Tardieu) Verdc.** Habit: Herb. Habitat: Rainforest, rivulet, road banks, and deep shade, 100–700 m. Vouchers: Gilbert et al. 4964, Magogo & Clover 726, Luke 3600 (EA).

***Adiantum incisum* Forssk.** Habit: Herb. Habitat: Riverside, path sides, and riverine forest, ca. 86 m. Vouchers: Ngumbau V & Mwadime N V0477 (EA, HIB), Kassner 137 (EA).

***Adiantum lunulatum* Burm. f.** Habit: Terrestrial or lithophytic herb. Habitat: Lowland forest or woodland, 160 m. Voucher: Luke WRQ 3147 (EA).

***Ceratopteris thalictroides* (L.) Brongn.** Habit: Herb. Habitat: Swampy areas, swamp forests, marshes, ca. 15 m. Vouchers: SAJIT–006213 & 005485 (EA, HIB), Luke WRQ & PA 4577 (EA).

***Cheilanthes involuta* (Sw.) Schelpe & N. C. Anthony** Habit: Herb. Habitat: Woodland, 90–450 m. Vouchers: SAJIT–006131 (EA, HIB), Napper 2180, Magogo FC & Glover PE 754 (EA).

***Pellaea dura* (Willd.) Hook.** Habit: Herb. Habitat: Forest or woodland, ca. 90 m. Vouchers: Verdcourt 5290, Luke WRQ & Robertson SA 2786A (EA).

***Pityrogramma calomelanos* (L.) Link** Habit: Herb. Habitat: Riverbanks, forest, ca. 150 m. Voucher: Luke 5273 (EA).

***Pteris atrovirens* Willd.** Habit: Herb. Habitat: Shady places, ca. 60 m. Vouchers: Faden RB 226, Luke WRQ 2936 (EA).

***Pteris hamulosa* (Christ) Christ** Habit: Terrestrial herb. Habitat: In shade on wet forest floors, ca. 100 m. Vouchers: Luke WRQ 2937, Luke & Robertson 2729 (EA).

***Pteris tripartita* Sw.** Habit: Terrestrial or epiphytic herb. Habitat: Moist area, ca. 60 m. Voucher: Faden RB 218 (EA).

***Vittaria elongata* Sw.** Habit: Epiphytic or lithophytic herb. Habitat: Low altitude forest, ca. 10–200 m. Vouchers: Robertson SA & Luke WRQ 5168, Magogo & Glover 222, Faden & Evans 70/414, Faden et al. 69/486 (EA).

**F12. Psilotaceae**

1 Genus, 1 Species

***Psilotum nudum* (L.) P. Beauv.** Habit: Herb. Habitat: Dry riverbeds, under cliff overhangs, on rocks, and in rock crevices, 0–100 m. Voucher: Dale in FD 3584 (EA).

**F13. Polypodiaceae**

4 Genera, 5 Species

***Microgramma mauritiana* (Willd.) Tardieu** Habit: Epiphytic herb. Habitat: Rainforest, evergreen thicket, and riverine forest, ca. 30–300 m. Vouchers: SAJIT–004664 & 005433, Ngumbau V & Mwadime N V0178 (EA, HIB), Gillett 18700, Polhill & Paulo 832, Luke WRQ & Robertson SA 2799 (EA).

***Microsorum punctatum* (L.) Copel.** Habit: Epiphytic, lithophytic or terrestrial herb. Habitat: Forest, evergreen thicket or beneath hedges in wet areas, 20–350 m. Vouchers: Verdcourt 3919, Luca, Jeffery & Kirika 230, Faden RB 69/484 (EA).

***Microsorum scolopendria* (Burm. f.) Copel.** Habit: Epiphytic, lithophytic or terrestrial herb. Habitat: Evergreen forest and thicket, 0–297 m. Vouchers: Ngumbau V & Mwadime N V0152 (EA, HIB), Verdcourt 3914, Polhill & Paulo 858, Faden RB 69/488 (EA).

***Platycerium alcicorne* Desv.** Habit: Epiphytic herb. Habitat: Evergreen thickets, 30–200 m. Vouchers: Robertson & Luke 6362, Verdcourt 2408, RB & AJ Faden 72/66 (EA).

***Platycerium angolense* Welw. ex Hook.** Habit: Epiphytic herb. Habitat: Mixed evergreen forest, woodland, ca. 5–150 m. Vouchers: RB & AJ Faden 74/1144, Luke WRQ & Robertson SA 2811 (EA).

**F14. Tectariaceae**

1 Genus, 1 Species

***Tectaria puberula* (Desv.) C. Chr.** Habit: Herb. Habitat: Moist forest, 1–150 m. Vouchers: Faden RB 70/808, Faden RB, Evans A & Msafiri F 952 (EA).

**F15. Thelypteridaceae**

4 Genera, 5 Species

***Chrismatopteris holttumii* N. Quansah & D.S. Edwards** Habit: Herb. Habitat: Forest, 150 m. Voucher: Faden RB 70/422B (EA).

***Christella dentata* (Forssk.) Brownsey & Jermy** Habit: Herb. Habitat: Along stream banks in forest or forest floor, 0–100 m. Voucher: Faden RB 70/419 (EA).

***Christella hispidula* (Decne.) Holttum** Habit: Herb. Habitat: Terrestrial on forest floor away from water or along stream banks in forests, ca. 150 m. Voucher: Luke WRQ & Robertson SA 2710 (EA).

***Cyclosorus interruptus* (Willd.) H. Itô** Habit: Herb. Habitat: Grassland swamps, 0–50 m. Voucher: Luke WRQ & PA 4580 (EA).

**Sphaerostephanos arbuscula subsp. africanus Holttum** Habit: Herb. Habitat: Shady valley, near streams or forest fringing, ca. 300 m. Vouchers: Faden RB 70/413, Drummond RB & Hemsley JH 1203 (EA).

**F16. Lygodiaceae**

1 Genus, 2 Species

***Lygodium kerstenii* Kuhn** Habit: Herb. Habitat: Lowland forests, near sea level. Voucher: Kersten 76 (EA).

***Lygodium microphyllum* (Cav.) R. Br.** Habit: Climber. Habitat: Swampy forest and thicket, ca. 10–200 m. Vouchers: Gardner in FD 1456, Drummond & Hemsley 1208, Robertson SA & Luke WRQ 5146 (EA).

### Part 3 Gymnosperm

**F17. Zamiaceae**

1 Genus, 1 species

***Encephalartos hildebrandtii* A. Braun & Bouché** Habit: Tree. Habitat: Coastal evergreen bushland and lowland forest, ca. 31 m. Vouchers: Mwangangi OM, Waiganjo T & Kamau P 3800 (EA).

### Part 4 Angiosperms

**F18. Acanthaceae**

30 Genera, 108 Species

***Anisotes galanae* (Baden) Vollesen** Habit: Shrub. Habitat: *Acacia* and *Acacia*-*Commiphora* bushland, 25–550 m. Voucher: Robertson 5284 (EA): Vulnerable.

***Anisotes parvifolius* Oliv.** Habit: Shrub. Habitat: *Acacia*-*Terminalia* and *Acacia*-*Commiphora* bushland, 25–650 m. Vouchers: Bally, PRO 16793, Drummond & Hemsley 4265 (EA).

***Anisotes tanensis* Baden** Habit: Shrub. Habitat: Bushland & limestone escarpments, 250–600 m. Voucher: Taylor WE 181 (EA).

***Asystasia ansellioides* C.B. Clarke** Habit: Herb. Habitat: Open grassland or *Hyphaene* grassland, 15–200 m. Vouchers: Luke WRQ & PA 4509, Jeffrey GM 5, Bally 6105, Rawlins 699 (EA).

**Asystasia gangetica subsp. micrantha (Nees) Ensermu** Habit: Herb. Habitat: Forest margin, woodland, wooded grassland, bushland, and sandy seashores, ca. 300 m. Vouchers: Ngumbau V & Mwadime N V0259 (EA, HIB), Jeffrey GW 500, Harvey et al. 54, Robertson & Faulkner 2303 (EA).

***Asystasia guttata* (Forssk.) Brummitt** Habit: Herb. Habitat: Isolated inselberg in ACB, ca. 600 m. Voucher: Luke WRQ 1396 (EA).

***Asystasia leptostachya* Lindau** Habit: Herb. Habitat: Swampy areas of rainforest, ca. 50 m. Voucher: Luke WRQ 1650 (EA).

***Asystasia linearis* S. Moore** Habit: Herb. Habitat: Tall grassland on cotton soil, 25–200 m. Voucher: Gregory s.n. (EA): Endangered.

***Asystasia minutiflora* Ensermu & Vollesen** Habit: Herb. Habitat: Lowland wet evergreen forest, lowland semi-deciduous forest, and thicket, ca. 400 m. Vouchers: Bidgood, Abdallah & Vollesen s.n., Luke 1650, Magogo & Glover 1101 (EA).

***Asystasia moorei* Ensermu** Habit: Herb. Habitat: Lowland evergreen forest, ca. 300 m. Voucher: Luke WRQ & Robertson SA 2703 (EA).

***Asystasia mysorensis* (Roth) T. Anderson** Habit: Herb. Habitat: Lowland forest, ca. 100 m. Vouchers: J Makin 14616, M Hucks 649 (EA).

***Asystasia* sp. B** Habit: Herb. Habitat: Riverine forest, 450 m. Voucher: Battiscombe 250 (EA).

***Avicennia marina* (Forssk.) Vierh.** Habit: Shrub. Habitat: Inland fringes of mangrove associations, sandy dunes, mud of tidal rivers, and salty creeks, sea level. Vouchers: Graham RM 251, Greenway 8933 (EA).

***Barleria acanthoides* Vahl** Habit: Herb. Habitat: Bushland, ca. 690 m. Voucher: Ossent J 18 (EA).

***Barleria argentea* Balf. f.** Habit: Herb. Habitat: Woodland, semi-desert bushland, grassland, disturbed, and eroded areas, ca. 100 m. Voucher: Gillett 19514 (EA).

***Barleria gracilispina* (Fiori) I. Darbysh.** Habit: Herb. Habitat: Woodland, grassland, and degraded areas, ca. 30 m. Voucher: Gillett & Gachathi 20522 (EA).

***Barleria inclusa* I. Darbysh.** Habit: Herb. Habitat: Open woodland, grassland or dry rocky, ca. 400 m. Voucher: Drummond RB & Hemsley 4138 (EA).

***Barleria linearifolia* Rendle** Habit: Herb. Habitat: Bushland and wooded grassland, ca. 30 m. Voucher: Robertson 1816 (EA).

***Barleria lukei* I. Darbysh.** Habit: Climber. Habitat: Forest, ca. 264 m. Vouchers: Ngumbau V & Mwadime N V099 (EA, HIB), Drummond & Hemsley 3976, Magogo & Glover, Robertson & Luke 1955 (EA): Endangered.

***Barleria maculata* S. Moore** Habit: Herb. Habitat: Dry rocky, wooded grassland, ca. 80 m. Vouchers: Drummond & Hemsley 1953, Luke 3492 (EA): Endangered.

***Barleria marginata* Oliv.** Habit: Herb. Habitat: Open bushland and lightly wooded grassland, 100–650 m. Vouchers: Luke, Mbinda & Madudu 6346, Tweedie 2197 (EA).

***Barleria maritima* I. Darbysh.** Habit: Herb. Habitat: Lowland bushland, scrub on coral rag, and along the shoreline, ca. 15 m. Vouchers: Symes 189, Mainwaring 3045, Napier 2301, Tweedie 3165, Rayner 259 (EA).

***Barleria paolioides* I. Darbysh.** Habit: Shrub. Habitat: Open woodland and grassland, 30–850 m. Voucher: Williams Sangai 832 (EA).

**Barleria polhillii I. Darbysh. subsp. polhillii** Habit: Shrub. Habitat: Dry woodland, bushland, grassland, and grazed areas, 0–200 m. Voucher: Luke Q 5472 (EA).

***Barleria ramulosa* C.B. Clarke** Habit: Shrub. Habitat: Dry open bushland and wooded grassland, 6–152 m. Vouchers: SAJIT–005909 (EA, HIB), Curtis 6, Drummond & Hemsley 4082, Luke & Robertson 2567 (EA).

***Barleria repens* Nees** Habit: Shrub. Habitat: Coastal dry forest and lowland woodland, 0–100 m. Vouchers: Graham 2102, Luke & Robertson 2598 (EA).

***Barleria* sp. E** Habit: Herb. Habitat: Bushland, 0–100 m. Vouchers: Rawlins 771, Bally 16859 & 16864 (EA).

***Barleria submollis* Lindau** Habit: Herb. Habitat: Bushland or wooded grassland, ca. 182 m. Vouchers: Ngumbau V & Mwadime N V0349 (EA, HIB), Robertson SA & Luke WRQ 6022, Kassner T 283 (EA).

***Barleria taitensis* S. Moore** Habit: Herb. Habitat: Bushland, grassland, and margin of riverine grassland, ca. 620 m. Vouchers: Bally PRO 16890, Mwadime N 6 (EA).

***Barleria usambarica* Lindau** Habit: Herb. Habitat: Lowland forest margins, bushland, and grassland, 0–600 m. Vouchers: Robertson 6558, RB & AJ Faden 74/1157 (EA): Vulnerable.

***Barleria ventricosa* Hochst. ex Nees** Habit: Herb. Habitat: Woodland and along forest margins, 700 m. Voucher: Luke WRQ & PA 6023 (EA).

***Barleria whytei* S. Moore** Habit: Herb. Habitat: Coastal forest on coral rag, 0–10 m. Vouchers: Drummond & Hemsley 3923, Luke 2907, Luke & Saidi 5829 (EA): Endangered.

***Blepharis kenyensis* Vollesen** Habit: Herb. Habitat: Coastal bushland, 0–150 m. Vouchers: Kimeu JM 532, Graham RM 1630 (EA): Endangered, Endemic.

***Blepharis maderaspatensis* (L.) B. Heyne ex Roth** Habit: Herb. Habitat: Grassland, bushland, woodland, and forest habitats, ca. 95 m. Vouchers: SAJIT–006127 & 004654 (EA, HIB), Robertson SA & Luke WRQ 6028, Jeffrey GM 15 (EA).

***Blepharis pratensis* S. Moore** Habit: Herb. Habitat: Bushland, 0–50 m. Vouchers: Taylor WE s.n., Polhill & Paulo 690, Makin 416, Luke 3064, 3398 & 3562 (EA): Vulnerable.

***Cephalophis lukei* Vollesen** Habit: Herb. Habitat: Wet evergreen lowland forest, 25–400 m. Vouchers: Harvey & Vollens 41, Luke 3377 (EA).

***Chlamydacanthus lindavianus* H. Winkl.** Habit: Herb. Habitat: Coastal forest, 10–250 (–600) m. Vouchers: Ngumbau V & Mwadime N V0421 (EA, HIB), Luke WRQ & Robertson SA 2787, Luke 2941, Luke et al. 6273 (EA): Near Threatened.

***Crabbea velutina* S. Moore** Habit: Herb. Habitat: Woodland, bushland, riverine forests, and thicket, ca. 35 m. Voucher: Luke Q 1485 (EA).

***Crossandra mucronata* Lindau** Habit: Herb. Habitat: Woodland, bushland, upland forests, and dry riverine forest, 50–300 m. Voucher: Whyte A s.n. (EA).

***Crossandra pungens* Lindau** Habit: Herb. Habitat: Evergreen or semi-evergreen coastal forest and thicket, 0–500 m. Vouchers: Festo L, Luke Q & P 2693, Drummond & Hemsley 3755A, Gillespie 312, Rawlins 71 (EA).

***Crossandra stenostachya* (Lindau) C.B. Clarke** Habit: Herb. Habitat: Bushland and riverine forest, ca. 60 m. Vouchers: Faden 74/986, Festo 2283 (EA).

***Dicliptera albicaulis* (S. Moore) S. Moore** Habit: Herb. Habitat: Open *Acacia* woodland, short grassland, roadsides, ca. 800 m. Voucher: Mumiukha 29 (EA).

***Dicliptera heterostegia* Nees** Habit: Herb. Habitat: Coastal riverine forest, forest margins, secondary woodland, and thicket, ca. 300 m. Voucher: Magogo FC & Glover PE 137 (EA).

***Dicliptera inconspicua* I. Darbysh.** Habit: Herb. Habitat: Lowland moist semi-deciduous forest and riverine forest, 0–450 m. Voucher: Festo L, Luke Q & P 2692 (EA): Vulnerable.

***Dicliptera maculata* subsp. A** Habit: Herb. Habitat: Lowland moist forest or roadsides, ca. 300 m. Vouchers: Luke 2922, Luke stone & Baer 8243 (EA): Endemic.

***Duosperma crenatum* (Lindau) P.G. Mey.** Habit: Shrub. Habitat: Deciduous woodland and scrub, ca. 800 m. Voucher: Bally PRO 16824 (EA).

***Duosperma grandiflorum* Vollesen** Habit: Shrub. Habitat: Bushland, woodland, and wooded grassland, ca. 300 m. Voucher: Mungai et al. 239/83 (EA).

***Dyschoriste hildebrandtii* (S. Moore) S. Moore** Habit: Shrub. Habitat: Woodland, wooded grassland, and bushland, ca. 100 m. Vouchers: Drummond & Hemsley 4124, Robertson SA & Luke WRQ 5981 (EA).

***Ecbolium amplexicaule* S. Moore** Habit: Herb. Habitat: Forest, thicket, riverine forest, and sisal plantations, 0–400 m. Vouchers: Luke Q 1465, Bally 13065, Robertson 5902, Drummond & Hemsley 3921 (EA).

**Ecbolium subcordatum C.B. Clarke var. subcordatum** Habit: Herb. Habitat: Lowland *Acacia* and *Acacia*-*Commiphora* bushland, riverbanks, and river-beds, ca. 182 m. Vouchers: Bally PRO 16757, Luke et al. 644, Greenway 9818 (EA).

**Ecbolium subcordatum var. glabratum Vollesen** Habit: Herb. Habitat: Low bushland and riverbanks, 60–152 m. Vouchers: Bally PRO 16819, Luke et al. TPR 387 (EA).

***Ecbolium viride* (Forssk.) Alston** Habit: Herb. Habitat: Bushland, riverbeds, and coastal bushland on sand dunes, 0–50 m. Voucher: Faden 74/1114 (EA).

***Elytraria acaulis* (L.f.) Lindau** Habit: Herb. Habitat: Alluvial grassland with *Acacia*-*Combretum*-*Dobera* thicket, 25–800 m. Vouchers: Greenway & Rawlins 9460, Faden 74/1059 (EA).

***Elytraria minor* Dokosi** Habit: Herb. Habitat: Evergreen and semi-deciduous forest, secondary thickets, and cultivated areas, 15–450 (–650) m. Vouchers: SAJIT–006026 (EA, HIB), Greenway PJ & Rawlins SP 9366, Robertson SA & Luke WRQ 5986, Rawlins 34, Faden 71/798 (EA).

***Hygrophila auriculata* (Schumach.) Heine** Habit: Herb. Habitat: Seasonal pools or other stagnant water bodies, ca. 156 m. Vouchers: SAJIT–005982, Ngumbau V & Mwadime N V0435 (EA, HIB), Magogo FC & Glover PE 528, Drummond & Hemsley 3927 (EA).

**Hypoestes forskaolii subsp. hildebrandtii (Lindau) I. Darbysh.** Habit: Herb. Habitat: Dry grassland and open bushland, ca. 382 m. Vouchers: SAJIT–006066, Ngumbau V & Mwadime N V0365 (EA, HIB), Magogo FC & Glover PE 138 (EA).

***Indoneesiella echioides* (L.) Sreem.** Habit: Herb. Habitat: Bushland and grassland, ca. 2 m. Voucher: Ngumbau V & Mwadime N V0518 (EA, HIB): Exotic.

***Justicia anisophylla* (Mildbr.) Brummitt** Habit: Shrubby herb. Habitat: Lowland and semi-deciduous forest, ca. 200 m. Vouchers: Faden 77/421, Luke 29142 (EA): Near Threatened.

***Justicia baravensis* C.B. Clarke** Habit: Herb. Habitat: Sandy foreshores, coastal bushland, 0–15 m. Vouchers: Robertson & Brummit 6763, Kokwaro 3900 (EA).

***Justicia betonica* L.** Habit: Herb. Habitat: Grassland, bushland, and woodland, ca. 253 m. Vouchers: Ngumbau V & Mwadime N V0121 (EA, HIB), Robertson SA & Luke WRQ 5979 (EA).

***Justicia brevipila* Hedrén** Habit: Herb. Habitat: Bushland and grassland, 25–350 m. Vouchers: Luke et al. TRP785, Robertson & Luke 5728 (EA): Vulnerable.

***Justicia breviracemosa* Vollesen** Habit: Shrub. Habitat: Lowland forest and riverine forest, 10–250 m. Voucher: Luke 4271 (EA): Endangered.

***Justicia caerulea* Forssk.** Habit: Herb. Habitat: Grassland, bushland, riverine, and scrub, ca. 140 m. Vouchers: Thomas 38, RB & AJ Faden 74/985 (EA).

***Justicia calyculata* Deflers** Habit: Herb. Habitat: Woodland, bushland, and grassland, ca. 25 m. Vouchers: Robertson SA & Luke WRQ 6039, Luke 3355 (EA).

***Justicia capensis* Thunb.** Habit: Herb. Habitat: Forest or forest grassland, 5–715 m. Vouchers: Luke & Robertson 1810 & 2029 (EA).

***Justicia debilis* (Forssk.) Vahl** Habit: Herb. Habitat: Forest, ca. 186 m. Vouchers: Ngumbau V & Mwadime N V0336 (EA, HIB), Festo L & Luke Q 2436 (EA).

***Justicia drummondii* Vollesen** Habit: Herb. Habitat: Lowland forest, 75–200 m. Vouchers: Drummond & Hemsley 3792, Robertson & Luke 5985, Luke 4508 (EA): Critically Endangered, Endemic.

***Justicia engleriana* Lindau** Habit: Shrub. Habitat: Lowland to low montane evergreen forest, ca. 372 m. Vouchers: SAJIT–006150 (EA, HIB), Mbinda J & Luke WRQ 259, Magogo & Glover 139 (EA).

***Justicia faulknerae* Vollesen** Habit: Shrub. Habitat: Semi-deciduous lowland forest, 150–450 m. Vouchers: Luke 1590, Luke & Robertson 1819 (EA): Endangered.

***Justicia fittonioides* S. Moore** Habit: Herb. Habitat: Dry or semi-deciduous lowland forest, ca. 240 m. Vouchers: Ngumbau V & Mwadime N V107 (EA, HIB), Luke WRQ 1651, Robertson 6560 (EA).

***Justicia flava* (Forssk.) Vahl** Habit: Herb. Habitat: Forest margin and clearings, woodland, grassland, and bushland, (25–) 350 m. Vouchers: SAJIT–006110 (EA, HIB), Festo L & Luke WRQ 2443 (EA).

***Justicia galeata* Hedrén** Habit: Herb. Habitat: Forest and shrubland, ca. 274 m. Voucher: Dale IR 1077 (EA).

***Justicia gendarussa* Burm. f.** Habit: Shrub. Habitat: Abandoned gardens, ca. 0–300 m. Vouchers: Dale 2015, Reitsma 448 (EA): Naturalized.

***Justicia heterotricha* Mildbr.** Habit: Herb. Habitat: Forest, ca. 240 m. Vouchers: Luke & Robertson 2770, Luke WRQ 2931 (EA): Vulnerable.

***Justicia inaequifolia* Brummitt** Habit: Shrub. Habitat: Evergreen or semi deciduous lowland forest or coral rag thicket, ca. 420 m. Vouchers: SAJIT–006088 (EA, HIB), Luke WRQ & Robertson SA 2699, BR Adams 87, Luke 1828, Magogo & Glover 136, Robertson SA & Polhill 4833 (EA).

***Justicia nyassana* Lindau** Habit: Herb. Habitat: Lowland forest, riverine forest, and woodland, ca. 100 m. Voucher: Drummond & Hemsley 3872 (EA).

***Justicia odora* (Forssk.) Lam.** Habit: Herb. Habitat: Dry wooded grassland, open woodland, and scrub, 0–300 m. Voucher: Field CR 59 (EA).

***Justicia pseudorungia* Lindau** Habit: Shrub. Habitat: Lowland to montane evergreen forest, and riverine forest, 15 m. Vouchers: SAJIT–006220 (EA, HIB), Taylor WE s.n., Robertson 5085, Faden & Evans 71/736 (EA).

***Justicia scandens* Vahl** Habit: Herb. Habitat: Forest, bushland, and lake shores, ca. 50 m. Voucher: Luke & Robertson 2690 (EA).

**Justicia schimperiana subsp. campestris Vollesen** Habit: Shrub. Habitat: Bushland and dry riverine forest, ca. 35 m. Vouchers: Luke Q 1521, Robertson & Luke 5328, Drummond & Hemsley 4119 (EA).

**Justicia sp. aff. fittonioides S. Moore.** Habit: Herb. Habitat: Forest, ca. 150 m. Voucher: Luke WRQ & Robertson SA 5985 (EA): Endemic.

***Justicia* sp. F of FTEA** Habit: Herb. Habitat: Lowland forest, 200–300 m. Voucher: Robertson & Luke 5989 (EA): Endemic.

***Justicia stachytarphetoides* (Lindau) C.B. Clarke** Habit: Shrub. Habitat: Lowland forest, riverine forest, thicket, and swamp forest, 5–400 m. Vouchers: MG Gilbert et al. 5909, Robertson & Luke 5337, BR Adams 78 (EA).

***Justicia striata* (Klotzsch) Bullock** Habit: Herb. Habitat: Lowland to montane forest, ca. 239 m. Vouchers: Ngumbau V & Mwadime N V0114 & 0354 (EA, HIB), Magogo FC & Glover PE 878, Drummond & Hemsley 3829 (EA).

***Lankesteria alba* Lindau** Habit: Herb. Habitat: Forest, 25–500 m. Vouchers: SAJIT–006186 & 005933 (EA, HIB), Luke WRQ 870, Greenway & Rawlins 9351, Luke & Robertson 2623, Drummond & Hemsley 1126 (EA).

**Megalochlamys revoluta (Lindau) Vollesen subsp. revoluta** Habit: Shrubby herb. Habitat: Bushland or grassland thicket, ca. 250 m. Voucher: Bally PRO 16778 (EA).

***Megalochlamys tanaensis* Vollesen** Habit: Shrubby herb. Habitat: Lowland forest, ca. 25 m. Vouchers: Gillett et al. 19922, Luke 10727 (EA): Critically Endangered, Endemic.

***Megalochlamys trinervia* (C.B. Clarke) Vollesen** Habit: Shrubby herb. Habitat: Woodland or bushland, 25–200(–400) m. Vouchers: Brenan et al. 14774, Greenway & Rawlins 9459, Bally PRO16750, Luke 10726, Luke TPR247 (EA).

***Monothecium aristatum* (Nees) T. Anderson** Habit: Herb. Habitat: Lowland and medium altitude wet evergreen forest or swampy forest, ca. 200 m. Vouchers: Greenway & Rawlins 9358, K & L Holm 8, Robertson & Luke 5970, BR Adams 86 (EA).

***Phaulopsis gediensis* Mankt.** Habit: Herb. Habitat: Lowland forest, thicket, and shrubland, 0–500 m. Vouchers: Robertson SA 6575, Luke WRQ 3968, Drummond & Hemsley 1091, Luke et al. 3372 (EA).

***Pseuderanthemum hildebrandtii* Lindau** Habit: Shrub. Habitat: Coastal lowland, medium altitude forest, thicket, and bushland, ca. 199 m. Vouchers: Ngumbau V & Mwadime M V0402 (EA, HIB), Meester V de 336, Magogo FC & Glover PE 721 (EA).

***Pseuderanthemum subviscosum* (C.B. Clarke) Stapf** Habit: Herb. Habitat: Forest, riverine forest, and bushland, ca. 365 m. Vouchers: Luke 3947, Glover et al. 1154, Luke WRQ 886, Magogo & Glover 75 (EA).

**Rhinacanthus dichotomus (Lindau) I. Darbysh. var. dichotomus** Habit: Herb. Habitat: Bushland, coastal thicket, margins of cultivation, and roadsides, ca. 199 m. Vouchers: Ngumbau V & Mwadime N V0481 (EA, HIB), Festo L, Luke Q & P 2694 (EA).

***Rhinacanthus pulcher* Milne-Redh.** Habit: Herb. Habitat: Bushland, 225–552 m. Voucher: Gillett 21100 (EA).

***Rhinacanthus rotundifolius* C.B. Clarke** Habit: Herb. Habitat: Bushland, 0–350 m. Vouchers: Gillett 19527, Luke et al. in TPR 640 (EA).

***Ruellia amabilis* S. Moore** Habit: Herb. Habitat: Lowland and riverine forest, 25–450 m. Voucher: Gillett & Kibuwa 19949 (EA).

***Ruellia patula* Jacq.** Habit: Herb. Habitat: Bushland, woodland, and riverine forest, 60–400 m. Vouchers: Harvey & Vollens 52, Curtis A 13, Magogo & Glover 274 (EA).

***Ruellia prostrata* Poir.** Habit: Herb. Habitat: Grassland, bushland, and woodland, ca. 10 m. Vouchers: Luke et al. TRP775, M Robertson 6363 (EA).

***Ruellia tuberosa* L**. Habit: Herb. Habitat: Roadside or secondary grassland, ca. 150 m. Vouchers: Luke PA & WRQ 3076, Napier 6195, Luke 3076 (EA): Exotic.

***Ruspolia hypocrateriformis* (Vahl) Milne-Redh.** Habit: Shrub. Habitat: Bushland, 0–100 m. Voucher: Bally 13702 (EA).

***Sclerochiton boivinii* (Baill.) C.B. Clarke** Habit: Shrub. Habitat: Moist forest, 0–400 m. Vouchers: SAJIT–006181 & 005546, Ngumbau V & Mwadime N V012 (EA, HIB), Luke WRQ 891, Dale 3664, Drummond & Hemsley 1143, Polhill & Robertson 4795 (EA).

**Sclerochiton vogelii (Nees) T. Anderson subsp. holstii (Lindau) Napper** Habit: Shrub. Habitat: Sub-tropical and tropical moist lowland, 0–500 m. Vouchers: SAJIT–004648, Ngumbau V & Mwadime N V022 (EA, HIB).

***Thunbergia alata* Bojer ex Sims** Habit: Climber. Habitat: Dry bushland to disturbed and secondary vegetation, ca. 15 m. Vouchers: SAJIT–006206 (EA, HIB), Magogo FC & Glover PE 310, Fabus 23 (EA).

***Thunbergia kirkii* Hook. f.** Habit: Shrub. Habitat: Evergreen, semi-evergreen lowland, and lower montane forest, ca. 152 m. Vouchers: SAJIT–005541 (EA, HIB), Luke WRQ 1628, Gardner 1407, Magogo & Glove 619, Robertson & Luke 6454 (EA).

***Thunbergia schimbensis* S. Moore** Habit: Herb. Habitat: Woodland or wooded grassland, ca. 297 m. Vouchers: Ngumbau V & Mwadime N V0157 (EA, HIB), Luke Q 1487, Drummond & Hemsley 4085, Verdcourt 1934 (EA).

***Thunbergia stelligera* Lindau** Habit: Climber. Habitat: Lowland forest, 100–650 m. Vouchers: Luke WRQ 3115, Luke & Robertson 2645 (EA).

***Trichaulax mwasumbii* Vollesen** Habit: Herb. Habitat: Woodland or coastal thicket, 50–200 m. Vouchers: Ngumbau V & Mwadime N V017 (EA, HIB), Verdcourt 3607, Faden 71/800, Luke 1947 (EA).

***Whitfieldia elongata* (P. Beauv.) De Wild. & T. Durand** Habit: Shrub. Habitat: Evergreen lowland and lower montane forest, ca. 150 m. Vouchers: Mwadime N 16, Luke WRQ 888, Drummond & Hemsley 1146, Bally 13711, Faden RB 70/809 (EA).

***Whitfieldia orientalis* Vollesen** Habit: Shrub. Habitat: Evergreen lowland forest, 220–370 m. Vouchers: Faden 71/767, Luke 893B, Luke 4729 (EA).

**F19. Achariaceae**

3 Genera, 3 Species

***Lindackeria bukobensis* Gilg** Habit: Shrub. Habitat: Forest or forest edge, often in open mixed forest, ca. 50 m. Voucher: J Adanson 271 (EA).

***Rawsonia lucida* Harv. & Sond.** Habit: Tree. Habitat: Lowland and upland rainforest, dry evergreen forest, semi-swamp, and riverine forest, ca. 237 m. Vouchers: SAJIT–006027, Ngumbau V & Mwadime N V0168 (EA, HIB), Luke WRQ & Robertson SA 217 & 4821(EA).

**Xylotheca tettensis (Klotzsch) Gilg var. kirkii** (**Oliv.) Wild** Habit: Shrub. Habitat: Lowland woodland, bushland, and wooded grassland, 0–700 m. Vouchers: Ngumbau V & Mwadime N V0218 & 0542 (EA, HIB), Dale in FD 3879, Luke 3083 (EA).

**F20. Aizoaceae**

3 Genera, 5 Species

***Sesuvium portulacastrum* (L.) L.** Habit: Herb. Habitat: Maritime shores, coastal dunes, and beaches, 0–200 m. Vouchers: Luke Q 6147, RM Graham 2221, Luke PA & WRQ 5648 (EA).

***Trianthema ceratosepala* Volkens & Irmsch** Habit: Herb. Habitat: Bushland, 135 m. Voucher: Bally 16715 (EA).

***Trianthema portulacastrum* L**. Habit: Herb. Habitat: Cultivated ground, 0–250 m. Vouchers: Linder 2634, Verdcourt B 3617 (EA).

***Trianthema triquetra* Willd.** Habit: Herb. Habitat: Grassland, bushland, woodland, and saline sands, ca. 5 m. Vouchers: Rawlins SP 828, Drummond & Hemsley 4090, Luke Q 6147 (EA).

***Zaleya pentandra* (L.) C. Jeffrey** Habit: Herb. Habitat: Woodland, bushland or grassland, ca. 30 m. Vouchers: Reitsma J 413, Drummond & Hemsley 1046, Muchiri J 490 (EA).

**F21. Alismataceae**

2 Genera, 2 Species

***Burnatia enneandra* Micheli** Habit: Herb. Habitat: Edges of slow flowing rivers and streams, shallow lakes, waterholes, and swamps, ca. 5 m. Voucher: Mwadime N 2573 (EA).

***Limnophyton obtusifolium* (L.) Miq.** Habit: Herb. Habitat: Swamps & pools, 15 m. Vouchers: Luke Q 5635, RM Graham 2164, Hildebrandt 1912 (EA).

**F22. Amaranthaceae**

20 Genera, 38 Species

**Achyranthes aspera L. var. aspera** Habit: Herb. Habitat: Disturbed areas, ca. 0–240 m. Vouchers: Brown ES 1412, Robertson SA 3799, Hooper & Townsend 1163 (EA).

**Achyranthes aspera var. sicula** L. Habit: Herb. Habitat: Disturbed areas, ca. 0–300 m. Voucher: Magogo FC & Glover PE 153 (EA).

***Aerva lanata* (L.) Juss. ex Schult.** Habit: Herb. Habitat: Cultivated area, disturbed area, bushland, and grassland, ca. 186 m. Vouchers: SAJIT–006137, Ngumbau V & Mwadime N V0347 (EA, HIB), Luke WRQ 5468, Magogo FC & Glover PE 924, MacNaughton 96 (EA).

***Allmaniopsis fruticulosa* Suess.** Habit: Herb. Habitat: Bushland, 110–200 m. Voucher: Gillett 16528 (EA).

***Alternanthera pungens* Kunth** Habit: Herb. Habitat: Roadsides, grassland, ca. 0–200 m. Vouchers: Jeffery GW 522, Rawlins 839 (EA).

***Alternanthera sessilis* (L.) R.Br. ex DC.** Habit: Herb. Habitat: Damp forests and abandoned cultivation, ca. 0–300 m. Vouchers: Magogo FC & Glover PE 110, Drummond & Hemsley 4174 (EA).

***Amaranthus cruentus* L.** Habit: Herb. Habitat: Cultivated lands, forest margins, ca. 20 m. Vouchers: Wakefield, Jeffery GW 431 (EA).

***Amaranthus dubius* Mart. ex Thell.** Habit: Herb. Habitat: Cultivated area, waste places, roadsides, and grassland, ca. 0–50 m. Voucher: Jeffery GW 441 (EA): Naturalized.

**Amaranthus graecizans subsp. silvestris (Vill.) Brenan** Habit: Herb. Habitat: Cultivated ground, grassland, ca. 0–350 m. Voucher: Luke WRQ & PA 5694 (EA).

***Amaranthus spinosus* L.** Habit: Herb. Habitat: Cultivated ground ca. 2 m. Voucher: Mwadime N & Chesire C 256 (EA).

***Amaranthus viridis* L.** Habit: Herb. Habitat: Cultivated and disturbed areas, ca. 2 m. Voucher: Sturrock 2008 B (EA).

**Atriplex farinosa subsp. keniensis (Brenan) Friis & M.G. Gilbert** Habit: Shrub. Habitat: In sand at or near high tide mark. Vouchers: Festo L, Luke Q & P 2794, Graham 2166 (EA).

***Atriplex semibaccata* R.Br.** Habit: Herb. Habitat: Cultivated area, ca. 10 m. Voucher: Kennox M 121 (EA): Introduced.

***Celosia argentea* L.** Habit: Herb. Habitat: Cultivated lands, grassland, rivers, ca. 610 m. Vouchers: Weruer 993, Greenway & Rawlins 9461 (EA).

***Celosia hastata* Lopr.** Habit: Herb. Habitat: Bushland and woodland, 3–106 m. Vouchers: Ngumbau V & Mwadime N V0482 (EA, HIB), Festo L & Luke Q 2675, Magogo FC & Glover PE 914 (EA).

***Celosia schweinfurthiana* Schinz** Habit: Herb. Habitat: Lowland forest, clearings, especially near water, ca. 0–50 m. Vouchers: Robertson SA & Luke 5859, Magogo FC & Glover PE 915 (EA).

***Celosia stuhlmanniana* Schinz** Habit: Herb. Habitat: Shaded places, forest, and scrub, ca. 60 m. Voucher: Nyange M 508 (EA).

***Celosia trigyna* L.** Habit: Herb. Habitat: Cultivated lands or forest clearings, 0–100 m. Vouchers: Jeffery GW 445, McCrag in CM 9170 (EA).

***Centemopsis gracilenta* (Hiern) Schinz** Habit: Herb. Habitat: Woodland or cultivated lands, ca. 50 m. Voucher: Rawlins 776 (EA).

***Centrostachys aquatica* (R.Br.) Moq.** Habit: Herb. Habitat: Pools, waterholes, rivers, and lake shores, ca. 20 m. Voucher: Hooper & Townsend 1227 (EA).

***Cyathula braunii* Gilg ex Schinz** Habit: Herb. Habitat: Forest undergrowth, ca. 360 m. Vouchers: SAJIT–005931, Ngumbau V & Mwadime N V0368 (EA, HIB), Gillett 18714, Luke WRQ et al. 3328 (EA).

***Cyathula coriacea* Schinz** Habit: Herb. Habitat: Thicket, woodland, and roadside, 30–300 m. Voucher: Greenway 8864 (EA).

**Cyathula prostrata (L.) Blume var. prostrata** Habit: Herb. Habitat: Deciduous to evergreen vine thickets, ca. 304 m. Vouchers: SAJIT–006155, Ngumbau V & Mwadime N V0294 (EA, HIB), Luke WRQ 4714 (EA).

**Digera muricata (L.) Mart. subsp. muricata** Habit: Herb. Habitat: Waste ground, grassland, and bushland, ca. 150 m. Vouchers: RM Graham in FD 648 & 1993 (EA).

***Gomphrena celosioides* Mart.** Habit: Herb. Habitat: Roadsides, coastal grassland, and cultivated area, ca. 50–300 m. Vouchers: Festo L, Luke Q & P 2703, Hooper & Townsend 1168, Robertson SA 3480 (EA).

***Gomphrena globosa* L.** Habit: Herb. Habitat: On grassland, ca. 100 m. Vouchers: Jeffery GW 468, WE Taylor (EA): Naturalized.

***Hermbstaedtia gregoryi* C.B. Clarke** Habit: Herb. Habitat: Sand dune, 20 m. Vouchers: Frazier J 1101, Moomaw 1562 (EA): Endemic.

***Neocentema alternifolia* (Schinz) Schinz** Habit: Herb. Habitat: Roadside or cultivation area, 0–50 m. Voucher: Kabuye CHS, Gilbet VC & Robertson 43 (EA).

***Nothosaerva brachiata* (L.) Wight** Habit: Herb. Habitat: Waste ground, grassland, and bushland, ca. 300 m. Vouchers: Thuiru 78, Luke WRQ 3501 (EA).

***Psilotrichum cyathuloides* Suess. & Launert** Habit: Herb. Habitat: Forests, 0–150 m. Voucher: Rawlins 394 (EA).

***Psilotrichum fallax* C.C. Towns.** Habit: Herb. Habitat: Forests on limestone, 0–380 m. Vouchers: Ngumbau V and Mwadime N V109 (EA, HIB), Magogo & Glover 4141, Adams 71, Herbarium Techniques Course III 078 (EA).

***Psilotrichum gnaphalobryum* (Hochst.) Schinz** Habit: Herb. Habitat: Open *Acacia* and *Acacia*-*Commiphora* bushland, 140–790 m. Voucher: Luke PA, WRQ et al. 3833 (EA).

***Psilotrichum majus* Peter** Habit: Herb. Habitat: Forest undergrowth and margins, ca. 285 m. Vouchers: Ngumbau V & Mwadime N V0122 (EA, HIB), Luke PA, WRQ 4306, Magogo FC & Glover PE 79 & 261 (EA).

***Psilotrichum scleranthum* Thwaites** Habit: Herb. Habitat: Forest margin, grassland, and coconut plantation, ca. 417 m. Vouchers: Ngumbau V & Mwadime N V0318 (EA, HIB), Luke Q & Robertson SA 5495, Ross KS 142 (EA).

***Psilotrichum sericeum* (J. Koenig ex Roxb.) Dalzell** Habit: Herb. Habitat: Abandoned land, grassland, and bushland, 15–420 m. Vouchers: Robertson SA 3801, Pakia M 113, GM Mungai & SM Rucina 307 (EA).

**Pupalia lappacea var. argyrophylla C.C. Towns.** Habit: Herb. Habitat: Coastal bushland, grassland, and cultivated area, 0–530 m. Vouchers: SAJIT–006184, Ngumbau V & Mwadime N V0319 (EA, HIB), Festo L & Luke Q 2525, Magogo & Glover 125, Moggridge 157, Gillespie 383 (EA).

**Pupalia lappacea var. velutina (Moq.) Hook.f.** Habit: Herb. Habitat: Woodland, grassland, thickets or roadsides, ca. 200 m. Vouchers: Luke WRQ & Robertson SA 1528, Luke WRQ 3327 (EA).

***Salsola africana* (Brenan) Botsch.** Habit: Herb. Habitat: Semi-desert scrub, ca. 90 m. Voucher: Battiscombe 223 (EA).

***Tecticornia indica* (Willd.) K. Sheph. & Paul G. Wilson** Habit: Herb. Habitat: Salt marshes by the sea and mangrove swamps, ca. 5 m. Vouchers: Gillett JB 20439, Luke Q 6149 (EA).

***Volkensinia prostrata* (Volkens ex Gilg) Schinz** Habit: Herb. Habitat: Bushland or grassland, ca. 180 m. Voucher: Gillett 19505 (EA).

**F23. Amaryllidaceae**

4 Genera, 5 Species

***Crinum politifolium* R. Wahlstr.** Habit: Herb. Habitat: Riverine vegetation or damp places, ca. 86 m. Vouchers: Ngumbau V & Mwadime N V0480 (EA, HIB), Luke WRQ & PA 4581 (EA).

**Crinum stuhlmannii Baker subsp. stuhlmannii** Habit: Herb. Habitat: Grassland or wooded grassland, ca. 0–300 m. Vouchers: Magogo & Glover 468, Jeffery & Kirika 251, Graham K. 1844 (EA).

***Cryptostephanus haemanthoides* Pax** Habit: Herb. Habitat: Deciduous bushland, ca. 20 m. Vouchers: Luke TPR 574, Robertson SA 5371D (EA).

**Cyrtanthus sanguineus subsp. wakefieldii (Sealy) Nordal** Habit: Herb. Habitat: Woodland, ca. 0–250 m. Vouchers: Walker in Bally 7406, Wakefield 42 (EA).

**Scadoxus multiflorus (Martyn) Raf. subsp. multiflorus** Habit: Herb. Habitat: Grassland, riverine vegetation, coastal rocks or montane vegetation, ca. 0–350 m. Voucher: Magogo FC & Glover PE 367 (EA).

**F24. Anacardiaceae**

7 Genera, 13 Species

***Anacardium occidentale* L.** Habit: Tree. Habitat: Cultivated area, abandoned cultivated areas, ca. 250 m. Vouchers: RE Perdue & SP Kibuwa 10116, Napier 3274, R Karisa 24 (EA): Naturalized.

***Lannea alata* (Engl.) Engl.** Habit: Tree. Habitat: Bushland and coastal forests edges, ca. 88–450 m. Vouchers: SAJIT–006442 (EA, HIB), Dale IR 3885, Polhill & Paulo 598, Luke WRQ 950 (EA).

***Lannea cotoneaster* (Chiov.) Sacleux** Habit: Shrub. Habitat: Bushland, 75–600 m. Voucher: Njoroge Thairu 86 (EA).

**Lannea schweinfurthii var. acutifoliolata (Engl.) Kokwaro** Habit: Tree. Habitat: Coastal forests, 1–460 m. Vouchers: SAJIT–004658 & 004663, Ngumbau V & Mwadime N V0361 & 0333 (EA, HIB) Greenway 9799, RM Graham in FD 1784, Gilbert MG & Kuchar P 5866, Luke WRQ & Robertson SA 2775 (EA): Near Threatened.

**Lannea schweinfurthii var. stuhlmannii (Engl.) Kokwaro** Habit: Tree. Habitat: Woodland, bushland, dry forests, and river valleys, ca. 20–450 m. Vouchers: Drummond & Hemsley 4166, Luke WRQ 920, Robertson SA 3498 (EA).

***Lannea triphylla* (Hochst. ex A. Rich.) Engl.** Habit: Tree. Habitat: Grassland, and bushland, ca. 70 m. Vouchers: Drummond & Hemsley 4102, JB Gillet 16486 (EA).

**Lannea welwitschii var. ciliolata Engl.** Habit: Tree. Habitat: Lowland rainforest, riverine forest, and mountain slopes, 110–450 m. Vouchers: Verdcourt in EAH 11322, Magogo & Glover 177, Faden & Faden 74/318 (EA).

***Mangifera indica* L.** Habit: Tree. Habitat: Cultivated area, abandoned cultivated areas, ca. 100 m. Voucher: Bally 13945 (EA): Cultivated.

**Ozoroa insignis subsp. reticulata (Baker f.) J.B. Gillett** Habit: Tree. Habitat: Wooded grassland, ca. 20–400 m. Vouchers: Magogo FC & Glover PE 133, RM Graham in CM 14563 (EA).

***Ozoroa obovata* (Oliv.) R. Fern. & A. Fern.** Habit: Tree. Habitat: Bushland, woodland, and forest edges, ca. 88 m. Vouchers: SAJIT–006448, 005484 (EA, HIB), Robertson SA 4240, Robertson SA 7761, Jeffery 150, Rawlins 654 (EA).

**Sclerocarya birrea subsp. caffra (Sond.) Kokwaro** Habit: Tree. Habitat: Wooded grassland, riverine woodland, and bushland, ca. 5 m. Vouchers: SAJIT–006111 (EA, HIB), Sangai GW 15779, Gray M & Luke WRQ 271 (EA).

***Sclerocarya gillettii* Kokwaro** Habit: Tree. Habitat: Bushland, 150–300 m. Voucher: Robertson 1846 (EA).

***Searsia crenulata* (A. Rich.) Moffett** Habit: Tree. Habitat: Evergreen bushland, woodland, riverine associations, and forests edges, ca. 1–300 m. Vouchers: Ngumbau V & Mwadime N V0359, Robertson SA 4217, Jeffrey 154 (EA).

***Sorindeia madagascariensis* DC.** Habit: Tree. Habitat: Riverine, coastal, and upland forests, ca. 0–297 m. Vouchers: SAJIT–006085 & 005918, Ngumbau V & Mwadime N V073 (EA, HIB), Mwadime N 11, Magogo FC & Estes R 1195, RM Graham in FD 1575, Gillet & Kibuwa 19945, Greenway & Rawlins 9356 (EA).

**F25. Ancistrocladaceae**

1 Genus, 1 Species

***Ancistrocladus robertsoniorum* J. Léonard** Habit: Liana. Habitat: Forest in disturbed areas, 60–80 m. Vouchers: Luke WRQ 1641, Drummond & Hemsley 3936, Polhill & Robertson 4786, Robertson 3649 (EA): Endemic.

**F26. Annonaceae**

17 Genera, 37 Species

**Annona senegalensis Pers. subsp. senegalensis** Habit: Shrub. Habitat: Grassland, woodland and forest, ca. 20–300 m. Vouchers: SAJIT–005602 & 005547, Ngumbau V & Mwadime N V0283 (EA, HIB), Katz SS 21, Rawlins S 645, Magogo FC & Glover PE 50 (EA).

***Artabotrys brachypetalus* Benth.** Habit: Liana. Habitat: Riverine vegetation, sometimes in dry woodland, 0–150 m. Voucher: Festo L, Luke Q & P 2724 (EA).

**Artabotrys modestus subsp. macranthus Verdc.** Habit: Liana. Habitat: Forest, woodland to grassland, 60–490 m. Vouchers: Ngumbau V & Mwadime N V0431 & V0320 (EA, HIB), Festo L, Luke Q & P 2753, Luke WRQ 3104 (EA).

***Artabotrys monteiroae* Oliv.** Habit: Liana. Habitat: Moist forest edge or moist grassland, ca. 270 m. Vouchers: Ngumbau V & Mwadime N V0504, V0536 & V040 (EA, HIB), Luke, Kibet & Amini 6303, Robertson SA, Beentje HJ & Khayota 21 & 94 (EA).

**Asteranthe asterias (S. Moore) Engl. & Diels subsp. asterias** Habit: Shrub. Habitat: Forest, 0–60 (–500) m. Vouchers: SAJIT–005449 & 006182, Ngumbau V & Mwadime N V0236 & V0417 (EA, HIB), Verdcourt B 11355, Luke WRQ & Robertson SA 524 (EA).

***Cananga odorata* (Lam.) Hook. f. & Thomson** Habit: Tree. Habitat: Plantations, 0–750 m. Vouchers: Jeffery GW 365, RM Graham in FD 2342 (EA): Cultivated.

***Huberantha stuhlmannii* (Engl.) Chaowasku** Habit: Shrub. Habitat: Shrubland, forest, and savannah, under 100 m. Vouchers: Festo L & Luke Q 2521, Rawlins 262 & 303B (EA): Near Threatened.

***Isolona cauliflora* Verdc.** Habit: Tree. Habitat: Moist forests to evergreen bushland, 20–500 m. Vouchers: SAJIT–005564 (EA, HIB), Luke WRQ & Robertson SA 1803, Luke WRQ 2930 (EA): Vulnerable.

***Lettowianthus stellatus* Diels** Habit: Tree. Habitat: Forest and riverine forest, 10–280 m. Vouchers: SAJIT–005578 (EA, HIB), Luke WRQ & Robertson SA 2768, Luke WRQ 3304 (EA).

***Mkilua fragrans* Verdc.** Habit: Shrub. Habitat: Moist forests, 0–400 m. Vouchers: SAJIT–006082, 005532, 005535 & 005919, Ngumbau V & Mwadime N V059 (EA, HIB), Faden RB & Faden AJ 77/572, Robertson SA, Luke WRQ 235 (EA): Vulnerable.

***Monanthotaxis buchananii* (Engl.) Verdc.** Habit: Liana. Habitat: Forest, bushland, and grassland, 100–400 m. Vouchers: SAJIT–004645 & 006002, Ngumbau V & Mwadime N V0301 (EA, HIB), Robertson SA & Luke WRQ 4779, 2743A, Drummond & Hemsley 4485, Verdcourt 1953 (EA).

***Monanthotaxis faulknerae* Verdc.** Habit: Liana. Habitat: Forest and savannah, 30–370 m. Vouchers: Festo L, Laizer G & P 2635, Luke WRQ 3351, Luke WRQ, PA 3822 (EA): Endangered.

***Monanthotaxis fornicata* (Baill.) Verdc.** Habit: Liana. Habitat: Forest, woodland, and bushland, 0–450 m. Vouchers: SAJIT–005447, 005448 & 005599 (EA, HIB), Festo L, Luke Q & P 2635, Battiscombe E 1044, Robertson SA 4252 (EA).

***Monanthotaxis trichocarpa* (Engl. & Diels) Verdc.** Habit: Liana. Habitat: In evergreen forest, 100–300 m. Vouchers: SAJIT–005920, Ngumbau V & Mwadime N V0302 (EA, HIB), Robertson SA 3697, Verdcourt 1895, Magogo & Glover 288 (EA).

***Monodora grandidieri* Baill.** Habit: Shrub. Habitat: Forest, bushland, and grassland, 10–290 m. Vouchers: Ngumbau V & Mwadime N V0434 (EA, HIB), Festo L, Luke Q & P 2804, Dale 2030, Kokwaro JO 3974, Robertson SA 4263 (EA).

***Monodora junodii* Engl. & Diels** Habit: Shrub. Habitat: On rocky outcrops, woodland, and dry areas, 10–15 m. Vouchers: Robertson SA 5708 & 5889, Faden RB 71/273, Luke WRQ 3560 (EA).

***Ophrypetalum odoratum* Diels** Habit: Shrub. Habitat: Woodland, forest, 0–600 m. Vouchers: Verdcourt B 1075 & 3952, Drummond & Hemsley 3981, Greenway PJ 9634 (EA): Near Threatened.

***Polyceratocarpus* sp. ?nov.** Habit: Tree. Habitat: Forest, 300–370 m. Vouchers: Luke 1621 & 881, Luke & Stone 8245 (EA): Endemic.

***Sphaerocoryne gracilis* (Engl. & Diels) Verdc. subsp. gracilis** Habit: Shrub. Habitat: Moist to dry forest, and shrubland, 30–350 m. Vouchers: SAJIT–005922, Ngumbau V & Mwadime N V0315 (EA, HIB), Luke WRQ & Robertson SA 2597, Robert SA & Luke WRQ 5190 (EA).

***Toussaintia orientalis* Verdc.** Habit: Tree. Habitat: Shrubland and forest, 140–610 m. Voucher: Luke WRQ & Robertson SA 1038 (EA): Endangered.

***Uvaria acuminata* Oliv.** Habit: Liana. Habitat: Bushland, forest, and woodland, 0–810 m. Vouchers: SAJIT–005496, Ngumbau V & Mwadime N V0161 (EA, HIB), Kuchar P 13454, Robertson SA 6456, Luke WRQ & Robertson SA 516 (EA).

***Uvaria denhardtiana* Engl. & Diels** Habit: Shrub. Habitat: Open scrub or coastal bushland, 0–300 m. Vouchers: Kirika P, Mbale M & Mbatha M 780, J Adamson 260 (EA): Near Threatened.

***Uvaria faulknerae* Verdc.** Habit: Liana. Habitat: Bushland or woodland, 45–180 m. Vouchers: Robertson SA & Luke WRQ 5688, Verdcourt 3605, Luke WRQ 118 & 3468 (EA): Endangered.

***Uvaria kirkii* Oliv. ex Hook. f.** Habit: Shrub. Habitat: Bushland, grassland, and woodland, 0–450 m. Vouchers: Ngumbau V & Mwadime N V0467 (EA, HIB), Festo L, Luke Q 2557, Rawlins 361(EA): Near Threatened.

**Uvaria leptoclados Oliv. subsp. leptoclados** Habit: Shrub. Habitat: Bushland, 30–457 m. Vouchers: Hawthorne W 510, Hildebrandt 1971, RM Graham in FD 1763 (EA).

**Uvaria lucida Benth. subsp. lucida** Habit: Shrub. Habitat: Forest, grassland, and bushland, ca. 0–400 m. Vouchers: SAJIT–005460, Ngumbau V & Mwadime N V0296 (EA, HIB), Robertson SA 4775, Muchiri J 514, Robertson SA 4232 (EA).

***Uvaria puguensis* D.M. Johnson** Habit: Liana. Habitat: Evergreen coastal forest, 200–390 m. Vouchers: Ngumbau V & Mwadime N V060 & 0364 (EA, HIB), Nyange M 525 (EA): Critically Endangered.

***Uvaria welwitschii* (Hiern) Engl. & Diels** Habit: Liana. Habitat: Evergreen coastal forests, 2–300 m. Vouchers: SAJIT–005582, Ngumbau V & Mwadime M V0472 (EA, HIB), Festo L & Luke Q 2526, Verdcourt 2124, Robertson SA 2786, Luke WRQ 536 (EA).

***Uvariodendron gorgonis* Verdc.** Habit: Tree. Habitat: Lowland rainforest, 180–800 m. Vouchers: Ngumbau V & Mwadime M V0528 (EA, HIB), Brenan JPM, Brenan JH, Gillett JB, Kanuri K & Chomba W 14601, Verdcourt B 1890 & 1911 (EA): Endangered.

***Uvariodendron kirkii* Verdc.** Habit: Shrub. Habitat: Evergreen coastal forest, often on coral, 5–450 m. Vouchers: SAJIT–005580 & 005445, Ngumbau V & Mwadime N V0516 (EA, HIB), Luke WRQ 1044 & 3408 (EA): Vulnerable.

***Uvariodendron schmidtii* Q. Luke, ined.** Habit: Tree. Habitat: Lowland rainforest, ca. 390 m. Voucher: Luke WRQ 2919 (EA): Endemic.

***Uvariodendron* sp. nov. 1 of CFS** Habit: Tree. Habitat: Forest. Voucher: Luke 1654 (EA).

***Uvariodendron* sp. nov. 2 of CFS** Habit: Tree. Habitat: Forest. Voucher: Luke 2929 (EA).

***Xylopia aethiopica* (Dunal) A. Rich.** Habit: Tree. Habitat: Forest, 40–280 m. Vouchers: Luke WRQ 3378, Luke WRQ & Robertson SA 2709 (EA).

***Xylopia arenaria* Engl.** Habit: Shrub. Habitat: Woodland and open coastal bushland, 30–600 m. Vouchers: Luke WRQ & Robertson SA 2134, 6000 & 1686, Moggridge 389, Gisau 10, Trump 96 (EA): Vulnerable.

***Xylopia holtzii* Engl.** Habit: Tree. Habitat: Forest and woodland, 50–400 m. Vouchers: SAJIT–005445, Ngumbau V & Mwadime N V0257 (EA, HIB), Robertson SA & Abio Gafo L 6812 (EA).

***Xylopia keniensis* D. M. Johnson** Habit: Tree. Habitat: Coastal forest, ca. 450 m. Vouchers: Luke 13949, Tim P et al. NMK 722, Luke & Robertson 2723 (EA): Endemic.

**F27. Apiaceae**

2 Genera, 2 Species

***Centella asiatica* (L.) Urb.** Habit: Herb. Habitat: Damp grassland along river and forest clearings, ca. 150 m. Voucher: Luke WRQ & Robertson SA 2753 (EA).

***Foeniculum vulgare* Mill.** Habit: Herb. Habitat: Naturalized in disturbed places, ca. 30 m. Voucher: Rexton K 32.F (EA): Exotic.

**F28. Apocynaceae**

53 Genera, 101 Species

***Adenium obesum* (Forssk.) Roem. & Schult.** Habit: Shrub. Habitat: Woodland and dry forest, 20–700 m. Vouchers: SAJIT–004679 & 005913 (EA, HIB), Greenway PJ & Rawlins SP 8924 (EA).

***Alafia caudata* Stapf** Habit: Liana. Habitat: Forest and bushland, 0–800 m. Vouchers: Ngumbau V & Mwadime N V0378 (EA, HIB), Magogo & Glover 791, Luke & Robertson 1680 & 985 (EA).

***Alafia microstylis* K. Schum.** Habit: Liana. Habitat: Forest, riverine forest, and bushland, 30–250 m. Vouchers: Mwadime N 22, Luke WRQ & Robertson SA 2818, Robertson & Luke 5505 (EA).

***Ancylobothrys petersiana* (Klotzsch) Pierre** Habit: Liana. Habitat: Forest, bushland, and thicket, ca. 50 m. Vouchers: SAJIT–004643, 005430 & 005590 (EA, HIB), Robertson SA 4227, Beentje 2286, Kuchar 13531 (EA).

***Ancylobothrys tayloris* (Stapf) Pichon** Habit: Liana. Habitat: Forest, riverine forest, and bushland, ca. 110 m. Vouchers: Ngumbau V & Mwadime N V0488 & 0358 (EA, HIB), Luke WRQ et al. 6196, Drummond & Hemsley 3935 (EA).

***Asclepias aurea* (Schltr.) Schltr.** Habit: Herb. Habitat: Grassland or waterlogged grassland, ca. 0–200 m. Vouchers: SAJIT–005518 (EA, HIB), Magogo FC & Glover PE 120 (EA).

***Asclepias curassavica* L.** Habit: Herb. Habitat: Forest and grassland, 0–50 m. Voucher: Jeffery K332 (EA): Naturalized.

***Aspidoglossum masaicum* (N.E.Br.) Kupicha** Habit: Herb. Habitat: Forest, grassland, ca. 206 m. Vouchers: Ngumbau V & Mwadime N V0206 (EA, HIB), Luke WRQ 3110 (EA).

***Baissea myrtifolia* (Benth.) Pichon** Habit: Liana. Habitat: Forest and bushland, 0–450 m. Vouchers: Ngumbau V & Mwadime N V0325 & 0104 (EA, HIB), Dale 3580, Luke 1602, Musyoki & Hansen 1004 (EA).

***Baseonema gregoryi* Schltr. & Rendle** Habit: Liana. Habitat: Dry bushland or evergreen forest, ca. 620 m. Voucher: Mwadime N 4 (EA).

***Calotropis gigantea* (L.) W. T. Aiton** Habit: Shrub. Habitat: Bare and degraded lands, 1–100 m. Vouchers: SAJIT–005440 (EA, HIB), Gloves 15300, Tweedie 4032 (EA).

***Calotropis procera* (Aiton.) W. T. Aiton** Habit: Shrub. Habitat: Degraded roadsides, ca. 65 m. Voucher: SAJIT–006439 (EA, HIB).

**Caralluma arachnoidea (P.R.O. Bally) M.G. Gilbert var. arachnoidea** Habit: Herb. Habitat: Grassland, bushland, and riverine scrub, 200–300 m. Voucher: Robertson SA & Luke WRQ 6029 (EA).

**Caralluma arachnoidea var. breviloba (P.R.O. Bally) M. G. Gilbert** Habit: Herb. Habitat: Grassland, bushland, and riverine scrub, ca. 400 m. Voucher: Luke WRQ & Robertson SA2109 (EA).

***Carissa bispinosa* (L.) Desf. ex Brenan** Habit: Shrub. Habitat: Forest and woodland, 0–400 m. Vouchers: Luke Q 1469, Robertson & Luke 5367, Luke & Robertson 2144 (EA).

***Carissa spinarum* L.** Habit: Shrub. Habitat: Bushland, riverine forest, and thicket. Voucher: Luke WRQ & PA sr.

***Carissa tetramera* (Sacleux) Stapf** Habit: Shrub. Habitat: Forest, thicket, and bushland, 0–450 m. Vouchers: Luke Q 1519, Drummond & Hemsley 4179 (EA).

***Carvalhoa campanulata* K. Schum.** Habit: Shrub. Habitat: Moist forest, ca. 350 m. Vouchers: Ngumbau V & Mwadime N V0311 (EA, HIB), Luke WRQ & Robertson SA 2727, Robertson 4673 (EA).

***Catharanthus roseus* (L.) G. Don** Habit: Herb. Habitat: Roadsides in dry savannah, urban open spaces, and in cultivated areas, ca. 100 m. Voucher: Spjut RW & Ensor PD 2631 (EA).

**Ceropegia abyssinica Decne. var. abyssinica** Habit: Climber. Habitat: Bushland, 80 m. Voucher: Nyange M 371 (EA).

**Ceropegia ampliata E. Mey. var. oxyloba H. Huber** Habit: Climber. Habitat: Coastal forest edges, bushland, and rocky ground, ca. 115 m. Voucher: Archer PG 401 (EA).

***Ceropegia aristolochioides* Decne.** Habit: Climber. Habitat: Bushland, thicket, and dry forest, ca. 15 m. Vouchers: Luke 4052, Kabuye CHS, Gilbert VC & Robertson SA 84/66 (EA).

***Ceropegia distincta* N.E.Br.** Habit: Climber. Habitat: Bushland, coral reef, woodland, and riverine, 0–100 m. Vouchers: Hawthorne 217, Masinde 860, Bally 12191 (EA).

***Ceropegia galeata* H. Huber** Habit: Climber. Habitat: Bushland, 50–250 m. Vouchers: Bally 12184 & S220, Bayliss 3 (EA).

***Ceropegia inornata* P.R.O. Bally ex Masinde** Habit: Climber. Habitat: Coastal forest and bushland, ca. 50 m. Vouchers: Robertson et al. 6742, Masinde 814 (EA).

***Ceropegia konasita* Masinde** Habit: Climber. Habitat: Bushland and thickets, ca. 60 m. Voucher: Mbinda & Pakia 218 (EA).

***Ceropegia nilotica* Kotschy** Habit: Climber. Habitat: Mixed deciduous woodland, ca. 300 m. Vouchers: Robertson SA & Luke WRQ 6060, Moomaw JC 1616, Masinde 816 (EA).

**Ceropegia racemosa var. voiensis Masinde** Habit: Herb. Habitat: Bushland and thicket, ca. 50–650 m. Voucher: Archer 402 (EA).

**Ceropegia seticorona var. dilatiloba P.R.O. Bally** Habit: Climber. Habitat: Bushland, 50 m. Vouchers: Kabuye CHS, Gilbert VC & Robertson SA 84/66, Drummond & Hemsley 3889 (EA).

***Ceropegia somalensis* Chiov.** Habit: Climber. Habitat: Bushland, ca. 300 m. Voucher: Adams 145 (EA).

***Chlorocyathus monteiroae* Oliv.** Habit: Climber. Habitat: Bushland, 300–800 m. Vouchers: Drummond & Hemsley 4227, Robertson & Luke 6167, Bock H 16047 (EA).

***Cryptolepis africana* (Bullock) Venter & R.L. Verh.** Habit: Climber. Habitat: Coastal lowland forest, 0–100 m. Vouchers: Drummond & Hemsley 3836, Luke & Mbinda 5978, Festo L, Luke Q & P 2784 (EA).

***Cryptolepis apiculata* K. Schum.** Habit: Climber. Habitat: Lowland forest and shrubby thickets in grassland, 30–350 m. Vouchers: Ngumbau V & Mwadime N V0373 & V0211 (EA, HIB), Saufferer 788, Kimeu JM 688, Graham 1526 (EA).

***Cryptolepis hypoglauca* K. Schum.** Habit: Climber. Habitat: Moist evergreen lowland and coastal forest, ca. 220 m. Vouchers: Ngumbau V & Mwadime N V0326 (EA, HIB), Drummond & Hemsley 3917, Rawlins 892 (EA).

***Cryptolepis nigrescens* (Wennberg) L. Joubert & Bruy** Habit: Liana. Habitat: Secondary forest and savannah, ca. 106 m. Voucher: Bally PRO 1995 (EA).

***Cynanchum crassiantherae* Liede** Habit: Climber. Habitat: Bushland and coastal dunes, near sea level. Voucher: Festo et al 2777 (EA).

**Cynanchum gerrardii (Harv.) Liede subsp. gerrardii** Habit: Climber. Habitat: Dry bushland, 2 m. Vouchers: SAJIT–006246 (EA, HIB), Luke Q 1471 (EA).

**Cynanchum hastifolium subsp. longirostrum Goyder** Habit: Climber. Habitat: Bushland, ca. 200 m. Voucher: Tweedie 3199 (EA).

***Cynanchum insipidum* (E. Mey.) Liede & Khanum** Habit: Herb. Habitat: Open grassland, bushland, woodland, and forest, ca. 20 m. Vouchers: SA Robertson 6721, Magogo FC & Glover PE 967 (EA).

***Cynanchum resiliens* (B.R. Adams & R.W.K. Holland) Goyder** Habit: Climber. Habitat: Bushland, 40–500 m. Vouchers: Drummond & Hemsley 4060, Gillett 16473 (EA).

**Cynanchum viminale (L.) L. subsp. viminale** Habit: Climber. Habitat: Dry scrub, rocky area, woodland, and riverine, ca. 590 m. Vouchers: Luke WRQ & Robertson SA 1471, Adams 146, Makin s.n in EA 13047 (EA).

**Cynanchum viminale subsp. odontolepis (Balf.f.) Goyder (Balf.f.) Goyder** Habit: Climber. Habitat: Dry coastal forest or woodland, 0–200 (–800) m. Vouchers: SAJIT–006446, Ngumbau V & Mwadime N V0494 (EA, HIB), Adams 144, Robertson & Luke 5611 (EA).

***Desmidorchis retrospiciens* Ehrenb.** Habit: Herb. Habitat: Semi-desert, ca. 0–100 m. Voucher: Gillett 19504 (EA).

***Dictyophleba lucida* (K. Schum.) Pierre** Habit: Liana. Habitat: Forest, riverine, ca. 410 m. Vouchers: Ngumbau V & Mwadime N V0308 & 058, SAJIT–005510 (EA, HIB), Luke WRQ & Robertson SA 234 (EA).

***Echidnopsis ericiflora* Lavranos** Habit: Herb. Habitat: Dry bushland, 250–450 m. Voucher: Powys & Heath 793 (EA): Endemic.

***Echidnopsis sharpei* A.C. White & B. Sloane** Habit: Herb. Habitat: Rocky slopes and woodland, ca. 450 m. Vouchers: Faden & Faden 74/1045, Luke WRQ 919 (EA).

***Edithcolea grandis* N.E.Br.** Habit: Herb. Habitat: Dry woodland, mostly on rocky ground, 200 m. Vouchers: SAJIT–005910 (EA, HIB), Karisa R 40 (EA).

***Fockea angustifolia* K. Schum.** Habit: Climber. Habitat: Bushland and coastal thicket, ca. 350 m. Vouchers: Luke WRQ et al. 6188, Drummond & Hemsley 4045 (EA).

***Funtumia africana* (Benth.) Stapf** Habit: Tree. Habitat: Forest and riverine, 30–150 m. Voucher: Luke WRQ 890 (EA).

***Gymnema sylvestre* (Retz.) R.Br. ex Sm.** Habit: Liana. Habitat: Forest margins and dry bushland, ca. 410 m. Vouchers: Ngumbau V & Mwadime N V0307 & V039 (EA, HIB), Cunningham-van Someren GR Sh 14, MD Graham 1970 (EA).

***Holarrhena pubescens* Wall. ex G. Don** Habit: Tree. Habitat: Forest, riverine, woodland, and bushland, ca. 407 m. Vouchers: SAJIT–006064 (EA, HIB), Luke Q 1409, Gachathi 50/88 (EA).

***Huernia andreaeana* (Rauh) L.C. Leach** Habit: Herb. Habitat: Dry deciduous bushland, ca. 400 m. Voucher: Rauh Ke867 (EA).

***Huernia archeri* L.C. Leach** Habit: Herb. Habitat: Rocky bushland, 200–300 m. Vouchers: Luke & Robertson 2546 & 6041, Robertson 7061 (EA): Endemic.

***Hunteria zeylanica* (Retz.) Gardner ex Thwaites** Habit: Tree. Habitat: Coral rag forest, riverine forest, and coastal bushland, 0–350 m. Vouchers: Mwadime N 33, Omino EA 89 (EA).

***Kanahia laniflora* (Forssk.) R.Br.** Habit: Shrub. Habitat: Seasonal watercourses. Voucher: Kibuwa 2465 (EA).

***Landolphia eminiana* K. Schum. ex Hallier f.** Habit: Liana. Habitat: Riverine, woodland, and thicket, ca. 300 m. Voucher: Lap LJ 307 (EA).

***Landolphia kirkii* Dyer** Habit: Liana. Habitat: Forest, Woodland, bushland, thicket, and wooded grassland, ca. 60–270 m. Vouchers: Luke Q 1508, Luke WRQ & Robertson SA 2821, Kokwaro 3960 (EA).

***Landolphia watsoniana* Romburgh** Habit: Liana. Habitat: Moist forest, 0–250 m. Vouchers: Ngumbau V & Mwadime N V006 & V0226 (EA, HIB), Luke WRQ & Robertson SA 2820, Luke 3124 (EA).

***Marsdenia crinita* Oliv.** Habit: Liana. Habitat: Forest, ca. 142 m. Vouchers: SAJIT–006007 (EA, HIB), Nyange M 574 (EA).

***Marsdenia faulknerae* (Bullock) Omlor** Habit: Liana. Habitat: Forest margins, 0–400 m. Voucher: Luke & Luke 4749 (EA).

***Marsdenia macrantha* (Klotzsch) Schltr.** Habit: Liana. Habitat: Woodland and forest margins, ca. 60 m. Vouchers: Festo L, Luke Q & P 2791, Robertson & Luke 5536 (EA).

***Marsdenia rubicunda* N.E.Br.** Habit: Liana. Habitat: Bushland and riverine, ca. 20 m. Vouchers: Magogo FC & Glover PE 877, RM Graham 2335, Kimeu JM 698, Robertson SA 3436 (EA).

***Marsdenia stelostigma* K. Schum.** Habit: Liana. Habitat: Bushland, 0–300 m. Voucher: Mungai et al. 93 (EA).

***Marsdenia taylorii* Schltr. & Rendle** Habit: Liana. Habitat: Dry forest, ca. 200 m. Vouchers: Luke 4695, Taylor s.n. (EA).

***Mascarenhasia arborescens* A. DC.** Habit: Tree. Habitat: River banks, forest, 0–750 m. Vouchers: Ngumbau V & Mwadime N V0374 (EA, HIB), Magogo FC & Estes R 1206 (EA).

***Mondia ecornuta* (N.E.Br.) Bullock** Habit: Liana. Habitat: Coastal bushland, woodland, and mangrove forest edge, 0–700 m. Vouchers: Ngumbau V & Mwadime N V0381 (EA, HIB), Luke WRQ 1637, Hawthorne W 434 (EA).

***Mondia whitei* (Hook.f.) Skeels** Habit: Liana. Habitat: Bushland, 5–250 m. Vouchers: Hawthorne W 529, Robertson SA & Luke WRQ 2352, Robertson SA, Beentje HJ, Luke WRQ & Khayota B 41 (EA).

***Oncinotis tenuiloba* Stapf** Habit: Liana. Habitat: Forest, riverine, swamp forest, and secondary forest, ca. 20 m. Vouchers: Luke & Robertson 2325, Luke 10720 (EA).

***Orbea denboefii* (Lavranos) Bruyns** Habit: Herb. Habitat: Woodland, ca. 700 m. Voucher: Heath & Powys 790 (EA).

***Orbea distincta* (E.A. Bruce) Bruyns** Habit: Herb. Habitat: Bushland, ca. 230 m. Vouchers: Luke & Robertson 2337A, Ritchie s.n. in Bally S52, Robertson SA 6902 (EA).

***Oxystelma bornouense* R.Br.** Habit: Climber. Habitat: Riverine, ca. 60 m. Vouchers: GRC van Someren 1072, Greenway 9245 (EA).

***Pachycarpus bisacculatus* (Oliv.) Goyder** Habit: Herb. Habitat: Waterlogged grassland or woodland, ca. 100 m. Vouchers: SAJIT–005521 (EA, HIB), Luke WRQ 3162, Drummond & Hemsley 1102 (EA).

***Pentatropis nivalis* (J.F. Gmel.) D.V. Field & J.R.I. Wood** Habit: Climber. Habitat: Bushland and riverine forests, ca. 20 m. Vouchers: SAJIT–005903, Ngumbau V & Mwadime N V0454 (EA, HIB), Luke Q 6153 (EA).

***Pergularia daemia* (Forssk.) Chiov.** Habit: Climber. Habitat: Dry bushland, savannah or forest margins, 0–150 m. Vouchers: SAJIT–006210 & 005936 (EA, HIB), Magogo FC & Glover PE 868 (EA).

***Pleiocarpa pycnantha* (K. Schum.) Stapf** Habit: Tree. Habitat: Evergreen forest, riverine, and swamp forest, ca. 300 m. Vouchers: Luke WRQ 893A & 1405, RB Faden & AJ Faden 77/751 (EA).

***Pleioceras orientale* Vollesen** Habit: Tree. Habitat: Coastal thicket, 300–750 m. Voucher: Luke et al. 9445 (EA): Vulnerable.

**Raphionacme sp. cf. jurensis N.E. Br.** Habit: Climber. Habitat: Woodland. Voucher: Archer 520 (EA): Endemic.

**Raphionacme splendens Schltr. subsp. splendens** Habit: Herb. Habitat: Forest, woodland, and grassland, ca. 80 m. Vouchers: Luke et al. 4494, Luke & Robertson 2331, Adamson 220, Bally 5916, Robertson 6647 (EA).

***Rauvolfia mombasiana* Stapf** Habit: Shrub. Habitat: Riverine forest and coastal forest, 0–550m. Vouchers: SAJIT–005462, Ngumbau V & Mwadime N V116 & 0225 (EA, HIB), Mussa Mandia 64 (EA).

***Saba comorensis* (Bojer ex A.DC.) Pichon** Habit: Liana. Habitat: Closed-forest, fringing forest, and savannah woodland, ca. 5–80 m. Vouchers: SAJIT–006093 (EA, HIB), Elliot CW 1348, Magogo FC & Glover PE 145 (EA).

***Schizostephanus alatus* Hochst. ex K. Schum.** Habit: Climber. Habitat: Bushland or degraded forest patches, ca. 10–300 m. Vouchers: Mwadime N 5, Luke 2454, Robertson 4939, RB Faden, AJ & Faden JB, Gillett & N Gachathi 77/427 (EA).

***Schizozygia coffaeoides* Baill.** Habit: Shrub. Habitat: Moist forest, ca. 204 m. Vouchers: SAJIT–005943 & 005579, Ngumbau V & Mwadime N V063 & 0212 (EA, HIB), Robertson SA & Luke WRQ 5184 (EA).

***Secamone gracilis* N.E. Br.** Habit: Climber. Habitat: Coastal forest, 0–500 m. Voucher: Hawthorne 191 (EA).

***Secamone parvifolia* (Oliv.) Bullock** Habit: Liana. Habitat: Thickets and coastal forest, ca. 245 m. Vouchers: Ngumbau V & Mwadime N V0269 (EA, HIB), Kokwaro JO 3562, Polhill & Paulo 613 (EA).

***Secamone punctulata* Decne.** Habit: Liana. Habitat: Thickets, riverine, and coastal forest, ca. 5–200 m. Vouchers: SAJIT–006084, Ngumbau V & Mwadime N V0410 & 0513 (EA, HIB), Graham RM FD 2004, Gerhardt K & Steiner M 50 (EA).

***Secamone retusa* N.E.Br.** Habit: Liana. Habitat: Lowland and coastal forest, 0–350 m. Vouchers: SAJIT–004659, Ngumbau V & Mwadime N V0364, 0458 & 0513 (EA, HIB), Magogo FC & Glover PE 840, Luke 3344, Greenway 1082, Harvey et al. 53 (EA).

***Secamone stuhlmannii* K. Schum.** Habit: Climber. Habitat: Thickets, ca. 0–800 m. Voucher: Reitsma J 407 (EA).

***Stathmostelma pauciflorum* K. Schum** Habit: Herb. Habitat: Waterlogged grassland, ca. 0–400 m. Vouchers: Dale IR K3544, Kassner J 159 (EA).

***Strophanthus courmontii* Sacleux ex Franch.** Habit: Liana. Habitat: Riverine forest and thickets, ca. 40–280 m. Vouchers: Ngumbau V & Mwadime N V100 & 0386 (EA, HIB), Perdue & Kibuwa 10221, Luke et al. 136, Faden 74/301 (EA).

***Strophanthus kombe* Oliv.** Habit: Liana. Habitat: Evergreen forest and dense bushland, ca. 30 m. Vouchers: Kimeu JM 664, Luke WRQ et al. 4604G, JB Gillet 21065 (EA).

***Strophanthus petersianus* Klotzsch** Habit: Liana. Habitat: Coastal forest and woodland, 0–650 m. Vouchers: Graham 1712, Robertson 4122 (EA).

***Strophanthus zimmermannianus* Monach.** Habit: Liana. Habitat: Moist forest, 0–800 m. Vouchers: Ngumbau V & Mwadime N V0123 (EA, HIB), Luke PA, WRQ 4528, Luke WRQ 877 & 5276, Magogo & Glover 4911, Luke et al. 4528 (EA).

***Tabernaemontana elegans* Stapf** Habit: Tree. Habitat: Forest, coastal bushland, and thicket, 0–400 m. Vouchers: SAJIT–005492 (EA, HIB), Kimeu JM 526, Magogo FC & Glover PE 746 (EA).

***Tabernaemontana pachysiphon* Stapf** Habit: Tree. Habitat: Moist forest and riverine forest, 0–400 m. Vouchers: SAJIT–005514 (EA, HIB), Magogo FC & Glover PE 201 (EA).

***Tacazzea apiculata* Oliv.** Habit: Liana. Habitat: Swamp and lake bank forests, ca. 30 m. Voucher: Luke et al. 778 (EA).

***Vincetoxicum apiculatum* (K. Schum.) Meve & Liede** Habit: Climber. Habitat: Coastal, riverine forest, 0–300 m. Vouchers: Robertson SA 6894, Roberson & Luke 6313, Drummond & Hemsley 3941 (EA).

***Vincetoxicum conspicuum* (N.E. Br.) Meve & Liede** Habit: Climber. Habitat: Moist forest, ca. 65 m. Vouchers: Luke & Pakia 7458, Luke & Robertson 2309, Nyange M 519 (EA).

***Vincetoxicum cernuum* (Decne.) Meve & Liede** Habit: Climber. Habitat: Bare ground, coral thickets, and sand dunes, 0–100 m. Vouchers: SAJIT–005586 (EA, HIB), Vorontsova MS 110, Napper 1273 (EA).

***Vincetoxicum stenolobum* (K. Schum.) Meve & Liede.** Habit: Climber. Habitat: Coastal, riverine forest, 0–300 m. Vouchers: Luke et al. TPR 782, Luke & Luke 5975, Kasnner 395 (EA).

***Vincetoxicum tenuipedunculatum* (K. Schum.) Meve & Liede** Habit: Climber. Habitat: Upland and coastal forest, ca. 80–240 m. Vouchers: SAJIT–005988 (EA, HIB), Archer PG 511 (EA).

***Voacanga africana* Stapf** Habit: Tree. Habitat: Forest or riverine forest, ca. 60 m. Vouchers: Luke WRQ & PA 5403, Drummond & Hemsley 3902, Luke 3829, Nyange M 504 (EA).

***Wrightia demartiniana* Chiov.** Habit: Shrub. Habitat: Dry bushland, ca. 620 m. Vouchers: Mwadime N 2429, Robertson 1771 (EA).

***Xysmalobium stocksii* N.E.Br.** Habit: Herb. Habitat: Grassland and woodland, ca. 350 m. Vouchers: Luke WRQ 3098B, Magogo & Clover 481 (EA).

**F29. Aponogetonaceae**

1 Genus, 1 Species

**Aponogeton abyssinicus var. albiflorus Lye** Habit: Herb. Habitat: Temporary pools, ca. 35 m. Voucher: Luke Q 1489 (EA).

**F30. Araceae**

11 Genera, 16 Species

**Amorphophallus maximus (Engl.) N.E.Br. subsp. maximus** Habit: Herb. Habitat: Woodland and bushland, ca. 50 m. Voucher: Hildebrandt 2018 (EA).

***Anchomanes abbreviatus* Engl.** Habit: Herb. Habitat: Evergreen forest, 0–800 m. Vouchers: SAJIT–006158, 005511 & 005979 (EA, HIB), Luke WRQ & Robertson SA 4711, Magogo FC & Glover PE 566 (EA).

***Callopsis volkensii* Engl.** Habit: Herb. Habitat: Forest, 45–800 m. Vouchers: SAJIT–005543, Ngumbau V & Mwadime N V030 (EA, HIB), Magogo FC & Glover PE 1041 (EA): Near Threatened.

***Culcasia orientalis* Mayo** Habit: Climber. Habitat: Riverine and secondary forest, 0–750 m. Vouchers: Ngumbau V & Mwadime N V0372 (EA, HIB), Greenway PJ, Rawlins SP 9360, Magogo FC & Glover PE 622 (EA).

***Gonatopus boivinii* (Decne.) Engl.** Habit: Herb. Habitat: Forest, woodland, and wooded grassland, ca. 70 m. Vouchers: Kimeu JM, Meso M Ot 601, Magogo FC & Glover PE 580, Robertson 5679 (EA).

***Gonatopus marattioides* (Peter) Bogner** Habit: Herb. Habitat: Dry lowland forest and woodland, 3–350 m. Voucher: Luke WRQ et al. 3331 (EA): Vulnerable.

***Gonatopus petiolulatus* (Peter) Bogner** Habit: Herb. Habitat: Evergreen forest, 0–400 m. Voucher: Luke WRQ & PA 4499 (EA).

***Lemna aequinoctialis* Welw.** Habit: Herb. Habitat: Rock pools, small pools, and rice fields, ca. 350 m. Vouchers: Drummond & Hemsley 4141, Polhill & Paulo 845(EA).

***Pistia stratiotes* L.** Habit: Herb. Habitat: Open still freshwater, 15–182 m. Vouchers: SAJIT–006224 & 006022 (EA, HIB), Luke PA, WRQ 5623, Magogo FC & Glover PE 908 (EA).

***Spirodela polyrrhiza* (L.) Schleid.** Habit: Herb. Habitat: Still water, ca. 450 m. Voucher: Gillett & Kibuwa 19956 (EA).

***Stylochaeton bogneri* Mayo** Habit: Herb. Habitat: Coastal evergreen forest, ca. 207 m. Vouchers: SAJIT–006022 (EA, HIB), Luke WRQ & PA 4502 (EA): Endangered.

***Stylochaeton borumensis* N.E. Br.** Habit: Herb. Habitat: Bushland, woodland, and grassland, 0–200 m. Vouchers: SAJIT–005986 & 005987 (EA, HIB).

***Stylochaeton salaamicus* N.E.Br.** Habit: Herb. Habitat: Forest or grassland, 30–250 m. Vouchers: Rawlins SP 149, Beentje HJ 2364, Luke WRQ & Robertson SA 2694 & 6186 (EA).

**Stylochaeton sp. cf. milneanus Mayo** Habit: Herb. Habitat: Forest. Voucher: R & L 6104 (EA).

***Wolffia arrhiza* (L.) Horkel ex Wimm.** Habit: Herb. Habitat: Ditches and pools, ca. 0–350 m. Voucher: Drummond & Hemsley 4164 (EA).

***Zamioculcas zamiifolia* (Lodd.) Engl.** Habit: Herb. Habitat: Forest, woodland, bushland, and grassland, 0–610 m. Vouchers: Luke Q 1547, Magogo FC & Glover PE 1129 (EA).

**F31. Araliaceae**

1 Genus, 1 Species

***Cussonia zimmermannii* Harms** Habit: Tree. Habitat: Forest and woodland, 20–400 m. Vouchers: Hawthorne W 553, Magogo FC & Glover PE 680, Drummond & Hemsley 1198, RM Graham in FD 1806, Robertson SA 3631 (EA).

**F32. Araucariaceae**

1 Genus, 1 Species

***Araucaria cunninghamii* Aiton ex D. Don** Habit: Tree. Habitat: Dry forest and thicket, ca. 15 m. Voucher: Perdue RE 10034 (EA): Cultivated.

**F33. Arecaceae**

7 Genera, 8 Species

***Areca catechu* L.** Habit: Tree. Habitat: Open secondary forest, ca. 0–100 m. Voucher: DP Kariuki EA 16085 (EA): Cultivated.

***Borassus aethiopum* Mart.** Habit: Tree. Habitat: Riverine flats, coastal plains, and open secondary forest, ca. 0–200 m. Voucher: Luke WRQ & Robertson SA sr.

***Cocos nucifera* L.** Habit: Tree. Habitat: Tropical seashores, ca. 0–100 m. Voucher: Verdcourt B 2120 (EA): Cultivated.

***Elaeis guineensis* Jacq.** Habit: Tree. Habitat: Open forest, ca. 0–200 m. Vouchers: Luke PA, WRQ 4517, Shimba Hills Survey Unit 56 (EA).

***Hyphaene compressa* H. Wendl.** Habit: Tree. Habitat: Open grassland, 150 m. Voucher: Luke WRQ & Robertson SA sr.

***Hyphaene coriacea* Gaertn.** Habit: Shrub. Habitat: Savannah and grassland, 0–300m. Vouchers: Kirika P 576, Shimba Hills Survey Unit 42 (EA).

***Phoenix reclinata* Jacq.** Habit: Tree. Habitat: Open forests, savannah woodland, or low scrub thickets, ca. 30 m. Voucher: Moomaw JC 1289 (EA).

***Raphia farinifera* (Gaertn.) Hyl.** Habit: Tree. Habitat: Moist swampy ground, ca. 0–250 m. Voucher: Magogo FC & Glover PE sr.

**F34. Aristolochiaceae**

2 Genera, 3 Species

***Aristolochia albida* Duch.** Habit: Climber. Habitat: Forest grassland, clearings, and edges, ca. 260 m. Vouchers: SAJIT–005595, Ngumbau V & Mwadime N V004 (EA, HIB), Festo L, Luke Q & P 2673 (EA).

***Aristolochia bracteolata* Lam.** Habit: Climber. Habitat: Riverine forest, 30–100 m. Vouchers: AB Katende 1796, Q Luke et al. TPR 479 (EA).

***Hydnora sinandevu* Beentje & Q. Luke** Habit: Herb. Habitat: Scattered tree grassland, ca. 2 m. Vouchers: Luke 3033, Bally 2051 & 10434 (EA).

**F35. Asparagaceae**

7 Genera, 40 Species

***Albuca abyssinica* Jacq.** Habit: Herb. Habitat: In grassland, ca. 382 m. Vouchers: Ngumbau V & Mwadime N V0357 (EA, HIB), Luke WRQ 3153 (EA).

***Asparagus africanus* Lam**. Habit: Climber. Habitat: Forest edges and bushy wooded areas, ca. 2 m. Vouchers: SAJIT–006236 (EA, HIB), Rawlins SP 84 (EA).

***Asparagus buchananii* Baker** Habit: Climber. Habitat: Grassland and wooded grassland, 0–150 m. Vouchers: Williams 299, Graham RM 1931 (EA).

***Asparagus falcatus* L.** Habit: Climber. Habitat: Bushland and dry forest, 0–850 m. Vouchers: SAJIT–006428 (EA, HIB), Greenway 9623, Luke WRQ et al. 6193, Bally 5837, Greenway 9623 (EA).

***Asparagus faulknerae* Sebsebe** Habit: Climber. Habitat: Bushland, dry forest, and woodland, 0–250 (–450) m. Vouchers: Bally & Smith B14364, Bjornstad 211 (EA).

***Asparagus flagellaris* (Kunth) Baker** Habit: Shrub. Habitat: Grassland and wooded bushland, 0–100 m. Vouchers: Mwadime N 612, Waterman 1093 (EA).

***Asparagus humilis* Engl.** Habit: Herb. Habitat: Coral outcrops, salt flats near mangrove, 0–15 m. Vouchers: Luke WRQ 3029, De Meester 19/79 (EA).

***Asparagus leptocladodius* Chiov.** Habit: Shrub. Habitat: Grassland or bushed grassland, 0–750 m. Voucher: Ngweno 7C (EA).

***Asparagus scaberulus* A. Rich.** Habit: Climber. Habitat: Woodland, grassland, and bushed grassland, (60)–350 m. Voucher: Makin in EA 13055 (EA).

***Asparagus setaceus* (Kunth) Jessop** Habit: Climber. Habitat: Woodland and grassland, ca. 50 m. Voucher: Luke WRQ & Robertson SA 989 (EA).

***Chlorophytum angustissimum* (Poelln.) Nordal** Habit: Herb. Habitat: Grassland, ca. 50 m. Voucher: Luke 3025 (EA).

**Chlorophytum cameronii var. pterocaulon (Welw. ex Baker) Nordal** Habit: Herb. Habitat: Woodland and grassland, ca. 350 m. Vouchers: Drummond & Hemsley 4156, Polhill & Paulo 834 (EA).

**Chlorophytum filipendulum subsp. amaniense (Engl.) Nordal & A.D. Poulsen** Habit: Herb. Habitat: Forest, 30–650 m. Vouchers: Ngumbau V & Mwadime N V021 & V0407 (EA, HIB), Magogo & Glover 784, Polhill & 795 (EA).

***Chlorophytum holstii* Engl.** Habit: Herb. Habitat: Forest or woodland, ca. 80 m. Vouchers: Robertson & Luke 6240, Gray M 435 (EA).

***Chlorophytum suffruticosum* Baker** Habit: Herb. Habitat: Rocks and termite mounds, ca. 10 m Vouchers: Luke Q 1553, Drummond & Hemsley 1012 (EA).

***Chlorophytum tenerrimum* Peter ex Poelln.** Habit: Herb. Habitat: Lowland forest and thickets, 70–150 m. Vouchers: SAJIT–006130 (EA, HIB), Drummond & Hemsley 3794, Polhill & Paulo 851, Musyoki & Hansen 994 (EA).

***Dipcadi longifolium* (Lindl.) Baker** Habit: Herb. Habitat: On sand along the coast, 0–30 m. Vouchers: SAJIT–006242 & 004675 (EA, HIB), Luke WRQ 3118, Bally 8877, Gillett 20836, Greenway 10458 (EA).

***Dipcadi viride* (L.) Moench** Habit: Herb. Habitat: Grassland, bushland, and woodland, ca. 500 m. Vouchers: Magogo FC & Glover PE 600, Meester-Manger Cats V de 42 (EA).

***Dracaena aletriformis* (Haw.) Bos** Habit: Shrub. Habitat: Evergreen or semi-deciduous forest, 50–300 m. Vouchers: Verdcourt 3606, Robertson & Luke 4781, Schmidt 1712 (EA).

***Dracaena fragrans* (L.) Ker Gawl.** Habit: Shrub. Habitat: Moist forest, ca. 210 m. Vouchers: Ngumbau V & Mwadime N V0391 (EA, HIB), Robertson SA 4207 (EA).

***Dracaena laxissima* Engl.** Habit: Climber. Habitat: Moist forest or riverine forest, ca. 1–400 m. Voucher: Luke WRQ & Robertson SA 242A (EA).

***Dracaena mannii* Baker** Habit: Shrub. Habitat: Moist forest, riverine forest, forest margins, and secondary bushland, ca. 300 m. Vouchers: SAJIT–005505 (EA, HIB), Robertson SA & Luke WRQ 5185 (EA).

***Drimia altissima* (L.f.) Ker Gawl.** Habit: Herb. Habitat: Grassland, bushed grassland, ca. 30 m. Vouchers: Magogo FC & Glover PE 213, Irwin 453, Jeffrey 293 (EA).

***Drimia macrocarpa* Stedje** Habit: Herb. Habitat: Grassland and bushland, ca. 35 m. Vouchers: Magogo FC & Glover PE 30, Archer 531, Drummond & Hemsley 1030 (EA).

***Ledebouria kirkii* (Baker) Stedje & Thulin** Habit: Herb. Habitat: Grassland or bushland, ca. 0–300 m. Voucher: Gilbert 6048 (EA).

***Sansevieria itumea* (Mbugua) Jankalski** Habit: Herb. Habitat: Thickets lowlands of the coastal region. Voucher: Bally & Smith B14404A (EA).

***Sansevieria arborescens* Cornu ex Gérôme & Labroy** Habit: Herb. Habitat: Evergreen coastal forest or evergreen bushland, 0–600 m. Vouchers: Reitsma J 543, Luke WRQ et al. 5703 (EA).

***Sansevieria bagamoyensis* N.E.Br.** Habit: Herb. Habitat: Open forest, coastal bushland, and thicket, 0–400 m. Vouchers: Graham 1808, Faden et al. 77/460 (EA).

***Sansevieria ballyi* L.E. Newton** Habit: Herb. Habitat: Rock faces, ca. 180 m. Vouchers: Mrs Robertson SA 7602, Newton 5856, Newton 5594 (EA): Endemic.

***Sansevieria conspicua* N.E.Br.** Habit: Herb. Habitat: Littoral forest and evergreen bushland, 2–150 m. Vouchers: Luke 3092, Drummond & Hemsley 3845, Rawlins SP 382, Robertson & Luke 2284 (EA).

***Sansevieria ehrenbergii* Schweinf. ex Baker** Habit: Herb. Habitat: Dry wooded grassland, dry bushland, and thickets, ca. 20 m. Voucher: Mrs Robertson SA 7723 (EA).

***Sansevieria fischeri* (Baker) Marais** Habit: Herb. Habitat: Dry bushland, 250–550 m. Voucher: Bally PRO 16893 (EA).

***Sansevieria forskaoliana* (Schult. f.) Hepper & J.R.I. Wood** Habit: Herb. Habitat: Dry or evergreen bushland and grassland, ca. 100 m. Voucher: Robertson 1778 (EA).

***Sansevieria francisii* Chahinian** Habit: Herb. Habitat: Coastal forest, low altitude. Voucher: Horwood 432 (EA): Endemic.

***Sansevieria gracilis* N.E.Br.** Habit: Herb. Habitat: Dry bushland and thicket, 0–100 m. Voucher: Luke WRQ & Robertson SA 1478 (EA).

***Sansevieria hyacinthoides* (L.) Druce** Habit: Herb. Habitat: Dry bushland or riverine forest, 50–600 m. Voucher: Verdcourt 3220 (EA).

***Sansevieria kirkii* Baker** Habit: Herb. Habitat: Forest, thicket, and riverine fringe, 5–180 m. Vouchers: Luke 4012 & 4013 (EA).

***Sansevieria nitida* Chahinian** Habit: Herb. Habitat: No data. Voucher: Chahinian 301 (EA): Endemic.

***Sansevieria perrotii* Warb.** Habit: Herb. Habitat: Bushland and bushed grassland, 550 m. Voucher: Sangai GW 15602 (EA).

***Sansevieria powelii* N.E.Br.** Habit: Herb. Habitat: Dry bushland and scattered tree grassland, ca. 650 m. Vouchers: Faden RB & AJ 71/810, Perdue RE & Kibuwa SP 10126, Gillett JB 19970 (EA).

***Sansevieria raffillii* N.E.Br.** Habit: Herb. Habitat: Dry bushland, woodland or dry forest, 0–500 m. Voucher: Polhill R & Paulo S 682 (EA).

**F36. Asphodelaceae**

1 Genus, 12 Species

***Aloe deserti* A. Berger** Habit: Shrub. Habitat: Deciduous thickets, ca. 259 m. Voucher: Bally PRO 8866 (EA): Near Threatened.

***Aloe kilifiensis* Christian** Habit: Herb. Habitat: Bushland, 3–380 m. Vouchers: Magogo FC & Glover PE 430, Jeffery GW 710, Frazier 881 (EA): Endangered.

***Aloe massawana* Reynolds** Habit: Herb. Habitat: Open thickets, 0–20 m. Voucher: SA Robertson 6365 (EA): Vulnerable.

***Aloe microdonta* Chiov.** Habit: Herb. Habitat: Bushland, ca. 60 m. Voucher: Gillett 16544 (EA).

***Aloe parvidens* M.G. Gilbert & Sebsebe** Habit: Herb. Habitat: *Acacia* woodland, ca. 560 m. Voucher: Tweedie 1199 (EA).

***Aloe penduliflora* Baker** Habit: Shrub. Habitat: Bushland, ca. 645 m. Voucher: Robertson & Luke 6159 (EA).

***Aloe rabaiensis* Rendle** Habit: Shrub. Habitat: Bushland, 18–500 m. Vouchers: Robertson SA & Luke WRQ 2211, Verdcourt 3193, Bally 8537, Hooper & Transcend 1156 (EA).

***Aloe ruspoliana* Baker** Habit: Herb. Habitat: Bushland, open rocky hillsides, ca. 400 m. Voucher: Bally & Smith 14990 (EA).

**Aloe secundiflora Engl. var. secundiflora** Habit: Herb. Habitat: Grassland, woodland, ca. 200 m. Voucher: Mwendia CW 16247 (EA).

***Aloe ukambensis* Reynolds** Habit: Herb. Habitat: Woodland, ca. 600 m. Voucher: Gilbert MG 1739 (EA): Vulnerable.

***Aloe vituensis* Baker** Habit: Shrub. Habitat: Bushland, ca. 290 m. Voucher: Newton & Powys 3569 (EA).

**Aloe volkensii Engl. subsp. volkensii** Habit: Shrub. Habitat: Riverine woodland, ca. 10–410 m. Vouchers: Luke WRQ & Robertson SA 1026, Robertson SA 6379 (EA).

**F37. Asteraceae**

49 Genera, 82 Species

***Acanthospermum hispidum* DC.** Habit: Herb. Habitat: Disturbed places, ca. 260 m. Voucher: Waaijenberg H 8 (EA): Naturalized.

***Acmella uliginosa* (Sw.) Cass.** Habit: Herb. Habitat: Forest, swampy area, and grassland, ca. 40 m. Vouchers: Luke WRQ & PA 6032, Nyange M 457 (EA).

***Adenostemma viscosum* J.R. Forst. & G. Forst.** Habit: Herb. Habitat: Wet ground and riverine, ca. 300 m. Vouchers: Luke WRQ & Robertson SA 2755, Magogo FC & Glover PE 102 & 373 (EA).

***Ageratum conyzoides* L.** Habit: Herb. Habitat: Woodland, grassland, and cultivated land, ca. 300 m. Voucher: Magogo FC & Glover PE 371 (EA): Naturalized.

***Aspilia kotschyi* (Sch. Bip. ex Hochst.) Oliv.** Habit: Herb. Habitat: Grassland, waste land, bushland, and woodland, ca. 350 m. Vouchers: Ngumbau V & Mwadime N V0241 (EA, HIB), Rawlins SP 817 (EA).

***Aspilia macrorrhiza* Chiov.** Habit: Herb. Habitat: Sand dunes or coral, 0–10 m. Voucher: Luke, PA & WRQ 6148 (EA): Endangered.

***Aspilia mossambicensis* (Oliv.) Wild** Habit: Shrub. Habitat: Swamps, forest, woodland, bushland, and grassland, ca. 45–200 m. Vouchers: SAJIT–006147, Ngumbau V & Mwadime N V0196 (EA, HIB), Festo L, Luke WRQ & PA 2802, Magogo FC & Glover PE 325 (EA).

***Athroisma pusillum* T. Erikss.** Habit: Herb. Habitat: Woodland, 350–500 m. Vouchers: Drummond & Hemsley 4073, Faden & Faden 72/120 (EA): Endemic.

***Bidens holstii* (O. Hoffm.) Sherff** Habit: Herb. Habitat: Forest, bushland, and grassland, ca. 700 m. Voucher: Luke & Robertson 2050 (EA).

***Bidens pilosa* L.** Habit: Herb. Habitat: Neglected places, ca. 90 m. Vouchers: Magogo FC & Glover PE 825, Craig M 9179 (EA): Naturalized.

***Bidens schimperi* Sch.Bip. ex Walp.** Habit: Herb. Habitat: Disturbed areas, woodland, bushland, and grassland, 250–372 m. Vouchers: SAJIT–006146, Ngumbau V & Mwadime N V0149 (EA, HIB), Gillett 18677, Someren, HD van 43 (EA).

***Bidens taylorii* (S. Moore) Sherff** Habit: Herb. Habitat: Grassland, bushland, and forest, ca. 60 m. Vouchers: Robertson SA & Luke WRQ 6051, Magogo & Glover 651 (EA).

***Blepharispermum ellenbeckii* Cufod.** Habit: Shrub. Habitat: Bushland, ca. 150 m. Voucher: Mungai & Rucina 404/84 (EA).

***Blepharispermum minus* S. Moore** Habit: Shrub. Habitat: Bushland and grassland, 30–450 m. Vouchers: Luke et al. TPR 650, Bally 16886 (EA).

***Blepharispermum zanguebaricum* Oliv. & Hiern** Habit: Shrub. Habitat: Dry forest and bushland, ca. 150 m. Vouchers: Robertson SA & Beentje HJ 4061, Robertson SA, Beentje HJ, Luke WRQ & Khayota B 152 (EA).

***Blumea axillaris* (Lam.) DC.** Habit: Herb. Habitat: Swampy, riverine, and grassland, ca. 1–300 m. Voucher: Magogo FC & Glover PE 526 (EA).

***Bothriocline longipes* (Oliv. & Hiern) N.E.Br.** Habit: Herb. Habitat: Wooded grassland or riverine vegetation, ca. 200 m. Voucher: Gilbert VC 3505 (EA).

***Brachylaena huillensis* O. Hoffm.** Habit: Tree. Habitat: Forest and bushland, ca. 500 m. Vouchers: Katz SS 75/33, Luke WRQ 915 (EA): Near Threatened.

***Crassocephalum crepidioides* (Benth.) S. Moore** Habit: Herb. Habitat: Riverine, forest, and cultivation area, ca. 45 m. Vouchers: Luke WRQ et al. 4728, Makokha D, Malombe I & Saidi C 1567 (EA).

***Cyanthillium cinereum* (L.) H. Rob.** Habit: Herb. Habitat: Cultivated area and grassland, ca. 243 m. Vouchers: Ngumbau V & Mwadime N V024 (EA, HIB), Luke WRQ & PA 5691, Kirika P & Muthoka P 738, Rawlins 430, Perdue & Kibuwa 10037, Robertson 3307 (EA).

***Dicoma tomentosa* Cass.** Habit: Herb. Habitat: Grassland, bushland, woodland, and rocky area, ca. 400 m. Voucher: van Someren 946 (EA).

***Eclipta prostrata* (L.) L.** Habit: Herb. Habitat: Swampy and riverine area, ca. 23 m. Vouchers: Kirika P, Muthoka P & Mbale M 755, Bally PRO & Smith AR 14373 (EA).

***Emilia bellioides* (Chiov.) C.Jeffrey** Habit: Herb. Habitat: Sand dunes and coral, 0–10 m. Vouchers: Luke WRQ 5464, Rawlins 105, Greenway & Rawlins 9421 (EA): Vulnerable.

***Emilia coccinea* (Sims) G. Don** Habit: Herb. Habitat: Grassland and bushland, ca. 186 m. Vouchers: Ngumbau V & Mwadime N V0348 (EA, HIB), Fukuoka N 284, Cunningham-van Someren GR Sh41 (EA).

***Emilia sonchifolia* (L.) DC. ex Wight** Habit: Herb. Habitat: Swampy, grassland, and cultivation area, 1–600 m. Vouchers: Jeffery 780, Greenway & Rawlins 9375 (EA).

***Emilia tricholepis* C. Jeffrey** Habit: Herb. Habitat: Woodland, ca. 5–200 m. Voucher: Brathany C 58 (EA).

***Erigeron aegyptiacus* L.** Habit: Herb. Habitat: Grassland, cultivation, riverbeds, floodplains, ca. 100 m. Voucher: Njoroge Thairu 38 (EA).

***Erigeron bonariensis* L.** Habit: Herb. Habitat: Grassland, 0–500 m. Voucher: Magogo FC & Glover PE 344 (EA).

***Erythrocephalum marginatum* (O. Hoffm.) S. Ortiz & A.P. Cout.** Habit: Herb. Habitat: Forest, ca. 53 m. Vouchers: SAJIT–004651, Ngumbau V & Mwadime N V0130 (EA, HIB), Magogo FC & Glover PE 937, Simpson BL 388 (EA).

***Erythrocephalum minus* Oliv.** Habit: Herb. Habitat: Grassland, forest, woodland, and roadsides, ca. 45 m. Vouchers: Graham RM 1928, Drummond & Hemsley 4146, Luke & Luke 3980 (EA).

***Ethulia angustifolia* Bojer ex DC.** Habit: Herb. Habitat: Cultivation, riverine, roadside, and grassland, ca. 210 m. Vouchers: Ngumbau V & Mwadime N V0376 (EA, HIB), Festo L, Luke Q & P 2803 (EA).

***Ethulia faulknerae* C. Jeffrey** Habit: Herb. Habitat: Bushland, coral, 0–15 m. Vouchers: Luke WRQ 15341, Gregory, Festo 2803 (EA).

***Ethulia gracilis* Delile** Habit: Herb. Habitat: A common weed, ca. 256 m. Vouchers: Fukuoka N 517, Kirika P 934, Kuchar P 7267 (EA).

***Flaveria trinervia* (Spreng.) C. Mohr** Habit: Herb. Habitat: Waste ground, swampy, and cultivation. Voucher: Drummond & Hemsley 1047 (EA): Naturalized.

***Galinsoga parviflora* Cav.** Habit: Herb. Habitat: Disturbed areas and roadside, ca. 0–100 m. Voucher: Jeffery GW 776 (EA): Naturalized.

***Geigeria acaulis* Benth. & Hook.f. ex Oliv. & Hiern** Habit: Herb. Habitat: Grassland and bushland, ca. 250 m. Voucher: Gillett & Gachathi 20527 (EA).

***Grangea maderaspatana* (L.) Poir.** Habit: Herb. Habitat: Riverine, ca. 5–100 m. Voucher: Luke et al. TPR 144 (EA).

***Grauanthus linearifolius* (O. Hoffm.) Fayed** Habit: Herb. Habitat: Grassland and forest, 1–150 m. Vouchers: Greenway & Rawlins 9455, Hooper & Townsend 1181 (EA).

**Gutenbergia cordifolia Benth. ex Oliv. var. cordifolia** Habit: Herb. Habitat: Grassland, riverine, forest, roadside, and cultivated area, ca. 300 m. Voucher: Boyle B 132 (EA).

***Gutenbergia pembensis* S. Moore** Habit: Herb. Habitat: Woodland, grassland, and forest, 1–400 m. Vouchers: Ngumbau V & Mwadime N V0461 (EA, HIB), Simpson, BL 76, Magogo FC & Glover PE 1084, Boyle B 132, Luke & Robertson 1780, Kassner 366 (EA).

***Gymnanthemum coloratum* (Willd.) H. Rob. & B. Kahn** Habit: Shrub. Habitat: Woodland or riverine, 160–250 m. Vouchers: Magogo FC & Glover PE 1142, Faden 77/705 (EA).

***Gynura colorata* Peter ex F. G. Davies** Habit: Herb. Habitat: Forest and rocky area, 0–850 m. Voucher: Musyoki BM & Hansen OJ 974 (EA).

***Gynura pseudochina* (L.) DC.** Habit: Herb. Habitat: Grassland and woodland, ca. 0–100m. Vouchers: Festo L & Luke WRQ 2691, Kassner 475 (EA).

***Gynura scandens* O. Hoffm.** Habit: Climber. Habitat: Forest, riverine and woodland, ca. 1–200 m. Vouchers: Magogo FC & Glover PE 975, Luke & Robertson 1789 (EA).

***Helichrysum glumaceum* DC.** Habit: Herb. Habitat: Grassland or dry bushland, ca. 350 m. Vouchers: Luke WRQ & Robertson SA 2541, Drummond RB & Hemsley JH 4047 (EA).

***Jeffreycia hildebrandtii* (Vatke) H. Rob., S. C. Keeley & Skvarla** Habit: Shrub. Habitat: Forest margins, swampy area, riverine, bushland, and roadsides, 1–700 m. Vouchers: Kirika P, Muthoka P & Mbale M 749, Magogo FC & Glover PE 171 (EA).

***Jeffreycia zanzibarensis* (Less.) H. Rob., S. C. Keeley & Skvarla** Habit: Herb. Habitat: Forest margins, bushland, and roadsides, ca. 96 m. Vouchers: SAJIT–006126 (EA, HIB), Adamson 11, Drummond & Hemsley 1155 (EA).

**Kleinia abyssinica (A. Rich.) A. Berger var. abyssinica** Habit: Herb. Habitat: Bushland, ca. 60 m. Vouchers: SAJIT–004666 (EA, HIB), Simpson BL 390 (EA).

**Kleinia abyssinica var. hildebrandtii (Vatke) C. Jeffrey** Habit: Herb. Habitat: Rocky, grassland, bushland, woodland, and forest, 20–250 m. Vouchers: Luke WRQ & Robertson SA 964, Musyoki & Hansen 971 (EA).

***Kleinia implexa* (P.R.O. Bally) C. Jeffrey** Habit: Herb. Habitat: Bushland and rocky area, ca. 340 m. Vouchers: Luke WRQ & Robertson SA 2566, Drummond & Hemsley 4092 (EA).

***Kleinia odora* (Forssk.) DC.** Habit: Shrub. Habitat: Bushland, ca. 75 m. Voucher: Bally PRO 16891 (EA).

***Kleinia schweinfurthii* (Oliv. & Hiern) A. Berger** Habit: Herb. Habitat: Bushland, ca. 200 m. Voucher: Luke & Luke 4427 (EA).

***Laggera brevipes* Oliv. & Hiern** Habit: Herb. Habitat: Grassland, woodland, and waste ground, 1–200 m. Voucher: Magogo FC & Glover PE 1137 (EA).

***Launaea cornuta* (Hochst. ex Oliv. & Hiern) C. Jeffrey** Habit: Herb. Habitat: Grassland and cultivated area, ca. 227 m. Vouchers: Ngumbau V & Mwadime N V0275 (EA, HIB), Magogo FC & Glover PE 813 (EA).

***Launaea intybacea* (Jacq.) Beauverd** Habit: Herb. Habitat: Riverbed, coral, and cultivated area, ca. 6 m. Vouchers: Greenway 9267, Mwadime N & Chesire C 195 (EA).

***Launaea nana* (Baker) Chiov.** Habit: Herb. Habitat: Grassland, ca. 450 m. Voucher: Luke WRQ et al. 4604E (EA).

***Launaea sarmentosa* (Willd.) Sch.Bip. ex Kuntze** Habit: Herb. Habitat: High water mark, beach, and sandy shores, 0–15 m. Vouchers: SAJIT–006260 (EA, HIB), Rawlins 184, Robertson 6136, Drummond & Hemsley 4001 (EA).

***Microglossa hildebrandtii* O. Hoffm.** Habit: Liana. Habitat: Forest and bushland, 1–600 m. Vouchers: Robertson SA & Luke WRQ 5591, Magogo FC & Glover PE 737, Luke WRQ 1811 (EA).

***Mikania chenopodiifolia* Willd.** Habit: Climber. Habitat: Forest, swampy, and riverine, 0–300 m. Voucher: Magogo FC & Glover PE 1126 (EA).

***Nicolasia nitens* (O. Hoffm.) Eyles** Habit: Herb. Habitat: Bushland, riverine, and temporary pools, 300–600 m. Voucher: Mungai et al. 284/83 (EA).

***Orbivestus homilanthus* (S. Moore) H. Rob.** Habit: Shrub. Habitat: Forest margin, bushland, and beach, 0–15 m. Voucher: Gillespie JB 204 (EA).

***Pluchea dioscoridis* (L.) DC.** Habit: Shrub. Habitat: Riverine, woodland, and swampy, ca. 61 m. Vouchers: Sangai GW EA 15795, Kibuwa 2466, Robertson & Luke 5816, Polhill & Paulo 830 (EA).

***Pluchea sordida* (Vatke) Oliv. & Hiern** Habit: Shrub. Habitat: Riverine, ca. 256 m. Vouchers: Luke Q 5651, Kuchar P 11974, Kirika P 932 (EA).

***Porphyrostemma monocephala* (E.A. Bruce) Leins** Habit: Herb. Habitat: Grassland, ca. 45 m. Voucher: Makin 422 (EA).

***Pseudoconyza viscosa* (Mill.) D’Arcy** Habit: Herb. Habitat: Riverine forest, ca. 1–300 m. Vouchers: Luke 3354, Jeffery GW 703, Magogo FC & Glover PE 388 (EA).

***Sigesbeckia orientalis* L.** Habit: Herb. Habitat: Cultivated and ruderal area, ca. 240 m. Vouchers: Greenway PJ 9178, Pierce T, David & James (EA).

***Solanecio angulatus* (Vahl) C. Jeffrey** Habit: Climber. Habitat: In rivers and streams or woodland, 250–600 m. Voucher: Luke WRQ & PA sr.

***Solanecio cydoniifolius* (O. Hoffm.) C.Jeffrey** Habit: Climber. Habitat: Forest margin, cultivated area, and coral, ca. 3 m. Vouchers: van Someren VGL Sh 3, Wakefield s.n., Mwadime & Chesire 278 (EA).

***Sphaeranthus africanus* L.** Habit: Herb. Habitat: Riverine, 1–450 m. Vouchers: Luke WRQ 2938, Nyange M 489 (EA).

***Sphaeranthus bullatus* Mattf.** Habit: Herb. Habitat: Riverine and rocky area, ca. 60 m. Voucher: Luke & Luke 3827a (EA).

**Sphaeranthus kirkii Oliv. & Hiern var. kirkii** Habit: Herb. Habitat: Riverine, ca. 137 m. Vouchers: Fukuoka N 270, Magogo & Glover 760 (EA).

**Sphaeranthus kirkii var. cyathuloides (O. Hoffm.) Beentje** Habit: Herb. Habitat: Bushland, 1–1750 m. Voucher: Thomas 125 (EA).

***Sphaeranthus spathulatus* A. Peter** Habit: Herb. Habitat: Ponds and streams, 1–250 m. Voucher: Festo L & Luke Q 2577 (EA).

***Sphaeranthus ukambensis* Vatke & O. Hoffm.** Habit: Herb. Habitat: Riverine, bushland, grassland, and woodland, 1–450 m. Voucher: Tana R. Primate Res. Expedition 298 (EA).

***Sphaeranthus zavattarii* Cufod.** Habit: Herb. Habitat: Grassland and swampy area, 100–450 m. Voucher: Makin EA 14692 (EA).

***Synedrella nodiflora* (L.) Gaertn.** Habit: Herb. Habitat: Waste land or cultivated land, ca. 213 m. Vouchers: SAJIT–006071, Ngumbau V & Mwadime N V0335 (EA, HIB), Magogo FC & Glover PE 722 (EA): Naturalized.

***Tridax procumbens* L.** Habit: Herb. Habitat: Weed of cultivated areas, 213–381 m. Vouchers: Waaijenberg H 10, Magogo FC & Glover PE 723 & 351 (EA): Naturalized.

**Vernonia colorata (Willd.) Drake subsp. colorata** Habit: Tree. Habitat: Woodland or riverine, ca. 60 m. Vouchers: Faden & Faden 77/705, Nyange M 506 (EA).

***Vernonia popeana* C. Jeffrey** Habit: Herb. Habitat: Bushland, wooded grassland, and woodland, 0–150 m. Voucher: Luke & Robertson 2514 (EA).

***Vernonia wakefieldii* Oliv.** Habit: Shrub. Habitat: Bushland, ca. 350 m. Vouchers: SAJIT–005429 (EA, HIB), Robertson SA & Beentje HJ 4062, Drummond & Hemsley 4058 (EA).

***Vernoniastrum aemulans* (Vatke) H. Rob.** Habit: Herb. Habitat: Roadside, cultivations, grassland, woodland, and bushland, ca. 80–375 m. Vouchers: Ngumbau V & Mwadime N V0147 (EA, HIB), Waaijenbrg H 3, Magogo FC & Glover PE 716, 1087, 398 & 500 (EA).

***Wollastonia biflora* (L.) DC.** Habit: Shrub. Habitat: Bushland, coral, and sandy shores, 0–15 m. Voucher: Festo L & Luke WRQ 2454 (EA).

**F38. Balsaminaceae**

1 Genus, 1 Species

***Impatiens walleriana* Hook. f.** Habit: Herb. Habitat: Upland and coastal rainforest, ca. 200 m. Vouchers: SAJIT–005953, Ngumbau V & Mwadime N V0397 (EA, HIB), Magogo FC & Glover PE 129, Luke 1336 (EA).

**F39. Basellaceae**

1 Genus, 2 Species

***Basella alba* L.** Habit: Climber. Habitat: Moist places, ca. 136 m. Voucher: SAJIT–005968 (EA, HIB).

***Basella paniculata* Volkens** Habit: Climber. Habitat: Bushland and forest, ca. 20 m. Vouchers: Robertson SA 5720, Adams BR 59 (EA).

**F40. Begoniaceae**

1 Genus, 1 Species

***Begonia wakefieldii* Gilg ex Engl.** Habit: Herb. Habitat: Forest and bushland, ca. 136–280 m. Vouchers: SAJIT–005952 (EA, HIB), Luke WRQ & Robertson SA 478, Luke WRQ 1634 (EA).

**F41. Bignoniaceae**

5 Genera, 5 Species

***Fernandoa magnifica* Seem**. Habit: Tree. Habitat: Coastal thicket and woodland, 5–450 m. Vouchers: SAJIT–006157, Ngumbau V & Mwadime N V0385 (EA, HIB), Magogo FC & Glover PE 168 (EA).

***Kigelia africana* (Lam.) Benth.** Habit: Tree. Habitat: Grassland and woodland, ca. 300 m. Voucher: Jeffery 111 (EA).

***Markhamia zanzibarica* (Bojer ex DC.) K. Schum.** Habit: Shrub. Habitat: Coastal thicket and woodland, ca. 230 m. Vouchers: Ngumbau V & Mwadime N V0466 (EA, HIB), Shimba Hills Survey Unit 90, Kimeu JM 536 (EA).

**Spathodea campanulata P. Beauv. subsp. nilotica (Seem.) Bidgood** Habit: Shrub. Habitat: Scrubland and secondary forests, ca. 100 m. Voucher: Luke WRQ 3089 (EA).

***Stereospermum kunthianum* Cham.** Habit: Tree. Habitat: Dry areas of deciduous forest and woodland, ca. 20 m. Voucher: Magogo FC & Glover PE 642 (EA).

**F42. Bixaceae**

2 Genera, 2 Species

***Bixa orellana* L.** Habit: Shrub. Habitat: Cultivated, naturalized, ca. 30–150 m. Vouchers: SAJIT–005493 (EA, HIB), Spjut RW 2637 (EA): Naturalized.

***Cochlospermum religiosum* (L.) Alston** Habit: Tree. Habitat: Dry forests. Voucher: Graham RM 2334 (EA).

**F43. Boraginaceae**

7 Genera, 28 Species

***Argusia argentea* (L.f.) Heine** Habit: Shrub. Habitat: Sea shore, ± sea level. Voucher: Greenway 10442 (EA).

**Cordia crenata subsp. meridionalis Warfa** Habit: Shrub. Habitat: Bushland, ca. 60 m. Voucher: Thairu 110 (EA).

***Cordia faulknerae* Verdc.** Habit: Liana. Habitat: Riverine bushland, 0–250 m. Vouchers: SA Robertson & WRQ Luke 5732, Luke 1573 (EA).

***Cordia goetzei* Gürke** Habit: Tree. Habitat: Coastal forest, rainforest, riverine forest, and ground water forest, 5–60 m. Vouchers: Mwadime N & Ndungu K 27, RB Faden & AJ Faden 77/657 (EA).

**Cordia guineensis Thonn. subsp. mutica Verdc.** Habit: Shrub. Habitat: Limestone outcrops, 0–10 (–50) m. Vouchers: SAJIT–006185 (EA, HIB), Mwadime N 46, Luke Q 1413 (EA).

***Cordia monoica* Roxb.** Habit: Tree. Habitat: Dry woodland, ca. 15 m. Voucher: Mariette Steiner 290 (EA).

***Cordia sinensis* Lam.** Habit: Tree. Habitat: Grassland and bushland, ca. 60 m. Voucher: Drummond & Hemsley 4185 (EA).

***Cordia somaliensis* Baker** Habit: Shrub. Habitat: Coastal bushland, high-tidemark, and coral rag, 0–15 (–150) m. Vouchers: Kirika P, Muthoka P & Mbale M 758, Greenway & Rawlins 8904 (EA).

***Cordia* sp. B of FTEA** Habit: Shrub. Habitat: *Acacia*-*Combretum* woodland. Vouchers: Muchiri 567, Kuchar 13691 (EA).

***Cordia subcordata* Lam.** Habit: Tree. Habitat: Shore, high-tidemark, and mangrove swamps, 0–15 (–75) m. Vouchers: Kimeu JM 667, Gillett 20837, Verdcourt 1070 (EA).

***Cordia torrei* E.S. Martins** Habit: Tree. Habitat: Forest and rock outcrop, 200–420 m. Vouchers: Verdcourt 2414, Luke & Robertson 1876 (EA).

***Ehretia amoena* Klotzsch** Habit: Shrub. Habitat: Woodland, 0–600 m. Vouchers: Ngumbau V & Mwadime N V0507 (EA, HIB), Luke WRQ et al. 6192 (EA).

***Ehretia bakeri* Baker** Habit: Shrub. Habitat: Forest, bushland, 199–700 m. Vouchers: SAJIT–006456, Ngumbau V & Mwadime N V0424 (EA, HIB), Shimba Hills Survey Unit 84, R & L 2047, Drummond & Hemsley 4201 (EA).

**Ehretia cymosa var. silvatica (Gürke) Brenan** Habit: Tree. Habitat: Forest, grassland, and bushland, ca. 10 m. Voucher: Luke WRQ 3073 (EA).

***Euploca ovalifolia* (Forsk.) Diane & Hilger** Habit: Herb. Habitat: Bushland, woodland, and grassland, ca. 15 m. Voucher: Luke Q 5657 (EA).

***Euploca strigosa* (Willd.) Diane & Hilger** Habit: Herb. Habitat: Woodland, grassland, and along road sides, ca. 186 m. Vouchers: SAJIT–006134, Ngumbau V & Mwadime N V0343 (EA, HIB), Luke Q 1550, Graham RM 1901 (EA).

***Heliotropium benadirense* Chiov.** Habit: Herb. Habitat: Sand dunes and sandy places, 0–120 m. Voucher: Luke Q 5488 (EA).

***Heliotropium gorinii* Chiov.** Habit: Herb. Habitat: Above high tide and roadsides, 0–15 m. Vouchers: Verdcourt 3909, Rayner 296a, Greenway 13138 (EA).

**Heliotropium pectinatum Vaupel subsp. pectinatum** Habit: Herb. Habitat: Grassland, and *Commiphora*-*Acacia* bushland, 15–620 m. Voucher: Polhill & Paulo 593 (EA).

***Heliotropium simile* Vatke** Habit: Herb. Habitat: Bushland and scrub, ca. 150 m. Voucher: Mungai et al. 112 (EA).

***Heliotropium* sp. A of FTEA** Habit: Herb. Habitat: Grassland with scattered *Acacia*, ca. 96 m. Voucher: Gillett 16522 (EA).

**Heliotropium steudneri Vatke subsp. steudneri** Habit: Herb. Habitat: Bushland and grassland, 0–200 m. Vouchers: Ngumbau V & Mwadime N V0455 (EA, HIB), Luke Q 5658 (EA).

***Heliotropium zeylanicum* (Burm.f.) Lam.** Habit: Herb. Habitat: Bushland or grassland, ca. 20 m. Voucher: Luke Q 5659 (EA).

***Hilsenbergia nemoralis* (Gürke) J.S. Mill.** Habit: Tree. Habitat: Forest, woodland, and coral, 0–600 m. Vouchers: Drummond & Hemsley 1106, Magogo & Glover 727, J Adamson 289 (EA).

***Hilsenbergia petiolaris* (Lam.) J. S. Mill.** Habit: Shrub. Habitat: Forest, woodland, and coral, 0–30 (–660) m. Vouchers: SAJIT–005604, Ngumbau V & Mwadime N V0298 (EA, HIB), Bridson 129, Jeffery K75, Verdcourt 1081, Festo L & Luke Q 2561 (EA).

***Hilsenbergia teitensis* (Gürke) J. S. Mill.** Habit: Shrub. Habitat: Bushland and forest, 60–850 m. Vouchers: SAJIT–005422 (EA, HIB), Drummond & Hemsley 4267 (EA).

***Trichodesma indicum* (L.) Lehm.** Habit: Herb. Habitat: Grassland and coconut plantations, 0–45 m. Voucher: Linder 2650 (EA).

***Trichodesma zeylanicum* (Burm. f.) R. Br.** Habit: Herb. Habitat: Forest, 15 m. Voucher: GW Sangai 15597 (EA).

**F44. Burmanniaceae**

1 Genus, 1 Species

***Afrothismia baerae* Cheek** Habit: Herb. Habitat: Evergreen coastal, 360–400 m. Voucher: Baer S s.n. (EA): Critically Endangered, Endemic.

**F45. Burseraceae**

2 Genera, 13 Species

***Boswellia neglecta* S. Moore** Habit: Tree. Habitat: Bushland, ca. 400–620 m. Vouchers: Mwadime N2, Luke WRQ 196, Robertson SA & Khayota B 6416 (EA).

**Commiphora africana (A. Rich.) Engl. var. africana** Habit: Tree. Habitat: Grassland and bushland, ca. 110 m. Vouchers: Kuchar P 12902, Kibuwa SP 1224 (EA).

**Commiphora africana var. glaucidula (Engl.) J. B. Gillett** Habit: Tree. Habitat: Bushed grassland, ca. 450 m. Vouchers: Rawlins SP 130, Luke WRQ 915 (EA).

**Commiphora africana var. oblongifoliolata (Engl.) J. B. Gillett** Habit: Tree. Habitat: Bushed grassland, ca. 20 m. Vouchers: Gillett JB 20354, Cunningham van Someren GR Sh 82, Kibuwa SP 19845 (EA).

**Commiphora campestris subsp. glabrata (Engl.) J. B. Gillett** Habit: Tree. Habitat: Bushland, 10–200 m. Voucher: Faden 77/476 (EA).

**Commiphora edulis subsp. boiviniana (Engl.) J. B. Gillett** Habit: Tree. Habitat: Bushland and thickets dominated by Sapotaceae, ca. 70 m. Vouchers: van Someren VGL Sh 61, Robertson SA 4083 (EA).

**Commiphora eminii subsp. zimmermannii (Engl.) J. B. Gillett** Habit: Tree. Habitat: Forest, 5–110 m. Vouchers: Luke WRQ & Robertson SA 212, RB & AJ Faden77/650 (EA).

***Commiphora erosa* Vollesen** Habit: Tree. Habitat: Woodland, 90–400 m. Voucher: Robertson SA 1849 (EA).

***Commiphora kua* (R. Br. ex Royle) Vollesen** Habit: Tree. Habitat: Coastal thicket, 2–360 m. Vouchers: SAJIT–006237 (EA, HIB), Robertson SA 4082, Polhill R & Paulo S 495 (EA).

***Commiphora obovata* Chiov.** Habit: Shrub. Habitat: Coastal bushland, 50–100 m. Vouchers: Gillett JB 20353, Mahasi AS in EAH 14890 (EA): Near Threatened.

***Commiphora pseudopaolii* J. B. Gillett** Habit: Tree. Habitat: Bushland, 60–600 m. Vouchers: Gillett 16374, Verdcourt & Polhill 2709 (EA): Near Threatened.

***Commiphora pteleifolia* Engl.** Habit: Shrub. Habitat: Coastal thicket, ca. 10 m. Vouchers: Luke Q 1477, RB Faden, AJ Faden, JB Gillett & N Gachathi 77/475 (EA).

***Commiphora sennii* Chiov.** Habit: Shrub. Habitat: Bushland, ca. 200 m. Voucher: Gillet & Gachathi 20535 (EA).

***Commiphora unilobata* J. B. Gillett & Vollesen** Habit: Shrub. Habitat: Bushland, ca. 70 m. Vouchers: Gillett 21108 & 21109 (EA).

***Commiphora zanzibarica* (Baill.) Engl.** Habit: Tree. Habitat: Coastal thickets and coral, 2–510 m. Vouchers: Willan 341, RB & SA Faden 77/650, SA Robertson 3663 (EA).

**F46. Buxaceae**

1 Genus, 1 Species

***Buxus obtusifolia* (Mildbr.) Hutch.** Habit: Shrub. Habitat: Forest, 280–450 m. Vouchers: SAJIT–005537, Ngumbau V & Mwadime N V0496 (EA, HIB), Mutanga JG & Kamau 6, Hawthorne W 139 (EA): Vulnerable.

**F47. Cactaceae**

2 Genera, 2 Species

***Opuntia ficus-indica* (L.) Mill.** Habit: Shrub. Habitat: Dry arid areas, 0–300 m. Voucher: Luke WRQ & PA sr: Cultivated.

***Rhipsalis baccifera* (Sol.) Stearn** Habit: Epiphytic herb. Habitat: Wet forests and riverine forest, ca. 145 m. Vouchers: Faden RB & Beentje HJ 85/47, Magogo FC & Glover PE 633 (EA).

**F48. Calophyllaceae**

1 Genus, 1 Species

***Calophyllum inophyllum* L.** Habit: Tree. Habitat: Rocky and sandy seashores, 0–20 m. Voucher: Robertson SA 4926 (EA).

**F49. Campanulaceae**

2 Genera, 4 Species

**Lobelia fervens Thunb. subsp. fervens** Habit: Herb. Habitat: Grassland, forest margins, roadsides, streamside or on coastal sand, ca. 136 m. Vouchers: SAJIT–005960 & 005945 (EA, HIB), Magogo FC & Glover PE 734, Polhill & Paulo 488 (EA).

**Lobelia fervens subsp. recurvata (E. Wimm.) Thulin** Habit: Herb. Habitat: On moist ground in grassland or woodland, ca. 15 m. Vouchers: Mwadime N & Luke WRQ 784 & 419 (EA).

**Wahlenbergia abyssinica (Hochst. ex A. Rich.) Thulin subsp. abyssinica** Habit: Herb. Habitat: Cultivated or waste ground, ca. 300 m. Vouchers: Magogo FC & Glover PE 121, Thulin M 300 (EA).

***Wahlenbergia napiformis* (A. DC.) Thulin** Habit: Herb. Habitat: Woodland, grassland, and roadsides, ca. 300 m. Vouchers: Luke WRQ & Robertson SA 2823, Thulin M 298 (EA).

**F50. Canellaceae**

1 Genus, 1 Species

***Warburgia stuhlmannii* Engl.** Habit: Tree. Habitat: Forest and woodland, 20–225 m. Vouchers: Luke WRQ 15340, Graham 2208 (EA): Vulnerable.

**F51. Cannabaceae**

2 Genera, 3 Species

***Celtis mildbraedii* Engl.** Habit: Tree. Habitat: Lowland rainforest, ca. 160–300 m. Vouchers: Mwadime N 2, Luke WRQ & Robertson SA 2810, RB Faden & AJ Faden 77/678, RB Faden, AJ Faden & N Gachathi 77/526 (EA).

***Celtis philippensis* Blanco** Habit: Tree. Habitat: Lowland, groundwater, and riverine forest, ca. 30–207 m. Vouchers: SAJIT–006021 (EA, HIB), Mwadime N 30, Luke WRQ & Robertson SA 504, Thomas Mwadime in Mrs Robertson SA 7807, Drummond & Hemsley 4014, Verdcourt 2405 (EA).

***Trema orientalis* (L.) Blume** Habit: Tree. Habitat: Margins of lowland and upland rainforest, ca. 280 m. Vouchers: SAJIT–005457 (EA, HIB), Jeffery 193 (EA).

**F52. Capparaceae**

7 Genera, 37 Species

**Boscia angustifolia A. Rich. var. angustifolia** Habit: Tree. Habitat: Woodland and bushland, ca. 200 m. Voucher: Robertson SA & Luke Q 4721 (EA).

***Boscia coriacea* Pax** Habit: Tree. Habitat: Bushland and grassland, ca. 150 m. Vouchers: Kuchar P 13709, Drummond RB 4093, Graham RM 1586 (EA).

***Boscia keniensis* Beentje** Habit: Shrub. Habitat: Bushland, ca. 100–350 m. Vouchers: Bally 2024, Robertson SA 319 (EA).

***Boscia mossambicensis* Klotzsch** Habit: Tree. Habitat: Woodland, bushland, and thicket, ca. 630 m. Voucher: Luke PA WRQ 4400 (EA).

***Boscia salicifolia* Oliv.** Habit: Tree. Habitat: Woodland, bushland, bamboo thicket, and grassland, ca. 300 m. Vouchers: Burstyne P 70, Bally J 13944 (EA).

***Cadaba carneoviridis* Gilg & Gilg-Ben.** Habit: Shrub. Habitat: Bushland and grassland, 30–600 m. Vouchers: Gilbert MG 5862, Robertson SA 2086 (EA).

**Cadaba farinosa Forssk. subsp. farinosa** Habit: Shrub. Habitat: Bushland and grassland, 0–300 m. Vouchers: Greenway PJ 9440, Moomaw JC 1578 (EA).

**Cadaba farinosa subsp. adenotricha (Gilg & Gilg-Ben.) R. A. Graham** Habit: Shrub. Habitat: Bushland and grassland, 0–400 m. Vouchers: Luke WRQ et al. 5411, Rayner RW 163 (EA).

***Cadaba gillettii* R.A. Graham** Habit: Shrub. Habitat: Bushland, 100–750 m. Voucher: Mrs J Adamson 103 (EA).

***Cadaba glandulosa* Forssk.** Habit: Shrub. Habitat: Bushland and grassland, ca. 600 m. Voucher: Greenway 8866 (EA).

***Cadaba rotundifolia* Forssk.** Habit: Shrub. Habitat: Bushland, 0–400 m. Voucher: SAJIT–005425 (EA, HIB).

***Cadaba ruspolii* Gilg** Habit: Shrub. Habitat: Bushland and woodland, ca. 180 m. Voucher: Bally 2010 (EA).

**Capparis erythrocarpos var. rosea (Klotzsch) De Wolf** Habit: Shrub. Habitat: Woodland and bushland, 0–400 m. Vouchers: SAJIT–006432, Ngumbau V & Mwadime N V0497 (EA, HIB), Greenway PJ & Rawlins SP 9353 (EA).

**Capparis fascicularis DC. var. fascicularis** Habit: Shrub. Habitat: Bushland and grassland, 600 m. Voucher: Battiscombe 800 (EA).

**Capparis fascicularis var. scheffleri (Gilg & Benedict) DeWolf** Habit: Shrub. Habitat: Forest and bushland, ca. 0–400 m. Vouchers: Ngumbau V & Mwadime N V0445 (EA, HIB), Sangai GW 15690, Luke WRQ 3475 (EA).

**Capparis sepiaria var. fischeri (Pax) DeWolf** Habit: Shrub. Habitat: Bushland and grassland, 180 m. Voucher: Battiscombe 269 (EA).

**Capparis sepiaria var. stuhlmannii (Gilg) DeWolf** Habit: Shrub. Habitat: Woodland, bushland, and riverine forest, 0–300 m. Vouchers: Rawlins SP 168, Magogo FC & Glover PE 684 (EA).

**Capparis sepiaria var. subglabra (Oliv.) DeWolf** Habit: Shrub. Habitat: Bushland, 0–200 m. Vouchers: Luke Q 1441, Mrs. J Adamson 299 RM in FD 1612, Bally 1991 (EA).

**Capparis spinosa var. aegyptia (Lam.) Boiss.** Habit: Liana. Habitat: Bushland and coral outcrops, 0–200 m. Vouchers: SAJIT–005575 (EA, HIB), Taiti S 19 (EA).

***Capparis tomentosa* Lam.** Habit: Liana. Habitat: Bushland, grassland, and riverine, 0–200 m. Vouchers: Ngumbau V & Mwadime N V0190 (EA, HIB), Kuchar P 12903, Sangai GW EA 15762 (EA).

**Capparis viminea Hook. f. & Thomson ex Oliv. var. viminea** Habit: Shrub. Habitat: Forest, ca. 50 m. Voucher: Luke & Robertson 1562 (EA).

***Cladostemon kirkii* (Oliv.) Pax & Gilg** Habit: Tree. Habitat: Forest or thicket, 0–800 m. Vouchers: Festo L & Luke Q 2562, Dale IR 3795 (EA).

***Maerua angolensis* DC.** Habit: Tree. Habitat: Woodland, bushland, and grassland, 0–300 m. Vouchers: Robertson SA 7763 & 5075, Drummond & Hemsley 4218 (EA).

***Maerua calantha* Gilg** Habit: Shrub. Habitat: Bushland, riverine, and, woodland, 45–460 m. Vouchers: Gillett JB 20401, F Thomas 65 (EA).

***Maerua crassifolia* Forssk.** Habit: Tree. Habitat: Bushland or semi-desert scrub, ca. 180 m. Voucher: Robertson SA & Luke WRQ 5585 (EA).

***Maerua decumbens* (Brongn.) DeWolf** Habit: Shrub. Habitat: Bushland, 50 m. Voucher: Robertson SA 7635 (EA).

***Maerua denhardtiorum* Gilg** Habit: Shrub. Habitat: Bushland, 90–780 m. Voucher: Sampson 1(EA).

***Maerua endlichii* Gilg & Gilg-Ben.** Habit: Shrub. Habitat: Bushland, ca. 23 m. Voucher: Ngumbau V & Mwadime N V0456 (EA, HIB).

***Maerua glauca* Chiov.** Habit: Shrub. Habitat: Bushland and thicket, below 100 m. Vouchers: Luke Q & Robertson 2148, Greenway 8964 (EA).

***Maerua grantii* Oliv.** Habit: Shrub. Habitat: Woodland, bushland, and grassland, ca. 50 m. Voucher: Rawlins SP 452 (EA).

***Maerua holstii* Pax** Habit: Liana. Habitat: Lowland rainforest and woodland, 30–450 m. Vouchers: Ngumbau V & Mwadime N V0486 (EA, HIB), Gillett JB 20387 (EA).

***Maerua kaessneri* Gilg & Gilg-Ben.** Habit: Shrub. Habitat: Bushland, 150–350 m. Voucher: Robertson SA & Luke WRQ 5685 (EA).

***Maerua kirkii* (Oliv.) F. White** Habit: Shrub. Habitat: Woodland, bushland, and riverine forest, ca. 100 m. Vouchers: Luke WRQ & Robertson SA 183, Luke WRQ 3330 (EA).

***Maerua macrantha* Gilg** Habit: Shrub. Habitat: Woodland and bushland, 30–50 m. Vouchers: Festo L, Luke Q & P 2709, Rawlins 395, Bally 2155, Drummond & Hemsley 4038 (EA).

***Maerua mungaii* Beentje** Habit: Shrub. Habitat: Woodland, 100–150 m. Voucher: Parker I 513/H (EA): Endemic.

***Maerua sessiliflora* Gilg** Habit: Shrub. Habitat: Deciduous bushland, semi-desert scrub, 300–750 m. Voucher: Bally 2187 (EA).

**Maerua triphylla A. Rich. var. triphylla** Habit: Shrub. Habitat: Forest, ca. 181 m. Vouchers: Ngumbau V & Mwadime N V0223 (EA, HIB).

**Maerua triphylla var. calophylla (Gilg) DeWolf** Habit: Shrub. Habitat: Bushland, grassland, and riverine, ca. 60–200 m. Vouchers: Ngumbau V & Mwadime N V0412 (EA, HIB), Robertson SA & Luke WRQ 5575 (EA).

**Maerua triphylla var. johannis (Volkens & Gilg) DeWolf** Habit: Shrub. Habitat: Bushland and grassland, ca. 200 m. Vouchers: Bally 14389, Drummond 3868 (EA).

**Maerua triphylla var. pubescens (Klotzsch) DeWolf** Habit: Shrub. Habitat: Rainforest, bushland, and grassland, ca. 20 m. Vouchers: Adamson J 6138, Roberson SA 4256 (EA).

***Ritchiea capparoides* (Andrews) Britten** Habit: Shrub. Habitat: Forest and bushland, 0–300 m. Vouchers: Ngumbau V & Mwadime N V0379 (EA, HIB), Hooper SS & Townsend CC 1214 (EA).

***Thilachium africanum* Lour**. Habit: Shrub. Habitat: Woodland, bushland, and grassland, 25–300 m. Vouchers: Luke WRQ & PA 5696, Graham MD 1536 (EA).

***Thilachium roseomaculatum* Y.B. Harv. & Vollesen** Habit: Herb. Habitat: Denser forest parts, 50–150 m. Vouchers: Harvey, Mwachala & Vollesen 49, Robertson & Luke 6251, Luke 3454 & 3472 (EA): Endemic.

***Thilachium thomasii* Gilg** Habit: Shrub. Habitat: Woodland and bushland, ca. 50 m. Vouchers: SAJIT–004646 (EA, HIB), Rawlins SP 133 & 9426, Drummond & Hemsley 4052 (EA).

**F53. Cardiopteridaceae**

1 Genus, 1 Species

***Leptaulus holstii* (Engl.) Engl.** Habit: Shrub. Habitat: Lowland rainforests, ca. 289 m. Vouchers: Ngumbau V & Mwadime N V032 (EA, HIB), Luke WRQ 1852 (EA).

**F54. Caryophyllaceae**

1 Genus, 5 Species

***Polycarpaea corymbosa* (L.) Lam.** Habit: Herb. Habitat: Roadside and grassland, ca. 30 m. Voucher: Festo L, Luke Q & P 2653 (EA).

***Polycarpaea eriantha* Hochst. ex A. Rich.** Habit: Herb. Habitat: Grassland, ca. 480 m. Voucher: Magogo FC & Estes R 1246 (EA).

***Polycarpaea grahamii* Turrill** Habit: Herb. Habitat: Near the shore. Voucher: Graham MD 1617 (EA): Endemic.

***Polycarpaea spicata* Wight ex Arn.** Habit: Herb. Habitat: Woodland, 0–500 m. Voucher: Luke WRQ 5460 (EA).

***Polycarpaea tenuistyla* Turrill** Habit: Herb. Habitat: Bushland, ca. 25 m. Voucher: Musyoki BM & Hansen OJ 982 (EA): Endemic.

**F55. Casuarinaceae**

1 Genus, 1 Species

***Casuarina equisetifolia* L.** Habit: Tree. Habitat: Coastal bushland and seashore, ca. 2 m. Vouchers: SAJIT–006252 (EA, HIB), Greenway 9619, MacNaughton159, Whellan 1851 (EA): Naturalized.

**F56. Celastraceae**

12 Genera, 29 Species

**Apodostigma pallens (Planch. ex Oliv.) R. Wilczek var. pallens** Habit: Liana. Habitat: Forest, woodland, and riverine, ca. 0–239 m. Vouchers: Ngumbau V & Mwadime N V078 & V0306 (EA, HIB), Magogo FC & Glover PE 969, Luke WRQ 3601 (EA).

**Apodostigma pallens var. dummeri N. Hallé** Habit: Liana. Habitat: Forest, ca. 20 m. Vouchers: Mwadime N 18, Luke WRQ 16135 (EA).

***Elachyptera holtzii* (Loes. ex Harms) R. Wilczek** Habit: Liana. Habitat: Evergreen forest to semi-evergreen forest, 120–460 m. Voucher: Luke WRQ 3601 (EA).

***Elachyptera parvifolia* (Oliv.) N. Hallé** Habit: Liana. Habitat: Forest, woodland, ca. 30 m. Vouchers: SAJIT–005527 (EA, HIB), Festo L, Luke Q & P 2696 (EA).

***Elaeodendron schlechterianum* (Loes.) Loes.** Habit: Shrub. Habitat: Woodland and forest, 0–410 m. Vouchers: Ngumbau V & Mwadime N V0303 (EA, HIB), Luke Q 1265, van Someren VGL Sh103 (EA).

***Elaeodendron schweinfurthianum* (Loes.) Loes.** Habit: Shrub. Habitat: Coastal bushland and forest, 0–30 m. Voucher: Magogo FC & Glover PE 1133 (EA).

***Gymnosporia buchananii* Loes.** Habit: Shrub. Habitat: Forest, 60–400 m. Vouchers: Luke WRQ & Robertson SA 500, Magogo FC & Glover PE 267 (EA).

**Gymnosporia gracilis Loes. subsp. gracilis** Habit: Shrub. Habitat: Evergreen forest margins and thicket, 0–500 m. Vouchers: SAJIT–005464 (EA, HIB), Magogo FC & Glover PE 289, Adamson J 5834, Spjut 4550, Brenan, Gillett & Kanuri 14675 (EA).

***Gymnosporia heterophylla* (Eckl. & Zeyh.) Loes.** Habit: Shrub. Habitat: Forest margins and woodland thicket, 0–100 m. Vouchers: Luke Q 1546, Graham RM FD Ox352 (EA).

***Gymnosporia masindei* (Gereau) Jordaan** Habit: Shrub. Habitat: Bushland, 30–245 m. Voucher: Luke WRQ & Robertson SA 2498 (EA).

***Gymnosporia putterlickioides* Loes.** Habit: Tree. Habitat: Woodland and riverine, ca. 30 m. Vouchers: Luke Q 1430, Festo, L & Luke Q 2600, Polhill & Paulo 873 (EA).

***Gymnosporia senegalensis* (Lam.) Loes.** Habit: Shrub. Habitat: Woodland, grassland, and riverine, ca. 45 m. Voucher: Nash LT 40 (EA).

**Loeseneriella africana var. richardiana (Cambess.) R. Wilczek ex N. Hallé** Habit: Liana. Habitat: Riverine forest and thickets, ca. 372 m. Vouchers: SAJIT–006159 (EA, HIB), Mwadime N et al. 615, Luke WRQ 1392 (EA).

**Loeseneriella crenata (Klotzsch) R. Wilczek ex N. Hallé var. crenata** Habit: Liana. Habitat: Forest, bushland, 0–80 m. Voucher: Luke Q 1419 (EA).

***Maytenus undata* (Thunb.) Blakelock** Habit: Tree. Habitat: Forest, riverine, and woodland, 0–407 m. Vouchers: SAJIT–006054 & 005549, Ngumbau V & Mwadime N V0525 (EA, HIB), Luke WRQ & PA 6255 (EA).

**Mystroxylon aethiopicum (Thunb.) Loes. subsp. aethiopicum** Habit: Tree. Habitat: Coastal woodland, 0–250 m. Vouchers: SAJIT–005901, Ngumbau V & Mwadime N V0128 (EA, HIB), Magogo FC & Glover PE 507 (EA).

***Pleurostylia africana* Loes.** Habit: Tree. Habitat: Woodland and forest, 0–500 m. Vouchers: Luke Q 1429, RB Faden & AJ Faden 77/707 (EA).

**Pristimera andongensis var. volkensii (Loes.) N. Hallé & B. Mathew** Habit: Liana. Habitat: Bushland and woodland, ± 100 m. Vouchers: Mwadime N 19, Gray M & Luke WRQ 302 (EA).

***Pristimera longipetiolata* (Oliv.) N. Hallé** Habit: Liana. Habitat: Forest margins, 0–15 m. Voucher: RB & AJ Faden 74/1148 (EA).

***Salacia elegans* Welw. ex Oliv.** Habit: Liana. Habitat: Forest and woodland, 0–250 m. Vouchers: SAJIT–005990 (EA, HIB), Luke Q 1448, Luke WRQ & Robertson SA 2739 (EA).

***Salacia erecta* (G. Don) Walp.** Habit: Liana. Habitat: Evergreen forest, ca. 50 m. Voucher: Luke WRQ & Robertson SA 521 (EA).

***Salacia lehmbachii* Loes.** Habit: Shrub. Habitat: Forest, ca. 45 m. Vouchers: SAJIT–005554 (EA, HIB), Luke WRQ et al. 4725 (EA).

***Salacia leptoclada* Tul.** Habit: Liana. Habitat: Forest, ca. 70 m. Vouchers: Luke Q 1502, Robertson SA & Luke WRQ 6024 (EA).

***Salacia madagascariensis* (Lam.) DC.** Habit: Liana. Habitat: Forest and woodland, 0–600 m. Vouchers: Ngumbau V & Mwadime N V0140, SAJIT–006176 (EA, HIB), Luke Q 43 (EA).

**Salacia sp. cf. erecta (G. Don) Walp.** Habit: Shrub. Habitat: Forest. Voucher: Birch 62/222 (EA).

***Salacia stuhlmanniana* Loes.** Habit: Habitat: Forest, riverine, bushland, and woodland, 0–36 m. Vouchers: SAJIT–006175 (EA, HIB), Robertson SA 5470 (EA).

***Simicratea welwitschii* (Oliv.) N. Hallé** Habit: Liana. Habitat: Forest, ca. 210 m. Vouchers: Ngumbau V & Mwadime N V0377 (EA, HIB), Mwadime N 20, Luke WRQ & Robertson SA 2819 (EA).

***Simirestis goetzei* (Loes.) N. Hallé ex R. Wilczek** Habit: Liana. Habitat: Forest, ca. 90 m. Voucher: Kimeu JM 689 (EA).

***Simirestis scheffleri* (Loes.) N. Hallé** Habit: Liana. Habitat: Forest, ca. 100 m. Voucher: Moggridge 519 (EA).

**F57. Ceratophyllaceae**

1 Genus, 1 Species

***Ceratophyllum demersum* L.** Habit: Herb. Habitat: Water bodies, lakes, rivers, and swamps, ca. 0–15 m. Vouchers: SAJIT–006227 (EA, HIB), Rawlins SP 428 (EA).

**F58. Chrysobalanaceae**

2 Genera, 2 Species

***Dactyladenia barteri* (Hook. f. ex Oliv.) Prance & F. White** Habit: Tree. Habitat: Forest, ca. 410 m. Voucher: Ngumbau V & Mwadime N V0304 (EA, HIB): Cultivated.

**Hirtella zanzibarica Oliv. subsp. zanzibarica** Habit: Tree. Habitat: Forest, ca. 407 m. Vouchers: SAJIT–006030, 005517 & 006063, Ngumbau V & Mwadime M V0250 (EA, HIB), Luke WRQ & Robertson SA 239 (EA).

**F59. Cleomaceae**

4 Genera, 9 Species

***Cleome gynandra* L.** Habit: Herb. Habitat: Waste, disturbed or cultivated ground, ca. 20 m. Voucher: Jeffery K206 (EA).

***Coalisina tenella* (L. f.) Roalson & J.C. Hall** Habit: Herb. Habitat: *Hyphaene* grassland, ca. 5–360 m. Voucher: Jeffery K206 (EA).

***Sieruela allamani* (Chiov.) Roalson & J.C. Hall** Habit: Herb. Habitat: Bushland, semi-desert scrub, 150–175 m. Vouchers: Mrs J Adamson 616, Gillett 19508 (EA).

***Sieruela briquetii* (Polhill) Roalson & J.C. Hall** Habit: Herb. Habitat: Bushland and roadside, ca. 0–210 m. Vouchers: Simpson BL 361, Magogo FC & Glover PE 512 & 1205 (EA).

***Sieruela hirta* (Klotzsch) Roalson & J. C. Hall** Habit: Herb. Habitat: Bushland and grassland, ca. 175 m. Voucher: Festo L & Luke Q 2705 (EA).

***Sieruela parvipetala* (R.A. Graham) Roalson & J.C. Hall** Habit: Herb. Habitat: *Acacia*-*Commiphora*-*Lannea*-*Boswellia* bushland 120–420 m. Vouchers: Mungai GM & Rucina SM 416/84, JB Gillett 16400, J Makin 14472 (EA).

***Sieruela strigosa* (Bojer) Roalson & J.C. Hall** Habit: Herb. Habitat: Sandy seashores, ± 0 m. Vouchers: SAJIT–006262 (EA, HIB), Greenway PJ & Rawlins SP 9422 (EA).

***Sieruela usambarica* (Pax ex Engl.) Roalson & J.C. Hall** Habit: Herb. Habitat: Rainforest and bushland, ca. 30 m. Vouchers: Simpson BL 156, Robertson SA & Luke WRQ 6020 (EA).

***Stylidocleome brachycarpa* (Vahl ex DC.) Roalson & J.C. Hall** Habit: Herb. Habitat: *Acacia*-*Commiphora* bushland. Vouchers: Lucas 34, JB Gillett 21126/B (EA).

**F60. Clusiaceae**

1 Genus, 3 Species

***Garcinia buchananii* Baker** Habit: Tree. Habitat: Evergreen forest, often riverine, and thickets, ca. 85 m. Vouchers: Ngumbau V & Mwadime N V0485 (EA, HIB), Luke WRQ & Robertson SA 1642 & 1899 (EA).

***Garcinia livingstonei* T. Anderson** Habit: Tree. Habitat: Woodland, thicket, and grassland, ca. 5 m. Vouchers: SAJIT–006092 (EA, HIB), Robertson SA 4314 (EA).

***Garcinia volkensii* Engl.** Habit: Shrub. Habitat: Evergreen forest, ca. 210 m. Vouchers: Ngumbau V & Mwadime N V0390 (EA, HIB), Luke Q & Robertson 2655, Luke WRQ 3973 (EA).

**F61. Colchicaceae**

2 Genera, 2 Species

**Gloriosa superba L. var. superba** Habit: Herb. Habitat: Forest margin and coastal bushland, ca. 290 m Vouchers: SAJIT–006261 & 005544, Ngumbau V & Mwadime N V0287 (EA, HIB), Magogo FC & Glover PE 973 (EA).

***Iphigenia oliveri* Engl.** Habit: Herb. Habitat: Grassland and bushland, ca. 0–30 m. Vouchers: Adams 12, Luke et al. 5925, Luke & Luke 4379 (EA).

**F62. Combretaceae**

5 Genera, 31 Species

***Combretum aculeatum* Vent.** Habit: Shrub. Habitat: Bushland, woodland, and riverine, ca. 15 m. Vouchers: Mwadime N 460, 489 & 656, Luke WRQ 121 (EA).

**Combretum apiculatum Sond. subsp. apiculatum** Habit: Tree. Habitat: Woodland, ca. 150 m. Vouchers: Msafiri F & Mwendwa H 441, Luke WRQ 3471 (EA).

***Combretum butyrosum* (G. Bertol.) Tul.** Habit: Liana. Habitat: Riverine forest, 50–450 m. Vouchers: Ngumbau V & Mwadime N V0453 (EA, HIB), Festo L, Luke Q & P 2755 (EA).

***Combretum chionanthoides* Engl. & Diels** Habit: Liana. Habitat: Forest margin, 50–170 m. Vouchers: Ngumbau V & Mwadime N V0413 (EA, HIB), Brenan JPM & JH Gillet JB 14657 (EA).

***Combretum constrictum* (Benth.) M. A. Lawson** Habit: Climber. Habitat: Swamps and riverine forest, ca. 15 m. Vouchers: RM Graham in FD 2243, Festo 2419 (EA).

***Combretum contractum* Engl. & Diels** Habit: Shrub. Habitat: Bushland, 150–396 m. Vouchers: Graham RM 2047, Bally 8162, Dale 2049 (EA).

***Combretum exalatum* Engl.** Habit: Shrub. Habitat: Bushland, ca. 300 m. Vouchers: Festo L, Luke Q & P 2789, Robertson SA 6396 (EA).

***Combretum falcatum* (Welw. ex Hiern) Jongkind** Habit: Liana. Habitat: Bushland and forest margins, 0–70 m. Vouchers: Ngumbau V & Mwadime N V0530 & V0436 (EA, HIB), Luke WRQ 894 (EA).

**Combretum hereroense Schinz subsp. hereroense** Habit: Shrub. Habitat: Grassland and bushland, ca. 70 m. Vouchers: Ngumbau V & Mwadime N V0448 (EA, HIB), Dale in FD 3666, Robertson SA & Luke WRQ 5715 (EA).

**Combretum hereroense subsp. volkensii (Engl.) Wickens** Habit: Shrub. Habitat: Grassland or bushland, 50–500 m. Vouchers: Power 10994, Hildebrandt 2337, Luke 916 (EA).

***Combretum holstii* Engl.** Habit: Liana. Habitat: Woodland and riverine forest, ca. 0–300 m. Voucher: Robertson SA 5084 (EA).

***Combretum illairii* Engl.** Habit: Liana. Habitat: Forest, woodland, and bushland, 30–800 m. Vouchers: SAJIT–004665 & 005569, Ngumbau V & Mwadime N V0228 (EA, HIB), Kuchar P 13482 (EA).

***Combretum molle* R.Br. ex G. Don** Habit: Tree. Habitat: Wooded grassland, 30–340 m. Vouchers: Spjut RW 3809, Shimba Hills Survey Unit 30, Drummond RB & Hemsley JH 4101 (EA).

***Combretum padoides* Engl. & Diels** Habit: Liana. Habitat: Riverine forest, swamp forest, 0–280 m. Vouchers: SAJIT–006070, Ngumbau V & Mwadime N V0324 (EA, HIB), Robertson SA & Luke WRQ 5647, Luke WRQ & Robertson SA 2718 (EA).

***Combretum paniculatum* Vent.** Habit: Liana. Habitat: Forest, woodland, and riverine forest, 10–156 m. Vouchers: Ngumbau V & Mwadime N V0429 (EA, HIB), Graham RM 1694, Rawlins SP 253 (EA).

***Combretum pentagonum* M.A. Lawson** Habit: Liana. Habitat: Forest and thicket, 20–153 m. Vouchers: Ngumbau V & Mwadime N V0505 (EA, HIB), Robertson SA & Luke WRQ 4516 (EA).

***Combretum schumannii* Engl.** Habit: Tree. Habitat: Forest, riverine forest, and woodland, 0–140 m. Vouchers: Ngumbau V & Mwadime N V0538 (EA, HIB), Kuchar P 13548 (EA).

***Combretum tenuipetiolatum* Wickens** Habit: Tree. Habitat: Coastal forest, ca. 100 m. Voucher: Robertson SA & Luke WRQ 2289 (EA): Critically Endangered.

***Combretum umbricola* Engl.** Habit: Liana. Habitat: Riverine forest and coastal woodland, ca. 280 m. Voucher: Luke WRQ & Robertson SA 2717 (EA).

***Conocarpus lancifolius* Engl. & Diels** Habit: Tree. Habitat: Semi-desert coastal zone, 10–570 m. Voucher: Gillett JB 20314 (EA): Near Threatened, Cultivated.

***Lumnitzera racemosa* Willd.** Habit: Shrub. Habitat: Mangrove swamps, 0–30 m. Vouchers: Mrs Robertson SA 7747, Luke Q 5649, Brathay 1982 Expedition 55 (EA).

***Pteleopsis myrtifolia* (M.A. Lawson) Engl. & Diels** Habit: Tree. Habitat: Riverine, forest, woodland, bushland, and grassland, 0–150 m. Vouchers: Ngumbau V & Mwadime N V0530 (EA, HIB), Luke WRQ & Robertson SA 2822, Faden 70/256 (EA).

***Pteleopsis tetraptera* Wickens** Habit: Tree. Habitat: Bushland, wooded grassland, and forest, 0–300 m. Vouchers: Luke WRQ & Robertson SA 5531, Luke WRQ & Robertson SA 2796 (EA).

**Terminalia boivinii Tul.** Habit: Shrub. Habitat: Bushland and forest, 0–30 m. Vouchers: SAJIT–005585 (EA, HIB), Festo L, Luke Q & P 2699, Moomaw 1602, Donald 96 in FD 2496 (EA).

***Terminalia brevipes* Pamp.** Habit: Tree. Habitat: Riverine forest and bushland, 20–280 m. Vouchers: Brathay Expedition 46, Tweedie 1866 (EA).

***Terminalia catappa* L.** Habit: Tree. Habitat: Sandy or rocky beaches, ca. 5 m. Vouchers: Ndakala J 407, Faden RB & Faden AJ 77/390 (EA): Naturalized.

***Terminalia parvula* Pamp.** Habit: Tree. Habitat: Bushland, ca. 60 m. Voucher: Bally 16708 (EA).

***Terminalia prunioides* M.A. Lawson** Habit: Tree. Habitat: Bushland and riverine thicket, 30–360 m. Vouchers: Ngumbau V & Mwadime N V0453 (EA, HIB), Rawlins SP 335, Luke WRQ & Robertson SA 2113 (EA).

***Terminalia sambesiaca* Engl. & Diels** Habit: Tree. Habitat: Forest, riverine forest, and woodland, 70–830 m. Vouchers: Mohamed Abdulla 1159, Verdcourt 3934, Dale in FD 3660 (EA).

**Terminalia sp. aff. spinosa Engl.** Habit: Tree. Habitat: Forest. Voucher: Luke et al. TPR766 (EA).

***Terminalia spinosa* Engl.** Habit: Tree. Habitat: Bushland, woodland, and grassland, 0–330 m. Vouchers: SAJIT–006440 (EA, HIB), Rawlins SP 246, Beentje HJ 2275 (EA).

**F63. Commelinaceae**

5 Genera, 31 Species

***Aneilema aequinoctiale* (P. Beauv.) Loudon** Habit: Herb. Habitat: Moist or dry evergreen forests, 0–150 m. Vouchers: Robertson SA & Luke WRQ 5156, RB Faden & AJ Faden 290 & 1270, Polhill & Paulo 825 (EA).

***Aneilema calceolus* Brenan** Habit: Herb. Habitat: Evergreen forest, 10–450 m. Vouchers: Faden & Faden 77/378, 74/1069 & 77/565 (EA).

***Aneilema clarkei* Rendle** Habit: Herb. Habitat: Evergreen forest, woodland, and thicket, 0–250 m. Vouchers: Luke Q 1457, Faden & Evans 71/714, Faden & Faden 77/714 (EA).

**Aneilema hockii De Wild. subsp. hockii** Habit: Herb. Habitat: Bushland, woodland, and grassland, 15–160 m. Vouchers: Luke & Robertson 2565, Faden & Faden 74/1065 (EA).

**Aneilema indehiscens Faden subsp. indehiscens** Habit: Herb. Habitat: Bushland and thicket, ca. 15 m. Vouchers: Faden & Faden 74/380 & 74/1184 (EA).

***Aneilema lamuense* Faden** Habit: Herb. Habitat: Sand dunes, 0–50 m. Voucher: Faden & Faden 74/1083 (EA).

***Aneilema nyasense* C.B. Clarke** Habit: Herb. Habitat: Forest, swamp, riverine, and bushland, 50–300 m. Vouchers: Luke WRQ et al. 3379 & 4735 (EA).

**Aneilema petersii (Hassk.) C.B. Clarke subsp. petersii** Habit: Herb. Habitat: Grassland, bushland, wooded grassland, and woodland, 0–500 m. Vouchers: Luke WRQ 3470, Q Luke & SA Robertson 2495, SA Robertson & Q Luke 6320 (EA).

***Aneilema succulentum* Faden** Habit: Herb. Habitat: Bushland, forest, and woodland, 10–600 m. Vouchers: Faden et al. 72/39, Faden & Faden 74/1152 (EA): Endemic.

***Aneilema tanaense* Faden** Habit: Herb. Habitat: Bushland, 10–250 m. Vouchers: Faden & Faden 77/582, 77/ 738 & 74/1185 (EA): Endemic.

***Aneilema taylorii* C.B. Clarke** Habit: Herb. Habitat: Riverine forest, 30–400 m. Vouchers: Verdcourt 2410 & 1921, Faden & Faden 77/611 (EA).

***Coleotrype bruechneriana* Mildbr.** Habit: Herb. Habitat: Forest and riverine, ca. 150–320 m. Vouchers: Luke WRQ & PA 8193, Brenan et al. 14587, Luke & Luke 8193 (EA).

**Commelina africana subsp. zanzibarica Faden** Habit: Herb. Habitat: Forest, riverine, and grassland, 0–250 m. Vouchers: Faden & Faden 74/1123, Greenway & Rawlins 9343 (EA).

***Commelina benghalensis* L.** Habit: Herb. Habitat: Palm forest, 0–400 m. Vouchers: Graham RM 2005, Meester-Manger Cats V de 38, Robertson SA 5221 (EA).

***Commelina bracteosa* Hassk.** Habit: Herb. Habitat: Forest, woodland, grassland, bushland, and cultivated lands, 45–305 m. Vouchers: Ngumbau V & Mwadime N V0290 & 084 (EA, HIB), Luke Q 1456, Robertson SA & Luke WRQ 5984 (EA).

***Commelina erecta* L.** Habit: Herb. Habitat: Forest and riverine forest, 0–550 m. Vouchers: Ngumbau V & Mwadime N V055 &0338 (EA, HIB), Robertson 7386, Faden & Faden 74/1068 (EA).

***Commelina forskaolii* Vahl** Habit: Herb. Habitat: Grassland, bushland, woodland, forest, cultivated lands, and riverine, ca. 30 m. Voucher: Faden & Faden 74/1249 (EA).

***Commelina livingstonii* C.B. Clarke** Habit: Herb. Habitat: Grassland, woodland, and bushland, ca. 100 m. Voucher: Faden & Faden 74/1189 (EA).

***Commelina lukei* Faden** Habit: Herb. Habitat: Forest, bushland, grassland, and roadside, ca. 5 m. Vouchers: Verdcourt 1056 & 1851, SA Robertson 7377, Q Luke & et al. 7080 (EA).

***Commelina mascarenica* C.B. Clarke** Habit: Herb. Habitat: Roadside, grassland, and open bushland, ca. 230 m. Vouchers: Ngumbau V & Mwadime N V0230 (EA, HIB), Rayner 294 (EA).

***Commelina petersii* Hassk.** Habit: Herb. Habitat: Moist forest, ca. 297 m. Voucher: Ngumbau V & Mwadime N V075 (EA, HIB).

**Commelina sp. aff. bracteosa Hassk.** Habit: Herb. Habitat: Forest. Voucher: R & L 6018 (EA).

**Commelina sp. aff. erecta L.** Habit: Herb. Habitat: Forest. Voucher: K et al. TPR638 (EA).

***Commelina zambesica* C.B. Clarke** Habit: Herb. Habitat: Grassland, woodland, and forest, ca. 186 m. Vouchers: Ngumbau V & Mwadime N V0339 (EA, HIB), Magogo & Glover 70/562 (EA).

***Cyanotis axillaris* (L.) D. Don ex Sweet** Habit: Herb. Habitat: Wooded grassland and water bodies, 5–300 m. Voucher: Faden & Faden 74/1211 (EA).

***Cyanotis foecunda* DC. ex Hassk.** Habit: Herb. Habitat: Woodland, bushland, thickets, and grassland, ca. 230 m. Vouchers: Robertson SA & Luke WRQ 6151, Luke Q & SA Robertson 2523 (EA).

***Cyanotis lanata* Benth.** Habit: Herb. Habitat: Grassland, bushland, and woodland, ca. 50 m. Voucher: Polhill R & Paulo S 787 (EA).

**Cyanotis repens Faden & D. M. Cameron subsp. repens** Habit: Herb. Habitat: Dry or moist forest, ca. 250 m. Vouchers: Robertson SA & Luke WRQ 6017, Luke Q 1455, Faden & Faden 74/1174, Adams 98 (EA).

***Cyanotis* sp. 1** Habit: Herb. Habitat: Forest. Voucher: Rawlins 455 (EA).

***Murdannia blumei* (Hassk.) Brenan** Habit: Herb. Habitat: Swamps, ± 10 m. Vouchers: Greenway PJ & Rawlins 9374, Luke Q 5684 (EA).

***Murdannia simplex* (Vahl) Brenan** Habit: Herb. Habitat: Grassland, bushland, and woodland, forest, ca. 239 m. Vouchers: SAJIT–005993 (EA, HIB), Magogo FC & Glover PE 366 (EA).

**F64. Connaraceae**

4 Genera, 6 Species

***Agelaea pentagyna* (Lam.) Baill.** Habit: Liana. Habitat: Riverine forest, coastal forest, and woodland, 0–150 m. Voucher: Luke WRQ 902 (EA).

***Connarus longistipitatus* Gilg** Habit: Liana. Habitat: Woodland, ca. 300 m. Vouchers: Luke WRQ & Robertson SA 2728, Graham Q 290 in FD 1695 (EA).

***Ellipanthus madagascariensis* (G. Schellenb.) Capuron ex Keraudren** Habit: Tree. Habitat: Forest, 0–100 m. Vouchers: SAJIT–005929 & 005513 (EA, HIB), Faden RB & Faden AJ 77/570, Luke WRQ & Robertson SA 216 (EA): Near Threatened.

**Rourea coccinea subsp. boiviniana (Baill.) Jongkind** Habit: Shrub. Habitat: Coastal forest, bushland, riverine, and woodland, 0–750 m. Vouchers: Ngumbau V & Mwadime N V0492 (EA, HIB), Kuchar P 13484, Luke WRQ 3128 (EA).

***Rourea minor* (Gaertn.) Alston** Habit: Liana. Habitat: Woodland, riverine, and forest, ca. 250 m. Vouchers: Luke WRQ & Robertson SA 2722, Drummond & Hemsley 4034A (EA).

***Rourea orientalis* Baill.** Habit: Shrub. Habitat: Forest, woodland, and bushland, ca. 156 m. Vouchers: SAJIT–005478, Ngumbau V & Mwadime N V0432 (EA, HIB), Luke Q 1506, Luke WRQ & Robertson SA 514 (EA).

**F65. Convolvulaceae**

14 Genera, 44 Species

**Astripomoea hyoscyamoides (Vatke) Verdc. var. hyoscyamoides** Habit: Herb. Habitat: Grassland, bushland, roadside, and cultivated area, ca. 50–100 m. Vouchers: Robertson SA & Luke WRQ 6197, Jeffrey 8, Fukuoka N 276 (EA).

**Astripomoea malvacea (Klotzsch) A. Meeuse var. malvacea** Habit: Herb. Habitat: Woodland and grassland, 60 m. Voucher: Kassner 437 (EA).

***Convolvulus jefferyi* Verdc.** Habit: Climber. Habitat: Grassland, forest, and sand dunes, 0–150 m. Vouchers: Luke Q 5447, Jeffery 321, Irwin 271 (EA).

***Cressa cretica* L.** Habit: Herb. Habitat: Coastal dunes or sandy places, ± 0 m. Vouchers: Luke Q 5474, Rawlins 182 & 391 (EA).

***Evolvulus alsinoides* (L.) L.** Habit: Herb. Habitat: Grassland, woodland, roadside, and cultivated area, ca. 100 m. Vouchers: Magogo FC & Glover PE 817, Battiscombe 774 (EA).

***Hewittia malabarica* (L.) Suresh** Habit: Climber. Habitat: Grassland, bushland, forest edges, and waste places, ca. 0–200 m. Vouchers: SAJIT–006205, Ngumbau V & Mwadime N V0286 & 0396 (EA, HIB), Magogo FC & Glover PE 73 (EA).

***Ipomoea albivenia* (Lindl.) Sweet** Habit: Climber. Habitat: Coastal bushland, 50–200 m. Voucher: Wakefield (EA).

***Ipomoea aquatica* Forssk.** Habit: Herb. Habitat: Swampy, ca. 50 m. Voucher: Greenway & Rawlins 9450 (EA).

***Ipomoea barteri* A. Chev.** Habit: Herb. Habitat: Grassland and woodland, 0–200 m. Voucher: Whyte (EA).

***Ipomoea bullata* Oliv.** Habit: Climber. Habitat: Bushland, ca. 280 m. Voucher: Luke WRQ & Robertson SA 2212 (EA).

***Ipomoea cairica* (L.) Sweet** Habit: Climber. Habitat: Seashores, forest clearings, and grassland, ca. 15 m. Voucher: Simpson BL 118 (EA).

**Ipomoea coptica (L.) Roth ex Roem. & Schult. var. coptica** Habit: Herb. Habitat: Grassland, woodland, roadside, and cultivated area, ca. 190 m. Vouchers: Drummond & Hemsley 4239, Rawlins 826 (EA).

**Ipomoea coptica var. acuta Choisy.** Habit: Climber. Habitat: Cultivated area, 0–10 m. Voucher: Festo L & Luke Q 2583 (EA).

***Ipomoea eriocarpa* R.Br.** Habit: Climber. Habitat: Grassland and cultivated ground, ca. 0–150 m. Vouchers: Festo L & Luke Q 2468, Magogo FC & Glover PE 1059 (EA).

***Ipomoea ficifolia* Lindl.** Habit: Climber. Habitat: Bushland and cultivated ground, 20–390 m. Vouchers: Herbarium Techn. Course II 054, Irwin 277, Festo L & Luke Q 2527 (EA).

***Ipomoea garckeana* Vatke** Habit: Climber. Habitat: Bushland and cultivated ground, 30–350 m. Vouchers: Drummond & Hemsley 4083, Jeffery 544 (EA, HIB).

**Ipomoea hildebrandtii Vatke subsp. orientalis Verdc.** Habit: Climber. Habitat: Grassland or bushland, ca. 304 m. Vouchers: Bally PRO 16798, Irwin 156 (EA).

***Ipomoea irwiniae* Verdc.** Habit: Climber. Habitat: Bushland and grassland, 0–500 m. Vouchers: Irwin PH 276, Luke WRQ & PA 5688, Jeffery 49 (EA).

***Ipomoea kotschyana* Hochst. ex Choisy** Habit: Herb. Habitat: Grassland, ± 0 m. Vouchers: Tweedie 1057 & 1874, Irwin 273 (EA).

***Ipomoea marginata* (Desr.) Verdc.** Habit: Climber. Habitat: Swampy, wooded grassland, and woodland, ca. 100 m. Vouchers: Magogo FC & Glover PE 999, Festo L & Luke Q 2360 (EA).

***Ipomoea mauritiana* Jacq.** Habit: Liana. Habitat: Rainforest, riverine, and bushland, ca. 65 m. Vouchers: Irwin 440, Mwadime N & Chesire 236 (EA).

**Ipomoea mombassana Vatke subsp. mombassana** Habit: Climber. Habitat: Bushland and woodland, ca. 0–304 m. Vouchers: Kuchar P 11819, Irwin PH 164 & 280 (EA).

***Ipomoea obscura* (L.) Ker-Gawl.** Habit: Climber. Habitat: Bushland, grassland, and woodland, ca. 20 m. Vouchers: Gray M & Luke WRQ 292, Festo 2647 9 (EA).

***Ipomoea ochracea* (Lindl.) G. Don** Habit: Herb. Habitat: Forest, ca. 60–365 m. Vouchers: Drummond & Hemsley 4079, Polhill R & Paulo S 917 (EA).

**Ipomoea pes-caprae subsp. brasiliensis (L.) Ooststr.** Habit: Herb. Habitat: Sandy shores, and roadside, 0–750 m. Vouchers: SAJIT–006251 (EA, HIB), Verdcourt 1175, Kirika P, Muthoka P & Mbale M 761 (EA).

***Ipomoea pes-tigridis* L.** Habit: Climber. Habitat: Grassland and thickets, 0–183 m. Vouchers: Ngumbau V & Mwadime N V0193 (EA, HIB), Luke Q 5632 (EA).

***Ipomoea pileata* Roxb.** Habit: Climber. Habitat: Rainforest, bushland, and grassland, ca. 75 m. Vouchers: Graham 1963, Jeffery 270 (EA).

***Ipomoea shupangensis* Baker** Habit: Climber. Habitat: Forest, bushland, and coral, ca. 20 m. Vouchers: Mwadime N 43, Pakia M et al. 986, Luke WRQ 10704 (EA).

***Ipomoea ticcopa* Verdc.** Habit: Herb. Habitat: Grassland, 0–25 m. Vouchers: SAJIT–006245 (EA, HIB), Tweedie 982 (EA).

***Ipomoea urbaniana* (Dammer) Hallier f.** Habit: Climber. Habitat: Forest, grassland, ca. 10 m. Vouchers: Robertson SA 4208, Irwin 394, Luke 3078 (EA).

**Ipomoea wightii var. kilimandschari (Dammer) Verdc.** Habit: Climber. Habitat: Open forest and scrub, ca. 50 m. Voucher: Graham RM 509 (EA).

***Ipomoea violacea* L.** Habit: Herb. Habitat: Bushland or coral, ± 0 m. Vouchers: Jeffery 381, Bally 1894, Irwin 260 (EA).

***Jacquemontia ovalifolia* (Choisy) Hallier f.** Habit: Climber. Habitat: Sandy beaches, 10–750 m. Vouchers: Kirika P, Muthoka P & Mbale M 756, Festo 2447, Hooper & Townsend 1194 (EA).

***Jacquemontia paniculata* (Burm. f.) Hallier f.** Habit: Climber. Habitat: Grassland, 0–360 m. Vouchers: Ngumbau V & Mwadime N V0231 (EA, HIB), Magogo FC & Glover PE 1048 (EA).

***Jacquemontia tamnifolia* (L.) Griseb.** Habit: Climber. Habitat: Grassland and cultivated place, ca. 272 m. Vouchers: Ngumbau V & Mwadime N V0272 (EA, HIB), Magogo FC & Glover PE 861 (EA).

**Merremia ampelophylla Hallier f. var. ampelophylla** Habit: Herb. Habitat: Bushland or roadside, ca. 20 m. Vouchers: C van Someren in EAH 10560, Festo 2645 (EA).

***Merremia semisagitta* (Peter) Dandy** Habit: Herb. Habitat: Not known, ca. 20m. Voucher: Gregory (EA).

***Merremia* sp. C of FTEA** Habit: Herb. Habitat: Woodland, ca. 90 m. Voucher: Rawlins 864 (EA): Endemic.

***Metaporana densiflora* (Hallier f.) N. E. Br.** Habit: Liana. Habitat: Forest edge, 50–250 m. Vouchers: Ngumbau V & Mwadime N V0562 (EA, HIB), Mwadime N 24, Drummond & Hemsley 4245 (EA).

***Operculina turpethum* (L.) Silva Manso** Habit: Herb. Habitat: Swampy, ca. 100 m. Voucher: Rawlins 188 (EA).

**Seddera hirsuta Hallier f. var. hirsuta** Habit: Herb. Habitat: Bushland, 90–460 m. Voucher: Drummond & Hemsley 4069 (EA).

***Seddera suffruticosa* (Schinz) Hallier f.** Habit: Herb. Habitat: Grassland, 0–250 m. Voucher: Drummond & Hemsley 3768 (EA).

***Stictocardia incompta* (Hallier f.) Hallier f.** Habit: Climber. Habitat: Mangrove forest, bushland, 0–800 m. Voucher: Luke WRQ 1322 (EA).

***Stictocardia macalusoi* (Mattei) Verdc.** Habit: Climber. Habitat: Sand dunes and sea shore, ± 0 m. Voucher: Luke Q 5482 (EA).

***Xenostegia tridentata* (L.) D. F. Austin & Staples** Habit: Climber. Habitat: Grassland, wooded and grassland, 30–213 m. Vouchers: Ngumbau V & Mwadime N V0330 (EA, HIB), Magogo FC & Glover PE 151, Robertson SA 4225 (EA).

**F66. Costaceae**

1 Genus, 1 Species

***Costus afer* Ker Gawl.** Habit: Herb. Habitat: Moist forests at forest edges, 160–210 m. Vouchers: Ngumbau V & Mwadime N V0375 (EA, HIB), Magogo FC & Glover PE 563, Drummond RB & Hemsley JH 1200 (EA).

**F67. Crassulaceae**

1 Genus, 7 Species

***Kalanchoe ballyi* Raym.-Hamet ex Cufod.** Habit: Herb. Habitat: Coastal woodland, ca. 85 m. Vouchers: Ngumbau V & Mwadime N V0414 (EA, HIB), Robertson SA & Luke WRQ 5139 (EA): Endemic.

***Kalanchoe fadeniorum* Raadts** Habit: Herb. Habitat: Semi-evergreen thicket on sandy soils, ca. 360 m. Voucher: RB & AJ Faden 77/777 (EA): Extinct in the wild, Endemic.

***Kalanchoe glaucescens* Britten** Habit: Herb. Habitat: Bushland, grassland, and forest, ca. 550 m. Voucher: Reitsma J 508 (EA).

**Kalanchoe nyikae Engl. subsp. nyikae** Habit: Herb. Habitat: Bushland, ca. 304 m. Vouchers: Jeffery GW 248, Luke WRQ & Robertson SA 2223 (EA).

**Kalanchoe lateritia Engl. var. lateritia** Habit: Herb. Habitat: Woodland and lowland forest, 1–200 m. Vouchers: Robertson SA & Luke WRQ 6488, 6002 & 4925, Drummond & Hemsley 3790 (EA).

***Kalanchoe obtusa* Engl.** Habit: Herb. Habitat: Evergreen to semi-deciduous forest, 1–450 m. Voucher: Robertson SA 5442 (EA).

***Kalanchoe rotundifolia* (Haw.) Haw.** Habit: Herb. Habitat: Bushland, 500–700 m. Voucher: Rauh W 860 (EA).

**F68. Cucurbitaceae**

18 Genera, 48 Species

***Cephalopentandra ecirrhosa* (Cogn.) C. Jeffrey** Habit: Climber. Habitat: Deciduous woodland and bushland, ca. 45 m. Voucher: Bally PRO 2057 (EA).

***Citrullus lanatus* (Thunb.) Matsum. & Nakai** Habit: Climber. Habitat: Grassland and bushland ca. 50 m. Vouchers: Jeffery GW 135, Drummond & Hemsley 44 (EA): Cultivated.

***Coccinia grandiflora* Cogn. ex Engl.** Habit: Climber. Habitat: Lowland and upland rainforest, 100–269 m. Vouchers: Ngumbau V & Mwadime N V003 (EA, HIB), Kimeu JM 522, Drummond & Hemsley 3874 (EA).

***Coccinia grandis* (L.) Voigt** Habit: Climber. Habitat: Deciduous bushland and woodland, 0–199 m. Vouchers: SAJIT–006116 & 006222, Ngumbau V & Mwadime N V0427 (EA, HIB), Festo L, Luke Q & P 2800, Drummond & Hemsley 3901 (EA).

***Coccinia microphylla* Gilg** Habit: Climber. Habitat: Lowland rainforest, ca. 180 m. Vouchers: Milne-Redhead E & Taylor P 7104, Drummond & Hemsley 4087 & 4040 (EA).

***Coccinia pwaniensis* Holstein** Habit: Climber. Habitat: Lowland rainforest, 80–300 m. Vouchers: Drummond & Hemsley 1078 & 3953, Saunders, FC Magogo & P Glover 493 (EA).

***Coccinia trilobata* (Cogn.) C. Jeffrey** Habit: Climber. Habitat: Wooded grassland and deciduous bushland, ca. 30 m. Voucher: Simpson BL 383 (EA).

***Corallocarpus ellipticus* Chiov.** Habit: Climber. Habitat: Bushland on coral sand near sea level. Vouchers: Robertson SA 3809, Bogdan 4723, Lucas, Jeffrey & Kirrika 266 (EA).

***Corallocarpus epigaeus* (Rottler) C. B. Clarke** Habit: Climber. Habitat: Wooded grassland and deciduous bushland, ca. 550 m. Voucher: Williams JG 12588 (EA).

***Ctenolepis cerasiformis* (Stocks) Hook. f.** Habit: Climber. Habitat: Deciduous bushland and thicket, ca. 5 m. Vouchers: Kimeu JM 657, Rawlins 843, Mwadime N 438 (EA).

***Cucumis aculeatus* Cogn.** Habit: Climber. Habitat: Forest edge, ca. 142 m. Voucher: SAJIT–005999 (EA, HIB).

***Cucumis dipsaceus* Ehrenb. ex Spach** Habit: Climber. Habitat: Bushland and woodland, ca. 425 m. Voucher: Fukuoka N 250 (EA).

***Cucumis engleri* (Gilg) Ghebret. & Thulin** Habit: Climber. Habitat: Bushland, wooded grassland, and grassland, ca. 330 m. Voucher: Drummond & Hemsley 4220 (EA).

***Cucumis ficifolius* A. Rich.** Habit: Climber. Habitat: Deciduous bushland and grassland, 0–300 m. Voucher: Jeffery GW 696 (EA).

***Cucumis maderaspatanus* L.** Habit: Climber. Habitat: Seasonal swamps, flood plain, and valley grassland, ca. 30 m. Vouchers: Cunningham-van Someren GR 280, Nyange M 406 (EA).

**Cucumis prophetarum subsp. dissectus (Naudin) C. Jeffrey** Habit: Climber. Habitat: Grassland, ca. 60 m. Vouchers: Luke WRQ & PA 4574B, Drummond & Hemsley 3994 & 4206 (EA).

***Cucumis sacleuxii* Paill. & Bois** Habit: Climber. Habitat: Rain and swamp or other ground water forest, ca. 210 m. Vouchers: Ngumbau V & Mwadime N V0371 (EA, HIB), Rawlins SP 56, Luke WRQ & PA 5687 (EA).

***Cucumis* sp. *(Oreosyce* sp. A of FTEA)** Habit: Climber. Habitat: Bushland. Vouchers: Rawlins, Gillespie 242 (EA): Endemic.

***Cyclantheropsis parviflora* (Cogn.) Harms** Habit: Climber. Habitat: Lowland evergreen forest, deciduous woodland, and bushland, ca. 70 m. Voucher: Ngumbau V & Mwadime N V0443 (EA, HIB).

***Diplocyclos palmatus* (L.) C. Jeffrey** Habit: Climber. Habitat: Swamps, ground water forest, and seasonal swamp grasslands, ca. 0– 50 m. Vouchers: Sangai GW 15723, Polhill & Paulo 720 (EA).

***Diplocyclos tenuis* (Klotzsch) C. Jeffrey** Habit: Climber. Habitat: Coastal bushland, 0–90 m. Voucher: Robertson SA 3423 (EA).

***Eureiandra orientalis* Q. Luke, ined.** Habit: Climber. Habitat: Coastal forest and bushland, 0–100 m. Vouchers: Ngumbau V & Mwadime N V0409 & V0332 (EA, HIB), Gray M & Luke WRQ 282 (EA).

***Gerrardanthus grandiflorus* Gilg ex Cogn.** Habit: Climber. Habitat: Lowland rainforest, ca. 80 m. Vouchers: Ngumbau V & Mwadime N V072 (EA, HIB), Drummond & Hemsley 3958 (EA).

***Gerrardanthus lobatus* (Cogn.) C. Jeffrey** Habit: Climber. Habitat: Deciduous bushland, woodland, and wooded grassland, 0–400 m. Vouchers: Drummond & Hemsley 41158, Robertson SA 6398 (EA).

***Kedrostis abdallae* Zimm.** Habit: Climber. Habitat: Forest, ca. 35 m. Voucher: Luke Q 1529 (EA).

***Kedrostis foetidissima* (Jacq.) Cogn.** Habit: Climber. Habitat: Deciduous and semi-evergreen woodland, ca. 400 m. Vouchers: Faden RB & AJ 74/1196, Robertson SA 3491 (EA).

***Kedrostis heterophylla* Zimm.** Habit: Climber. Habitat: Lowland rainforest, 40–300 m. Vouchers: SAJIT–006138 (EA, HIB), Luke WRQ 1833, Luke WRQ & Robertson SA 2813 (EA).

***Kedrostis leloja* (Forssk. ex J.F Gmel.) C. Jeffrey** Habit: Climber. Habitat: Deciduous bushland and wooded grassland, 10–550 m. Vouchers: SAJIT–006129, Ngumbau V & Mwadime N V0245 (EA, HIB), Luke WRQ & Luke Q 2458 (EA).

***Lagenaria siceraria* (Molina) Standl.** Habit: Climber. Habitat: Cultivated area. Voucher: Waaijenberg 29 (EA).

***Lagenaria sphaerica* (Sond.) Naudin** Habit: Climber. Habitat: Evergreen and deciduous forests, 0–50 m. Vouchers: Katz SS 197/75, Drummond & Hemsley 3903, Nyange M 473 (EA).

***Luffa cylindrica* M. Roem.** Habit: Climber. Habitat: *Hyphaene* woodland, 5–15 m. Vouchers: Festo L & Luke Q 2476, Leauthaud C 120 (EA): Naturalized.

***Momordica anigosantha* Hook. f.** Habit: Climber. Habitat: Lowland, groundwater forest and forest remnants, ca. 300 m. Vouchers: Ngumbau V & Mwadime N V0221 (EA, HIB), Faden RB 70/822 (EA).

***Momordica boivinii* Baill.** Habit: Climber. Habitat: Deciduous bushland, thicket, woodland, and wooded grassland, 0–200 m. Vouchers: SAJIT–005951, Ngumbau V & Mwadime N V0345 (EA, HIB), Luke & Robertson 2291, Magogo FC & Glover PE 215 (EA).

***Momordica calantha* Gilg** Habit: Climber. Habitat: Forests edge and valley grasslands, ca. 100 m. Vouchers: Ngumbau V & Mwadime N V119 (EA, HIB), Kabuye CHS, Gilbert VC & Robertson A 115, Magogo FC & Glover PE 306 (EA).

***Momordica charantia* L.** Habit: Climber. Habitat: Lowland rainforest and riverine forest, 100 m. Voucher: Verdcourt 3942 (EA).

***Momordica leiocarpa* Gilg** Habit: Climber. Habitat: Lowland rainforest, 60–300 m. Vouchers: Drummond & Hemsley 1196, Verdcourt 1875, Luke WRQ 2890 (EA).

***Momordica littorea* Thulin** Habit: Climber. Habitat: Bushland, 10–150 m. Voucher: Luke Q 5440 (EA).

***Momordica peteri* Zimm.** Habit: Climber. Habitat: Lowland rainforest, ca. 280 m. Vouchers: Reitsma J 344, Luke WRQ 1638 (EA).

***Momordica rostrata* Zimm.** Habit: Climber. Habitat: Deciduous bushland, thicket, woodland, and wooded grassland, 15–350 m. Vouchers: Ngumbau V & Mwadime N V0447 (EA, HIB), Kuchar P 12907, Drummond & Hemsley 4110 (EA).

***Momordica spinosa* (Gilg) Chiov.** Habit: Climber. Habitat: Deciduous bushland and semi-desert scrub, ca. 330 m. Voucher: Bally (EA).

***Momordica trifoliolata* Hook. f.** Habit: Climber. Habitat: Deciduous bushland and woodland, ca. 0–86 m. Vouchers: Ngumbau V & Mwadime N V0478 (EA, HIB), Stuhlmann 6970 (EA).

***Peponium vogelii* (Hook. f.) Engl.** Habit: Climber. Habitat: Lowland and upland rainforest, 80–207 m. Vouchers: Ngumbau V & Mwadime N V065 (EA, HIB), Verdcourt B 2121, Magogo FC & Estes R 1211 (EA).

***Pilogyne scabra* (L. f.) W. J. de Wilde & Duyfjes** Habit: Climber. Habitat: Damp places, 80–272 m. Vouchers: Ngumbau V & Mwadime N V106 (EA, HIB), Brenan JPM & Brenan JH 14642 (EA).

***Trochomeria macrocarpa* (Sond.) Harv.** Habit: Climber. Habitat: Deciduous woodland on sandy soils, ca. 120 m. Voucher: SAJIT–005489 (EA, HIB).

***Zehneria monocarpa* G.W. Hu, V.M. Ngumbau & Q.F. Wang, sp. nov** Habit: Climber. Habitat: Bushland, ca. 199 m. Vouchers: SAJIT 007172 & 007173, Ngumbau V & Mwadime N V0399 (EA, HIB), Mwadime 2404 (EA): Endemic.

***Zehneria pallidinervia* (Harms) C. Jeffrey** Habit: Climber. Habitat: Deciduous bushland, ca. 0–88 m. Vouchers: SAJIT–006443 & 006241 (EA, HIB), Beentje HJ 2345 (EA).

***Zehneria peneyana* (Naudin) Schweinf. & Asch.** Habit: Climber. Habitat: Swampy and marshy areas, 20–425 m. Vouchers: Ngumbau V & Mwadime N V0555 (EA, HIB), Festo L & Luke Q 2453 (EA).

***Zehneria thwaitesii* (Schweinf.) C. Jeffrey** Habit: Climber. Habitat: Lowland grassland, margins of rivers, 30–223 m. Vouchers: Ngumbau V & Mwadime N V0540 & 0124 (EA, HIB), Robertson SA 5854 (EA).

**F69. Cymodoceaceae**

2 Genera, 2 Species

***Thalassodendron ciliatum* (Forssk.) Hartog** Habit: Herb. Habitat: Shallow waters, 0–33 m. Voucher: SAJIT–006253 (EA, HIB).

***Cymodocea rotundata* Ehrenb. & Hempr. ex Asch.** Habit: Herb. Habitat: Clear water, 0–10 m. Vouchers: SAJIT–006255 & 006266 (EA, HIB).

**F70. Cyperaceae**

18 Genera, 111 Species

***Alinula paradoxa* (Cherm.) Goetgh. & Vorster** Habit: Herb. Habitat: In and near rice fields, 0–30 m. Voucher: Coppejans 5690 (EA).

***Bolboschoenus maritimus* (L.) Palla** Habit: Herb. Habitat: In and near swamps, 0–30 m. Vouchers: SAJIT–005912 (EA, HIB), Luke Q 5630 (EA).

***Bulbostylis abortiva* (Steud.) C.B. Clarke** Habit: Herb. Habitat: Shallow soil over rock, grassland, ca. 50 m. Voucher: Kaesnner 346 (EA).

***Bulbostylis afroorientalis* (Lye) R.W. Haines** Habit: Herb. Habitat: Grassland with scattered trees, 80–300 m. Vouchers: Luke & Luke 4387, Lye & Katende 6288, Faden & Evans 71/631 (EA).

***Bulbostylis argenteobrunnea* C.B. Clarke** Habit: Herb. Habitat: Mixed grasslands and cleared bushland, ca. 150 m. Vouchers: Bogdan 5339, Magogo FC & Esters R 1177 (EA).

***Bulbostylis barbata* (Rottb.) C.B. Clarke** Habit: Herb. Habitat: Mixed woodland and bushland, ca. 0–120 m. Vouchers: Polhill & Paulo 847, Kirika P, Muthoka P & Mbale M 753 (EA).

**Bulbostylis boeckeleriana (Schweinf.) Beetle var. boeckeleriana** Habit: Herb. Habitat: Dry grassland, ca. 0–500 m. Vouchers: Luke WRQ & Kabuye CHS 5120, Luke WRQ & Robertson SA 5120 (EA).

**Bulbostylis boeckeleriana var. transiens (K. Schum.) R.W. Haines** Habit: Herb. Habitat: Grassland and open bushland, 100–400 m. Vouchers: Moomaw 931, Robertson 5120 (EA).

***Bulbostylis burchellii* (Ficalho & Hiern) C.B. Clarke** Habit: Herb. Habitat: Open coastal forests and marginal mangrove vegetation, 10–75 m. Vouchers: Rexton Karisa s.n., Karisa L 52 (EA).

***Bulbostylis contexta* (Nees) Bodard** Habit: Herb. Habitat: Rocky area, ca. 0–400 m. Vouchers: Gilbert MG & Kuchar P 5899, Magogo FC & Glover PE 356, Drummond & Hemsley1116 (EA).

***Bulbostylis densecaespitosa* (Lye) R. W. Haines** Habit: Herb. Habitat: Coastal forest, 50–300 m. Vouchers: Moormaid JC 931, Luke & Robertson 2199, Robertson et al. 7010 (EA): Endemic.

***Bulbostylis filamentosa* (Vahl) C. B. Clarke** Habit: Herb. Habitat: Seasonally wet habitats, grassland, and crevices in rock faces, 0–326 m. Vouchers: Magogo FC & Glover PE 23, Lye & Katende 6283, Magogo & Glover 231 (EA).

**Bulbostylis hispidula (Vahl) R. W. Haines subsp. hispidula** Habit: Herb. Habitat: Coastal wooded grassland, 0–100 m. Voucher: Mwachala G et al. 856 (EA).

**Bulbostylis hispidula subsp. filiformis (C. B. Clarke) R. W. Haines** Habit: Herb. Habitat: Grassland, bushland on sandy dunes, dry banks, roadsides, and wet rock crevices, ca. 0–50 m. Vouchers: Rawlins s.n., Robertson SA 3222, Kuchar P 7205 (EA).

**Bulbostylis hispidula subsp. halophila (Lye) R. W. Haines** Habit: Herb. Habitat: Short grassland, sandy beaches, and abandoned cultivation, 0–15 m. Vouchers: Moomaw JC 816, Cunningham-van Someren GR Sh107 (EA).

**Bulbostylis hispidula subsp. intermedia (Lye) R. W. Haines** Habit: Herb. Habitat: Dry grassland and shallow soil on rocks, near sea level. Voucher: Lye 6280 (EA): Endemic.

***Bulbostylis microelegans* (Lye) R. W. Haines** Habit: Herb. Habitat: Grassland and scrub, ca. 10 m. Voucher: Festo L & Luke WRQ 2729 (EA).

***Bulbostylis pilosa* (Willd.) Cherm.** Habit: Herb. Habitat: Seasonally flooded wooded grassland, mangrove swamps, and bushland, 1–400 m. Vouchers: Ngumbau V & Mwadime N V0150 (EA, HIB), Drummond RB & Hemsley JH 1174 (EA).

***Courtoisina assimilis* (Steud.) Maquet** Habit: Herb. Habitat: Streamside, ditches, and seasonal pools, ca. 500 m. Voucher: Faden RB & Faden AJ 116 (EA).

***Cyperus afrodunensis* Lye** Habit: Herb. Habitat: On sand dunes and sandy soil near seashore. Voucher: Bogdan A 2537 (EA).

***Cyperus alopecuroides* Rottb.** Habit: Herb. Habitat: In swamps and seasonally wet grasslands, ca. 20 m. Voucher: Rawlins SP 633 (EA).

***Cyperus amabilis* Vahl** Habit: Herb. Habitat: In grassland and wooded grassland, ca. 0–15 m. Vouchers: Rawlins SP 88, Magogo FC & Glover PE 1024, RB & AJ Faden 71 (EA).

***Cyperus articulatus* L.** Habit: Herb. Habitat: In swamps, lakeshores, wet grasslands, and pools, ca. 0–190 m. Vouchers: Sangai GW 15839, Luke PA & WRQ 4087 (EA).

***Cyperus blysmoides* Hochst. ex C. B. Clarke** Habit: Herb. Habitat: Seasonally wet habitats, flooded grasslands, and, swampy areas, ca. 30 m. Voucher: Kabuye et al. TPR 449 (EA).

***Cyperus boreobellus* Lye** Habit: Herb. Habitat: On damp shallow sandy soil and rocky pools, 350–400 m. Vouchers: Drummond & Hemsley 4153, Luke 5582A (EA): Endemic.

***Cyperus chordorrhizus* Chiov.** Habit: Herb. Habitat: On sand dunes, sea level. Vouchers: Luke Q 5646, Frazier J 896 (EA).

***Cyperus colymbetes* Kotschy & Peyr.** Habit: Herb. Habitat: Muddy areas, on swampy ground, 10–400 m. Vouchers: Kabuye et al. TPR 382, Luke PA & WRQ 4388 (EA).

***Cyperus compressus* L.** Habit: Herb. Habitat: In roadside ditches, drainage trenches, permanent, and seasonal pools, ca. 0–300 m. Vouchers: Gilbert MG & Kuchar 5898, Magogo FC & Glover PE 1064 (EA).

***Cyperus crassipes* Vahl** Habit: Herb. Habitat: On shores and sand dunes, 0–60 m. Vouchers: SAJIT–006263 (EA, HIB), Greenway PJ & Rawlins SP 9293, Greenway PG 11336 (EA).

***Cyperus cuspidatus* Kunth** Habit: Herb. Habitat: Grassland, ca. 0–30 m. Vouchers: Langridge WP 139, Greenway PJ 10840 (EA).

***Cyperus denudatus* L. f.** Habit: Herb. Habitat: Riversides, floodplains, swamps, damp grassland, and moist rock crevices, ca. 0–150 m. Voucher: Magogo FC & Glover PE 339 (EA).

***Cyperus difformis* L.** Habit: Herb. Habitat: In swamps, alongside water edge, 0–198 m. Voucher: Magogo FC & Glover PE 556 (EA).

***Cyperus diurensis* Boeckeler** Habit: Herb. Habitat: In grassland, woodland, and rocky outcrops, 15–200 m. Vouchers: Greenway PJ & Rawlins SP 8956, Adams 64, L Festo & WRQ Luke 2492 (EA).

**Cyperus dubius Rottb. subsp. dubius** Habit: Herb. Habitat: Near the sea on sandy beaches, 0–300 m. Vouchers: Robertson SA 7299, Robertson SA et al. 270 (EA).

**Cyperus dubius var. macrocephalus (C.B. Clarke) Kük.** Habit: Herb. Habitat: Riverine, dry bushland, and grassland, 50–380 m. Vouchers: Kabuye CHS, Gilbert VC & Robertson A 84/84A, Magogo FC & Glover PE 307A, Luke 4004 (EA).

***Cyperus exaltatus* Retz.** Habit: Herb. Habitat: Grasslands and pond margins, ca. 0–30 m. Vouchers: Kuchar P 13556, Luke WRQ et al. 5718, Luke WRQ 3464 (EA).

***Cyperus foliaceus* C.B. Clarke** Habit: Herb. Habitat: Woodlands, seasonally wet habitats, swamps, along streams, and pools, 0–198 m. Vouchers: Magogo FC & Glover PE 103 & 574 (EA).

***Cyperus frerei* C.B. Clarke** Habit: Herb. Habitat: Sand dunes, sandy mangrove shores, 0–10 m. Voucher: Mwadime N & Luke WRQ 2612 (EA).

***Cyperus grandis* C.B. Clarke** Habit: Herb. Habitat: Swamps, in stagnant or moving water, 0–400 m. Vouchers: Harper GB 113/55, Drummond RB & Hemsley JH 1092 (EA).

***Cyperus haspan* L.** Habit: Herb. Habitat: Swampy or marshy sites, seasonally wet grassland, 0–426 m. Vouchers: Mwachala G et al. 857, Gilbert MG & Kuchar P. 5894, Sangai GW 15794 (EA).

***Cyperus hemisphaericus* Boeckeler** Habit: Herb. Habitat: Open grassland, *Brachystegia* woodland, and wooded grassland, 0–140 m. Vouchers: Mamur JC 981, Luke WRQ 3199 (EA).

***Cyperus holstii* Kük.** Habit: Herb. Habitat: Seasonally wet grassland, alongside pools, and swamps, 0–250 m. Vouchers: Verdcourt 3959, Luke & Grey 4027, Luke 4605 (EA).

***Cyperus involucratus* Rottb.** Habit: Herb. Habitat: Swampy grassland and lake shores, 30 m. Vouchers: Brathany 1982 Expedition 49 (EA).

***Cyperus kaessneri* C.B. Clarke** Habit: Herb. Habitat: Seasonally damp habitats and bushland, 0–400 m. Vouchers: Drummond & Hemsley 3745, Adams BR 7 (EA).

***Cyperus kwaleensis* Lye** Habit: Herb. Habitat: On shallow sandy soils over outcropping rocks, ca. 360 m. Voucher: Drummond & Hemsley 4204 (EA): Endemic.

***Cyperus laevigatus* L.** Habit: Herb. Habitat: Damp sandy places, ca. 45 m. Vouchers: Festo L & Luke WRQ 2439, Makin J 417 (EA).

***Cyperus latifolius* Poir.** Habit: Herb. Habitat: In swamps, marshes, boggy grasslands, and roadside, 0–195 m. Voucher: Magogo FC & Glover PE 576 (EA).

**Cyperus laxus subsp. buchholzii (Boeckeler) Lye** Habit: Herb. Habitat: Forest, secondary vegetation, on stream banks, 100 m. Vouchers: Greenway & Rawlins 9364, Robertson & Luke 5951 (EA).

***Cyperus ligularis* L.** Habit: Herb. Habitat: Swamps, marshes, and creek margins. Voucher: Vera de Measter 209 (EA).

**Cyperus longus var. pallidus Boeckeler** Habit: Herb. Habitat: Lake edges, swamps, and temporary pools, ca. 65 m. Voucher: Nattrass MS 1521 (EA).

***Cyperus luteus* Boeckeler** Habit: Herb. Habitat: Wet grassland and secondary forest, 0–228 m. Vouchers: Ngumbau V & Mwadime N V048 (EA, HIB), Musyoki & Hansen 996 (EA).

***Cyperus meeboldii* Kük.** Habit: Herb. Habitat: Wet habitats or in mud, 30–353 m. Vouchers: Luke & Gray 4050, Mwachala G et al 808 (EA).

***Cyperus microumbellatus* Lye** Habit: Herb. Habitat: In swampy area, ca. 380 m. Voucher: Magogo FC & Glover PE 323 (EA): Endemic.

**Cyperus michelianus subsp. pygmaeus (Rottb.) Asch. & Graebn.** Habit: Herb. Habitat: Seasonally wet habitats, damp sandy, ca. 30 m. Voucher: Faden & Faden 72/139 (EA).

***Cyperus mollipes* (C. B. Clarke) K. Schum.** Habit: Herb. Habitat: Grassland, ca. 0–20 m. Vouchers: Napper D 2179, Magogo FC & Glover PE 316, Waayenberg H 17, Kabuye et al. TPR 249, Luke WRQ & Robertson SA 2195 (EA).

**Cyperus niveus var. leucocephalus (Kunth) Fosberg** Habit: Herb. Habitat: Dry grassland, 0–200 m. Vouchers: Mwadime N 2652, Ross K 164, Drummond & Hemsley 4171 (EA).

***Cyperus oblongoincrassatus* Kük.** Habit: Herb. Habitat: Rocky sites, dry *Acacia*-*Commiphora* bushland, ca. 350 m. Voucher: Drummond & Hemsley 4097 (EA).

***Cyperus pectinatus* Vahl** Habit: Herb. Habitat: Swamps, lake margins, and shallow water, ca. 15 m. Vouchers: SAJIT–006223 (EA, HIB), Mwadime N 2662 (EA).

***Cyperus prolifer* Lam.** Habit: Herb. Habitat: Swamp edges, streamside, and seasonally flooded grasslands, 0–450 m. Vouchers: Ngumbau V & Mwadime N V0255 (EA, HIB), Magogo FC & Glover PE 119 (EA).

**Cyperus renschii Boeckeler var. renschii** Habit: Herb. Habitat: Forests, forest swamps, along forest streams, or roadside, (0–) 150 m. Vouchers: Ngumbau V & Mwadime N V110 (EA, HIB) Magogo FC & Glover PE 135, Moomaw 1063 (EA).

**Cyperus rotundus L. subsp. rotundus** Habit: Herb. Habitat: Roadsides, sandy fields, and cultivated ground, ca. 260 m. Voucher: Waaijenberg H 6 (EA).

**Cyperus rotundus subsp. tuberosus (Rottb.) Kük.** Habit: Herb. Habitat: Roadsides, sandy fields, and cultivated ground, 140–300 m. Vouchers: Robertson SA et al. 342, Faden RB & Evans A 627 (EA).

***Cyperus rubicundus* Vahl** Habit: Herb. Habitat: Seasonally wet habitats, 0–353 m. Vouchers: Bryan R Adames 54, Mwachala G et al. 814 (EA).

***Cyperus squarrosus* L.** Habit: Herb. Habitat: Grassland and roadside, ca. 200 m. Vouchers: Festo L & Luke WRQ 2474, Luke et al. 6325 (EA).

***Cyperus tenax* Boeckeler** Habit: Herb. Habitat: Seasonally flooded grassland, swampy grassland, mangrove edge, and woodland, 0–297 m. Vouchers: Ngumbau V & Mwadime N V0151 (EA, HIB), Luke PA & WRQ 5676B (EA).

***Cyperus zanzibarensis* C. B. Clarke** Habit: Herb. Habitat: No data. Voucher: Taylor s.n. (EA).

***Cyperus zollingeri* Steud.** Habit: Herb. Habitat: In seasonally wet habitats, 0–650 m. Vouchers: Ngumbau V & Mwadime N V0537 (EA, HIB), Gillespie 155, Faden et al. 71/645, Luke et al. 5912 (EA).

***Diplacrum africanum* C. B. Clarke** Habit: Herb. Habitat: On bare sand or mud in marshy grassland, ca. 60–400 m. Voucher: Luke & Luke 5963 (EA).

***Eleocharis atropurpurea* (Retz.) J. Presl & C. Presl** Habit: Herb. Habitat: Seasonal pools, seepage areas, and rice paddies ca. 5 m. Voucher: Mwadime N 2568 (EA).

***Eleocharis dulcis* (Burm.f.) Trin. ex Hensch.** Habit: Herb. Habitat: Swamps and shallow parts of lakes, 0–100 m. Voucher: Kirika P, Mbale M & Mbatha M 778 (EA).

***Eleocharis geniculata* (L.) Roem. & Schult.** Habit: Herb. Habitat: Lake shores and swamps, 0–150 m. Voucher: Luke PA & WRQ 5640 (EA).

***Fimbristylis bisumbellata* (Forssk.) Bubani** Habit: Herb. Habitat: Mud flats and sandy riverbanks, 0–150 m. Voucher: Sangai GW 15848 (EA).

***Fimbristylis bivalvis* (Lam.) Lye** Habit: Herb. Habitat: Streams, roadsides, and bushland, 1–100 m. Voucher: Taylor s.n. (EA).

***Fimbristylis cymosa* R.Br.** Habit: Herb. Habitat: Sandy foreshores, edges of mangrove swamps, coral rock, and saline marshes, 0–45 m. Vouchers: Robertson SA & Abuodha J 6916, Polhill & Paulo 897 (EA).

***Fimbristylis dichotoma* (L.) Vahl** Habit: Herb. Habitat: Margins of rice fields and swamps, ca. 0–150 m. Vouchers: Luke WRQ et al. 6186, Drummond & Hemsley 1164 (EA).

***Fimbristylis ferruginea* (L.) Vahl** Habit: Herb. Habitat: Sandy soils by the coast, 0–50 m. Vouchers: Kuchar P 10052, Smith, Beentje & Muasya 244 (EA).

***Fimbristylis littoralis* Gaudich.** Habit: Herb. Habitat: Dry river beds and drying swamp, ca. 0–300 m. Vouchers: Luke WRQ et al. 5712, Polhill & Paulo 897, Makin 418 (EA).

***Fimbristylis pilosa* Vahl** Habit: Herb. Habitat: Seasonally flooded grassland and bushed grasslands, ca. 30 m. Vouchers: Luke et al. 6317, Gillespie 277 & 280 (EA).

***Fimbristylis polytrichoides* (Retz.) Vahl** Habit: Herb. Habitat: Seasonally inundated mangrove swamp, 0–15 m. Vouchers: Rawlins SP 12080, Bogdan 4708, Polhill & Paulo 896 (EA).

**Fimbristylis quinquangularis subsp. macroglumis (Lye) Verdc.** Habit: Herb. Habitat: Marshy grassland, 65 m. Voucher: Luke WRQ 17419 (EA).

***Fimbristylis ovata* (Burm.f.) J. Kern** Habit: Herb. Habitat: Plain grassland, seasonally wooded grassland, and grazed fallow areas, 0–381 m. Vouchers: Magogo & Glover 384, Adams BR 13 (EA).

***Fimbristylis triflora* (L.) K. Schum. ex Engl.** Habit: Herb. Habitat: Tidal mud, wooded grassland, and mangrove swamps, 0–45 m. Vouchers: Kirika P & Muthoka P 734, Luke & Mbinda 5880, Polhill & Paulo 900 (EA).

***Fuirena ciliaris* (L.) Roxb.** Habit: Herb. Habitat: Coastal streams, pools, seasonal swamp edges, wet grassland, and weed in rice fields, 0–300 (–500) m. Voucher: Gilbert MG & Kuchar P 5897 (EA).

***Fuirena claviseta* Peter** Habit: Herb. Habitat: Swamp, river, stream banks, and drainage ditches, 0–300 (–500) m. Voucher: Luke WRQ & PA 4385 (EA).

***Fuirena ochreata* Nees ex Kunth** Habit: Herb. Habitat: Seasonally wet grassland, edge of permanent swamp and stream, 0–450 m. Vouchers: SAJIT–005487 (EA, HIB), Magogo FC & Glover PE 163 (EA).

***Fuirena umbellata* Rottb.** Habit: Herb. Habitat: Seasonally wet grassland, swamp forest, stream, and lake banks, ca. 0–213 m. Vouchers: Luke WRQ & PA 6027, Magogo & Glover 736 (EA).

***Kyllinga cartilaginea* K. Schum.** Habit: Herb. Habitat: Lowland forest, coconut grooves, beach crest, dunes, and *Brachystegia* woodland, 0–50 (–200) m. Vouchers: Ngumbau V & Mwadime N V097 (EA, HIB), Townsend CC & Hooper SS 1212 (EA).

***Kyllinga crassipes* Boeckeler** Habit: Herb. Habitat: Seasonally wet grassland, old cultivations, and woodlands, 0–200 m. Voucher: Bogdan 4703 (EA).

***Kyllinga erecta* Schumach.** Habit: Herb. Habitat: Wet depressions, swamps, lake, pool, and dam fringes, 0–353 m. Vouchers: Mwachala G et al. 809, Luke WRQ et al. 5716, Gilbert & Kuchar 5896 (EA).

***Kyllinga microstyla* C.B. Clarke** Habit: Herb. Habitat: Bushland or scattered tree grassland, ca. 300 m. Voucher: Faden & Evans 71/628 (EA).

**Kyllinga nervosa var. flava (C.B. Clarke) Lye** Habit: Herb. Habitat: Bushland, 50–250 m. Voucher: Wamukoya 80 (EA).

**Kyllinga polyphylla Willd. ex Kunth var. polyphylla** Habit: Herb. Habitat: Moist grassland, ca. 426 m. Vouchers: Kuchar P 12875, Magogo FC & Glover PE 108 (EA).

**Kyllinga polyphylla var. elatior (Kunth) Kük.** Habit: Herb. Habitat: Grassland, 60 m. Voucher: Adams BR 19 (EA).

***Mariscus longibracteatus* Cherm.** Habit: Herb. Habitat: Streamside, permanently or seasonally swampy, and moist sites in shade, ca. 10 m. Vouchers: Luke WRQ et al. 5628, Mwachala G et al. 852, Festo 2424 (EA).

***Mariscus maderaspatanus* (Willd.) Napper** Habit: Herb. Habitat: Grassland or roadsides, 0–200 m. Vouchers: Luke & Mbinda 5973A, Jeffrey 14, Luke, Mbinda & Madudu 6375 (EA).

***Mariscus psilostachys* C.B. Clarke** Habit: Herb. Habitat: Dry grassland and rocky outcrops, 0–100 m. Voucher: Luke WRQ & PA 4505 (EA).

***Mariscus sumatrensis* (Retz.) J. Raynal** Habit: Herb. Habitat: Forest clearings, path sides, grassland, and woodland, 0–381 m. Vouchers: Magogo FC & Glover PE 351, Magogo FC & Estes R 1218 (EA).

***Oxycaryum cubense* (Poepp. & Kunth) Palla** Habit: Herb. Habitat: Floating in lakes, swamps, and pools, ca. 0–300 m. Voucher: Kabuye et al. TPRs 531 (EA).

***Pycreus hildebrandtii* C.B. Clarke** Habit: Herb. Habitat: Floating in lakes, swamps, and pools, 0–800 m. Voucher: Festo L & Luke WRQ 2608 (EA).

***Pycreus macrostachyos* (Lam.) J. Raynal** Habit: Herb. Habitat: Lakes, ponds, and at river edges. Vouchers: Luke Q 5631, Luke WRQ & PA 5728 (EA).

***Pycreus pelophilus* (Ridl.) C.B. Clarke** Habit: Herb. Habitat: Near water, ca. 15 m. Voucher: Festo L & Luke WRQ 2606 (EA).

**Pycreus polystachyos (Rottb.) P. Beauv. subsp. polystachyos** Habit: Herb. Habitat: Margins of rivers, riverbanks, lake margins, and sandy beaches, 0–200 m. Vouchers: Luke WRQ et al. 5719, Drummond RB & Hemsley JH 1163 (EA).

**Pycreus polystachyos var. laxiflorus** (**Benth.) C.B. Clarke** Habit: Herb. Habitat: Margins of rivers, and sandy beaches, ca. 55 m. Vouchers: Luke WRQ 17417, Mwadime N & Chesire C 168 (EA).

***Pycreus pumilus* (L.) Nees** Habit: Herb. Habitat: Along swampy and streamside, ca. 50 m. Vouchers: Kirika P & Muthoka P 736, Luke et al. 6324, Gillet & Kuchar 5900 (EA).

***Queenslandiella hyalina* (Vahl) Ballard** Habit: Herb. Habitat: Grassland and bushland on coral rag, 0–30 m. Vouchers: Schlieben 12140, Gillespie 14 (EA).

***Remirea maritima* Aubl.** Habit: Herb. Habitat: Sandy seashores, just above the high tide level. Voucher: Robertson 3675 (EA).

***Rhynchospora perrieri* Cherm.** Habit: Herb. Habitat: Swamp areas, roadside ditches, alongside streams, and in damp places, ca. 300 m. Voucher: Bogdan A 5347 (EA).

***Schoenoplectiella articulata* (L.) Lye** Habit: Herb. Habitat: Grassland, wooded grassland, and *Acacia* bushland zone, 0–300 m. Vouchers: Verdcourt 3958, Kuchar P 12876, Kirika P, Muthoka P & Mbale M 743 (EA).

***Schoenoplectiella juncea* (Willd.) Lye** Habit: Herb. Habitat: Seasonal pools, swamps, along drainage lines, and stagnant water, 0–600 m. Voucher: Gillespie 282 (EA).

***Schoenoplectiella lateriflora* (J.F. Gmel.) Lye** Habit: Herb. Habitat: Swamps, pond, lake shores, and seasonal pools, ca. 0–300 m. Vouchers: Gillespie 283, Sangai in EA 15793 (EA).

***Schoenoplectus scirpoides* (Schrad.) Browning** Habit: Herb. Habitat: Lakes, riverine fringes, and coastal saltmarsh, 0–200 m. Voucher: Luke 5642 (EA).

***Scleria achtenii* De Wild.** Habit: Herb. Habitat: Occasionally in permanent water, 0–60 m. Voucher: Luke & Luke 5994 (EA).

***Scleria bambariensis* Cherm.** Habit: Herb. Habitat: Swamps and seasonally wet grassland, ca. 60 m. Vouchers: Bogdan A 3344, Luke & Luke 5993 (EA).

***Scleria boivinii* Steud.** Habit: Herb. Habitat: Swampy forest and lowland forest, ca. 60 m. Voucher: Luke et al. 5920 (EA).

***Scleria foliosa* Hochst. ex A. Rich.** Habit: Herb. Habitat: Swamp edges, seasonally damp areas, and rice fields, ca. 30 m. Voucher: Luke & Gray 4058 (EA).

***Scleria hildebrandtii* Boeckeler** Habit: Herb. Habitat: Grassland and lowland forest, 30–215 m. Vouchers: Luke & Luke 5964, Hildebrandt 2044 (EA).

***Scleria lagoensis* Boeckeler** Habit: Herb. Habitat: Grassland, *Terminalia*-*Combretum* and *Brachystegia*-*Uapaca* woodland, and forest edge grassland, ca. 750 m. Voucher: Luke & Luke 5962 (EA).

***Scleria lithosperma* (L.) Sw.** Habit: Herb. Habitat: Evergreen forest, forest, and plantation edges, 20–200 m. Vouchers: Ngumbau V & Mwadime N V087 (EA, HIB), Magogo FC & Glover PE 98 (EA).

***Scleria parvula* Steud.** Habit: Herb. Habitat: Seasonally flooded grassland and swampy stream banks, ca. 60 m. Vouchers: Bogdan 3344, Luke WRQ & PA 4078 & 5993 (EA).

***Scleria racemosa* Poir.** Habit: Herb. Habitat: Swampy ground in forest, lake shores, and swamps, muddy valley bottoms, ca. 0–28 m. Vouchers: SAJIT–006217 (EA, HIB), Luke WRQ 3607, Magogo & Glover 539, Kirika P 949 (EA).

**F71. Dichapetalaceae**

2 Genera, 9 Species

***Dichapetalum arenarium* Breteler** Habit: Liana. Habitat: Coastal forest and bushland, 0–450 m. Vouchers: Ngumbau V & Mwadime N V0361 (EA, HIB), Luke WRQ & Robertson SA 501 (EA).

***Dichapetalum fadenii* Breteler** Habit: Shrub. Habitat: Forest on limestone outcrops, 200–220 m. Voucher: Faden et al. 77/416 (EA): Endemic.

***Dichapetalum fructuosum* Hiern** Habit: Liana. Habitat: Coastal forest, ca. 200 m. Vouchers: Luke WRQ & Robertson SA 2707, Magogo & Glover 179 (EA).

**Dichapetalum madagascariense Poir. var. madagascariense** Habit: Liana. Habitat: Evergreen forest, 200 m. Vouchers: Magogo FC & Glover PE 250, Greenway 9652, Verdcourt 1899 (EA).

***Dichapetalum mossambicense* (Klotzsch) Engl.** Habit: Liana. Habitat: Coastal forest, forest edges, coastal, and secondary bushland, 0–400 m. Vouchers: SAJIT–006028, Ngumbau V & Mwadime N V0334 (EA, HIB), Luke WRQ & Robertson SA 522 (EA).

***Dichapetalum ruhlandii* Engl.** Habit: Liana. Habitat: Forest and associated bushland, 50–382 m. Vouchers: SAJIT–006079 (EA, HIB), Luke WRQ 871, Verdcourt 1880 (EA).

***Dichapetalum* sp. ?nov.** Habit: Shrub. Habitat: Moist forest, ca. 231 m. Voucher: Ngumbau V & Mwadime N V113 (EA, HIB): Endemic.

***Dichapetalum zenkeri* Engl.** Habit: Shrub. Habitat: Lowland mostly evergreen forest, 30–700 m. Vouchers: Ngumbau V & Mwadime N V0428 & 0529 (EA, HIB), Graham MD 2132, Kimeu JM 691 (EA).

***Tapura fischeri* Engl.** Habit: Tree. Habitat: Forest or sometimes riverine, ca. 220 m. Vouchers: Luke WRQ 873, Gillett & Kibuwa, Faden RB & Evans A 71/730 (EA).

**F72. Didiereaceae**

1 Genus, 1 Species

***Calyptrotheca taitensis* (Pax & Vatke) Brenan** Habit: Shrub. Habitat: Deciduous bushland, open woodland, and grassland, ca. 30 m. Voucher: Robertson 6600 (EA).

**F73. Dilleniaceae**

1 Genus, 2 Species

***Tetracera boiviniana* Baill.** Habit: Shrub. Habitat: Coastal woodland and thicket, 50–350 m. Vouchers: SAJIT–005464 & 005490 (EA, HIB), Robertson SA & Luke Q 5712 (EA).

***Tetracera litoralis* Gilg** Habit: Shrub. Habitat: Lowland rainforest and dry evergreen forest, 0–50 (–500) m. Vouchers: SAJIT–005515 & 005977, Ngumbau V & Mwadime N V0367 & V045 (EA, HIB), Simpson BL 375 (EA).

**F74. Dioscoreaceae**

2 Genera, 7 Species

***Dioscorea asteriscus* Burkill** Habit: Climber. Habitat: Rainforest, lowland dry evergreen forest, and coastal evergreen bushland, 0–160 m. Vouchers: SAJIT–006003 & 005947 (EA, HIB), Kabuye CHS 84, Kirika P, Nyamongo D & Sanyanyi S 39664, Luke WRQ 2878 (EA).

***Dioscorea buchananii* Benth.** Habit: Climber. Habitat: Riverine forest and near mangrove swamps, ca. 300 m. Vouchers: Mutanga JG & Kamau P 22, Kuchar P 12003 (EA).

***Dioscorea dumetorum* (Kunth) Pax** Habit: Climber. Habitat: Edges of lowland rainforest, dry evergreen forest, and evergreen bushlands, 0–650 m. Vouchers: SAJIT–006015 & 005524, Ngumbau V & Mwadime N V079 & 0252 (EA, HIB), Katz SS 32, Magogo FC & Glover PE 1062 (EA).

**Dioscorea hirtiflora subsp. orientalis Milne-Redh.** Habit: Climber. Habitat: Dry evergreen forest, thickets, and rocky places, 0–570 m. Vouchers: Jeffery GW 276, Robertson SA & Luke WRQ 5158 (EA).

**Dioscorea quartiniana A. Rich. var. quartiniana** Habit: Climber. Habitat: Lowland rainforest, riverine forest, and forest edges, ca. (50–) 300 m. Vouchers: Mwadime N 26, Robertson SA 4934 (EA).

***Dioscorea sansibarensis* Pax** Habit: Climber. Habitat: Lowland rainforest, riverine forest, and coastal evergreen bushland, ca. 0–300 m. Vouchers: SAJIT–005927 (EA, HIB), Brenan JPM & JB Gillet 14644, Gillett JB 18713 (EA).

***Tacca leontopetaloides* (L.) Kuntze** Habit: Herb. Habitat: Grassland, bushland or woodland, ca. 0–227 m. Vouchers: SAJIT–005375, Ngumbau V & Mwadime N V0281 (EA, HIB), Magogo FC & Glover PE 484, Napier 6294, RM Graham 2120 (EA).

**F75. Droseraceae**

1 Genus, 1 Species

***Drosera indica* L.** Habit: Herb. Habitat: Wet mud in swamps, pools, and streams, ca. 30 m. Voucher: Festo L, Luke Q & P 2728 (EA).

**F76. Ebenaceae**

2 Genera, 15 Species

**Diospyros abyssinica (Hiern) F. White subsp. abyssinica** Habit: Tree. Habitat: Rainforest and evergreen forest, 0–385 m. Vouchers: Luke WRQ 1855, Muasya JM 708 (EA).

***Diospyros amaniensis* Gürke** Habit: Tree. Habitat: Rainforest or ground water forest, 250–500 m. Vouchers: Luke WRQ & Kahumbu P 4546G, Magogo & Glover 82, White 11191 (EA).

***Diospyros bussei* Gürke** Habit: Tree. Habitat: Wet bushland or wooded grassland, 0–600 m. Vouchers: Dale IR K3539, Drummond & Hemsley 4183 (EA).

***Diospyros consolatae* Chiov**. Habit: Tree. Habitat: Semi-evergreen or evergreen dry forest, thicket, and woodland, 0–300 m. Vouchers: Dale IR K3768, Greenway 9815 (EA).

***Diospyros greenwayi* F. White** Habit: Tree. Habitat: Semi-deciduous and evergreen forest, 0–400 m. Vouchers: SAJIT–005571 & 006062, Ngumbau V & Mwadime N V0233 (EA, HIB), Mohamed HB 3860, Luke WRQ & Robertson SA 219 (EA): Vulnerable.

***Diospyros kabuyeana* F. White** Habit: Tree. Habitat: Evergreen, semi-deciduous lowland forest, and sometimes riverine, 20–700 m. Vouchers: Dale IR 3426, Luke WRQ & Robertson SA 525 (EA).

**Diospyros loureiroana subsp. rufescens (Caveney) Verdc.** Habit: Tree. Habitat: Evergreen bushland, 0–750 m. Vouchers: SAJIT–005479 (EA, HIB), Luke WRQ & Robertson SA 2779, RM Graham 323 in FD 1821 (EA).

***Diospyros mespiliformis* Hochst. ex A.DC** Habit: Tree. Habitat: Moist semi-deciduous forest and riverine forest, ca. 0–250 m. Voucher: Luke WRQ & Robertson SA 535 (EA).

**Diospyros natalensis (Harv.) Brenan subsp. natalensis** Habit: Tree. Habitat: Moist semi-deciduous forest, dry evergreen forest, and thicket, ca. 0–250 m. Vouchers: Luke Q 1568, Drummond RB & Hemsley JH 4018, Faden 77/690 (EA).

***Diospyros occulta* F. White** Habit: Tree. Habitat: Evergreen forest, 0–300 m. Vouchers: Luke WRQ 1849, Luke & Robertson 1700 (EA).

***Diospyros shimbaensis* F. White** Habit: Tree. Habitat: Coastal evergreen, 0–450 m. Vouchers: SAJIT–005545 (EA, HIB), Luke WRQ 1850 (EA): Endangered.

***Diospyros squarrosa* Klotzsch** Habit: Tree. Habitat: Woodland and bushland, and hillsides, 0–100 m. Vouchers: SAJIT–005477 (EA, HIB), Luke Q 1423, Drummond RB & Hemsley JH 4018 (EA).

***Euclea divinorum* Hiern** Habit: Shrub. Habitat: Grassland, open bushland, and thicket, 0–200 m. Voucher: Robertson SA & Luke WRQ 74 (EA).

**Euclea natalensis subsp. obovata F. White** Habit: Shrub. Habitat: Grassland, evergreen, and deciduous thicket, 0–301 m. Vouchers: SAJIT–005971 (EA, HIB), Gardner HM 1417, Cunningham-van Someren (EA).

**Euclea racemosa subsp. schimperi (A. DC.) F. White** Habit: Shrub. Habitat: Grassland bushland, woodland, and riverine forest or scrub, ca. 2 m. Voucher: SAJIT–006112 (EA, HB).

**F77. Eriocaulaceae**

1 Genus, 1 Species

***Eriocaulon elegantulum* Engl.** Habit: Herb. Habitat: Seasonal ponds and ditches, ca. 0–50 m. Vouchers: Ngumbau V & Mwadime N V0549 (EA, HIB), Magogo FC & Glover PE 355 (EA).

**F78. Erythroxylaceae**

2 Genera, 3 Species

***Erythroxylum emarginatum* Thonn.** Habit: Shrub. Habitat: Evergreen forest, coastal forest, and transitional bushland, ca. 2 m. Voucher: Mwadime N & Chesire C 184 (EA).

***Erythroxylum platyclados* Bojer** Habit: Tree. Habitat: Evergreen bushland on coral, sand dunes, and wooded grassland, 12–180 m. Voucher: Luke Q 1418 (EA).

***Nectaropetalum kaessneri* Engl.** Habit: Tree. Habitat: Dry evergreen forest, thickets, and waste ground, 0–390 m. Voucher: Dale in FD 3874 (EA).

**F79. Euphorbiaceae**

26 Genera, 87 Species

***Acalypha bussei* Hutch.** Habit: Herb. Habitat: Coastal forest, 0–160 m. Vouchers: Drummond & Hemsley 3993, Gillett 18640, Verdcourt 1079 (EA).

***Acalypha ciliata* Forssk.** Habit: Herb. Habitat: Wooded grassland, deciduous, and coastal bushland, 0–150 m. Voucher: Gillespie 219 (EA).

***Acalypha echinus* Pax & K. Hoffm.** Habit: Shrub. Habitat: Riverine forest, 60–300 m. Vouchers: Festo L, Luke Q & P 2757, Pakia M et al. 988, Drummond & Hemsley 3340 (EA).

***Acalypha engleri* Pax** Habit: Shrub. Habitat: Forest undergrowth, edges, and associated bushland, (100–) 400–500 m. Vouchers: Ngumbau V & Mwadime N V018 (EA, HIB), Luke WRQ & Robertson SA 1556, Magogo FC & Glover PE 140 (EA).

**Acalypha fruticosa Forssk. var. fruticosa** Habit: Shrub. Habitat: Coastal deciduous bushland and thicket, ca. 0–272 m. Vouchers: Ngumbau V & Mwadime N V043 (EA, HIB), Kuchar P 10145 (EA).

***Acalypha indica* L.** Habit: Herb. Habitat: Rivers, base of outcrops, and ruderal, ca. 0–100 m. Vouchers: Gillespie 40, Faden RB & Faden AJ 74/1170 & 74/1019 (EA).

**Acalypha lanceolata var. glandulosa (Müll.Arg.) Radcl.-Sm.** Habit: Herb. Habitat: Grassland, 0–100 m. Vouchers: SAJIT–006179 (EA, HIB), Luke WRQ & PA 4510, Polhill & Paulo 537 (EA).

**Acalypha neptunica Müll.Arg. var. neptunica** Habit: Shrub. Habitat: Forest undergrowth, edges, and associated bushland, ca. 36 m. Vouchers: SAJIT–006178 & 005551 (EA, HIB), Luke WRQ & Robertson SA 2698 (EA).

**Acalypha neptunica var. pubescens (Pax) Hutch.** Habit: Shrub. Habitat: Thickets and dry forest understory, 0–150 m. Voucher: Rawlins 203 (EA).

***Acalypha ornata* Hochst. ex A. Rich.** Habit: Shrub. Habitat: Forest undergrowth, edges, wooded grassland, deciduous woodland, and thicket, 0–276 m. Vouchers: Ngumbau V & Mwadime N V044 & 0220 (EA, HIB), Magogo FC & Glover PE 485A (EA).

***Acalypha paniculata* Miq.** Habit: Shrub. Habitat: Forest, open places, edges, and riverine, ca. 100–280 m. Vouchers: SAJIT–006152, Ngumbau V & Mwadime N V115 (EA, HIB), Robertson SA & Luke WRQ 5159 (EA).

***Alchornea laxiflora* (Benth.) Pax & K. Hoffm.** Habit: Shrub. Habitat: Evergreen forest and riverine thickets near coast, ca. 10 m. Vouchers: SAJIT–005512 (EA, HIB), Luke Q 1566, Magogo FC & Glover PE 72 (EA).

***Argomuellera mjikendae* Q. Luke, ined.** Habit: Shrub. Habitat: Bushland, ca. 86 m. Vouchers: Ngumbau V & Mwadime N V0471 (EA, HIB), Luke WRQ et al. 5702 (EA).

***Caperonia fistulosa* Beille** Habit: Herb. Habitat: Swamps, flood plains, and other seasonally flooded areas, 0–360 m. Vouchers: SAJIT–006195 (EA, HIB), Luke WRQ & Robertson SA 2815 (EA).

***Cavacoa aurea* (Cavaco) J. Léonard** Habit: Tree. Habitat: Forest, ca. 100 m. Vouchers: Ngumbau V & Mwadime N V0382 (EA, HIB), Thomas Mwadime in Mrs Robertson SA 7790, Luke WRQ & Robertson SA 2697 (EA).

***Croton blanchetianus* Baill.** Habit: Shrub. Habitat: Forest. Voucher: Luke WRQ et al. 4333 (EA).

***Croton dichogamus* Pax** Habit: Shrub. Habitat: Dry forest, bushland, and thicket, ca. 253 m. Vouchers: SAJIT–005902, FOKP–10002 (EA, HIB).

***Croton kinondoensis* G.W. Hu, V.M. Ngumbau & Q.F. Wang, sp. nov.** Habit: Shrub. Habitat: Forest edge, ca. 5 m. Vouchers: SAJIT 007225, Ngumbau V & Mwadime N V0520 (EA, HIB), Malombe I, Mwadime N & Saidi KI 1647 (EA): Endemic.

***Croton megalocarpoides* Friis & M.G. Gilbert** Habit: Tree. Habitat: In semi-evergreen coastal forest, and bushland, 5–275 m. Vouchers: Robertson SA & Luke WRQ 5551, Muchiri 458 (EA, HIB).

***Croton menyharthii* Pax** Habit: Shrub. Habitat: Deciduous bushland and thicket, ca. 0–50 m. Vouchers: Drummond & Hemsley 4116, Gillespie 378 (EA).

***Croton pseudopulchellus* Pax** Habit: Shrub. Habitat: Dry evergreen forest, deciduous woodland, bushland, and thicket, ca. 0–59 m. Vouchers: Ngumbau V & Mwadime N V0440 (EA, HIB), Festo L & Luke Q 2596, Ross KS 133 (EA).

***Croton sylvaticus* Hochst.** Habit: Tree. Habitat: Secondary forest, forest edges, along rivers, and around lakes, ca. 60–410 m. Vouchers: Ngumbau V & Mwadime N V0300 & 0111 (EA, HIB), Luke WRQ & Robertson SA 515 (EA).

***Croton talaeporos* Radcl.–Sm.** Habit: Tree. Habitat: Coastal bushland and wooded grassland, 0–100 m. Vouchers: Muchiri J 565, J Adamson in Bally 5935 (EA).

**Dalechampia parvifolia Lam. var. parvifolia** Habit: Climber. Habitat: Coastal bushland, ca. 0–600 m. Vouchers: Magogo FC & Glover PE 864, Luke Q 5679, Luke Q 1462, Graham RM 1904, Drummond & Hemsley 4089, Gray M & Luke WRQ 270, Drummond & Hemsley 1054 (EA).

***Dalechampia trifoliata* Peter ex Verdc. & Greenway** Habit: Climber. Habitat: Deciduous and coastal bushland or woodland, 60–600 m. Vouchers: Drummond & Hemsley 4089, Field 3 (EA).

***Erythrococca kirkii* (Müll.Arg.) Prain** Habit: Shrub. Habitat: Forest edges, coastal bushland, and thicket, ca. 0–181 m. Vouchers: Ngumbau V & Mwadime N V0224 (EA, HIB), Magogo FC & Glover PE 219 (EA).

***Erythrococca pentagyna* Radcl.-Sm.** Habit: Shrub. Habitat: Moist coastal forest, 60–160 m. Vouchers: Ngumbau V & Mwadime N V0464 (EA, HIB), Luke WRQ 3136, Luke WRQ & Robertson SA 1911, Luke WRQ et al. 6191, Drummond & Hemsley 3944, Kokwaro 3931, Faden 77/524 (EA): Endemic.

***Erythrococca pubescens* Radcl.-Sm.** Habit: Shrub. Habitat: Forest, ca. 0–380 m. Vouchers: Ngumbau V & Mwadime N V0464, Drummond & Hemsley 4168 (EA).

***Erythrococca usambarica* Prain** Habit: Shrub. Habitat: Primary and secondary evergreen forest, ca. 300–400 m. Vouchers: Luke WRQ 872, Gillett JB & Robertson SA 23996 (EA).

***Euphorbia acalyphoides* Hochst. ex Boiss.** Habit: Herb. Habitat: Bushland, ca. 10 m. Voucher: Festo L, Luke Q & P 2625 (EA).

**Euphorbia breviarticulata Pax var. breviarticulata** Habit: Shrub. Habitat: *Acacia*-*Commiphora* bushland, ca. 60–365 m. Vouchers: Bally & Smith 14404, Kuchar P 7323 (EA).

***Euphorbia buruana* Pax** Habit: Herb. Habitat: Bushland, ca. 100 m. Voucher: M van Leeuwen 19 (EA).

**Euphorbia bussei var. kibwezensis (N.E.Br.) S. Carter** Habit: Tree. Habitat: Deciduous woodland. Voucher: Polhill R & Paulo S 816 (EA).

**Euphorbia candelabrum Trémaux ex Kotschy var. candelabrum** Habit: Tree. Habitat: Wooded grassland, ca. (15–) 450 m. Vouchers: Robertson SA, Luke WRQ & Awimbo J 5248, Luke WRQ 954 (EA).

**Euphorbia candelabrum var. bilocularis (N.E.Br.) S. Carter** Habit: Tree. Habitat: Open wooded grassland, ca. 550 m. Voucher: Bally E 212 (EA).

***Euphorbia crotonoides* Boiss.** Habit: Herb. Habitat: Open woodland or scattered bushland, ca. 186 m. Voucher: Ngumbau V & Mwadime N V0337 (EA, HIB).

***Euphorbia cryptospinosa* P.R.O. Bally** Habit: Herb. Habitat: Open dry deciduous bushland, ca. 230 m. Voucher: Luke WRQ & Robertson SA 2527 (EA).

**Euphorbia cuneata Vahl subsp. cuneata** Habit: Shrub. Habitat: Coastal forest and often cultivated as a hedge plant, 0–160 m. Vouchers: Tweedie 1024, Gillespie 62 (EA).

***Euphorbia fluminis* S. Carter** Habit: Shrub. Habitat: Dry deciduous woodland, ca. 15 m. Voucher: Gillett JB 19534 (EA): Endemic.

***Euphorbia furcata* N.E.Br.** Habit: Shrub. Habitat: *Acacia* bushland, ca. 300 m. Voucher: Bally 1326 (EA).

**Euphorbia gossypina Pax subsp. gossypina** Habit: Shrub. Habitat: In *Acacia*-*Commiphora* bushland, ca. 30 m. Vouchers: Drummond & Hemsley 4044, Faden RB & Faden A74/1054 (EA).

**Euphorbia heterochroma subsp. tsavoensis S. Carter** Habit: Shrub. Habitat: Deciduous bushland, ca. 550 m. Voucher: Luke WRQ 940 (EA).

***Euphorbia heterophylla* L.** Habit: Herb. Habitat: Disturbed localities, cultivated, and wasted land, ca. 500 m. Voucher: Magogo FC & Glover PE 984 (EA): Naturalized.

***Euphorbia hirta* L.** Habit: Herb. Habitat: Roadsides and waste places, ca. 20 m. Vouchers: Magogo FC & Glover PE 724, Polhill & Paulo 535 (EA).

***Euphorbia indica* Lam.** Habit: Herb. Habitat: Grassland and seasonally water-logged places, ca. 5 m. Vouchers: SAJIT–006099 (EA, HIB), Tweedie 3138 (EA).

***Euphorbia invenusta* (N.E.Br.) Bruyns** Habit: Herb. Habitat: Deciduous woodland, thicket, ca. 50 m. Vouchers: Robertson SA & Luke WRQ 4464, Greenway PJ & Rawlins SP 9457 (EA).

***Euphorbia kalisana* S. Carter** Habit: Herb. Habitat: *Acacia*-*Commiphora* bushland, ca. 100 m. Voucher: Faden 74/998 (EA).

***Euphorbia kaessneri* Pax** Habit: Shrub. Habitat: Grassland and bushland near the coast, 20–100 m. Vouchers: SAJIT–006441 (EA, HIB), Mungai GM 166/80 (EA).

***Euphorbia neovirgata* Bruyns** Habit: Herb. Habitat: Bushland, 50–200 m. Voucher: Bally 12196 (EA).

**Euphorbia nyikae Pax ex Engl. var. nyikae** Habit: Tree. Habitat: Deciduous woodland, 250–700 m. Vouchers: Bally 5781, Balslev 47 (EA).

**Euphorbia nyikae var. neovolkensii (Pax) S. Carter** Habit: Tree. Habitat: Sand dunes, seashores, and deciduous woodland, 2–250 m. Vouchers: Robertson SA & Luke WRQ 5377, Luke WRQ et al. 4331 (EA).

***Euphorbia pereskiifolia* Houllet ex Baill.** Habit: Shrub. Habitat: Bushland, ca. 136 m. Vouchers: SAJIT–005950 (EA, HIB), Robertson SA & Luke WRQ 5538 (EA).

***Euphorbia systyloides* Pax** Habit: Herb. Habitat: Bushland, ca. 95 m. Voucher: SAJIT–006132 (EA, HIB).

***Euphorbia tanaensis* P.R.O. Bally & S. Carter** Habit: Tree. Habitat: Semi-deciduous swampy forest, ca. 15 m. Voucher: Robertson SA 4327 (EA): Critically Endangered, Endemic.

***Euphorbia taruensis* S. Carter** Habit: Herb. Habitat: Leaf-litter amongst rocks beneath trees, 150–480 m. Voucher: Lauranos & Newton 12315 (EA): Endemic.

**Euphorbia tenuispinosa Gilli var. tenuispinosa** Habit: Shrub. Habitat: *Acacia*-*Commiphora* bushland and evergreen forest, 0–350 m. Vouchers: Sangai GW 15629, Drummond & Hemsley 4182 (EA).

***Euphorbia tirucalli* L.** Habit: Tree. Habitat: Woodland, ca. 50 m. Vouchers: Jeffery GW 701, Festo L, Luke Q & P 2643 (EA).

***Euphorbia pervittata* S. Carter** Habit: Shrub. Habitat: Open deciduous bushland, ca. 500 m. Voucher: Mungai et al. 367 (EA).

***Euphorbia wakefieldii* N.E.Br.** Habit: Tree. Habitat: Bushland and forest remnants, 50–275 m. Vouchers: Ngumbau V & Mwadime N V0416 (EA, HIB), Bally PRO s.n., Robertson SA & Luke WRQ 4536B (EA).

***Excoecaria bussei* (Pax) Pax** Habit: Tree. Habitat: Deciduous woodland, bushland, and wooded grassland, ca. 5 m. Voucher: Festo L & Luke Q 2553 (EA).

***Excoecaria madagascariensis* (Baill.) Müll.Arg.** Habit: Shrub. Habitat: Forest and thicket, commonly along rivers, ca. 50 m. Voucher: Dale in FD 3651 (EA).

***Givotia gosai* Radcl.–Sm.** Habit: Shrub. Habitat: *Acacia*-*Commiphora* open bushland, 50–600 m. Voucher: Gillett 16487 (EA).

***Jatropha curcas* L.** Habit: Tree. Habitat: Grassland, ca. 0–200 m. Voucher: Jeffery GW 418 (EA).

**Jatropha hildebrandtii Pax var. hildebrandtii** Habit: Shrub. Habitat: Dunes in open coastal bushland, 0–230 m. Voucher: Robertson SA 3793 (EA).

***Jatropha mollis* Pax** Habit: Shrub. Habitat: *Acacia*-*Commiphora* bushland, ca. 90 m. Voucher: Gillett 16430 (EA).

***Jatropha multifida* L.** Habit: Shrub. Habitat: Cultivated as a hedge plant and garden ornamental. Voucher: Bally PRO 2156 (EA).

***Jatropha prunifolia* Pax** Habit: Herb. Habitat: Dry coastal forest, thicket, and bushland, (20–) 60–350 m. Vouchers: Ngumbau V & Mwadime N V0156 (EA, HIB), Luke WRQ & Robertson SA 1472, Greenway PJ & Kanuri K 12756 (EA).

***Jatropha spicata* Pax** Habit: Herb. Habitat: Deciduous bushland and thicket, ca. 45 m. Voucher: Sangai GW 961 (EA).

**Jatropha stuhlmannii Pax subsp. stuhlmannii** Habit: Herb. Habitat: Grassland and coastal bushland, 0–100 m. Voucher: Gillett JB 20383 (EA).

***Jatropha velutina* Pax & K. Hoffm.** Habit: Herb. Habitat: Thicket and in *Acacia*-*Commiphora* bushland, 300–350 m. Voucher: Robertson SA & Luke WRQ 2213 (EA).

**Macaranga capensis (Baill.) Sim var. capensis** Habit: Tree. Habitat: Evergreen forest, (10–) 75 m. Voucher: Luke WRQ & Robertson SA 2759 (EA).

***Mallotus oppositifolius* (Geiseler) Müll.Arg.** Habit: Shrub. Habitat: Moist forests, 5–36 m. Vouchers: SAJIT–006183 & 005917, Ngumbau V & Mwadime N V0517 (EA, HIB), Luke Q 1401, Thomas Mwadime & Mrs Robertson SA 7798 (EA).

***Micrococca mercurialis* (L.) Benth.** Habit: Herb. Habitat: Woodland and bushland, ca. 36 m. Vouchers: SAJIT–006180 (EA, HIB), Festo L & Luke Q 2467, Luke WRQ 3338 (EA).

***Micrococca scariosa* Prain** Habit: Shrub. Habitat: Forest and associated thickets, 10–170 m. Vouchers: Ngumbau V & Mwadime N V029 (EA, HIB), Faden RB 216 (EA).

***Plesiatropha* sp. (*Mildbraedia* sp. A of FTEA)** Habit: Tree. Habitat: Bushland or forest edges, 70 m. Vouchers: Moggridge 398, Padwa B715 (EA).

**Necepsia castaneifolia subsp. kimbozensis (Radcl.-Sm.) Bouchat & J.Léonard** Habit: Tree. Habitat: Moist forests, ca. 150 m. Voucher: Malombe I, Mwadime N & Saidi 1654 (EA).

**Plesiatropha carpinifolia (Pax) Breteler var. carpinifolia** Habit: Shrub. Habitat: Forest, forest edges, and coastal woodland, 0–272 m. Vouchers: SAJIT–005923, Ngumbau V & Mwadime N V042 (EA, HIB), Luke WRQ & Robertson SA 1492, Ross KS 196, Festo L & Luke Q 2533 (EA).

***Pycnocoma littoralis* Pax** Habit: Shrub. Habitat: Dry coastal forest, ca. 90 m. Vouchers: Vorontsova MS 117, Luke WRQ & Robertson SA 2832 (EA): Vulnerable.

**Ricinodendron heudelotii var. tomentellum (Hutch. & E.A.) Radcl.–Sm.** Habit: Tree. Habitat: Semi-deciduous and dry evergreen forest, ca. 220–600 m. Vouchers: RB & AJ Faden 74/312 & 71/771 (EA).

***Shirakiopsis trilocularis* (Pax & K. Hoffm.) Esser** Habit: Tree. Habitat: Evergreen forest, ca. 210 m. Voucher: Luke WRQ & PA 4067 (EA).

***Spirostachys africana* Sond.** Habit: Tree. Habitat: Deciduous woodland, bushland, and wooded grassland, ca. 15 m. Vouchers: Robertson SA 4236, Dale in FD 3769 (EA).

***Spirostachys venenifera* (Pax) Pax** Habit: Tree. Habitat: Along rivers in deciduous and coastal bushland, ca. 0–30 m. Voucher: Verdcourt 5295A (EA).

***Suregada zanzibariensis* Baill.** Habit: Shrub. Habitat: Coastal forest, woodland, and bushland, 0–213 m. Vouchers: SAJIT–006204, 005948, 005466 & 004650, Ngumbau V & Mwadime N V0328 (EA, HIB), Kimeu JM, Meso M & Ot 624, Luke WRQ 3154 (EA).

**Tannodia tenuifolia var. glabrata Prain** Habit: Shrub. Habitat: Lowland evergreen forest and riverine, 160–660 m. Vouchers: Hawthorne 178, Faden et al. 77/514, Luke WRQ 3146 (EA).

***Tragia furialis* Bojer** Habit: Climber. Habitat: Forest and disturbed places, 0–250 m. Vouchers: SAJIT–006117 (EA, HIB), Luke Q 1403 (EA).

***Tragia glabrescens* Pax** Habit: Herb. Habitat: Coastal bushland and thickets, 170–365 m. Vouchers: Kuchar 7342, Polhil & Paulo 491, Kassner 466 (EA).

***Tragia hildebrandtii* Müll.Arg.** Habit: Herb. Habitat: Riverine, flood plains or disturbed places, 15 m. Voucher: Magogo FC & Glover PE 1000 (EA).

***Tragia kirkiana* Müll.Arg.** Habit: Climber. Habitat: Wooded and open grassland, ca. 183 m. Vouchers: Ngumbau V & Mwadime N V0195 (EA, HIB), Magogo FC & Glover PE 1136 (EA).

***Tragia plukenetii* Radcl.–Sm.** Habit: Climber. Habitat: Riverine, floodplains, and valley grassland, 0–100 m. Vouchers: Rawlins 83, JM Reitsma 410 (EA).

***Tragiella natalensis* (Sond.) Pax & K. Hoffm.** Habit: Climber. Habitat: Forest edges, bushland, riverine, lakesides and disturbed places, ca. 80–230 m. Vouchers: Ngumbau V & Mwadime N V061 (EA, HIB), Magogo FC & Glover PE 408 (EA).

**F80. Fabaceae**

80 Genera, 226 Species

**Abrus precatorius subsp. africanus Verdc.** Habit: Liana. Habitat: Grassland, thicket, and bushland, ca. 15 m. Vouchers: Tweedie 1249, Festo L, Luke Q & P 2708, Magogo FC & Glover PE 644 (EA).

***Abrus* sp. A of FTEA** Habit: Herb. Habitat: Forest edge and burnt grassland, 300–420 m. Vouchers: Magogo & Glover 548 & 122, Luke WRQ 3163 (EA): Endemic.

**Aeschynomene cristata var. pubescens J. Léonard** Habit: Shrub. Habitat: Swamps, edges of dams, lakes, and rivers, ca. 350 m. Vouchers: Drummond & Hemsley 3930 & 4177, Greenway & Rawlins 9448 (EA).

**Aeschynomene cristata Vatke var. cristata** Habit: Shrub. Habitat: Swamps in grassland, ca. 5–30 m. Voucher: Festo L & Luke Q 2455 (EA).

***Aeschynomene indica* L.** Habit: Shrub. Habitat: Mostly in wet places, floodplain grassland, wooded grassland, and desert grassland, ca. 96 m. Vouchers: Polhill & Paulo 667, Gillespie 157, JB Gillett 16508 (EA).

**Aeschynomene uniflora E. Mey. var. uniflora** Habit: Shrub. Habitat: Swamps, marshy grassland, riverside, and bushland, ca. 15 m. Vouchers: Drummond & Hemsley 4016, Bogdan 2617, Rawlins 36, Magogo FC & Glover PE 757, Luke Q 5671 (EA).

***Aeschynomene* sp. B of FTEA** Habit: Shrub. Habitat: Roadsides, ca. 25 m. Vouchers: Rawlins 410, Patterson 32 (EA): Endemic.

***Afzelia quanzensis* Welw.** Habit: Tree. Habitat: Thickets, woodland, and lowland dry evergreen forest, ca. 0–300 m. Vouchers: SAJIT–005470 (EA, HIB), Battiscombe 35, Hildebrandt 1967, Jeffery 115, Gilbert MG & Kanuri K 5863, Magogo FC & Glover PE 49 (EA).

**Albizia adianthifolia (Schum.) W. Wight var. adianthifolia** Habit: Tree. Habitat: Lowland rainforest, deciduous woodland, and wooded grassland, ca. 30 m. Vouchers: Hildebrandt 1936, Gardner 1443, RM Graham 312, Cunningham-van Someren GR Sh49 (EA).

***Albizia anthelmintica* Brongn.** Habit: Tree. Habitat: In dry open woodland, ca. 50–110 m. Vouchers: Ngumbau V & Mwadime N V0492 (EA, HIB), Rukunga G, Mungai G & Muthaura C 019/04 (EA).

**Albizia glaberrima var. glabrescens (Oliv.) Brenan** Habit: Tree. Habitat: Lowland rainforest, riverine forest, and coastal evergreen bushlands, 0–760 m. Vouchers: Dale in FH 3540, Mulwa PCW 58 (EA).

**Albizia gummifera (J.F. Gmel.) C.A.Sm. var. gummifera** Habit: Tree. Habitat: Rainforest and riverine forest, ca. 30 m. Vouchers: Shimba Hills Survey Unit 64, Luke WRQ 192 (EA).

***Albizia versicolor* Oliv.** Habit: Tree. Habitat: Deciduous woodland, bushland, and wooded grassland, ca. 0–153 m. Vouchers: Ngumbau V & Mwadime N V0508 (EA, HIB), St. Barbe Baker 43, CW Elliot Q 87 in FH 1677 & in CM 14009, RM Graham N331 in FH 1724 (EA).

**Alysicarpus glumaceus subsp. macalusoi (Mattei) Verdc.** Habit: Herb. Habitat: Sand dunes close to the sea, 0–10 m. Vouchers: Rawlins 47 & 99, Greenway & Rawlins 9285, Luke Q 5465 (EA).

**Alysicarpus glumaceus var. intermedius Verdc.** Habit: Herb. Habitat: Lowland and coastal bushland, 0–300 m. Vouchers: Bogdan A 2601, Drummond & Hemsley 3779 (EA).

***Alysicarpus ovalifolius* (Schumach.) J. Léonard** Habit: Herb. Habitat: Grassland, ca. 60 m. Vouchers: Tweedie 963, Rawlins 621, Polhill & Paulo 662 (EA).

**Alysicarpus vaginalis (L.) DC. var. vaginalis** Habit: Herb. Habitat: Old cultivations, roadsides, bushland, and open woodland, ca. 36 m. Vouchers: SAJIT–006168 (EA, HIB), Drummond & Hemsley 1063 & 3840, Luke Q 5682 (EA).

**Alysicarpus vaginalis var. villosus Verdc.** Habit: Herb. Habitat: Not known, ca. 200–420 m. Voucher: Magogo & Glover 58 (EA).

***Angylocalyx braunii* Harms** Habit: Tree. Habitat: Understorey of lowland rainforest, dry evergreen, and riverine forest, 0–800 m. Vouchers: SAJIT–005555, Ngumbau V & Mwadime N V031 (EA, HIB), Drummond & Hemsley 1145, Greenway & Rawlins 9368, CW Elliot in FD 1550, Robertson SA & Luke WRQ 5173 (EA): Vulnerable.

***Bauhinia mombassae* Vatke** Habit: Shrub. Habitat: Forest in riverine areas, ca. 210 m. Vouchers: Ngumbau V & Mwadime N V086 (EA, HIB), Wakefield, Hildebrandt 2006, Kelly 1230 & in CM 13911, Faden RB & AJ, Gillett JB & Gachathi N 77/442, Luke WRQ & Robertson SA 2793 (EA): Endangered, Endemic.

***Bauhinia taitensis* Taub.** Habit: Shrub. Habitat: Deciduous bushland, 330–610 m. Voucher: Parker I 307 (EA).

***Brachystegia spiciformis* Benth.** Habit: Tree. Habitat: Deciduous woodland, ca. 15 m. Vouchers: Greenway 9657, Jeffery 102, Moomaw 940, Kimeu JM 527, Magogo FC & Glover PE 456 (EA).

***Caesalpinia angolensis* (Oliv.) Herend. & Zarucchi** Habit: Liana. Habitat: Lowland rainforest, swamp forest, and coastal evergreen bushland, ca. 20 m. Voucher: Mwadime N 1 (EA).

***Caesalpinia bonduc* (L.) Roxb.** Habit: Shrub. Habitat: Near seashores, 0–15 m. Vouchers: Ngumbau V & Mwadime N V0519 (EA, HIB), RM Graham R 144 in FH 1562, F Thomas 199, Bally 2192 in CM 11617, Kirika P 203 (EA).

***Caesalpinia insolita* (Harms) Brenan & J. B. Gillett** Habit: Tree. Habitat: In a wooded gulley, ca. 150 m. Vouchers: Dale in FH 3572 & in CM 9273, Brenan, Gillett, Kanuri & Chomba 14599, Robertson SA & Luke WRQ 5982 (EA).

***Caesalpinia pulcherrima* (L.) Sw.** Habit: Shrub. Habitat: Near beach, ca. 5 m. Voucher: SAJIT–005456 (EA, HIB): Cultivated.

**Caesalpinia trothae Harms subsp. trothae** Habit: Shrub. Habitat: Deciduous bushland, ca. 150 m. Vouchers: RM Graham U 760 in FH 2239 & in CM 13918, Bamps P 6369 (EA).

***Caesalpinia volkensii* Harms** Habit: Shrub. Habitat: Margins of lowland rainforest, 90–417 m. Vouchers: Ngumbau V & Mwadime N V0321 (EA, HIB), Birch 62/146, Kabuye CHS, Gilbert VC & Robertson SA 110 (EA).

***Canavalia africana* Dunn** Habit: Liana. Habitat: Low altitude evergreen forest, 5–150 m. Voucher: Kassner 203 (EA).

***Canavalia cathartica* Thouars** Habit: Liana. Habitat: Coastal bushland on coral, 0–30 (–420) m. Vouchers: SAJIT–006016, Ngumbau V & Mwadime N V0187 (EA, HIB), Moomaw 860, Verdcourt 1071, Thorold 1554, Magogo FC & Glover PE 64 (EA).

***Canavalia rosea* (Sw.) DC.** Habit: Liana. Habitat: Beaches and edges of coastal bushland, 0–30 m. Vouchers: SAJIT–006257 (EA, HIB), Birch 62/75, Tweedie 1010, Elliot in FD 3364 (EA).

**Cassia abbreviata subsp. beareana (Holmes) Brenan** Habit: Tree. Habitat: Margin of evergreen forest, 460–1200 m. Vouchers: SAJIT–005998 & 006001, Ngumbau V & Mwadime N V0180 (EA, HIB), Jeffery 96, J Adamson 280 in Bally 5971, Kuchar P 13699, Magogo FC & Glover PE 875, Festo L & Luke Q 2421 (EA).

**Cassia abbreviata subsp. kaessneri (Baker f.) Brenan** Habit: Tree. Habitat: Wooded grassland and deciduous bushland, ca. 350 m. Vouchers: Drummond & Hemsley 4059, Luke WRQ & Robertson SA 2526 (EA).

**Cassia afrofistula Brenan var. afrofistula** Habit: Tree. Habitat: Coastal evergreen bushland, 0–90 m. Vouchers: Ngumbau V & Mwadime N V0521 (EA, HIB), J Adamson 192 in Bally 6092, Tweedie 996, RM Graham Z 303 in FH 1791 (EA).

***Centrosema pubescens* Benth.** Habit: Climber. Habitat: Forest, ca. 250 m. Voucher: Ngumbau V & Mwadime N V0243 (EA, HIB): Naturalized.

***Chamaecrista absus* (L.) H. S. Irwin & Barneby** Habit: Herb. Habitat: Grassland, deciduous bushland, and waste ground, ca. 140 m. Vouchers: Ngumbau V & Mwadime N V0232 (EA, HIB), Greenway & Rawlins 8959, Kirika P, Muthoka P & Mbale M 745 (EA).

***Chamaecrista fallacina* (Chiov.) Lock** Habit: Herb. Habitat: Grassland, ca. 100 m. Voucher: Drummond & Hemsley 3766 (EA).

***Chamaecrista mimosoides* (L.) Greene** Habit: Herb. Habitat: Forest margins, wooded grassland, grassland, cultivated, and waste ground, ca. 0–227 m. Vouchers: Ngumbau V & Mwadime N V0279 (EA, HIB), Tweedie 992, Moggridge 481, RM Graham DD453 in FH 1992, Jex Blake in Napier 2267 in CM 4980, Festo L & Luke Q 2452, Luke WRQ et al. 5709 (EA).

***Chamaecrista telfairiana* (Hook.f.) Lock** Habit: Herb. Habitat: Forest margin, ca. 10 m. Vouchers: Luke Q 6134, Magogo FC & Glover PE 341 (EA).

***Chamaecrista zambesica* (Oliv.) Lock** Habit: Herb. Habitat: Grassland and cultivated land, 80–460 m. Vouchers: Jeffery 232, Jeffery 288, Whyte, Festo L Luke Q & P 2627 (EA).

***Clitoria falcata* Lam.** Habit: Climber. Habitat: Forest, ca. 15–280 m. Vouchers: SAJIT–006230, Ngumbau V & Mwadime N V0249 (EA, HIB), Nyange M 456 (EA).

***Clitoria ternatea* L.** Habit: Climber. Habitat: Grassland, scrub, bushland, and old cultivations, 0–80 m. Vouchers: Battiscombe 267, Magogo FC & Glover PE 991 (EA).

***Cordyla africana* Lour.** Habit: Tree. Habitat: Riverine and ground water forest, ca. 150 m. Vouchers: Dale 3852, Luke WRQ 882 (EA).

**Craibia brevicaudata (Vatke) Dunn subsp. brevicaudata** Habit: Tree. Habitat: Evergreen coastal forest and fringing forest, 0–382 m. Vouchers: SAJIT–006077 (EA, HIB), Battiscombe 466, Greenway 9493, Muchiri J 426 (EA).

***Crotalaria axillaris* Aiton** Habit: Shrub. Habitat: Forest margins, deciduous woodland, bushland, and on coral crags, ca. 0–226 m. Vouchers: Ngumbau V & Mwadime N V046 (EA, HIB), Polhill & Paulo 843, Festo L & Luke Q 2539, Magogo FC & Glover PE 142 (EA).

**Crotalaria barkae subsp. teitensis (Sacleux) Polhill** Habit: Herb. Habitat: Deciduous woodland and bushland, ca. 243 m. Vouchers: Greenway 10436, Gillett JB 19548 (EA).

**Crotalaria barkae subsp. zimmermannii (Baker f.) Polhill** Habit: Herb. Habitat: Grassland and forest edges, 0–700 m. Vouchers: Hacker 122, Rawlins in EAH 11206, Greenway & Rawlins in EAH 11335, Soest LV 16013 (EA).

***Crotalaria bernieri* Baill.** Habit: Herb. Habitat: Grassland, coral rags, and foreshores, 0–100 m. Vouchers: Thorold 1562, Tweedie 1002, Greenway & Rawlins 9464 (EA).

**Crotalaria deserticola subsp. orientalis Polhill** Habit: Herb. Habitat: Coastal grassland and open woodland, ca. 0–243 m. Vouchers: RM Graham Q537 in FD 1938, Thorold 1557, Greenway & Rawlins 8960 (EA).

***Crotalaria emarginata* Benth.** Habit: Herb. Habitat: Grassland, swamp margins, cultivated ground, and roadsides, 0–300 m. Vouchers: Ngumbau V & Mwadime N V0288 (EA, HIB), Verdcourt 1105, Drummond & Hemsley 1033, Greenway & Rawlins 8958, Magogo FC & Glover PE 99 (EA).

***Crotalaria emarginella* Vatke** Habit: Herb. Habitat: Deciduous bushland, semi-desert grassland, upland evergreen bushland, and grassland, ca. 750 m. Voucher: Verdcourt 2095 (EA).

***Crotalaria goodiiformis* Vatke** Habit: Shrub. Habitat: Upland rainforest, dry evergreen forest, deciduous woodland, bushland, and wooded grassland, ca. 75 m. Vouchers: Drummond & Hemsley 3869, Luke Q 1526, Robertson SA & Luke WRQ 5998 (EA).

***Crotalaria grata* Polhill** Habit: Shrub. Habitat: Deciduous coastal bushland and grassland, below 50 m. Vouchers: MacNaughton 138 in FD 2717, Rawlins 781, Polhill & Paulo 583 (EA): Endemic.

***Crotalaria hyssopifolia* Klotzsch** Habit: Herb. Habitat: Deciduous woodland and grassland, ca. 10 m. Vouchers: RM Graham in FD 2026, Festo L & Luke Q 2607 (EA).

***Crotalaria juncea* L.** Habit: Herb. Habitat: Deciduous bushland and grassland, ca. 100 m. Voucher: Jeffery 530 (EA).

***Crotalaria kirkii* Baker** Habit: Herb. Habitat: Grassland and *Brachystegia* woodland, ca. 100 m. Vouchers: RM Graham 273, Hildebrandt 1932, Thorold 1558 (EA).

**Crotalaria laburnifolia L. subsp. laburnifolia** Habit: Shrub. Habitat: Deciduous bushland, grassland, and secondary scrub, 0–360 m. Voucher: Polhill & Paulo 707 (EA).

**Crotalaria laburnoides Klotzsch var. laburnoides** Habit: Herb. Habitat: Grassland, sand dunes, coral outcrops, and bushland near coast, ca. 10 m. Vouchers: SAJIT–006097 & 006259 (EA, HIB), Bogdan 5354, Irwin 173, Bogdan 2536 (EA).

**Crotalaria laburnoides var. nudicarpa Polhill** Habit: Herb. Habitat: Dry coastal scrub, beach dunes, and woodland, 0–10 m. Vouchers: Courtald 21, Tweedie 2201 (EA): Endangered.

***Crotalaria malindiensis* Polhill** Habit: Herb. Habitat: Woodland and *Acacia* bushland, grassland, and dunes near seashore, 70–230 m. Vouchers: Bogdan 2523, Tweedie 939, Gillespie 220, Festo L & Luke Q 2554 (EA).

***Crotalaria microcarpa* Benth.** Habit: Herb. Habitat: *Brachstegia* woodland, deciduous bushland, and grassland, ca. 100 m. Vouchers: Verdcourt 1928, Bork 15408 (EA).

***Crotalaria patula* Polhill** Habit: Herb. Habitat: Deciduous bushland, grassland, cultivated ground, and roadsides, 125–420 m. Vouchers: Drummond & Hemsley 4055, Polhill & Paulo 912 (EA).

***Crotalaria polysperma* Kotschy** Habit: Herb. Habitat: Grassland, woodland, often in disturbed places. Voucher: Luke WRQ & PA sr.

**Crotalaria retusa L. var. retusa** Habit: Herb. Habitat: Coastal grassland, waste places, and old cultivations, 0–240 m. Vouchers: SAJIT–005454, Ngumbau V & Mwadime N V0274 (EA, HIB), Drummond & Hemsley 3898, Festo L & Luke Q 2356 (EA).

**Crotalaria retusa var. tunguensis (Lima) Polhill** Habit: Herb. Habitat: Coastal grassland, bushland, coral crags, and salt marsh edges, 0–150 m. Vouchers: Napier 3283, Verdcourt 1097, Rawlins 875, Ndakala J 120 (EA).

***Crotalaria rhynchocarpa* Polhill** Habit: Shrub. Habitat: Dunes and secondary coastal bushland, less than 50 m. Vouchers: Polhill & Paulo 706, CW Elliot in FD 3375, Verdcourt 2120, Greenway PJ & Rawlins SP 9437 (EA).

***Crotalaria vallicola* Baker f.** Habit: Herb. Habitat: Forest, ca. 136 m. Voucher: SAJIT–005954 (EA, HIB).

***Crotalaria vasculosa* Benth.** Habit: Herb. Habitat: Grassland and woodland, ca. 20 m. Vouchers: Jeffery 847, Tweedie 1049, Luke 3411 (EA).

***Cynometra greenwayi* Brenan** Habit: Tree. Habitat: Sand dunes with scattered coral rock outcrops, ca. 8 m. Voucher: Greenway PJ 10440 (EA): Endemic.

***Cynometra lukei* Beentje** Habit: Tree. Habitat: Forest, 1–50 m. Voucher: Q Luke & SA Robertson 1267 (EA): Endangered.

***Cynometra suaheliensis* (Taub.) Baker f.** Habit: Tree. Habitat: Lowland dry evergreen forest, riverine forest, and coastal evergreen bushland, 0–150 m. Vouchers: RM Graham U 767 in FH 2238, Polhill & Paulo 737 & 824, Robertson SA 7257, Moomaw JC 1250 (EA).

***Cynometra webberi* Baker f.** Habit: Tree. Habitat: Evergreen woodland and deciduous woodland, 0–150 m. Vouchers: Drummond & Hemsley 4257, RM Graham B 699 in FH 2168, Polhill & Paulo 838, Graham RM 2168, Luke WRQ & Robertson SA 2814 (EA): Vulnerable.

**Dalbergia boehmii Taub. subsp. boehmii** Habit: Tree. Habitat: Dry evergreen, deciduous types of forest, woodland, bushland, and thicket, ca. 0–110 m. Vouchers: Ngumbau V & Mwadime N V0489 (EA, HIB), Wakefield (EA).

***Dalbergia bracteolata* Baker** Habit: Liana. Habitat: Coastal and deciduous thicket, ca. 100 m. Vouchers: Kassner 220, Luke WRQ & Robertson SA 2780 (EA).

***Dalbergia eremicola* Polhill** Habit: Shrub. Habitat: Bushlands and thickets, 75–810 m. Voucher: Bally PRO & Melville R 15252 (EA): Near Threatened.

***Dalbergia gloveri* Q. Luke, ined.** Habit: Liana. Habitat: Coastal forest, 30–320 m. Voucher: Luke WRQ & Baer S 8247 (EA): Endangered, Endemic.

***Dalbergia melanoxylon* Guill. & Perr.** Habit: Tree. Habitat: Deciduous woodland deciduous, coastal bushland, and wooded grassland, ca. 0–300 m. Vouchers: Drummond & Hemsley 4139, Greenway 8968, Robertson SA & Luke WRQ 5518, Magogo FC & Glover PE 841 (EA): Near Threatened.

**Dalbergia arbutifolia Baker subsp. arbutifolia** Habit: Shrub. Habitat: Woodland, ca. 0–300 m. Voucher: Chidzinga S & Mwadime N 752 (EA).

***Dalbergia vacciniifolia* Vatke** Habit: Liana. Habitat: Coastal bushland and thicket, 0–100 m. Vouchers: Bally 8896, Dale in FD 3774, Polhill & Paulo 697, Bally PRO 13260 & 13070, Robertson SA 4220 (EA): Vulnerable.

***Dendrolobium umbellatum* (L.) Benth.** Habit: Shrub. Habitat: Sandy beaches, near the sea. Vouchers: SAJIT–006104, Ngumbau V & Mwadime N V0511 (EA, HIB), Napier 3294 in CM 6307 (EA).

***Derris trifoliata* Lour.** Habit: Climber. Habitat: Mangrove swamps, coastal bushland, and thicket, 0–30 m. Vouchers: Rawlins 886, Polhill & Paulo 686, Dale in FD 3844 (EA).

***Desmodium adscendens* (Sw.) DC.** Habit: Herb. Habitat: Old cultivated fields, and grassland, ca. 15 m. Vouchers: SAJIT–006215 (EA, HIB), Magogo & Glover 534, Luke WRQ & Robertson SA 2692 (EA).

**Desmodium barbatum var. dimorphum (Baker) B. G. Schub.** Habit: Herb. Habitat: Grassland, woodland, ca. 372 m. Vouchers: SAJIT–006163 (EA, HIB), Magogo FC & Glover PE 1266 (EA).

**Desmodium barbatum var. procumbens B. G. Schub.** Habit: Herb. Habitat: Open and wooded grassland, ca. 35–1800 m. Vouchers: Kassner 121, Magogo FC & Glover PE 1 (EA).

***Desmodium gangeticum* (L.) DC.** Habit: Herb. Habitat: Deciduous woodland, riverine forest, and swampy places, ca. 183 m. Vouchers: SAJIT–006174, Ngumbau V & Mwadime N V0209 (EA, HIB), Thorold 1533, Kassner 391, Magogo FC & Glover PE 385 (EA).

***Desmodium ramosissimum* G. Don** Habit: Herb. Habitat: Grassland or dry sandy open areas, ca. 0–150 m. Vouchers: Magogo & Glover 27, Luke WRQ 3114 (EA).

**Desmodium salicifolium var. densiflorum B. G. Schub.** Habit: Herb. Habitat: Grassland, ca. 80 m. Vouchers: SAJIT–006197 (EA, HIB), Nyange M 372 (EA).

***Desmodium triflorum* (L.) DC** Habit: Herb. Habitat: Roadsides and lawns, ca. 0–100 m. Voucher: Luke WRQ 3120 (EA).

***Desmodium velutinum* (Willd.) DC.** Habit: Herb. Habitat: Forest edges, deciduous woodland, wooded, and open grassland, ca. 156 m. Vouchers: Ngumbau V & Mwadime N V0430 (EA, HIB), Drummond & Hemsley 3800, Rawlins 37, Magogo FC & Glover PE 1074 (EA).

***Dialium orientale* Baker f.** Habit: Tree. Habitat: Lowland dry evergreen forest and coastal evergreen bushland, 0–60 m. Vouchers: SAJIT–006458 (EA, HIB), Elliot in FH 1489 in CM 16435, Dale in FH 3898, J Adamson 342 in Bally 3836, Rawlins SP 339 (EA): Near Threatened.

**Dichrostachys cinerea (L.) Wight & Arn. subsp. cinerea** Habit: Shrub. Habitat: Deciduous bushland, scrub, wooded grassland, and deciduous woodland, ca. 80 m. Vouchers: SAJIT–005588 (EA, HIB), Drummond & Hemsley 1178 (EA).

**Dichrostachys cinerea subsp. keniensis Brenan & Brummitt** Habit: Shrub. Habitat: Bushland. Voucher: Magogo FC & Estes R 1237 (EA): Endemic.

**Dolichos sericeus subsp. glabrescens Verdc.** Habit: Climber. Habitat: Bushland. Voucher: Nash LT 32 (EA).

***Dolichos trilobus* L.** Habit: Climber. Habitat: Grassland, bushland, and forest, ca. 0–450 m. Vouchers: Ngumbau V & Mwadime N V0352 (EA, HIB), RM Graham in FD 2225 & 1952, Jeffery 327, Magogo FC & Glover PE 1265 (EA).

***Entada leptostachya Harms*** Habit: Liana. Habitat: Forest, ca. 200 m. Voucher: Robertson SA, Davis S & Morley J 7098 (EA)

***Entada rheedii* Spreng.** Habit: Liana. Habitat: Lowland rainforest and evergreen bushland, 0–600 m. Vouchers: Ngumbau V & Mwadime N V0490 (EA, HIB), RM Graham NN757 in FH 2227, Magogo FC & Glover PE 626 (EA).

***Eriosema glomeratum* (Guill. & Perr.) Hook. f.** Habit: Herb. Habitat: Grassland, scrub on coral, and sandy soils, ca. 227 m. Vouchers: SAJIT–005938 & 006162, Ngumbau V & Mwadime N V0285 (EA, HIB), Verdcourt 1885, Drummond & Hemsley 1032, Rawlins, Magogo FC & Glover PE 390 (EA).

***Eriosema parviflorum* E. Mey.** Habit: Herb. Habitat: Grassland, thicket, and swampy grassland, ca. 222 m. Vouchers: SAJIT–005991, Ngumbau V & Mwadime N V082 (EA, HIB), RM Graham in FD 1922, Kassner 444, Jeffery 252, Magogo FC & Glover PE 338 (EA).

**Eriosema pauciflorum Klotzsch var. pauciflorum** Habit: Herb. Habitat: Seasonally burnt grasslands, *Brachystegia* woodland, coastal dry forest, and roadsides, ca. 40 m. Vouchers: Magogo & Glover 404, Nyange M 342 (EA).

***Erythrina abyssinica* DC.** Habit: Tree. Habitat: Wooded grassland and deciduous woodland, ca. 200 m. Voucher: Magogo FC & Glover PE 68 (EA).

***Erythrina sacleuxii* Hua** Habit: Tree. Habitat: Bushland, open woodland, and evergreen coastal forest, 0–450 m. Vouchers: Ngumbau V & Mwadime N V0465 (EA, HIB), Greenway 9646, CW Elliot in FD 1376, Greenway & Rawlins 9361, Cunningham-van Someren GR in FD 9816 (EA).

***Erythrophleum suaveolens* (Guill. & Perr.) Brenan** Habit: Tree. Habitat: Riverine and lowland rainforest, ca. 0–245 m. Vouchers: SAJIT–006053, Ngumbau V & Mwadime N V0266 (EA, HIB), Verdcourt 2129, CW Elliot 1493, Luke WRQ & Robertson SA 220 (EA).

***Galactia argentifolia* S. Moore** Habit: Herb. Habitat: Grassland, 0–450 m. Vouchers: SAJIT–005958 (EA, HIB), Drummond & Hemsley 1137, Bogdan 3908, Graham in FD 1977, Magogo FC & Glover PE 403 (EA).

**Galactia striata var. villosa (Wight & Arn.) Verdc.** Habit: Herb. Habitat: Grassland and coastal bushland, 0–120 m. Voucher: Rawlins in EAH 11270 (EA).

***Gigasiphon macrosiphon* (Harms) Brenan** Habit: Tree. Habitat: Lowland rainforest, 30–600 m. Vouchers: Ngumbau V & Mwadime N V0526 (EA, HIB), TAM Gardner 1, Verdcourt 2404, Verdcourt 3935, Robertson SA 7579 (EA): Endangered.

***Hymenaea verrucosa* Gaertn.** Habit: Tree. Habitat: Lowland dry evergreen forest, woodland, and coastal evergreen bushland, 15–240 m. Vouchers: SAJIT–007184 (EA, HIB), RM Graham B263 in FH Ox. 289 & in CM 13951, Trump 100, Kimeu JM 522, Saufferer S 861, Luke WRQ & Robertson SA 220, RM Graham B263 in FH Ox. 289 & in CM 1395 (EA).

***Indigofera brachynema* J. B. Gillett** Habit: Herb. Habitat: Dry grassland and *Acacia*-*Commiphora* bushland, ca. 186 m. Vouchers: Ngumbau V & Mwadime N V0342 (EA, HIB), Greenway & Rawlins 9295, Luke Q 6142 (EA).

***Indigofera charlierana* Schinz** Habit: Herb. Habitat: Sandy coastal grassland, 0–230 m. Vouchers: Whyte, Tweedie 950, Bogdan 2524, Luke Q 6141 (EA).

***Indigofera colutea* (Burm. f.) Merr.** Habit: Herb. Habitat: Grassland, cultivated area, and bushland, ca. 0–20 m. Vouchers: Tweedie 1003, Robertson SA 3328 (EA).

***Indigofera congesta* Baker** Habit: Herb. Habitat: Grassland, seasonal swamps, and old cultivation, ca. 136 m. Vouchers: SAJIT–005957 (EA, HIB), Whyte, Graham RM 577 (EA).

***Indigofera cuneata* Oliv.** Habit: Herb. Habitat: Scattered tree grassland and cultivation especially on sand, 0–365 m. Vouchers: Makin 429, Drummond & Hemsley 3861, Moomaw 1132, Gillett JB 20348 (EA).

***Indigofera dendroides* Jacq.** Habit: Herb. Habitat: Grassland, cultivated ground, seasonal swamps, and flood plains, ca. 10 m. Vouchers: Rawlins 68 & 343, Magogo FC & Glover PE 1138 (EA).

***Indigofera dyeri* Britten** Habit: Herb. Habitat: Weed of cleared land and bushland, ca. 20 m. Vouchers: Mainwaring in Napier 3040, Robertson SA 3298 (EA).

***Indigofera hirsuta* L.** Habit: Herb. Habitat: Cultivated and waste areas, ca. 36 m. Vouchers: SAJIT–006172 (EA, HIB), MacNaughton 97, Jeffery 35, Luke WRQ & PA 5730 (EA).

***Indigofera kirkii* Oliv.** Habit: Herb. Habitat: Between Mangroves and forest, ca. 2 m. Voucher: Luke 3032 (EA).

***Indigofera longimucronata* Baker f.** Habit: Herb. Habitat: Rocky outcrops, lowland forests, and coconut groves, 0–350 m. Vouchers: Drummond & Hemsley 4178, Whyte A, Jeffery 233, Schrire BD, Robertson SA & Stirton CH 2573, Magogo FC & Estes R 1208 (EA).

***Indigofera longiracemosa* Baill.** Habit: Herb. Habitat: Coastal areas, 0–100 m. Vouchers: Mearns 2169, Swynnerton 281, Gisau in EAH 11207, Luke Q 1491, Luke WRQ & PA 5722A (EA).

***Indigofera malindiensis* J.B. Gillett** Habit: Herb. Habitat: Wooded sand dunes and on riverbanks, 0–450 m. Vouchers: Rawlins 941, Hacker 87, Rawlins SP 610 (EA).

***Indigofera microcarpa* Desv.** Habit: Herb. Habitat: Cultivated area, on the shores of receding lakes and rivers, 0–200 m. Vouchers: Jeffery 457, Rawlins 365, Luke Q 6120 (EA).

**Indigofera paniculata Vahl ex Pers. subsp. paniculata** Habit: Herb. Habitat: Grassland, dried up swamps, and old cultivation, ca. 0–372 m. Vouchers: SAJIT–006165 & 006171 (EA, HIB), Drummond & Hemsley 3965, Whyte, Mearns 2164, Magogo FC & Glover PE 290 (EA).

**Indigofera schimperi var. baukeana (Vatke) J. B. Gillett** Habit: Herb. Habitat: Grassland, ca. 100 m. Voucher: Drummond & Hemsley 3744 (EA).

***Indigofera spicata* Forssk.** Habit: Herb. Habitat: Common in disturbed grassland, cultivated areas, and waste place, 0–200 m. Voucher: Thorold in FD 1577 (EA).

**Indigofera strobilifera (Hochst.) Baker subsp. strobilifera** Habit: Herb. Habitat: Deciduous woodland, bushland, and waste places, ca. 36 m. Vouchers: SAJIT–006170 (EA, HIB), Rawlins 374 (EA).

**Indigofera strobilifera subsp. lanuginosa (Baker f.) J. B. Gillett** Habit: Herb. Habitat: Sandy grassland near the coast, 0–15 m. Vouchers: Hildebrandt 927/b, Bogdan 2572, Festo L & Luke Q 2464 (EA).

**Indigofera tanganyikensis var. paucijuga J. B. Gillett** Habit: Herb. Habitat: Grassland, ca. 10–350 m. Vouchers: Greenway PJ & Rawlins SP 8921, Drummond RB & Hemsley JH 4056 (EA).

**Indigofera tinctoria L. var. tinctoria** Habit: Herb. Habitat: Bush margins, cultivation, and secondary growth, ca. 100 m. Voucher: Peter 45187 (EA).

**Indigofera trita var. subulata (Poir.) Ali** Habit: Herb. Habitat: Secondary growth, ca. 0–237 m. Vouchers: Ngumbau V & Mwadime N V0189 (EA, HIB), Tweedie 988, Magogo FC & Glover PE 685 (EA).

***Indigofera vohemarensis* Baill.** Habit: Herb. Habitat: Grassy and stony slopes, ca. 20 m. Vouchers: Napier 3310, Robertson SA 3482 & 3296 (EA).

**Indigofera wituensis Baker f. var. wituensis** Habit: Herb. Habitat: Grassland, 0–300 m. Vouchers: A Whyte, Polhill & Paulo 889, Rawlins 73 (EA).

***Indigofera zanzibarica* J. B. Gillett** Habit: Herb. Habitat: Damp open places, often on sand, 0–100 m. Vouchers: SAJIT–006189 (EA, HIB), Rawlins 59 & 193, Faden AJ & RB 1128 (EA).

***Indigofera zenkeri* Baker f.** Habit: Herb. Habitat: Woodland, grassland and, cultivated area, ca. 0–300 m. Vouchers: Verdcourt 1879, OM Mwangangi 1315 (EA).

***Julbernardia magnistipulata* (Harms) Troupin** Habit: Tree. Habitat: Lowland rainforest, riverine forest, and coastal evergreen bushland, ca. 0–206 m. Vouchers: Ngumbau V & Mwadime N V0501 (EA, HIB), Drummond & Hemsley 4039, Sulemani in FH 3236, Polhill & Paulo 803, Robertson SA 6992 (EA).

**Lablab purpureus subsp. uncinatus Verdc.** Habit: Climber. Habitat: Variety of habitats. Voucher: Polhill RM & Paulo S 699 (EA).

***Leucaena leucocephala* (Lam.) de Wit** Habit: Tree. Habitat: A cultivated plant, ca. 50 m. Voucher: Bally 6363 (EA): Cultivated.

***Lysiloma latisiliquum* (L.) Benth.** Habit: Tree. Habitat: Along the lake shores, ca. 300 m. Voucher: Magogo FC & Glover PE 132 (EA): Exotic.

**Macrotyloma axillare var. glabrum (E. Mey.) Verdc.** Habit: Climber. Habitat: Mixed woodland, grassland, often near rivers, ca. 266 m. Vouchers: SAJIT–005940, Ngumbau V & Mwadime N V0292 & 050 (EA, HIB), Magogo FC & Glover PE 364 (EA).

**Macrotyloma uniflorum var. benadirianum (Chiov.) Verdc.** Habit: Climber. Habitat: Sand dunes, thin soil on coral rag, sea level. Vouchers: Greenway 9268, Rawlins 4 & 41 (EA).

**Macrotyloma uniflorum var. verrucosum Verdc.** Habit: Climber. Habitat: Grassland with scattered *Hyphaene*, *Sterculia*, dry *Acacia*, *Commiphora* thickets, 15–540 m. Vouchers: Drummond & Hemsley 1191, Tweedie 1200, Polhill & Paulo 886, Magogo FC & Glover PE 200 (EA).

***Microcharis garissaensis* (J.B. Gillett) Schrire** Habit: Herb. Habitat: *Acacia*-*Commiphora* open bushland, 100–700 m. Vouchers: Gillett 16530 & 16445 (EA).

***Microcharis latifolia* Benth.** Habit: Herb. Habitat: Seasonally damp grassy places, 0–500 m. Vouchers: Rawlins 74, Festo L & Luke Q 2574 (EA).

**Microcharis microcharoides var. latistipulata (J.B. Gillett) Schrire** Habit: Herb. Habitat: Forest, grassland, and savannah, ca. 100 m. Vouchers: Adamson T 53, Drummond RB & Hemsley JH 3784 (EA).

***Millettia lasiantha* Dunn** Habit: Liana. Habitat: Coastal and riverine evergreen forest, 10–300 m. Vouchers: Ngumbau V & Mwadime N V0392 & V062 (EA, HIB), Drummond & Hemsley 1122, RM Graham in FD 1801, Festo L, Luke Q & P 2775, Luke WRQ 3201 (EA).

***Millettia usaramensis* Taub.** Habit: Tree. Habitat: Wooded grassland and margins of lowland forest, 10–700 m. Vouchers: SAJIT–005481, Ngumbau V & Mwadime N V0531 (EA, HIB), Wakefield, RM Graham in FD 1779, Drummond & Hemsley 3885, Robertson SA & Luke WRQ 5188 (EA).

***Mimosa pigra* L.** Habit: Herb. Habitat: Swamps, especially along rivers and lake shores, ca. 15 m. Vouchers: SAJIT–006229 (EA, HIB), Jeffery K165, Luke WRQ 3148 (EA).

**Mimosa pudica var. unijuga (Duchass. & Walp.) Griseb.** Habit: Herb. Habitat: Probably an introduced weed, ca. 200 m. Vouchers: Jeffery K228, Magogo FC & Glover PE 662 (EA): Naturalized.

**Mucuna gigantea subsp. quadrialata (Baker) Verdc.** Habit: Liana. Habitat: Bushland and forest edges, ca. 40 m. Vouchers: RM Graham in FD 1937, Bally 1883, Nyange 580 (EA).

**Mucuna pruriens (L.) DC. var. pruriens** Habit: Liana. Habitat: Course grassland, bushland, riverine forest, forest edges, and abandoned cultivations, 0–700 m. Vouchers: Verdcourt 1873, RM Graham in FD 1960, Tweedie 1038, Bogdan 255A (EA).

***Mundulea sericea* (Willd.) A. Chev.** Habit: Shrub. Habitat: Lowland evergreen forest and deciduous bushland, ca. 10 m. Vouchers: Drummond & Hemsley 4208, Simpson BL 160, Shimba Hills Survey Unit 19 (EA).

***Neptunia oleracea* Lour.** Habit: Herb. Habitat: Freshwater of pools, lakes, and swamps, ca. 30 m. Vouchers: Mrs J Adamson 434 in Bally 6133, Leauthaud C 11, TPR781 (EA).

***Newtonia erlangeri* (Harms) Brenan** Habit: Tree. Habitat: Deciduous woodland, *Euphorbia* bushland, and thickets, ca. 35 m. Vouchers: Power in EAH 10971, Greenway & Rawlins 9435, Luke Q 1473 (EA): Endangered.

***Newtonia paucijuga* (Harms) Brenan** Habit: Tree. Habitat: Lowland rainforest and riverine forest, 75–300 m. Vouchers: Battiscombe 93, Webber 607 in CM 16977, Moggridge 384, Hawthorne W 483, Luke WRQ & Robertson SA 215 (EA): Vulnerable.

***Ophrestia hedysaroides* (Willd.) Verdc.** Habit: Liana. Habitat: Bushland, forest edges, grassland, and old cultivations, ca. 100 m. Vouchers: Verdcourt 1903, Kirk, RM Graham in FD 1914, Luke WRQ et al. 4334 (EA).

***Ormocarpum kirkii* S. Moore** Habit: Shrub. Habitat: *Acacia*-*Commiphora* or coastal bushland, grassland, ca. 20–153 m. Vouchers: Ngumbau V & Mwadime N V0509 (EA, HIB), Rawlins 273, Robertson SA 4226 (EA).

**Ormocarpum sennoides subsp. zanzibaricum Brenan & J.B. Gillett** Habit: Shrub. Habitat: Scrub on coral rock near shore, woodland, and shady places by streams, 0–600 m. Vouchers: SAJIT–006024 & 005566, Ngumbau V & Mwadime N V071 (EA, HIB), CG van Someren Sh127, RM Graham B 488 in FD 1980, Rawlins 710, Beentje 3738 (EA).

***Paramacrolobium coeruleum* (Taub.) J. Léonard** Habit: Tree. Habitat: Lowland rainforest, 8–360 m. Vouchers: Ngumbau V & Mwadime N V0362 (EA, HIB), Dale 3628, Greenway 9804, Pakia M 974 (EA).

***Parkia filicoidea* Oliv.** Habit: Tree. Habitat: Lowland rainforest and riverine forest, ca. 250 m. Vouchers: Hildebrandt 1975, Battiscombe 1 & Cunningham-van Someren GR Sh 56 (EA).

***Parkinsonia aculeata* L.** Habit: Tree. Habitat: Semi-dry vegetation, ca. 100 m. Voucher: SAJIT–005423 (EA, HIB): Cultivated.

***Parkinsonia anacantha* Brenan** Habit: Shrub. Habitat: Semi-desert scrub with *Acacia mellifera*, ca. 300 m. Voucher: LC Edwards 168 (EA).

***Philenoptera bussei* (Harms) Schrire** Habit: Tree. Habitat: In riverine vegetation and mixed deciduous woodland, ca. 0–200 m. Vouchers: Ngumbau V & Mwadime N V0506 (EA, HIB), Magogo FC & Glover PE 656, Luke Q 1510 (EA).

***Piliostigma thonningii* (Schumach.) Milne-Redh.** Habit: Tree. Habitat: Woodland, wooded grassland, and bushland, 5–227 m. Vouchers: SAJIT–006105 & 005451, Ngumbau V & Mwadime N V0280 (EA, HIB), Magogo FC & Glover PE 652 (EA).

***Pithecellobium dulce* (Roxb.) Benth.** Habit: Tree. Habitat: Coastal thickets, 0–500 m. Voucher: SAJIT–006121 (EA, HIB): Cultivated.

***Prioria msoo* (Harms) Breteler** Habit: Tree. Habitat: Ground water forest, ca. 95 m. Vouchers: Ngumbau V & Mwadime N V0484 (EA, HIB), Katende AB 1759 (EA): Vulnerable.

***Prosopis juliflora* (Sw.) DC.** Habit: Shrub. Habitat: *Acacia*-*Commiphora* dry bushland, ca. 100 m. Vouchers: Johnsson S et al. 154, E Mwangi 38 (EA): Cultivated.

***Platycelyphium voense* (Engl.) Wild** Habit: Tree. Habitat: Scattered in *Acacia*-*Commiphora* deciduous bushland, ca. 80 m. Voucher: Drummond & Hemsley 4132 (EA).

***Pseudarthria hookeri* Wight & Arn.** Habit: Herb. Habitat: Woodland, ca. 227 m. Vouchers: Ngumbau V & Mwadime N V0284 (EA, HIB), Magogo FC & Glover PE 1115 (EA).

**Pseudoeriosema borianii subsp. longipedunculatum Verdc.** Habit: Herb. Habitat: Bushland, grassland, and seasonal swampy grasslands, 45–300 m. Voucher: Rawlins 838 (EA).

**Pseudoprosopis euryphylla subsp. puguensis Brenan** Habit: Liana. Habitat: Forest, ca. 30–120 m. Vouchers: Luke WQR 3466, Robertson 6232 & 5808, Nyange M & Luke Q 0591 (EA).

***Pseudovigna argentea* (Willd.) Verdc.** Habit: Climber. Habitat: Grassland, 0–45 m. Vouchers: Ngumbau V & Mwadime N V0242 (EA, HIB), Irwin 257, RM Graham in FD 1907, Jeffery 241, Magogo FC & Glover PE 883 (EA).

***Rhynchosia albissima* Gand.** Habit: Herb. Habitat: Grassland, 0–200 m. Voucher: Drummond & Hemsley 3763 (EA).

**Rhynchosia congensis subsp. orientalis Verdc.** Habit: Climber. Habitat: Coastal grassland and bushland, 0–45(–780) m. Vouchers: SAJIT–006108 (EA, HIB), RM Graham in FD 1595, Tweedie 1051, Graham MD 2111 (EA).

***Rhynchosia densiflora* (Roth) DC.** Habit: Climber. Habitat: Forest, 20–75 m. Voucher: Ngumbau V & Mwadime N V0125 (EA, HIB).

***Rhynchosia hirta* (Andrews) Meikle & Verdc.** Habit: Climber. Habitat: Grassland, scrub, woodland, and evergreen forest margins, ca. 280 m. Vouchers: Ngumbau V & Mwadime N V0254 (EA, HIB), Magogo FC & Glover PE 446 (EA).

***Rhynchosia micrantha* Harms** Habit: Herb. Habitat: Grassland, bushland, and abandoned cultivations, ca. 5 m. Vouchers: SAJIT–006101 (EA, HIB), Bogdan 4731, Bogdan 2600, Thorold 1578 (EA).

**Rhynchosia minima (L.) DC. var. minima** Habit: Climber. Habitat: Bushland, *Acacia* scrub, and grassland particularly in coastal areas, ca. 0–200 m. Vouchers: Symes 165, Rawlins 887 & 76, Luke WRQ & PA 6018 (EA).

**Rhynchosia minima var. nuda (DC.) Kuntze** Habit: Climber. Habitat: Grassland, bushland, and dry *Acacia* scrub, ca. 350 m. Vouchers: Drummond & Hemsley 4136, Magogo FC & Glover PE 992 (EA).

***Rhynchosia sublobata* (Schumach.) Meikle** Habit: Climber. Habitat: Forest, 10–600 m. Vouchers: Ngumbau V & Mwadime N V0208 (EA, HIB), Nyange M 326 (EA).

**Rhynchosia velutina var. discolor (Baker) Verdc.** Habit: Climber. Habitat: Bushland and *Brachystegia* woodland, 0–30 m. Vouchers: Rawlins 860, Jeffery 843 (EA).

**Rhynchosia velutina Wight & Arn. var. velutina** Habit: Climber. Habitat: Bushland near coast, sandy beaches and dunes, coral rocks, 0–30 (–360) m. Vouchers: SAJIT–006109 (EA, HIB), Drummond & Hemsley 1021, Rawlins 876, Bogdan 4695, Luke Q 1443, Magogo FC & Glover PE 867 (EA).

**Rhynchosia viscosa (Roth) DC. var. viscosa** Habit: Climber. Habitat: Coarse grassland and marshy places, near sea level. Vouchers: Ngumbau V & Mwadime N V0244 (EA, HIB), Thorold 1553, Rawlins 884, Whyte (EA).

**Rhynchosia viscosa var. breviracemosa (Hauman) Verdc.** Habit: Climber. Habitat: Forest edges, bushland, grassland, and cultivated land, 0–200 m. Vouchers: RM Graham in FD 2027, Tweedie 2205, Jeffery 236, Kirika P, Mbale M & Mbatha M 771 (EA).

***Rothia hirsuta* (Guill. & Perr.) Baker** Habit: Herb. Habitat: Cultivated ground and disturbed places, ca. 30 m. Vouchers: Bogdan 2624, Festo L, Luke Q & P 2684, Robertson SA 3312 (EA).

***Samanea saman* (Jacq.) Merr.** Habit: Tree. Habitat: Forest. Voucher: Robertson SA & Luke Q 5425 (EA): Naturalized.

***Scorodophloeus fischeri* (Taub.) J. Léonard** Habit: Tree. Habitat: Lowland dry evergreen forest and riverine forest, 30–670 m. Vouchers: Wakefield, Luke WRQ & Robertson SA 214 (EA).

***Senegalia adenocalyx* (Brenan & Exell) Kyal. & Boatwr.** Habit: Shrub. Habitat: Bushland and secondary thickets, 45–450 m. Vouchers: Jeffery K311 & Battiscombe 781, Jeffery K495, Kuchar P 13518 (EA).

***Senegalia brevispica* (Harms) Seigler & Ebinger** Habit: Shrub. Habitat: Grassland, ca. 50–400 m. Voucher: Gerhardt K & Steiner M 78 (EA).

***Senegalia goetzei* (Harms) Kyal. & Boatwr.** Habit: Tree. Habitat: Wooded grassland and woodland, ca. 18 m. Voucher: Luke WRQ & Robertson SA 5546 (EA).

**Senegalia mellifera (Vahl) Seigler & Ebinger subsp. mellifera** Habit: Tree. Habitat: Dry scrub and deciduous bushland, ca. 300 m. Vouchers: Jeffery K490, Robertson SA 4235, Gillett JB 20351 (EA).

**Senegalia mellifera subsp. detinens (Burch.) Kyal. & Boatwr.** Habit: Tree. Habitat: Deciduous bushland and woodland, ca. 300 m. Voucher: Geffery GM 362 (EA).

***Senegalia pentagona* (Schumach.) Kyal. & Boatwr.** Habit: Liana. Habitat: Evergreen forest, ca. 400 m. Vouchers: Verdcourt B 2122 (EA), Luke WRQ & Robertson SA sr.

**Senegalia polyacantha subsp. campylacantha (Hochst. ex. A. Rich.) Kyal. & Boatwr.** Habit: Tree. Habitat: In wooded grassland and woodland, ca. 213 m. Voucher: Unknown 69 (EA).

***Senegalia rovumae* (Oliv.) Kyal. & Boatwr.** Habit: Tree. Habitat: Riverine forest and saline water swamp forest, 6–700 m. Vouchers: Greenway & Rawlins 9432, Luke WRQ & Robertson SA 1453 (EA).

**Senegalia senegal (L.) Britton var. senegal** Habit: Tree. Habitat: Wooded grassland, ca. 120 m. Vouchers: Elliott CW 1373, Verdcourt B 1916 (EA).

**Senegalia senegal var. kerensis (Schweinf.) Kyal. & Boatwr.** Habit: Tree. Habitat: Wooded grassland, ca. 243 m. Voucher: King JM 15 (EA).

***Senna alexandrina var. obtusata* (Brenan) Lock** Habit: Shrub. Habitat: Bush on sand, ca. 10 m. Voucher: Jeffery 329 (EA).

***Senna longiracemosa* (Vatke) Lock** Habit: Shrub. Habitat: Dry scrub, deciduous bushland, and semi desert scrub, ca. 300 m. Voucher: Drummond & Hemsley 4071 (EA).

***Senna obtusifolia* (L.) H.S. Irwin & Barneby** Habit: Herb. Habitat: Grassland, roadsides, and on waste ground, ca. 100 m. Vouchers: RM Graham DD 774 in FH 2285 & in CM 1362 (EA).

***Senna occidentalis* (L.) Link** Habit: Herb. Habitat: Waste places and disturbed area, ca. 100 m. Voucher: Luke WRQ & PA 5729 (EA).

***Senna siamea* (Lam.) H. S. Irwin & Barneby** Habit: Shrubby tree. Habitat: forest, ca. 50 m. Vouchers: Perdue & Kibuwa 10168, P Kuchar 13401 (EA): Cultivated.

***Senna singueana* (Delile) Lock** Habit: Shrub. Habitat: Woodland, wooded grassland, and bushland, ca. 150 m. Vouchers: Drummond & Hemsley 1149, Klotzli F 422, Magogo FC & Glover PE 268 (EA).

***Senna timoriensis* (DC.) H.S. Irwin & Barneby** Habit: Shrub. Habitat: Open forest and lowland, ca. 50 m. Voucher: Mrs Robertson SA 7768 (EA).

***Sesbania bispinosa* (Jacq.) W. Wight** Habit: Herb. Habitat: Marshes, ditches, a weed in cultivation area and in rice fields, 0–300 m. Vouchers: Hildebrandt 1990, RM Graham in FD 2107, Rawlins 305, Kirika P 204 (EA).

***Sesbania sericea* (Willd.) Link** Habit: Herb. Habitat: Marshy ground and old rice fields, 0–850 m. Vouchers: Jeffery 486, Sampson 60 (EA).

***Sesbania speciosa* Taub.** Habit: Herb. Habitat: Swamps, ditches, and riverbanks, ca. 100 m. Vouchers: Sampson 56, Gardner in FD 1368 (EA): Vulnerable.

***Sophora inhambanensis* Klotzsch** Habit: Shrub. Habitat: Sandy foreshores, ca. 10 m. Vouchers: RM Graham 274, CW Elliot in FD 1375, Greenway & Rawlins 8917 (EA).

***Sophora tomentosa* L.** Habit: Shrub. Habitat: Sandy foreshores. Vouchers: Thorold 1550, Napier in CM 6305 (EA).

***Spathionema kilimandscharicum* Taub.** Habit: Climber. Habitat: Bushland, ca. 270 m. Vouchers: Ngumbau V & Mwadime N V0442 (EA, HIB), Luke WRQ & Robertson SA 2192 (EA).

***Sphenostylis stenocarpa* (A. Rich.) Harms** Habit: Climber. Habitat: Grassland, wooded grassland, and woodland, ca. 183 m. Vouchers: Ngumbau V & Mwadime N V0205 (EA, HIB), R RM Graham in FD 1936 (EA).

***Stylosanthes erecta* P. Beauv.** Habit: Herb. Habitat: Sandy places near the coast usually just above loose dunes, sea level. Vouchers: D Davies 78, Bogdan 2525, Greenway 9492 (EA).

***Stylosanthes fruticosa* (Retz.) Alston** Habit: Herb. Habitat: Grassland and bushland, ca. 137 m. Vouchers: Drummond & Hemsley 3942, Magogo FC & Glover PE 95, Kirika P 941 (EA).

***Tamarindus indica* L.** Habit: Tree. Habitat: Woodland, wooded grassland and deciduous bushland, ca. 297 m. Vouchers: Ngumbau V & Mwadime N V0155 (EA, HIB), Bogdan 2556, Magogo FC & Glover PE 884 (EA): Naturalized.

***Tephrosia linearis* (Willd.) Pers.** Habit: Herb. Habitat: Grassland, rocky slopes, cultivation, and fallows, ca. 186 m. Vouchers: Ngumbau V & Mwadime N V0344 (EA, HIB), Patterson 40, Luke WRQ 3311 (EA).

***Tephrosia noctiflora* Baker** Habit: Herb. Habitat: Grassland, cultivation, and thickets, ca. 213 m. Vouchers: Ngumbau V & Mwadime N V0329 (EA, HIB), Jeffery 271, Thorold 1556, Luke WRQ & PA 5722B (EA).

***Tephrosia pentaphylla* (Roxb.) G. Don** Habit: Herb. Habitat: Black cotton soil and gravelly hillsides, 0–300 m. Vouchers: Wakefield, Rawlins 733, Magogo FC & Glover PE 988 (EA).

**Tephrosia pumila (Lam.) Pers. var. pumila** Habit: Herb. Habitat: Short grassland, *Acacia*-*Commiphora* bushland, and in cultivated land, ca. 30 m. Vouchers: Tweedie 1030, Magogo FC & Glover PE 931 (EA).

**Tephrosia pumila var. aldabrensis (J.R. Drumm. & Hemsl.) Bosman & A.J.P. de Haas** Habit: Herb. Habitat: Bushland and grassland, near the sea. Vouchers: Verdcourt 2138, Greenway 10457, Rawlins 17 (EA).

**Tephrosia purpurea subsp. dunensis Brummitt** Habit: Herb. Habitat: Sandy sea and lake shores. Vouchers: Hacker 77, Greenway & Rawlins 8923 & 8914 (EA).

**Tephrosia purpurea subsp. leptostachya (DC.) Brummitt** Habit: Herb. Habitat: Grassland and plantations, ca. 297 m. Vouchers: SAJIT–004653, Ngumbau V & Mwadime N V0148 (EA, HIB), Thorold 1534, RM Graham in FD 1789, Jeffery 459, Magogo FC & Glover PE 889 (EA).

**Tephrosia stormsii De Wild. var. stormsii** Habit: Herb. Habitat: Grassland, cultivated land, and deciduous woodland, ca. 0–100 m. Vouchers: Butler 25, MacNaughton 53, Thorold 1579, Luke WRQ et al. 6194 (EA).

**Tephrosia villosa subsp. ehrenbergiana (Schweinf.) Brummitt** Habit: Herb. Habitat: *Acacia*-*Commiphora* bushland, ca. 181 m. Vouchers: Ngumbau V & Mwadime N V0227 (EA, HIB), Napier 3263 (EA).

***Tephrosia vogelii* Hook.f.** Habit: Herb. Habitat: Grassland, forest margins, waste ground, and old cultivation, ca. 50 m. Voucher: Donald in FD 2369 (EA).

**Teramnus labialis subsp. arabicus Verdc.** Habit: Climber. Habitat: Grassland and bushland, ca. 30 m. Vouchers: Whyte, Agnew ADQ 9763 (EA).

**Teramnus repens subsp. gracilis (Chiov.) Verdc.** Habit: Herb. Habitat: Bushland, 0–50 m. Vouchers: Thorold 1574, Tweedie 1015, Greenway & Rawlins 9355 (EA).

***Tetrapleura tetraptera* (Schum. & Thonn.) Taub.** Habit: Tree. Habitat: Lowland rainforest, ca. 80 m. Voucher: Drummond & Hemsley 3960 (EA).

***Vachellia bussei* (Harms ex Y. Sjöstedt) Kyal. & Boatwr.** Habit: Tree. Habitat: Scrubland, bushland, ca. 50–400 m. Vouchers: Bally PRO 8553, Robertson SA 6402 (EA).

**Vachellia etbaica subsp. platycarpa (Brenan) Kyal. & Boatwr.** Habit: Tree. Habitat: Deciduous bushland, dry scrub, and grassland, ca. 350 m. Vouchers: Robertson SA 4120, Drummond & Hemsley 4114 (EA).

**Vachellia etbaica subsp. uncinata (Brenan) Kyal. & Boatwr.** Habit: Tree. Habitat: Wooded grassland, ca. 60 m. Vouchers: Hornby 3108, Graham RM 226, Moomaw JC 1412 (EA).

**Vachellia nilotica subsp. indica (Benth.) Kyal. & Boatwr.** Habit: Tree. Habitat: Cultivated area, ca. 5 m. Voucher: Mrs Robertson SA 7744 (EA).

**Vachellia nilotica subsp. leiocarpa (Brenan) Kyal. & Boatwr.** Habit: Tree. Habitat: Grassland near the coast and woodland, ca. 100 m. Vouchers: Bogdan 2612, Dale 3832, Mrs J Adamson 437 in Bally 6136 (EA).

**Vachellia nilotica subsp. subalata (Vatke) Kyal. & Boatwr.** Habit: Tree. Habitat: Wooded grassland and deciduous bushland, ca. 15 m. Vouchers: Magogo FC & Glover PE 863, Robertson SA 4968 (EA).

**Vachellia robusta subsp. usambarensis (Taub.) Kyal. & Boatwr.** Habit: Tree. Habitat: Woodland, 10–250 m. Voucher: Robertson SA 4379 (EA).

**Vachellia sieberiana (DC.) Kyal. & Boatwr. var. sieberiana** Habit: Tree. Habitat: Deciduous woodland, wooded grassland, and riverine forest, ca. 153 m. Vouchers: Ngumbau V & Mwadime N V0510 (EA, HIB), RM Graham 2281, Luke WRQ 3158 (EA).

***Vachellia stuhlmannii* (Taub.) Kyal. & Boatwr.** Habit: Shrub. Habitat: Woodland and grassland, 100–750 m. Voucher: Robertson SA & Luke Q 5624 (EA).

**Vachellia tortilis subsp. raddiana (Savi) Kyal. & Boatw** Habit: Tree. Habitat: Bushland, 50–100 m. Vouchers: RM Graham W309 in FH 1800, Greenway 9444 (EA).

**Vachellia tortilis subsp. spirocarpa (Hochst. ex. A. Rich.) Kyal. & Boatwr.** Habit: Tree. Habitat: Deciduous woodland, ca. 600 m. Voucher: Kuchar P 13716 (EA).

***Vachellia zanzibarica* (S. Moore) Kyal. & Boatwr.** Habit: Tree. Habitat: Woodland and wooded grassland, 0–460 m. Vouchers: Bogdan 3303, RM Graham L119 in FH 1620 in CM 13991, Sangai GRW 15609 (EA).

**Vigna friesiorum var. angustifolia Verdc.** Habit: Herb. Habitat: Grassland, deciduous bushland, and *Combretum* woodland, ca. 480 m. Vouchers: Tweedie 1226, Kassner 424 (EA).

***Vigna hosei* (Craib) Backer** Habit: Climber. Habitat: Grass at roadsides and wastelands, 0–200 m. Vouchers: SAJIT–006203 (EA, HIB), Whyte, Magogo FC & Estes R 1159 (EA).

***Vigna macrorhyncha* (Harms) Milne-Redh.** Habit: Climber. Habitat: Grassland, bushland, woodland, and forest, ca. 30 m. Vouchers: Napier 3363 in CM 6308, Meester V 92, Robertson SA 3356 (EA).

**Vigna membranacea subsp. caesia (Chiov.) Verdc.** Habit: Climber. Habitat: *Acacia*-*Commiphora* bushland, coastal mixed bushland, ca. 0–400 m. Vouchers: Polhill & Paulo 718, Jeffery GW 407, Gillett JB 16870 (EA).

**Vigna membranacea subsp. hapalantha (Harms) Verdc.** Habit: Climber. Habitat: Coastal grassland in thicket, ca. 15 m. Vouchers: Jex-Blake in Napier 2276, Polhill & Paulo 877, Tweedie 940, Gillet JB 20324 (EA): Endemic.

***Vigna reticulata* Hook.f.** Habit: Climber. Habitat: Grassland, bushland, usually on damp or swampy ground, ca. 0–700 m. Vouchers: RM Graham in FD 1905, Tweedie 1061, Jeffery GW 297 (EA).

**Vigna unguiculata subsp. dekindtiana (Harms) Verdc.** Habit: Climber. Habitat: Grassland, swamp, riverine forest, and dry scrub, ca. 0–300 m. Vouchers: Napier 3366 in CM 6309, RM Graham in FD 2101, Power 10969 (EA).

**Vigna unguiculata subsp. pubescens (R. Wilczek) Pasquet** Habit: Climber. Habitat: Bushland, grassland, and grassland with scattered trees, ca. 50 m. Vouchers: Tweedie 2223, Jeffery 57, Sampson 52 (EA).

**Vigna unguiculata var. spontanea (Schweinf.) Pasquet** Habit: Climber. Habitat: Bushland, 100 m. Voucher: Magogo FC & Glover PE 471 (EA).

**Vigna vexillata (L.) A. Rich. var. vexillata** Habit: Climber. Habitat: Grassland, grassland with scattered trees, and thicket, ca. 70 m. Vouchers: Brathay 1982 Expedn 35, Soest LV 193 (EA).

**Vigna vexillata var. angustifolia (Schumach. & Thonn.) Baker** Habit: Climber. Habitat: Grassland, ca. 500 m. Voucher: Magogo FC & Glover PE 994 (EA).

***Wajira praecox* (Verdc.) Thulin & Lavin** Habit: Climber. Habitat: Dry *Acacia*, *Terminalia*-*Commiphora* bushland and semi-desert associations, ca. 375 m. Vouchers: Greenway 9821, Luke WRQ & Robertson SA 2536 (EA).

***Xeroderris stuhlmannii* (Taub.) Mendonça & E. P. Sousa** Habit: Tree. Habitat: Deciduous woodland and bushland, ca. 100 m. Vouchers: Kassner 158, Dale in FD 3843 (EA).

***Zornia apiculata* Milne-Redh.** Habit: Herb. Habitat: Forest in dry scrub, grasslands, and rocky places, ca. 20 m. Vouchers: Jeffery 231, Festo 2547 (EA).

**Zornia capensis subsp. tropica Milne-Redh.** Habit: Herb. Habitat: Dry scrub and cultivated ground, ca. 0–350 m. Vouchers: Drummond & Hemsley 4145, Rawlins in EAH 12068 (EA).

***Zornia glochidiata* Rchb. ex DC.** Habit: Herb. Habitat: Grassland, rocky places, often cultivated ground or roadsides, ca. 50 m. Voucher: Jeffery 845 (EA).

**Zornia pratensis Milne-Redh. subsp. pratensis** Habit: Herb. Habitat: Grassland on hillsides, ca. 400 m. Voucher: Drummond & Hemsley 1113 (EA).

**F81. Flagellariaceae**

1 Genus, 1 Species

***Flagellaria guineensis* Schumach.** Habit: Climber. Habitat: Forest fringes and thicket, 0–400 m. Vouchers: SAJIT–005932, Ngumbau V & Mwadime N V0247 (EA, HIB), Magogo FC & Glover PE 128 (EA).

**F82. Francoaceae**

1 Genus, 1 Species

**Bersama abyssinica Fresen. subsp. abyssinica** Habit: Tree. Habitat: Lowland bushland, savannah, and gallery forests, ca. 246 m. Voucher: Luke WRQ & Robertson SA 2724 (EA).

**F83. Gelsemiaceae**

1 Genus, 2 Species

***Mostuea brunonis* Didr.** Habit: Shrub. Habitat: Lowland rainforest and riverine forest, ca. 86 m. Vouchers: SAJIT–006075, Ngumbau V & Mwadime N V0468 (EA, HIB), Luke WRQ 889, Luke Q 1554, Drummond & Hemsley 3805, RM Graham & Dale 3809 (EA).

***Mostuea microphylla* Gilg** Habit: Shrub. Habitat: Coastal evergreen bushland and lowland rainforest, 0–350 m. Vouchers: Ngumbau V & Mwadime N V0493 (EA, HIB), WE Taylor & Bally 5824, Luke Q 1496 (EA).

**F84. Gentianaceae**

4 Genera, 5 Species

***Canscora alata* (Roth) Wall.** Habit: Herb. Habitat: Streamside on wet mud, 0–600 m. Voucher: Luke WRQ 3316 (EA).

**Enicostema axillare (Poir. ex Lam.) A. Raynal subsp. axillare** Habit: Herb. Habitat: Moist or swampy sites in grassland, lake shores, and sparse woodland, ca. 200 m. Vouchers: Magogo FC & Glover PE 1049, Greenway & Rawlins 9453, Luke 6358 (EA).

**Enicostema axillare subsp. latilobum (N. E. Br.) A. Raynal** Habit: Herb. Habitat: Grassland, 0–550 m. Vouchers: Greenway 4968, Magogo & Glover 1049 (EA).

***Exacum oldenlandioides* (S. Moore) Klack.** Habit: Herb. Habitat: Moist sites grassland on river banks, ca. 54 m. Vouchers: Ngumbau V & Mwadime N V0554 (EA, HIB), Magogo FC & Glover PE 357 (EA).

***Exochaenium platypterum* (Baker) Schinz** Habit: Herb. Habitat: Damp sites woodland or grassland, ca. 300 m. Voucher: Luke WRQ et al. 7455 (EA).

***Exochaenium pumilum* (Baker) A. W. Hill** Habit: Herb. Habitat: Damp and often sandy sites in grassland or woodland, ca. 450 m. Voucher: Luke & Luke 5996 (EA).

**F85. Gesneriaceae**

1 Genus, 2 Species

**Streptocarpus ionantha subsp. rupicola (B. L. Burtt) Christenh.** Habit: Herb. Habitat: Rocky moist areas, ca. 200 m. Vouchers: Ngumbau V & Mwadime N V0408 (EA, HIB), Mabberley 718, Faden & Beentje 85/30 (EA): Critically Endangered.

***Streptocarpus pallidiflorus* C.B. Clarke** Habit: Herb. Habitat: Rock crevices or on banks in forest or unshaded areas, rarely epiphytic, 750 m. Vouchers: Luke & Robertson 20152, Luke 4705 (EA).

**F86. Gisekiaceae**

1 Genus, 2 Species

***Gisekia africana* (Lour.) Kuntze** Habit: Herb. Habitat: Open bushland and sand dunes. Vouchers: Rawlins SP 11578, Greenway & Rawlins 9291, Rawlins 155 (EA).

**Gisekia pharnaceoides L. var. pharnaceoides** Habit: Herb. Habitat: Grassland, bushland. Vouchers: Muchiri J 490, Napier 3395, Jeffery K190, GRW Sangai 951 (EA).

**F87. Goodeniaceae**

1 Genus, 2 Species

***Scaevola plumieri* (L.) Vahl** Habit: Shrub. Habitat: Coastal sand dunes. Vouchers: Kirika P, Muthoka P & Mbale M 762, Verdcourt 3910, Greenway 13134 (EA).

***Scaevola taccada* (Gaertn.) Roxb.** Habit: Shrub. Habitat: Strand vegetation on sand and coral rock. Vouchers: Napper 3288 & 1298, Drummond & Hemsley 1059 (EA).

**F88. Hernandiaceae**

2 Genera, 2 Species

***Gyrocarpus americanus* Jacq.** Habit: Tree. Habitat: Evergreen forest and derived secondary forest, 15–510 m. Voucher: Ngumbau V & Mwadime N V0405 (EA, HIB).

***Hernandia nymphaeifolia* (J. Presl) Kubitzki** Habit: Tree. Habitat: Sandy foreshores near high-water mark in littoral bushland, sea level. Vouchers: Drummond & Hemsley 3986, Bock in EAH 16317 (EA).

**F89. Hydrocharitaceae**

6 Genera, 8 Species

***Enhalus acoroides* (L. f.) Royle** Habit: Herb. Habitat: On sand or mud in sheltered water, up to 4 m deep. Vouchers: Frazier 912, Greenway & Rawlins 9377 (EA).

***Halophila gaudichaudii* J. Kuo** Habit: Herb. Habitat: On sand and mud in sheltered sea water, up to 2 m depth. Vouchers: Isaac 116, 100 & 21 (EA).

***Halophila ovalis* (R. Br.) Hook. f.** Habit: Herb. Habitat: On fine mud to coarse coral rubble, mid-tidal level to 12 m. Vouchers: SAJIT–006265 (EA, HIB), Isaac 66, Gillespie 317 (EA).

***Halophila stipulacea* (Forssk.) Asch.** Habit: Herb. Habitat: On sand and mud in sheltered sea water, up to about 7 m deep. Vouchers: Isaac 61, Rayner 288, Greenway & Rawlins 9325 (EA).

***Lagarosiphon cordofanus* Casp.** Habit: Herb. Habitat: Still or slow flowing freshwater, ca. 30 m. Vouchers: Polphill & Paulo 846, Festo 2714 (EA).

***Najas horrida* A. Braun ex Magnus** Habit: Herb. Habitat: Lakes, river-banks, and swamps, ca. 5 m. Voucher: Starzenski 50 (EA).

***Ottelia exserta* (Ridl.) Dandy** Habit: Herb. Habitat: Still freshwater or rarely wet mud, ca. 50 m. Vouchers: Hooper & Towsend 1205, Gillespie 339 & 340 (EA).

***Thalassia hemprichii* (Ehrenb. ex Solms) Asch.** Habit: Herb. Habitat: Fine mud to clean coral sand on reef platforms, and sublittoral flats, low water mark to 5 m depth. Vouchers: Frazier 885 & 871, Gillespie 319 (EA).

**F90. Hydroleaceae**

1 Genus, 1 Species

***Hydrolea zeylanica* (L.) Vahl** Habit: Herb. Habitat: Pond margins, streamside, open forest, and swampy area, 0–100 m. Vouchers: Ngumbau V & Mwadime N V0546 (EA, HIB), Mwadime N & Luke WRQ 2572 (EA).

**F91. Hypericaceae**

3 Genera, 3 Species

***Harungana madagascariensis* Lam. ex Poir.** Habit: Tree. Habitat: Lowland and upland rainforest, ca. 0–305 m. Vouchers: SAJIT–006167 & 005944, Ngumbau V & Mwadime N V0293 (EA, HIB), Luke WRQ & Robertson SA 222 (EA).

***Psorospermum febrifugum* Spach** Habit: Shrub. Habitat: Deciduous woodland and wooded grassland, ca. 50 m. Vouchers: Luke WRQ & Robertson SA 225, Donald 36 (EA).

***Vismia orientalis* Engl.** Habit: Tree. Habitat: Forest, ca. 229 m. Vouchers: SAJIT–006046, Ngumbau V & Mwadime N V0162 (EA, HIB), Luke WRQ & Robertson SA 519, Graham 2119 (EA).

**F92. Hypoxidaceae**

2 Genera, 2 Species

**Curculigo pilosa subsp. major (Baker) Wiland** Habit: Herb. Habitat: Grassland and woodland, ca. 350 m. Voucher: Magogo FC & Glover PE 469 (EA).

**Hypoxis angustifolia Lam. var. angustifolia** Habit: Herb. Habitat: Grassland and open woodland, ca. 136 m. Vouchers: SAJIT–005946 (EA, HIB), Magogo FC & Glover PE 211 (EA).

**Hypoxis angustifolia var. luzuloides (Robyns & Tournay) Wiland** Habit: Herb. Habitat: Grassland, swampy grassland, scattered tree grassland, and secondary grassland, ca. 445 m. Voucher: Magogo & Glover 498 (EA).

**F93. Icacinaceae**

2 Genera, 4 Species

***Iodes usambarensis* Sleumer** Habit: Liana. Habitat: Edges of lowland rainforest, sometimes persisting on cultivated ground, below 100 m. Vouchers: SAJIT–006221 (EA, HIB), Luke Q 1406, Rawlins 205 (EA).

***Pyrenacantha kaurabassana* Baill.** Habit: Climber. Habitat: Dry scrub and bushland to deciduous woodland, ca. 65 m. Vouchers: SAJIT–006438 (EA, HIB), Mwangangi OM 1299, Drummond & Hemsley 1023 (EA).

**Pyrenacantha malvifolia Engl. var. malvifolia** Habit: Liana. Habitat: Bushland. Voucher: Moomaw JC 1001 (EA).

***Pyrenacantha vogeliana* Baill.** Habit: Climber. Habitat: Moist forest, ca. 249 m. Vouchers: Ngumbau V & Mwadime N V001 (EA, HIB), Robertson SA & Luke WRQ 5162 (EA).

**F94. Iridaceae**

2 Genera, 2 Species

**Gladiolus dalenii subsp. andongensis (Baker) Goldblatt** Habit: Herb. Habitat: Grassland and light woodland, ca. 300 m. Voucher: Magogo FC & Glover PE 581 (EA).

***Afrosolen schimperi* (Aschers. & Klatt) Goldblatt & J. C. Manning** Habit: Herb. Habitat: Moist habitats, along streams, and seasonal marshes, ca. 20 m. Vouchers: Battiscombe 231, JB Gillespie 176 (EA).

**F95. Kewaceae**

1 Genus, 1 Species

***Kewa bowkeriana* (Sond.) Christenhusz** Habit: Herb. Habitat: Saline sandy maritime inland soils. Vouchers: Festo L & Luke Q 2585, Greenway & Rawlins 8912 (EA).

**F96. Lamiaceae**

18 Genera, 67 Species

***Aeollanthus zanzibaricus* S. Moore** Habit: Herb. Habitat: Rocks or shallow soil overlying rocks, 200–500 m. Vouchers: Festo L, Luke Q & P 2631, Magogo FC & Glover PE 903 (EA).

***Basilicum polystachyon* (L.) Moench** Habit: Herb. Habitat: Waterside and seasonally flooded areas, ca. 300 m. Vouchers: Mutangah 38, RB & AJ Faden 74/1279 (EA).

**Clerodendrum cephalanthum Oliv. subsp. cephalanthum** Habit: Shrub. Habitat: Coastal bushland, 0–330 m. Vouchers: Ngumbau V & Mwadime N V0418 (EA, HIB), Mwadime N 14, Magogo & Estes 1223, Drummond & Hemsley 3879 (EA).

***Clerodendrum hildebrandtii* Vatke var. fischeri (Gürke) Verdc.** Habit: Shrub. Habitat: Wooded grassland, secondary bush, 5–230 m. Vouchers: Magogo FC & Glover PE 1005, Festo L & Luke Q 2456 (EA).

**Clerodendrum robustum Klotzsch var. robustum** Habit: Shrub. Habitat: Wet grasslands, woodland, and forest clearings, 0–450 m. Vouchers: Jeffrey K140, Maggridge 138, Battscombe 50 & Gardener in FD 1454, Luke WRQ et al. 6189 (EA).

***Clerodendrum tricholobum* Gürke** Habit: Shrub. Habitat: Riverine bushland, woodland, and grassland mosaic, 0–300 m. Vouchers: J Adamson 312, Robertson & Luke 5617, Whyte (EA).

***Endostemon albus* A. J. Paton, Harley & M. M. Harley** Habit: Herb. Habitat: Grassy places, streamside in coastal, and deciduous bushland, ca. 15–350 m. Vouchers: Luke WRQ & PA 6021, PA & WRQ Luke 6021, Drummond & Hemsley 4172 (EA).

***Endostemon ctenoneurus* Harley** Habit: Herb. Habitat: Bushland, 0–400 m. Voucher: Festos L & Luke Q 2556 (EA).

***Endostemon gracilis* (Benth.) M. Ashby** Habit: Herb. Habitat: *Acacia*-*Commiphora* bushland, *Brachystegia* woodland, and cultivations, 0–350 m. Vouchers: SAJIT–006135 (EA, HIB), Kirika P, Muthoka P & Mbale M 740 (EA).

***Endostemon tereticaulis* (Poir.) M. Ashby** Habit: Herb. Habitat: Short grassland, coastal, and *Acacia*-*Commiphora* bushland, ca. 30 m. Vouchers: Magogo FC & Glover PE 687, Greenway 9486 (EA).

***Endostemon wakefieldii* (Baker) M. Ashby** Habit: Herb. Habitat: *Brachystegia* woodland, ca. 100 m. Voucher: Graham 1739 (EA): Endemic.

***Hoslundia opposita* Vahl** Habit: Shrub. Habitat: Open areas in grassland, woodland or forest, ca. 100 m. Vouchers: Gillett JB 20412, Magogo FC & Glover PE 176 (EA).

***Karomia gigas* (Faden) Verdc.** Habit: Tree. Habitat: Relict forest on limestone rocks, ca. 140 m. Vouchers: Faden et al. 77/439, Gillett 22775 (EA).

**Leonotis nepetifolia (L.) R.Br. var. nepetifolia** Habit: Shrub. Habitat: Grassland, bushland, and disturbed places. Voucher: Robertson SA 4974 (EA).

***Leucas glabrata* (Vahl) J. C. Manning & Goldblatt** Habit: Herb. Habitat: Damp ground in riverine, ca. 30 m. Voucher: Luke et al. TPR 501 (EA).

***Leucas martinicensis* (Jacq.) R.Br.** Habit: Herb. Habitat: Grassland and bushland, ca. 382 m. Vouchers: Ngumbau V & Mwadime N V0370 (EA, HIB), Graham RM 2044 (EA).

***Leucas nubica* Benth.** Habit: Herb. Habitat: Grassland, coastal or deciduous bushland, ca. 30 m. Voucher: Luke et al. TPR 550 (EA).

***Leucas tomentosa* Gürke** Habit: Shrub. Habitat: Bushland and rough grassland, ca. 200 m. Voucher: Mungai, Mutangah & Rucina 335/83 (EA).

**Leucas tsavoensis var. kilifiensis Sebald** Habit: Herb. Habitat: *Brachystegia* woodland or degraded woodland, 30–150 m. Voucher: Polhill RM & Paulo S 815 (EA): Endemic.

**Leucas urticifolia var. angustifolia Sebald** Habit: Herb. Habitat: Wooded grassland and abandoned cultivated areas, ca. 30 m. Vouchers: Luke et al. TPR 549, Brand s.n. (EA).

***Mesosphaerum pectinatum* (L.) Kuntze** Habit: Shrub. Habitat: Edges of rivers, lakes, swamps, thickets or disturbed habitats. Voucher: Luke WRQ & Robertson SA 2764 (EA).

***Mesosphaerum suaveolens* (L.) Kuntze** Habit: Herb. Habitat: Disturbed places, roadsides, and cultivated ground, ca. 30 m. Voucher: Magogo FC & Glover PE 876 (EA): Naturalized.

***Ocimum americanum* L.** Habit: Herb. Habitat: Roadsides, 0–500 m. Vouchers: Patterson GD 31, Magogo FC & Glover PE 880 (EA).

***Ocimum basilicum* L.** Habit: Herb. Habitat: Cultivated and disturbed ground, ca. 420 m. Vouchers: Bally 13279, Jeffery 208 (EA): Cultivated.

***Ocimum filamentosum* Forssk.** Habit: Herb. Habitat: *Brachystegia* woodland, *Combretum* or *Acacia*-*Commiphora* or coastal bushland, and grassland, ca. 195 m. Vouchers: Ngumbau V & Mwadime N V0457 (EA, HIB), Magogo FC & Glover PE 922 (EA).

***Ocimum fischeri* Gürke** Habit: Herb. Habitat: *Acacia*-*Commiphora* bushland, scrub, and rocky ground, 100–500 m. Voucher: Drummond & Hemsley 4123 (EA).

**Ocimum gratissimum L. var. gratissimum** Habit: Herb. Habitat: Dry riverine forest, semi-deciduous forest, ca. 30–160 m. Vouchers: Robertson SA et al. 175, Kimberly Medley 271 (EA).

***Ocimum obovatum* E. Mey. ex Benth.** Habit: Herb. Habitat: Grassland, open *Acacia* or *Brachystegia* woodland, ca. 100 m. Vouchers: Moomaw JC 1135, Ossent 73 (EA).

***Orthosiphon hanningtonii* (Baker) A.J. Paton** Habit: Herb. Habitat: *Setaria*-*Sporobolus* grassland, and *Terminalia* wooded grassland, 75–110 m. Voucher: Luke & Luke 6307 (EA).

***Orthosiphon parvifolius* Vatke** Habit: Herb. Habitat: Seasonally waterlogged grassland, ca. 35 m. Voucher: Luke Q 1488 (EA).

***Orthosiphon rubicundus* (D. Don) Benth.** Habit: Herb. Habitat: Woodland, ca. 150 m. Voucher: Luke WRQ 3138 (EA).

***Orthosiphon thymiflorus* (Roth) Sleesen** Habit: Herb. Habitat: Wooded grassland and deciduous woodland, ca. 59 m. Voucher: Ngumbau V & Mwadime N V0439 (EA, HIB).

***Platostoma africanum* P. Beauv.** Habit: Herb. Habitat: Cleared or swampy areas in open woodland, 100–400 m. Vouchers: Luke WRQ et al. 4722, Drummond & Hemsley 3877, Magogo & Glover 377, Spjut 2736 (EA).

***Plectranthus aegyptiacus* (Forssk.) C.Chr.** Habit: Herb. Habitat: *Acacia*-*Commiphora* bushland, cultivated, and disturbed ground, 0–1300 m. Voucher: Robertson SA & Luke WRQ 6052 (EA).

***Plectranthus amboinicus* (Lour.) Spreng.** Habit: Herb. Habitat: *Acacia*-*Commiphora* bushland, and wooded grassland, ca. 30 m. Voucher: Luke & Robertson 2568 (EA).

***Plectranthus auriglandulosus* A.J. Paton** Habit: Herb. Habitat: Dry coastal forest and bushland, 40–550 m. Vouchers: Luke 1585, Newton 3772, Maikweki, Mturi & Abio 605 (EA): Endemic.

***Plectranthus bojeri* (Benth.) Hedge** Habit: Herb. Habitat: Grassland, woodland, open rocky areas, and forest clearings, ca. 120 m. Vouchers: Robertson SA & Luke WRQ 6003, Robertson 6482 (EA).

***Plectranthus dupuisii* (Briq.) A. J. Paton** Habit: Herb. Habitat: Grassland or woodland, and seasonally flooded areas, 100–500 m. Voucher: Robertson & Luke 5871 (EA).

***Plectranthus flaccidus* (Vatke) Gürke** Habit: Herb. Habitat: Dunes in coastal scrub or evergreen forest, 0–150 (–450) m. Vouchers: Luke Q 5668, Verdcourt 1860, Faden 74 (EA).

***Plectranthus hadiensis* (Forssk.) Schweinf. ex Sprenger** Habit: Herb. Habitat: *Acacia*-*Commiphora* bushland, deciduous woodland, and dry evergreen bushland, ca. 800 m. Voucher: Luke WRQ & Robertson SA 2828 (EA).

***Plectranthus lasianthus* (Gürke) Vollesen** Habit: Herb. Habitat: *Acacia*-*Commiphora* bushland, 100 m. Voucher: Festo L, Luke Q & P 2637 (EA).

***Plectranthus leptophyllus* (Baker) A. J. Paton** Habit: Herb. Habitat: Openings and margins of closed evergreen forest, ca. 450 m. Voucher: Cokefield (EA).

***Plectranthus longipes* Baker** Habit: Herb. Habitat: Dry bushland or woodland, 70 m. Voucher: Robertson SA & Luke WRQ 6004 (EA).

***Plectranthus tetragonus* Gürke** Habit: Herb. Habitat: *Acacia*-*Commiphora* bushland, ca. 80 m. Voucher: Luke WRQ & PA sr.

***Plectranthus prostratus* Gürke** Habit: Herb. Habitat: *Acacia* scrub, ca. 60 m. Vouchers: Rawlins SP 766, Faden & Faden 74/1051 (EA).

***Premna chrysoclada* (Bojer) Gürke** Habit: Shrub. Habitat: Coastal *Brachystegia* woodland, scrub, and thicket, 0–450 m. Vouchers: SAJIT–005603, Robertson SA 4224, Mutanga JG & Kamau P 24 (EA).

**Premna discolor Verdc. var. discolor** Habit: Shrub. Habitat: Jurassic limestone outcrop with *Pandanus*, *Euphorbia*, *Gyrocarpus*, *Ficus* and *Cola*, ca. 260 m. Vouchers: Luke & Robertson 1886, Luke 1324 (EA).

**Premna discolor var. dianiensis Verdc.** Habit: Shrub. Habitat: Very mixed dry evergreen forests, 0–12 m. Vouchers: Friis 1211, Gillett & Kibuwa 19854, Robertson & Luke 5893 (EA).

***Premna gracillima* Verdc.** Habit: Liana. Habitat: Deciduous coastal thicket, 400–660 m. Vouchers: Ngumbau V & Mwadime N V017 (EA, HIB), Robertson SA & Luke WRQ 1820, Luke WRQ 2935 (EA).

***Premna hildebrandtii* Gürke** Habit: Shrub. Habitat: Dry coastal evergreen forest, 0–200 m. Vouchers: SAJIT–005589 (EA, HIB), Moggridge JY 97 (EA).

***Premna senensis* Klotzsch** Habit: Climber. Habitat: *Acacia* woodland, thicket, and riverine woodland, (100–) 300 m. Vouchers: Luke WRQ 3134, Magogo & Glover 530 (EA).

***Premna serratifolia* L.** Habit: Shrub. Habitat: Sandy shores, thicket, and riverine forest, 0–375 m. Vouchers: Magogo & Estes 1242, Magogo & Glover 1090 (EA).

***Premna velutina* Gürke** Habit: Shrub. Habitat: Grassland, forest, riverine woodland, 18–500 m. Vouchers: Bally 16827, Kibuwa 2458, Greenway & Rawlins 9370 (EA).

**Premna resinosa subsp. holstii (Gürke) Verdc.** Habit: Shrub. Habitat: Coastal evergreen forest and thicket on coral, 0–20 (–580) m. Vouchers: Ngumbau V & Mwadime N V0350 (EA, HIB), Gillet 18642, GW Jeffery 462, RB & AJ Faden 74/1116 (EA).

***Rotheca microphylla* (Blume) Callm. & Phillipson** Habit: Shrub. Habitat: Coastal thicket, forest scrub, and *Brachystegia*-*Hymenaea* woodland, 5–450 m. Vouchers: SAJIT–005567 (EA, HIB), Magogo FC & Glover PE 926, Gillett JB 20340, Drummond & Hemsley 3786, Musyoki & Hansen 1016, Jarman in Gillespie 70, Mwadime N 671 (EA).

***Rotheca rupicola* (Verdc.) Verdc.** Habit: Shrub. Habitat: *Acacia*-*Cordia* open bushland, ca. 160 m. Voucher: Hemming 83/111 (EA).

***Rotheca sansibarensis* (Gürke) Steane & Mabb.** Habit: Shrub. Habitat: Evergreen forest and edges, ca. 229 m. Vouchers: Ngumbau V & Mwadime N V0159 (EA, HIB), Magogo & Glover 855, Musyoki & Hansek 1037, Luke WRQ 3598 (EA).

**Tinnea aethiopica subsp. litoralis Vollesen** Habit: Shrub. Habitat: Grassland and deciduous woodland, 0–750 m. Vouchers: Ngumbau V & Mwadime N V0289 (EA, HIB), Festo L, Luke Q & P 2632, Greenway 9655 (EA).

**Tinnea aethiopica subsp. stolzii (Robyns & Lebrun) Vollesen** Habit: Shrub. Habitat: Grassland and deciduous woodland, ca. 0–100 m. Voucher: Luke & Robertson 1930 (EA).

***Vitex doniana* Sweet** Habit: Tree. Habitat: *Combretum* woodland and elephant grassland, ca. 0–36 m. Vouchers: SAJIT–006194 & 005558 (EA, HIB), RM Graham 756 in FD 2226 (EA).

***Vitex ferruginea* Schumach. & Thonn.** Habit: Tree. Habitat: Bushland and dry lowland forest, 0–570 m. Vouchers: SAJIT–005498, Ngumbau V & Mwadime N V0543 (EA, HIB), Luke WRQ 3133, Napier 3304, Polhill & Paulo 723, Rawlins (EA).

***Vitex mombassae* Vatke** Habit: Tree. Habitat: Scrub, thicket, and *Brachystegia* woodland, ca. 20–305 m. Vouchers: SAJIT–005475, Ngumbau V & Mwadime N V0297 (EA, HIB), Drummond & Hemsley 1156, Magogo & Glover 505, RB & AJ Faden 77/633 (EA).

***Vitex payos* (Lour.) Merr.** Habit: Tree. Habitat: Wooded grassland, *Acacia* woodland, and rocky outcrops, ca. 30 m. Voucher: Magogo & Glover 504 (EA).

***Vitex schliebenii* Moldenke** Habit: Shrub. Habitat: Bushland, *Acacia* scrub, thicket, and riverine, 5–500 m. Vouchers: Polhill & Paulo 649, RB & AJ Faden 74/1191, Gillespie 91 (EA).

***Vitex strickeri* Vatke & Hildebr.** Habit: Shrub. Habitat: Bushland, thickets, woodland, and forest edges, ca. 0–400 m. Vouchers: Kuchar P 13655, RM Graham 814 (EA).

***Vitex trifolia* L.** Habit: Shrub. Habitat: Open vegetation near shore, 0–20 m. Vouchers: Brenan et al. 14522, Templar 23, Jeffery 348 (EA).

***Vitex zanzibarensis* Vatke** Habit: Tree. Habitat: Deciduous coastal thicket and dry forest, 0–600 m. Vouchers: SAJIT–006091 (EA, HIB), SA Robertson & Luke 4910, Conservator of forests H65/43 (EA).

***Volkameria acerbiana* Vis.** Habit: Shrub. Habitat: *Acacia* woodland, scrub, and sand dunes, ca. 0–180 m. Vouchers: RM Graham in FD 2237, Battiscombe 229, Werner in Battiscombe 997, Festo L, Luke Q & P 2671 (EA).

***Volkameria glabra* (E. Mey.) Mabb. & Y.W. Yuan** Habit: Shrub. Habitat: Littoral sand dunes associations, coastal bushland, and evergreen thicket on coral cliffs, ca. 0–50 (–480) m. Vouchers: SAJIT–006177 (EA, HIB), Napier 3303 in CM 6237, Tweedie 2373, Gillespie 174 (EA).

**F97. Lauraceae**

1 Genus, 1 Species

***Cassytha filiformis* L.** Habit: Climber. Habitat: Bushland, scrub, mixed woodland, thickets, and forest patches, ca. 0–445 m. Vouchers: Allen JD 15123, Verdcourt 3960 (EA).

**F98. Lecythidaceae**

1 Genus, 1 Species

***Barringtonia racemosa* (L.) Spreng.** Habit: Tree. Habitat: In forest by rivers, mangroves, and bordering grassland, 0–450 (–750) m. Vouchers: Ngumbau V & Mwadime N V0394 (EA, HIB), Magogo FC & Glover PE 891 (EA).

**F99. Lentibulariaceae**

1 Genus, 5 Species

***Utricularia arenaria* A. DC.** Habit: Herb. Habitat: Boggy grassland, damp open sandy ground, and rock pavements. Vouchers: Magogo FC & Glover PE 320, Polhill & Paulo 902 (EA).

***Utricularia firmula* Welw. ex Oliv.** Habit: Herb. Habitat: Boggy grassland. Voucher: Magogo FC & Glover PE 321 (EA).

**Utricularia gibba subsp. exoleta (R.Br.) P. Taylor** Habit: Herb. Habitat: Shallow water or mud in swamps, pools, and ditches. Voucher: Rawlins in EAH 2125 (EA).

***Utricularia inflexa* Forssk.** Habit: Herb. Habitat: Still or slow flowing water in pools, lakes, and rivers, ca. 15 m. Vouchers: SAJIT–006228 (EA, HIB), Leauthaud C & Geopar 27, Drummond & Hemsley 4017 (EA).

***Utricularia stellaris* L.f.** Habit: Herb. Habitat: Still or slowly flowing water in lakes, marshes, and rivers. Voucher: Greenway 9242 (EA).

**F100. Linaceae**

1 Genus, 1 Species

***Hugonia castaneifolia* Engl.** Habit: Liana. Habitat: Dry evergreen or riverine forest, 7–150 m. Vouchers: SAJIT–006009, Ngumbau V & Mwadime N V0145 (EA, HIB), Greenway 9638 & 10839, Jeffery K300, Magogo FC & Glover PE 37 (EA).

**F101. Linderniaceae**

8 Genera, 11 Species

**Artanema longifolium var. amplexicaule Vatke** Habit: Herb. Habitat: Wet grassland, forest swamps, and plantations, 0–800 m. Vouchers: Hildebrandt 2001b, Graham 2217, Luke & Robertson 2334 (EA).

***Craterostigma hirsutum* S. Moore** Habit: Herb. Habitat: Shallow soil over rock where water runs during rains, ca. 350 m. Voucher: Drummond & Hemsley 4149 (EA).

***Craterostigma newtonii* (Engl.) Eb. Fisch., Schäferh. & Kai Müll.** Habit: Herb. Habitat: Wet grassland, marshes, banks of streams, and coasts, ca. 20 m. Voucher: Polhill & Paulo 893 (EA).

***Crepidorhopalon goetzei* (Engl.) Eb. Fisch.** Habit: Herb. Habitat: Boggy places besides streams, ca. 140 m. Voucher: Luke & Gray 4061 (EA).

***Crepidorhopalon hepperi* Eb. Fisch.** Habit: Herb. Habitat: Secondary grassland and wet forests, ca. 150 m. Vouchers: Luke 2899 & 3796, Luke & Gray 4061(EA).

***Crepidorhopalon whytei* (Skan) Eb. Fisch** Habit: Herb. Habitat: Riversides and marshy area, ca. 200 m. Voucher: Drummond & Hemsley 4007 (EA).

***Linderniella bolusii* (Hiern) Eb. Fisch., Schäferh. & Kai Müll.** Habit: Herb. Habitat: Wet, shallow, sandy soil surrounding outcrops, ca. 250 m. Vouchers: Polhill & Paulo 486, Drummond & Hemsley 4150 (EA).

***Lindernia parviflora* (Roxb.) Haines** Habit: Herb. Habitat: Sandy, muddy river, permanent pools and rice fields, ca. 27 m. Vouchers: SAJIT–005491, Ngumbau V & Mwadime N V0556 (EA, HIB), Tweedie 1039, Luke WRQ & Robertson SA 2751 (EA).

***Stemodiopsis rivae* Engl.** Habit: Herb. Habitat: In rock crevices, ca. 600 m. Voucher: Mwadime N & Luke WRQ 2428 (EA).

***Torenia thouarsii* (Cham. & Schltdl.) Kuntze** Habit: Herb. Habitat: Wet sandy soil, often shady, and by streams or pools, ca. 95 m. Vouchers: SAJIT–006140 & 005486 (EA, HIB), Graham 1704, Drummond & Hemsley 4012, Luke WRQ 1652 (EA).

***Vandellia humilis* (Bonati) Eb. Fisch., Schäferh. & Kai Müll**. Habit: Herb. Habitat: Margins of pools and water holes, rice fields, and streamside in lowland forests, 0–400 m. Vouchers: Ngumbau V & Mwadime N V0553 (EA, HIB), Drummond & Hemsley 4008, Luke & Robertson 2714, Luke 3044, Luke WRQ 2925 (EA).

**F102. Loganiaceae**

1 Genus, 12 Species

***Strychnos angolensis* Gilg** Habit: Liana. Habitat: Moist forest and riverine forest, ca. 407 m. Vouchers: SAJIT–006034 (EA, HIB), Luke WRQ 2926 (EA).

***Strychnos decussata* (Pappe) Gilg** Habit: Tree. Habitat: Dry forest and woodlands, ca. 500 m. Voucher: Luke WRQ & Robertson SA 57 (EA).

***Strychnos henningsii* Gilg** Habit: Tree. Habitat: Upland and lowland rainforest, semi-evergreen bushland, lowland dry evergreen forest, and riverine forest, 340 m. Vouchers: SAJIT–006444 (EA, HIB), Kuchar P 13523, Drummond & Hemsley 4226 (EA).

***Strychnos kasengaensis* De Wild.** Habit: Liana. Habitat: Riverine forest, ca. 200 m. Vouchers: Swynnerton R JM 44, Moreau JC 1072 (EA).

***Strychnos madagascariensis* Poir.** Habit: Tree. Habitat: Bushland and open woodland, 0–450 m. Vouchers: SAJIT–005525 (EA, HIB), MacNaughton 90 in FD 2622, Dale 3803, Drummond & Hemsley 4077, Muchiri J 441(EA).

***Strychnos madagascariensis* Poir. F. “b**” Habit: Tree. Habitat: Forest. Voucher: Robertson SA 5657 (EA).

***Strychnos madagascariensis* Poir. F. “e**” Habit: Tree. Habitat: Forest. Voucher: Ross KS 119 (EA).

***Strychnos mellodora* S. Moore** Habit: Tree. Habitat: Found in riverine and moist montane forest, ca. 400 m. Voucher: Luke WRQ & Robertson SA 2740 (EA).

***Strychnos mitis* S. Moore** Habit: Tree. Habitat: Forests and coastal bushland, ca. 20 m. Vouchers: Robertson SA & Luke WRQ 5511, Luke WRQ & Robertson SA 2100 (EA).

***Strychnos panganensis* Gilg** Habit: Liana. Habitat: Lowland rainforest and coastal evergreen bushland, 50–500 m. Vouchers: SAJIT–005488 & 004644 (EA, HIB), Luke Q 1480, Magogo FC & Glover PE 459, RM Graham 1581, Swynnerton 9, Moggridge 503 (EA).

***Strychnos scheffleri* Gilg** Habit: Liana. Habitat: Lowland rainforest, ca. 200 m. Vouchers: Ngumbau V & Mwadime N V066 (EA, HIB), Luke WRQ & Robertson SA 1415, RM Graham 2045, Drummond & Hemsley 1094, CF Elliott 327, Luke WRQ 884 (EA).

***Strychnos spinosa* Lam.** Habit: Tree. Habitat: Dry woodland and thickets, ca. 400 m. Vouchers: SAJIT–006000, Ngumbau V & Mwadime N V0216 (EA, HIB), Kuchar P 12901, Luke WRQ & Robertson SA 2791 (EA).

**Strychnos spinosa subsp. volkensii (Gilg) E.A. Bruce** Habit: Shrub. Habitat: Coastal evergreen bushland, ca. 10 m. Vouchers: Adamson J 5976, RM Graham 1770, Mrs J Adamson 285 in Bally 5976, F Thomas 198 (EA).

***Strychnos usambarensis* Gilg ex Engl**. Habit: Tree. Habitat: Lowland and upland rainforest, ca. 0–300 m. Vouchers: Sangai GW 986, Luke PA & WRQ 3565 (EA).

**F103. Lophiocarpaceae**

1 Genus, 1 Species

***Corbichonia decumbens* (Forssk.) Exell** Habit: Herb. Habitat: Woodland, cultivated, and disturbed ground, ca. 375 m. Voucher: Luke WRQ & PA 6019 (EA).

**F104. Loranthaceae**

11 Genera, 27 Species

***Agelanthus heteromorphus* (A. Rich.) Polhill & Wiens** Habit: Shrub. Habitat: Coastal and deciduous forest, ca. 150 m. Vouchers: Graham RM 1598, Luke & SA Robertson 2231 (EA).

***Agelanthus kayseri* (Engl.) Polhill & Wiens** Habit: Shrub. Habitat: Coastal bushland and mangrove stands, 0–600 m. Vouchers: Robertson, SA & Luke WRQ 5713, Wiens 452 (EA).

***Agelanthus longipes* (Baker & Sprague) Polhill & Wiens** Habit: Shrub. Habitat: Coastal forest and associated bushland, 30–650 m. Vouchers: Ngumbau V & Mwadime N V0101 (EA, HIB), Luke 4023, Magogo & Glover 968, Musyoki & Hansen 999 (EA).

***Agelanthus microphyllus* Polhill & Wiens** Habit: Shrub. Habitat: Deciduous bushland, ca. 30 m. Voucher: Moomaw 1033A (EA): Endangered.

***Agelanthus oehleri* (Engl.) Polhill & Wiens** Habit: Shrub. Habitat: Drier habitats, ca. 20 m. Voucher: Gilbert MG & Kuchar P 5928 (EA).

**Agelanthus sansibarensis (Engl.) Polhill & Wiens subsp. sansibarensis** Habit: Shrub. Habitat: Coastal bushland, mangrove swamps, and forest, ca. 181 m. Vouchers: Ngumbau V & Mwadime N V0222 (EA, HIB), Magogo & Glover 194, Verdcourt 2127, Kuchar P 12869, Magogo FC & Glover PE 61, Jeffery GW 122 (EA).

***Agelanthus subulatus* (Engl.) Polhill & Wiens** Habit: Shrub. Habitat: Forest to woodland, bushland, and wooded grassland, 10–380 m. Vouchers: Festo L & Luke Q 2560, Luke WRQ 3106, Polhill 4823, MG Gilbert & Kuchar 5853 (EA).

**Emelianthe panganensis (Engl.) Danser subsp. panganensis** Habit: Shrub. Habitat: Deciduous and coastal bushland, ca. 0–200 m. Vouchers: Robertson SA 4037, RM Graham in FD 1818 (EA).

**Emelianthe panganensis subsp. commiphorae Wiens & Polhill** Habit: Shrub. Habitat: Coastal and deciduous bushland or wooded grassland, ca. 15 m. Vouchers: SA Robertson 4037, Festo L, Luke Q & P 2799 (EA).

***Englerina heckmanniana* (Engl.) Polhill & Wiens** Habit: Shrub. Habitat: Woodland, ca. 700 m. Voucher: Rawlins SP 934 (EA).

***Englerina ramulosa* (Sprague) Polhill & Wiens** Habit: Shrub. Habitat: Coastal bushland, 0–60 m. Vouchers: Tweedie 2386, SA Robertson 3600, Gillespie 184 (EA): Endangered.

***Erianthemum alveatum* (Sprague) Danser** Habit: Shrub. Habitat: Coastal bushland, woodland, and wooded grassland, 0–300 m. Vouchers: Wiens 4532, Greenway 9791, SA Robertson & Luke 4925A (EA): Vulnerable.

***Erianthemum dregei* (Eckl. & Zeyh.) Tiegh.** Habit: Shrub. Habitat: Woodland, bushland, wooded grassland, and disturbed places, ca. 0–396 m. Vouchers: SAJIT–005598, Ngumbau V & Mwadime N V033 (EA, HIB), Robertson SA & Luke WRQ 5576, Magogo FC & Glover PE 966, Wiens 4527 (EA).

***Helixanthera kirkii* (Oliv.) Danser** Habit: Shrub. Habitat: Coastal bushland and mixed dry bushland, ca. 10 m. Vouchers: Festo L & Luke Q 2563, Polhill 4811 (EA).

***Oliverella hildebrandtii* (Engl.) Tiegh.** Habit: Shrub. Habitat: Deciduous and coastal bushland, ca. 0–30 m. Vouchers: Faden, Gillett & Gachachi 77/471, Festo L, Luke Q & P 2792, Magogo FC & Glover PE 686 (EA).

***Oncella ambigua* (Engl.) Tiegh.** Habit: Shrub. Habitat: Coastal bushland and riverine forest, 0–300 m. Vouchers: Festo L, Luke Q & P 2681, Gillett & Kibuwa 19913, Luke et al. in TPR 68, RM Graham in FD 1799 (EA).

***Oncella curviramea* (Engl.) Danser** Habit: Shrub. Habitat: Coastal forest and bushland, ca. 0–80 m. Vouchers: Robertson SA & Luke WRQ 5555, Polhill & SA Robertson 4846, Bally & Smith 14379, SA Robertson 6880 (EA).

***Oncocalyx cordifolius* Wiens & Polhill** Habit: Shrub. Habitat: Coastal bushland, on various hosts, near sea level. Vouchers: Gilbert MG 6834, SA Robertson & Luke 5559, MG Gilbert & Kuchar 5871, Buck in EA 16032 (EA).

***Oncocalyx fischeri* (Engl.) M.G. Gilbert** Habit: Shrub. Habitat: Woodland, ca. 10 m. Voucher: Festo L, Laizer G, Luke Q & P 2742 (EA).

***Oncocalyx kelleri* (Engl.) M.G. Gilbert** Habit: Shrub. Habitat: Deciduous bushland or commonly riverine, ca. 15 m. Voucher: Polhill & Paulo 500 (EA).

***Oncocalyx ugogensis* (Engl.) Wiens & Polhill** Habit: Shrub. Habitat: Deciduous bushland, ca. 15 m. Voucher: Gillett 16375 (EA).

***Plicosepalus curviflorus* (Benth. ex Oliv.) Tiegh.** Habit: Shrub. Habitat: Deciduous bushland, ca. 30 m. Voucher: Luke WRQ 5681 (EA).

***Plicosepalus kalachariensis* (Schinz) Danser** Habit: Shrub. Habitat: Mixed woodland, ca. 30 m. Voucher: Luke & SA Robertson 2368 (EA).

***Plicosepalus meridianus* (Danser) Wiens & Polhill** Habit: Shrub. Habitat: Deciduous bushland, ca. 100 m. Vouchers: RB & AJ Faden 74/1022, Luke WRQ 3513 (EA).

***Plicosepalus sagittifolius* (Engl.) Danser** Habit: Shrub. Habitat: Coastal bushland, ca. 450 m. Voucher: Karisa R9 (EA).

***Spragueanella rhamnifolia* (Engl.) Balle** Habit: Shrub. Habitat: Forest along coast or inland along rivers, ca. 30 m. Vouchers: SAJIT–004649 (EA, HIB), Verdcourt 1864, Wiens 4525.

***Taxillus wiensii* R. M. Polhill** Habit: Shrub. Habitat: Dry evergreen coastal forest, 100–150 m. Vouchers: Wiens D 4526, Polhill 4825, Luke & SA Robertson 2173, SA Robertson & Polhill 6861 (EA): Critically Endangered, Endemic.

**F105. Lythraceae**

4 Genera, 12 Species

***Ammannia auriculata* Willd.** Habit: Herb. Habitat: Mud by dams and ponds, wooded riverine flats, and damp grassland, ca. 5 m. Vouchers: F Thomas 70, Festo 2351 (EA).

**Ammannia capitellata (C. Presl) S.A. Graham & Gandhi subsp. capitellata** Habit: Herb. Habitat: Edges of permanent water or rice fields, ca. 50 m. Vouchers: Gilbert & Kuchar 588, Luke Q 5661 (EA).

**Ammannia parkeri (Verdc.) S. A. Graham & Gandhi var. parkeri** Habit: Herb. Habitat: Edges of water pans in open bushland, grassland, and flooded grassland, 30–460 m. Vouchers: Luke WRQ & Robertson SA 1550, Luke Q 1550, Luke et al. TPR 761 (EA).

**Ammannia parkeri var. longifolia (Verdc.) S. A. Graham & Gandhi** Habit: Herb. Habitat: Found in flooded grassland habitat, ca. 10 m. Vouchers: Festo L, Luke Q & P 2740, Whyte (EA): Vulnerable, Endemic.

***Ammannia pedicellata* (Hiern) S. A. Graham & Gandhi** Habit: Herb. Habitat: Seasonally flooded freshwater swamps or rice fields, 0–150 m. Vouchers: Luke Q 5634, Luke 3034, Greenway & Rawlins 9451 (EA).

***Ammannia prieuriana* Guill. & Perr.** Habit: Herb. Habitat: Sandy, bank of the river, and edge of swamp. Voucher: Luke WRQ 3606 (EA).

**Ammannia radicans (Guill. & Perr.) S. A. Graham & Gandhi var. radicans** Habit: Herb. Habitat: Swampy ground, grassy places on lava, pool edges, and rice-fields, ca. 95 m. Vouchers: SAJIT–006139 (EA, HIB), Luke WRQ 3315, SA Robertson 3512, Whyte (EA).

**Ammannia radicans var. floribunda (Sond.) S. A. Graham & Gandhi** Habit: Herb. Habitat: Marshes, swampy grassland, muddy ditches, riverbanks, and rice-fields, ca. 60 m. Vouchers: Polhill & Paulo 891, Festo L & Luke Q 2581 (EA).

***Ammannia senegalensis* Lam.** Habit: Herb. Habitat: Damp ground, seasonal freshwater short grass, and sedge swamp, 0–12 (–240) m. Vouchers: Hooper & Townsend 1127, Luke Q 5625 (EA).

***Ammannia stuhlmannii* (Koehne) S. A. Graham & Gandhi** Habit: Herb. Habitat: Open grassland, *Brachystegia* woodland, and irrigated rice fields, 15–75 m. Vouchers: Rawlins 865, Hooper & Townsend 1244, Polhill & Paulo 533, Festo L, Luke Q & P 2624 (EA).

***Ammannia urceolata* Hiern** Habit: Herb. Habitat: Floodplain grassland, ca. 75 m. Vouchers: Luke et al. in TPR 124 & 780 (EA).

***Lawsonia inermis* L**. Habit: Herb. Habitat: Temporarily flooded rocky river, riverine thicket, also near coast, ca. 30 m. Voucher: Polhill & Paulo 673 (EA).

***Pemphis acidula* J. R. Forst. & G. Forst.** Habit: Shrub. Habitat: Littoral at or even below high-water mark, coral rag thicket, and drier sides of swamp, 0–5 m. Vouchers: Kimeu JM 650, Kibuwa 1204, Williams Sangai 865, Greenway & Rawlins 8873 (EA).

***Sonneratia alba* Sm.** Habit: Tree. Habitat: Frequent in salty muds of mangrove swamps, ca. 5 m. Vouchers: SAJIT–006264 (EA, HIB), Kuchar P 13607 (EA).

**F106. Malpighiaceae**

3 Genera, 5 Species

***Acridocarpus alopecurus* Sprague** Habit: Liana. Habitat: Dry evergreen and riverine forest, 20–500 m. Vouchers: Ngumbau V & Mwadime N V090 (EA, HIB), Verdcourt 3920A, RM Graham in FD 1786, Luke Q 1408, Luke WQR et al. 4604F (EA).

***Acridocarpus zanzibaricus* A. Juss.** Habit: Shrub. Habitat: Forest, 0–180 m. Vouchers: SAJIT–005568 (EA, HIB), VG van Someren 1793, Battiscombe 801 (EA).

***Triaspis mozambica* A. Juss.** Habit: Liana. Habitat: Coastal and riverine bushland or thicket, dry evergreen forest, and lowland rainforest, 0–800 m. Vouchers: Ngumbau V & Mwadime N V0411 (EA, HIB), Drummond & Hemsley 3910, Jeffery 451, Festo L, Luke Q & P 2623 (EA).

***Triaspis niedenzuiana* Engl.** Habit: Liana. Habitat: Deciduous bushland, sometimes on stony hillsides or rock outcrops, ca. 500 m. Voucher: Kassner 533 (EA).

***Tristellateia africana* S. Moore** Habit: Liana. Habitat: Coastal bushland, thicket, and mangrove swamp, 0–50 m. Vouchers: Drummond & Hemsley 3980, VG van Someren 1791, Polhill & Paulo 688, Festo L, Luke Q & P 2725 (EA).

**F107. Malvaceae**

30 Genera, 117 Species

***Abelmoschus ficulneus* (L.) Wight & Arn.** Habit: Herb. Habitat: Grassland on clay, flood plains, riverbanks, and roadsides, ca. 20 m. Voucher: Robertson & Luke 5372 (EA).

***Abelmoschus moschatus* Medik.** Habit: Herb. Habitat: Riverine vegetation, ca. 28 m. Vouchers: SAJIT–006207 & 006208, Ngumbau V & Mwadime N V0541 (EA, HIB).

***Abutilon guineense* (Schumach.) Baker f. & Exell** Habit: Herb. Habitat: Grassland, bushland, and wooded bushland, ca. 30 m. Voucher: Kabuye et al. TPR 322 (EA).

**Abutilon mauritianum subsp. zanzibaricum (Mast.) Verdc.** Habit: Herb. Habitat: Coastal thicket, bushland, sandy grassland, in banana, and coconut plantations, 0–400 m. Vouchers: SAJIT–006114 (EA, HIB), Magogo & Glover 798, Wright 13, Jeffery 267, Magogo FC & Glover PE 302 (EA).

**Abutilon pannosum (G. Forst.) Schltdl. var. pannosum** Habit: Herb. Habitat: Bushland, 30–100 m. Voucher: Festo L, Luke Q & P 2626 (EA).

**Abutilon pannosum var. scabrum Verdc.** Habit: Herb. Habitat: Bushland and thicket, 15–105 m. Vouchers: BL Simpson 283, Thairu 18, F Thomas 155 (EA).

***Abutilon rotundifolium* Mattei** Habit: Herb. Habitat: On coral with *Commiphora*, dunes, and calcareous sandstone, 0–5 m. Vouchers: Rawlins 117, Greenway & Rawlins 9417, Gillespie 115, Luke Q 5473 (EA).

**Abutilon sp. nr. wituense Baker f.** Habit: Herb. Habitat: *Hyphaene* thicket and grassland, 30 m. Vouchers: Kabuye et al. TPR 44, 03A (EA).

***Abutilon wituense* Baker f.** Habit: Herb. Habitat: Riverine gallery forest, thicket, and dry sand rivers, 30–115 m. Vouchers: Luke & Robertson 2303, Kabuye et al. TPR 66, Thomas 10 (EA).

***Adansonia digitata* L.** Habit: Tree. Habitat: Dry forest or bushland, ca. 250 m. Vouchers: SAJIT–004671 & 005597 (EA, HIB), Kuchar P 11856 (EA).

***Rhodognaphalon schumannianum* A. Robyns** Habit: Tree. Habitat: Woodland, forest, bushland, 20–700 m. Voucher: Magogo FC & Glover PE 675 (EA).

***Byttneria fruticosa* K. Schum. ex Engl.** Habit: Tree. Habitat: Moist forest, especially riverine habitats, 10–400 m. Vouchers: Luke WRQ & Robertson SA 2706, Faden et al. 77/525, Luke 3303 (EA).

***Carpodiptera africana* Mast.** Habit: Tree. Habitat: Evergreen thicket and dry evergreen mixed forest, 0–550 m. Vouchers: Salim bin Said 135, J Adamson 305, Polhill & Paulo 757, Awanda 5 (EA).

***Christiana africana* DC.** Habit: Tree. Habitat: Forest along river banks and mangrove forest, 0–300 m. Vouchers: Luke WRQ 900, Luke 2916, Luke & Robertson 542 (EA).

***Cienfuegosia hildebrandtii* Garcke** Habit: Herb. Habitat: *Commiphora*-*Acacia*-*Thespesia* bushland, 100–450 m. Vouchers: Luke Q 1266, Q Jones 69/164, Kassner 439, Luke & Robertson 2138 (EA).

***Cola minor* Brenan** Habit: Tree. Habitat: Coastal evergreen forest or thicket, 50–250 m. Vouchers: Ngumbau V & Mwadime N V0533 (EA, HIB), Luke WRQ & PA 4500, Langridge 1/57, Verdcourt 1863, Polhill & Robertson 4830, Simpson BL 5 (EA).

***Cola octoloboides* Brenan** Habit: Tree. Habitat: Evergreen forest on limestone, 200–400 m. Vouchers: Faden et al. 70/943, Adams 125, Robertson et al. MDE 301, Robertson SA & Luke WRQ 4798 (EA): Endangered, Endemic.

***Cola porphyrantha* Brenan** Habit: Tree. Habitat: Evergreen forest, 45–950 m. Vouchers: Luke 1835, Magogo & Glover 1080, Brenan et al. 14557, Brenan JPM, Gillett JB, Kanuri K & Chomba W 14562, Luke WRQ et al. 4604C (EA): Critically Endangered.

***Cola pseudoclavata* Cheek** Habit: Tree. Habitat: Riverine or coastal evergreen or semi-deciduous forest, 30–600 m. Vouchers: Ngumbau V & Mwadime N V0524 (EA, HIB), Medley 344, Mohamed Abdullah in FD 3353, Verdcourt 1855, Greenway PJ & Rawlins SP 895 (EA).

***Cola uloloma* Brenan** Habit: Tree. Habitat: Coastal forest, 30–500 m. Vouchers: Robertson & Luke 4787, Drummond & Hemsley 3972, Luke & Robertson 494, Luke WRQ 2934 (EA).

***Corchorus aestuans* L.** Habit: Herb. Habitat: Grassland on heavy alluvial soils, ca. 10–300 m. Vouchers: Ngumbau V & Mwadime N V0278 (EA, HIB), Drummond & Hemsley 1089, Gillett 18637, Gardner 2280, Sangai GW 976 (EA).

***Corchorus baldaccii* Mattei** Habit: Herb. Habitat: *Commiphora* woodland, ca. 30 m. Voucher: Polhill & Paulo 602 (EA).

***Corchorus fascicularis* Lam.** Habit: Herb. Habitat: Seasonally flooded grassland or wooded grassland, ca. 10 m. Voucher: Luke et al. in TPR 457 (EA).

***Corchorus gillettii* Bari** Habit: Herb. Habitat: Open bushland on sand, 50–650 m. Voucher: Gillet 16335 (EA).

***Corchorus olitorius* L.** Habit: Herb. Habitat: Weed of cultivated area, 30–300 m. Voucher: Magogo FC & Glover PE 1054 (EA).

***Corchorus parviflorus* (Benth.) Domin** Habit: Herb. Habitat: Woodland, ca. 30 m. Voucher: Dale IR 1154 (EA): Exotic.

***Corchorus pseudoolitorius* Islam & Zaid** Habit: Herb. Habitat: Riverine vegetation, 10–600 m. Vouchers: Polhill & Paulo 829, Festo L & Luke Q 2449 (EA).

***Corchorus schimperi* Cufod.** Habit: Herb. Habitat: Grassland on sand or wet clay. Voucher: Mungai et al. 440/84 (EA).

***Corchorus trilocularis* L.** Habit: Herb. Habitat: Grassland, roadsides, and disturbed places, ca. 10 m. Vouchers: Festo L & Luke Q 2451, Magogo FC & Glover PE 869 (EA).

***Dombeya kirkii* Mast.** Habit: Shrub. Habitat: Forest edge, *Acacia* bushland, bushland on rocky slopes, ca. 600 m. Vouchers: Gillett 16843, Mwachala G 281 (EA).

***Dombeya taylorii* Baker f.** Habit: Tree. Habitat: Thicket or wooded grassland, lowland evergreen forest, 0–500 m. Vouchers: Ngumbau V & Mwadime N V0532 (EA, HIB), Roberston & Luke 5826, Whyte s.n., Verdcourt 1901, Graham RM 350, Robertson SA & Luke Q 5826, Gray M & Luke WRQ 273 (EA).

***Gossypioides kirkii* (Mast.) J. B. Hutch.** Habit: Liana. Habitat: Bushland, mixed coastal forest, and *Brachystegia* woodland, 0–450 m. Vouchers: SAJIT–006014, Ngumbau V & Mwadime N V0251 (EA, HIB), Verdcourt 1871, Polhill & Paulo 842, Obunyali & Omondi W 304 (EA).

***Gossypium barbadense* L.** Habit: Shrub. Habitat: Gardens and experimental plots, ca. 5 m. Voucher: Magogo & Glover 985 (EA): Cultivated.

***Gossypium herbaceum* L.** Habit: Shrub. Habitat: Woodland, ca. 10 m. Voucher: Luke Q 5486 (EA).

***Gossypium hirsutum* L.** Habit: Shrub. Habitat: Cultivated in gardens and coconut plantations, 0–150 m. Voucher: Greenway 10455 (EA): Cultivated.

***Gossypium somalense* (Gürke) J. B. Hutch., Silow & S. G. Stephens** Habit: Shrub. Habitat: *Acacia*-*Commiphora* thicket and *Acacia*-*Commiphora*-*Delonix* bushland, (300–) 400–700 m. Voucher: Gillett 21101 (EA).

***Grewia balensis* Kirkup & Sebsebe** Habit: Shrub. Habitat: *Acacia* bushland and coastal forest, 0–10 m. Vouchers: J Adamson in Bally 5973, Gillespie 211 & 218 (EA).

***Grewia calymmatosepala* K. Schum.** Habit: Shrub. Habitat: Forest and coastal forest, ca. 50 m. Vouchers: SAJIT–005934, Ngumbau V & Mwadime N V068 (EA, HIB), Drummond & Hemsley 3933 & 1123, Luke & Robertson 1925, Luke & Robertson 1925 (EA).

***Grewia capitellata* Bojer** Habit: Shrub. Habitat: Forest, forest edges, on coral rock, and sand near the shoreline, 0–500 m. Vouchers: SAJIT–005526, Ngumbau V & Mwadime N V0316 (EA, HIB), Luke & Robertson 1717 & 2716, Greenway 10842, Brenan, JPM, JH, Gillet JB et al. 14670 (EA).

***Grewia damine* Gaertn.** Habit: Shrub. Habitat: Woodland and thicket, ca. 650 m. Voucher: Kuchar P 11832 (EA).

***Grewia densa* K. Schum.** Habit: Shrub. Habitat: Forest, including riverine, and *Acacia*-*Commiphora* bushland, 0–450 m. Vouchers: Dale 2036, Luke et al. in TPR 662, Gillett & Kihuwa 19991 (EA).

***Grewia forbesii* Harv. ex Mast.** Habit: Liana. Habitat: Thicket, forest, woodland, and secondary vegetation, ca. 237 m. Vouchers: Ngumbau V & Mwadime N V0175 (EA, HIB), Robertson SA 4237, Rawlins 782 (EA).

***Grewia glandulosa* Vahl** Habit: Shrub. Habitat: Dry forest and bush on coral rag, 0– 20 m. Vouchers: SAJIT–005570 & 006119 (EA, HIB), Bridson 131, Bally & AR Smith 14382, Greenway 10453 (EA).

***Grewia holstii* Burret** Habit: Liana. Habitat: Forest, forest edges, clearings, thicket, and bushland, 0–650 m. Vouchers: SAJIT–005497, Ngumbau V & Mwadime N V052 (EA, HIB), Gillespie 202, Sjut & Ensor 2601, Drummond & Hemsley 3962, Mrs Robertson SA & Luke Q 5586 (EA).

***Grewia microcarpa* K. Schum.** Habit: Shrub. Habitat: Woodland, thicket, *Acacia*-*Commiphora* bushland, and riverine vegetation, 0–700 m. Vouchers: RM Graham 1760, RB Faden et al. 77/462, Gisau 38 (EA).

***Grewia pedunculata* K. Schum.** Habit: Shrub. Habitat: Dry bushland, wooded grassland, and dense thicket, 0–400 m. Vouchers: Drummond & Hemsley 4041, Verdcourt 5304, Luke & Robertson 1497, Robertson SA & Luke WRQ 6030, Luke Q 1497 (EA).

***Grewia plagiophylla* K. Schum.** Habit: Tree. Habitat: Forest, riverine thicket, coral rag thicket, and *Acacia*-*Commiphora* bushland, 0–500 m. Vouchers: Ngumbau V & Mwadime N V0192 (EA, HIB), Robertson 3543, Goyder et al. 4045, Robertson 4043, Kuchar P 13657 (EA).

**Grewia praecox subsp. latiovata C. Whitehouse** Habit: Shrub. Habitat: Coastal bush forest, thorny bushland, and scattered tree grassland, 0–550 m. Vouchers: RB & AJ Faden 72/89, Luke et al. in TPR 264, Luke et al. in TPR 422 (EA).

***Grewia stuhlmannii* K. Schum.** Habit: Shrub. Habitat: Forest, forest edges, and thicket, 0–500 m. Vouchers: Hooper & Townsend 1099, Drummond & Hemsley 3851, Medley 257, Greenway PJ in EAH 12202, Rawlins SP 352 (EA).

***Grewia triflora* (Bojer) Walp.** Habit: Shrub. Habitat: Coastal forest, thicket, often on coral rag, 0–30 (–100) m. Vouchers: SAJIT–006118 (EA, HIB), Drummond & Hemsley 3984, RB & AJ Faden 74/1256, Gillespie 71, Mrs Robertson SA 7756 (EA).

***Grewia tristis* K. Schum.** Habit: Shrub. Habitat: Dry *Acacia*-*Commiphora* bushland, 0–650 m. Vouchers: RB & AJ Faden 74/1282, Drummond & Hemsley 4057 (EA).

***Grewia truncata* Mast.** Habit: Shrub. Habitat: Thicket, bushland, and along river courses, ca. 0–186 m. Vouchers: Ngumbau V & Mwadime N V0356 (EA, HIB), Verdcourt 1872, Gillespie 88 (EA).

***Grewia villosa* Willd** Habit: Shrub. Habitat: Woodland and bushland, ca. 0–59 m. Vouchers: Ngumbau V & Mwadime N V0438 (EA, HIB), Gillespie 216 (EA).

***Heritiera littoralis* Aiton** Habit: Tree. Habitat: Mangrove swamps and forest on coral rag, 0–5 m. Vouchers: Abdulla 1139, MacNaughton 9246, Faden & Faden 77/646 (EA).

***Hermannia boranensis* K. Schum.** Habit: Herb. Habitat: *Acacia*-*Commiphora* bushland, 90–650 m. Voucher: Mungai 202 (EA).

***Hermannia exappendiculata* (Mast.) K. Schum.** Habit: Herb. Habitat: *Acacia*-*Commiphora* bushland, roadsides, old cultivation, coastal forest, and grassland, ca. 5 m. Vouchers: Gardner 1425, Gillett JB 203 (EA).

***Hermannia fischeri* K. Schum.** Habit: Herb. Habitat: Bushland, 300–550 m. Vouchers: Bally 16659, Drummond & Hemsley 4098, Tweedie 2570, Parker I GM/523/H (EA).

***Hermannia glanduligera* K. Schum. ex Schinz** Habit: Herb. Habitat: Road edges, old cultivation, and edges of seasonally wet areas, ca. 30 m. Voucher: Greenway PJ & Kanuri 12905 (EA).

***Hermannia kirkii* Mast.** Habit: Herb. Habitat: Forest, ca. 225–552 m. Voucher: Gillett JB 21097 (EA).

***Hermannia pseudofischeri* Cheek** Habit: Herb. Habitat: Dry bushland, ca. 150 m. Vouchers: Njoroge Thairu 98, Linder 3658 (EA).

***Hermannia uhligii* Engl.** Habit: Herb. Habitat: *Acacia*-*Commiphora* bushland or grassland, ca. 30 m. Voucher: Luke et al. in TPR 218 (EA).

***Hibiscus articulatus* Hochst. ex A. Rich.** Habit: Herb. Habitat: Woodland, grassland, and along roadsides, ca. 152 m. Voucher: Magogo FC & Glover PE 986 (EA).

***Hibiscus calyphyllus* Cav.** Habit: Shrub. Habitat: Thickets, roadsides in forests, riverine forests, and forest edges, ca. 30 m. Voucher: Festo L, Luke Q & P 2688 (EA).

***Hibiscus cannabinus* L.** Habit: Herb. Habitat: Grassland. Voucher: Luke WRQ & Robertson SA sr.

***Hibiscus ceratophorus* Thulin** Habit: Herb. Habitat: Bushland, 0–200 m. Vouchers: Mwadime N & Luke WRQ 2591 & 571 (EA).

***Hibiscus faulknerae* Vollesen** Habit: Shrub. Habitat: Bushland, 0–300 m. Vouchers: Ngumbau V & Mwadime N V0235 (EA, HIB), Drummond & Hemsley 3855, Magogo & Glover 797, Hooper & Townsend 1209, Festo L, Luke Q & P 2543 (EA).

***Hibiscus greenwayi* Baker f.** Habit: Shrub. Habitat: *Acacia*-*Commiphora* bushland, ca. 0–200 m. Vouchers: Polhill & Paulo 835, Verdcourt 3904 (EA).

***Hibiscus hildebrandtii* Sprague & Hutch.** Habit: Herb. Habitat: Grassland and bushland, 0–500 m. Vouchers: Drummond & Hemsley 4244, Adamson 52, Kabuye et al. in TPR 442, Festo L, Luke Q & P 2707, Graham RM 611 (EA).

***Hibiscus holstii* Mwachala** Habit: Liana. Habitat: Bushland and forest on limestone, 70–500 m. Vouchers: Ngumbau V & Mwadime N V027 (EA, HIB), Luke & Robertson 1666, 1858 & 2266 (EA): Vulnerable.

***Hibiscus micranthus* L. f.** Habit: Herb. Habitat: Grassland and bushland, ca. 92 m. Vouchers: SAJIT–006136 (EA, HIB), Greenway PJ 10849 (EA).

***Hibiscus panduriformis* Burm. f.** Habit: Herb. Habitat: Seasonally flooded areas in grassland and woodland, ca. 5 m. Vouchers: SAJIT–006102 (EA, HIB), Hooper & Townsend 1235 (EA).

***Hibiscus physaloides* Guill. & Perr.** Habit: Herb. Habitat: Woodland and abandoned cultivations ca. 227 m. Vouchers: Ngumbau V & Mwadime N V0271 (EA, HIB), Magogo & Estes 1202, Napier 6354, Luke WRQ et al. 4730 (EA).

***Hibiscus rhabdotospermus* Garcke** Habit: Herb. Habitat: Sandy or stony ground and among rocks on inselbergs, 300–700 m. Vouchers: van Someren 887, Mungai & Rucina 490/84 (EA).

***Hibiscus schizopetalus* (Mast.) Hook. f.** Habit: Shrub. Habitat: Coastal bushland and forest, 0–200 m. Vouchers: Ngumbau V & Mwadime N V0384 (EA, HIB), Jex-Blake s.n., Drummond & Hemsley 4256, Robertson & Luke 5811, Festo L, Luke Q & P 2661, Luke WRQ & PA 4578 (EA).

***Hibiscus sidiformis* Baill.** Habit: Herb. Habitat: Rocky areas in dry woodland, ca. 350 m. Voucher: Gilbert & Thulin 1738 (EA).

***Hibiscus surattensis* L.** Habit: Herb. Habitat: Coastal habitats such as sand dunes, ca. 15 m. Voucher: Luke Q 5666 (EA).

**Hibiscus sp. aff. lunariifolius Willd.** Habit: Herb. Habitat: Forest, ca. 0–10 m. Vouchers: SA Robertson 4247, Mwadime & Chesire 302 (EA).

***Hibiscus tiliaceus* L.** Habit: Shrub. Habitat: Sandy seashores and mangrove swamps, 0–20 m. Vouchers: Graham 738, Jeffery 142, Abdulla 2197 (EA).

***Hibiscus vitifolius* L.** Habit: Herb. Habitat: *Acacia* bushland, grassland, woodland, and forest edges, ca. 620 m. Vouchers: Drummond & Hemsley 3989, Festo L, Luke Q & P 2522 (EA).

***Leptonychia usambarensis* K. Schum.** Habit: Tree. Habitat: Moist forest, 200–350 m. Vouchers: Luke & Robertson 2721, Robertson SA & Luke WRQ 4605 (EA).

***Melhania annua* Thulin** Habit: Herb. Habitat: Sand dunes and eroded sand dunes, 0–50 m. Vouchers: Faden & Faden 74/1107, Gillepie 75 (EA).

***Melhania denhardtii* K. Schum**. Habit: Herb. Habitat: Open *Acacia*-*Commiphora* bushland, 5–250 m. Vouchers: Donald 2499, Graham 1641, Luke & Robertson 2524 (EA).

***Melhania ovata* (Cav.) Spreng.** Habit: Herb. Habitat: Bushland, ca. 30 m. Voucher: Reitsma J 293 (EA).

***Melhania parviflora* Chiov.** Habit: Herb. Habitat: *Acacia*-*Commiphora* woodland and coastal bushland, 0–850 m. Vouchers: Rawlins 800, Verdcourt 3950 (EA).

***Melhania rotundata* Hochst. ex Mast.** Habit: Herb. Habitat: Open to dense *Acacia*-*Commiphora* woodland or bushland, ca. 100 m. Vouchers: Gillet 16437, Luke WRQ & Robertson SA 2524 (EA).

***Melhania velutina* Forssk.** Habit: Herb. Habitat: Wooded grassland, bushland, and riverine forest, 0–700 m. Vouchers: Polhill & Paulo 874, Rawlins SP 82 (EA).

***Melochia corchorifolia* L.** Habit: Herb. Habitat: Roadsides, and grassland, 0–227 m. Vouchers: Ngumbau V & Mwadime N V0273 (EA, HIB), Jeffery K518, Drummond & Hemsley 3928, Luke et al. TPR 522, Kirika P, Mbale M & Mbatha M 772, Magogo FC & Glover PE 160 (EA).

***Melochia melissifolia* Benth.** Habit: Herb. Habitat: Swamps and swamp edges or less frequently sandy soils, ca. 30 m. Vouchers: SAJIT–005994 & 006199 (EA, HIB), Robertson & Luke 6345, Graham 1900, Luke & Luke 5995 (EA).

***Nesogordonia holtzii* (Engl.) Capuron ex L. C. Barnett & Dorr** Habit: Tree. Habitat: Evergreen coastal forest, 1–500 m. Vouchers: Dale 1127, Luke & Robertson 2627, Barnett, Dorr & Check 518, Robertson SA & Luke WRQ 5526 (EA).

***Pavonia arenaria var. microphylla* (Ulbr.) Verdc.** Habit: Herb. Habitat: Woodland, bushland, flood plains, and rocky slopes with dense grass cover, ca. 0–150 m. Voucher: Kabuye et al. TPR 219 (EA).

***Pavonia blepharicarpa* N.A. Brummitt & Vollesen** Habit: Herb. Habitat: Wooded grassland, 100–550 m. Voucher: Robertson 1815 (EA).

***Pavonia elegans* Garcke** Habit: Herb. Habitat: Mixed dry evergreen bushland, ca. 90 m. Vouchers: Verdcourt 3903, JB Gillett 16420 (EA).

***Pavonia ellenbeckii* Gürke** Habit: Herb. Habitat: Wooded grassland, ca. 100 m. Voucher: Drummond & Hemsley 3761 (EA).

***Pavonia leptocalyx* (Sond.) Ulbr.** Habit: Herb. Habitat: Marshy grassland, mixed coastal bushland, and thicket, 5–300 m. Vouchers: SAJIT–005980, Ngumbau V & Mwadime N V0181 (EA, HIB), Robertson & Luke 5995, RM Graham 805 in FD 2353, Greenway 9261, Robertson SA & Luke WRQ 5186 (EA).

***Pavonia sennii* Chiov.** Habit: Herb. Habitat: Woodland, 35–120 m. Voucher: Festo L, Luke Q & P 2597 (EA).

***Roifia dictyocarpa* (Webb) Verdc.** Habit: Herb. Habitat: Bushland with grassland and floodplain bushland, 200–750 m. Voucher: Gillett & Gachathi 20543 (EA).

***Senra incana* Cav.** Habit: Herb. Habitat: Bushland, scattered tree grassland, 35–120 m. Vouchers: Thairu 92, Kabuye et al. TPR 744 (EA).

***Sida acuta* Burm. f.** Habit: Herb. Habitat: Grassland, bushland, and forest edges, 0–150 m. Vouchers: Drummond & Hemsley 4237, Magogo FC & Glover PE 660 (EA).

***Sida chrysantha* Ulbr.** Habit: Herb. Habitat: Coastal grassland, wooded grassland, and bushland, 0–100 m. Vouchers: Rawlins 767, Drummond & Hemsley 4240 (EA).

***Sida cordifolia* L.** Habit: Herb. Habitat: Very mixed bushland and thicket, evergreen forest, and cultivated area, 0–450 m. Vouchers: Drummond & Hemsley 4236 & 4240, MacNaughton 93, Rawlins 175 & 767 (EA).

***Sida ovata* Forssk.** Habit: Herb. Habitat: Dry areas ca. 5–15 m. Vouchers: Luke WRQ et al. 5706, Robertson SA 6848 (EA).

**Sida rhombifolia var. serratifolia (R. Wilczek & Steyaert) Verdc.** Habit: Herb. Habitat: Forest margin, and scrub, 0–200 m. Voucher: Robertson SA 3439 (EA).

***Sida spinosa* L.** Habit: Herb. Habitat: Wastelands, ca. 10 m. Voucher: Festo L & Luke Q 2448 (EA).

***Sida tanaensis* Vollesen** Habit: Herb. Habitat: Open bushland and scattered tree grassland, 30–250 m. Voucher: Kabuye et al. TPR 346 (EA).

***Sterculia appendiculata* K. Schum** Habit: Tree. Habitat: Coastal and lowland riverine, 5–750 m. Vouchers: Ngumbau V & Mwadime N V0476 (EA, HIB), Gillet & Kibuwa 19948, Bally 2036, Dale 3772, Birch WH s.n., Mulwa PCW 60 (EA).

***Sterculia foetida* L.** Habit: Tree. Habitat: Lowland dry woodlands, 0–100 m. Voucher: Robertson SA 3662 (EA): Cultivated.

***Sterculia rhynchocarpa* K. Schum** Habit: Tree. Habitat: Woodland and bushland, ca. 10–70 m. Vouchers: Ngumbau V & Mwadime N V0446 (EA, HIB), Gathii S 142, Wamukoya K7–x62a (EA).

***Sterculia schliebenii* Mildbr.** Habit: Tree. Habitat: Coastal evergreen forest, 20–400 m. Vouchers: Luke 896, Faden, Evans & Mahasi 70/826, Luke & Robertson 1739, Luke WRQ & Robertson SA 534, Hawthorne W 276 (EA).

***Thespesia danis* Oliv.** Habit: Shrub. Habitat: Riverine grassland, coastal bushland, and thicket, 0–450 m. Vouchers: SAJIT–006449, Ngumbau V & Mwadime N V0129 (EA, HIB), Verdcourt 3217, Tweedie 1007, Magogo FC & Glover PE 700 (EA).

***Thespesia populnea* (L.) Corrêa** Habit: Shrub. Habitat: Mangrove swamps, 0–6 m. Vouchers: SAJIT–006249 (EA, HIB), Thomas s.n., Greenway & Rawlins 9484 (EA).

**Triumfetta flavescens Hochst. ex A. Rich. var. flavescens Corrêa** Habit: Shrub. Habitat: Open bushland, 225–552 m. Vouchers: P Kuchar, F Msafiri & R Seet 5840, J Mutanga & J Muasya 261, JB Gillett 21114 (EA).

***Triumfetta heterocarpa* Sprague & Hutch.** Habit: Herb. Habitat: *Acacia*-*Commiphora* bushland on sandy, silt or laterite soils, ca. 30 m. Voucher: GC van Someren 880 (EA).

***Triumfetta rhomboidea* Jacq.** Habit: Herb. Habitat: Grassland, ca. 95 m. Vouchers: SAJIT–006141 (EA, HIB), Magogo FC & Glover PE 455 (EA).

***Triumfetta tomentosa* Bojer** Habit: Herb. Habitat: Forests, swamp margins, and bushland, ca. 60 m. Vouchers: Magogo FC & Estes R 1191, Nyange M 510 (EA).

***Urena procumbens* L.** Habit: Herb. Habitat: Roadsides and waste places, 0–200 m. Voucher: SAJIT–006196 (EA, HIB).

**Urena lobata var. sinuata (L.) Borss. Waalk.** Habit: Herb. Habitat: Damp grassland and areas of cultivations, 0–15 m. Voucher: Luke 2915 (EA).

***Waltheria indica* L.** Habit: Herb. Habitat: Dry grassland, cultivated, and disturbed places, 0–300 m. Vouchers: SAJIT–006128 (EA, HIB), Katz SS KNMUS/H197/75, Magogo FC & Glover PE 31 (EA).

**F108. Melastomataceae**

7 Genera, 12 Species

***Antherotoma debilis* (Sond.) Jacques-Fél.** Habit: Herb. Habitat: Valley grassland, ca. 10 m. Voucher: RM Graham LL 731 in FD 2183 (EA).

***Antherotoma senegambiensis* (Guill. & Perr.) Jacques-Fél.** Habit: Herb. Habitat: Along river banks and forest margins. Voucher: Reitsma J 239 (EA).

***Clidemia hirta* (L.) D. Don** Habit: Shrub. Habitat: Moist or wet mixed forest and thickets, ca. 290 m. Vouchers: SAJIT–005504 (EA, HIB), Luke WRQ & Robertson SA 2712 (EA).

***Heterotis rotundifolia* (Sm.) Jacq.-Fél.** Habit: Herb. Habitat: Margins of rain forest, riverine forest, flood plains, and valley grassland, ca. 0–426 m. Vouchers: Ngumbau V & Mwadime N V083 & 0264 (EA, HIB), RM Graham in FD 2189, Bally 8898, Purse-glove 1727, Rawlins SP 342, Magogo FC & Glover PE 112 (EA).

***Memecylon fragrans* A. Fern. & R. Fern.** Habit: Shrub. Habitat: Coastal dry evergreen forest, 60–100 m. Vouchers: SAJIT–005976 & 006161, Ngumbau V & Mwadime N V0239 (EA, HIB), Drummond & Hemsley 3859, Verdcourt 1180, Perdue & Kibuwa 10010, Jeffery GW 616 (EA).

***Memecylon verruculosum* Brenan** Habit: Shrub. Habitat: Riverine forest and lowland evergreen forest, 300–750 m. Vouchers: Luke WRQ 2939, Gardner in FD 1447, Drummond & Hemsley 3971, Magogo & Glover 237 (EA): Vulnerable.

***Tristemma mauritianum* J. F. Gmel.** Habit: Shrub. Habitat: Marshy clearings in rainforest and swampy riverine forest, ca. 290 m. Vouchers: Glover & Etes 1153, Luke WRQ & Robertson SA 2715 (EA).

***Warneckea amaniensis* Gilg** Habit: Shrub. Habitat: Lowland rainforest and riverine forest, 40–600 m. Vouchers: SAJIT–006068 (EA, HIB), Gardner in FD 1409, Drummond & Hemsley 3931, Moomaw 1276, Brenan JPM & JH GilletJB 14593, Stone RD & Luke WRQ 2415 (EA).

***Warneckea hedbergiorum* Borhidi** Habit: Shrub. Habitat: Moist forest, ca. 250 m. Vouchers: Ngumbau V & Mwadime N V0238 (EA, HIB), Stone RD & Luke WRQ 2416 (EA).

***Warneckea mouririifolia* (Brenan) Borhidi** Habit: Shrub. Habitat: Lowland dry evergreen forest and rainforest, 45–100 m. Vouchers: SAJIT–005924 (EA, HIB), Drummond & Hemsley 3857, RM Graham in FD 1527, Polhill & Paulo 852, Luke Q 1501, Luke WRQ & Robertson SA 2805, Festo L, Luke PA & WRQ 2723 (EA): Vulnerable.

***Warneckea melindensis* (A. Fern. & R. Fern.) R. D. Stone & Q. Luke** Habit: Shrub. Habitat: Coastal forest and thicket, 10–100 m. Vouchers: Dale IR 3835, Luke WRQ 4574A, Graham 1951, Jeffery 570, Faden 71/751 (EA).

**Warneckea sansibarica (Taub.) Jacq.-Fél. subsp. sansibarica** Habit: Shrub. Habitat: Lowland dry evergreen forest and deciduous woodland, 10–100 m. Vouchers: Donald 1 in FD 2327, Trump 1361, Gisau in EAH 10985, Robertson SA & Abio, LG 6815, Luke WRQ & Robertson SA 2783, Gillett JB 20425, Robertson SA & Luke WRQ 5479 (EA).

**Warneckea sansibarica subsp. buchananii (Gilg) Borhidi** Habit: Tree. Habitat: Forest, ca. 30 m. Vouchers: Mohamed Abdullah 3351, Perdue RE & Kibuwa SP 10019 (EA).

**F109. Meliaceae**

9 Genera, 16 Species

***Azadirachta indica* A. Juss.** Habit: Tree. Habitat: Dry Habitat, ca. 0–20 m. Voucher: Hume L 10 (EA): Cultivated.

***Ekebergia capensis* Sparrm.** Habit: Tree. Habitat: Variable habitats, ca. 10 m. Voucher: Luke Q 1514 (EA).

***Lovoa swynnertonii* Baker f.** Habit: Tree. Habitat: Lowland and mid altitude rainforest, ca. 180 m. Vouchers: Verdcourt 3936a, White 11334, Luke WRQ & Robertson SA 2731 (EA): Near Threatened.

***Melia azedarach* L.** Habit: Tree. Habitat: Secondary grassland, thicket, and waste ground, ca. 100 m. Voucher: Perdue & Kibuwa 10118 (EA): Cultivated.

***Pseudobersama mossambicensis* (Sim) Verdc.** Habit: Tree. Habitat: Understorey and edges of moist lowland forest, 60–300 m. Vouchers: SAJIT–005519, 005553 & 005972, Ngumbau V & Mwadime N V085 (EA, HIB), White 11319, Greenway 9814, Trump 141, Robertson SA 4529 & 3460 (EA): Near Threatened.

***Toona ciliata* M. Roem.** Habit: Tree. Habitat: Thickets, forests, ca. 400 m. Voucher: Lennox K 10566 (EA): Cultivated.

***Trichilia emetica* Vahl** Habit: Tree. Habitat: Coastal forest, drier types of riparian forest, and wooded grassland, ca. 10–407 m. Vouchers: SAJIT–006042 (EA, HIB), White 11399, Sangai GW 971, Robertson SA 4238A (EA).

***Turraea floribunda* Hochst.** Habit: Shrub. Habitat: Evergreen forest and riverine forest, ca. 100–237 m. Vouchers: SAJIT–005509 & 005583, Ngumbau V & Mwadime N V0169 (EA, HIB), Brenan et al. 14622, Battiscombe 111, Robertson SA & Luke WRQ 5493, Luke WRQ & Robertson SA 2772 (EA).

***Turraea holstii* Gürke** Habit: Tree. Habitat: Moist to dry forest, ca. 99 m. Voucher: Kimeu JM 645 (EA).

**Turraea mombassana Hiern ex C. DC. subsp. mombassana** Habit: Shrub. Habitat: Evergreen forest, coastal scrub, and secondary forest, ca. 0–400 m. Vouchers: SAJIT–005925 & 005548, Ngumbau V & Mwadime N V0188 (EA, HIB), Drummond & Hemsley 1108, RM Graham 1947, Rawlins 230, Luke WRQ & Robertson SA 502, Rawlins SP 11261 (EA).

***Turraea nilotica* Kotschy & Peyr.** Habit: Tree. Habitat: Woodland, bushland, and wooded grassland, ca. 100 m. Vouchers: Luke Q 5641, Magogo FC & Glover PE 96, Greenway & Rawlins 8905 (EA).

***Turraea parvifolia* Deflers** Habit: Shrub. Habitat: *Acacia*-*Commiphora* bushland and banks of seasonal watercourses, ca. 260 m. Voucher: Hemming 83/42 (EA).

***Turraea robusta* Gürke** Habit: Tree. Habitat: Thickets and *Brachystagia* woodland, ca. 30 m. Voucher: Jeffery GW 446 (EA).

***Turraea wakefieldii* Oliv.** Habit: Tree. Habitat: Coastal forest and secondary forest, near sea level. Vouchers: Ngumbau V & Mwadime N V0201 (EA, HIB), Drummond & Hemsley 4187, Luke Q 1402, Robertson SA 5069 (EA).

***Xylocarpus granatum* J. Koenig** Habit: Tree. Habitat: In tidal mud of mangrove swamps. Vouchers: RM Graham in FD 2236, Sangai 849, Greenway & Rawlins 8869 (EA).

***Xylocarpus moluccensis* (Lam.) M. Roem.** Habit: Tree. Habitat: In coastal scrub, above high-water mark. Vouchers: Kibuwa 1203, RB & AJ Faden 77/553, Rawlins 144, Kuchar P 13584 (EA).

**F110. Menispermaceae**

9 Genera, 11 Species

***Albertisia undulata* (Hiern) Forman** Habit: Liana. Habitat: Dry thicket, 50–450 m. Vouchers: Luke WRQ & Robertson SA 2744 & 1745, NMK Mrima/Dzombo Exp. 218 & 311 (EA).

**Anisocycla blepharosepala subsp. tanzaniensis Vollesen** Habit: Liana. Habitat: Bushland, 1–150 m. Vouchers: Robertson SA 5485, Festo L, Luke Q & P 2660 (EA).

***Chasmanthera dependens* Hochst.** Habit: Liana. Habitat: Rainforest, regrowth, and fringing forest, ca. 600 m. Voucher: Festo L & Luke Q 2559 (EA).

**Cissampelos nigrescens Diels var. nigrescens** Habit: Climber. Habitat: Moist forest, ca. 15 m. Vouchers: SAJIT–006218 (EA, HIB), Luke WRQ 3125 (EA): Vulnerable.

***Cissampelos pareira* L.** Habit: Climber. Habitat: Forest, deciduous woodland, and secondary vegetation, ca. 15 m. Vouchers: SAJIT–006142, Ngumbau V & Mwadime N V0263 (EA, HIB), Luke Q 1452, Robertson SA & Luke WRQ 6035, Jeffery K360 (EA).

***Dioscoreophyllum volkensii* Engl.** Habit: Climber. Habitat: Lowland rainforest, 0–15 m. Vouchers: SAJIT–005538 (EA, HIB), Faden RB 69/489, Drummond & Hemsley 4037 (EA).

***Jateorhiza palmata* (Lam.) Miers** Habit: Climber. Habitat: Lowland rainforest and riverine forest, 0–231 m. Vouchers: Ngumbau V & Mwadime N V0331 (EA, HIB), Kirika P, Mbale M & Mbatha M 770, Faden RB 70/430, Graham 1797 (EA).

***Tiliacora funifera* (Miers) Oliv.** Habit: Liana. Habitat: Riverine, evergreen forest, and moist areas in woodland, ca. 20 m. Vouchers: Mwadime N 17, Robertson SA & Luke WRQ 5169 (EA).

***Tinospora caffra* (Miers) Troupin** Habit: Climber. Habitat: Lowland, upland rainforest, deciduous bushland, and often near rock outcrops, ca. 350 m. Vouchers: Robertson SA & Luke WRQ 5978, Drummond & Hemsley 4155 (EA).

***Tinospora oblongifolia* (Engl.) Troupin** Habit: Liana. Habitat: Lowland rainforest and coastal evergreen bushland, 0–304 m. Vouchers: Ngumbau V & Mwadime N V0523 (EA, HIB), Drummond & Hemsley 3905 & 3244, van Someren Sh 115, Robertson SA & Luke WRQ 1410, (EA).

***Triclisia sacleuxii* (Pierre) Diels** Habit: Liana. Habitat: Evergreen forest, 50–500 m. Vouchers: Ngumbau V & Mwadime N V0474 (EA, HIB), Mahasi AS EA 14893, Mwadime N 51 (EA).

**F111. Menyanthaceae**

1 Genus, 1 Species

***Nymphoides forbesiana* (Griseb.) Kuntze** Habit: Herb. Habitat: Shallow water at the edge of lakes, pools, and rivers, ca. 0–30 m. Vouchers: Gillett 18725, Sacleux 2306, Whyte 40 (EA).

**F112. Metteniusaceae**

2 Genera, 2 Species

***Apodytes dimidiata* E. Mey. ex Arn.** Habit: Tree. Habitat: Widespread from coastal evergreen bushland to upland rainforest, ca. 0–200 m. Vouchers: SAJIT–006145 (EA, HIB), Luke Q 1427, Robertson SA 4218, Polhill & Paulo 820 (EA).

***Rhaphiostylis beninensis* (Hook. f. ex Planch.) Planch. ex Benth.** Habit: Liana. Habitat: Lowland rainforests. Voucher: Luke WRQ 8327B (EA).

**F113. Molluginaceae**

2 Genera, 4 Species

***Glinus oppositifolius* (L.) Aug. DC.** Habit: Herb. Habitat: Dry regions, seasonally swampy, and dry river beds, ca. 40 m. Vouchers: SAJIT–006212 (EA, HIB), Luke Q 1425, Rawlins SP 289, Jeffery K466 (EA).

***Glinus lotoides* L.** Habit: Herb. Habitat: Woodlands, mixed savannah woodland, grassland, riverine forest, and river banks ca. 15 m. Voucher: Reitsma J 413 (EA).

***Glinus setiflorus* Forssk.** Habit: Herb. Habitat: Dry river bed, bushland, and woodland, ca. 90 m. Vouchers: Greenway 9236, JB Gillett 16414 (EA).

***Hypertelis umbellata* (Forsk.) Thulin** Habit: Herb. Habitat: Roadsides, cultivated areas. Vouchers: Townsend CC & Hooper SS 1224, Jeffery K607 (EA).

**F114. Montiniaceae**

1 Genus, 1 Species

**Grevea eggelingii var. keniensis (Verdc.) Verdc.** Habit: Shrub. Habitat: Evergreen forest, near sea-level. Vouchers: SAJIT–006089 & 005444 (EA, HIB), Napier 3348 (EA): Endangered, Endemic.

**F115. Moraceae**

8 Genera, 34 Species

**Antiaris toxicaria var. subsp. welwitschii (Engl.) C.C. Berg usambarensis (Engl.) C. C. Berg** Habit: Tree. Habitat: Rainforest, evergreen forests, and riverine, ca. 300 m. Voucher: Verdcourt 3937 (EA).

***Dorstenia alta* Engl.** Habit: Shrub. Habitat: Undershrub in evergreen forest and near streams, up to 800 m. Vouchers: Ngumbau V & Mwadime N V0400 (EA, HIB), Luke WRQ & Robertson SA 2695, Drummond & Hemsley 1199, Faden et al. 70/944, B Adams 103 (EA).

***Dorstenia barnimiana* Schweinf.** Habit: Herb. Habitat: Wooded and open grassland, ca. 300 m. Voucher: Luke WRQ & Robertson SA 2560 (EA).

**Dorstenia cuspidata var. brinkmaniana Hijman** Habit: Herb. Habitat: Deciduous, coastal bushland, thickets, wooded grassland, and along rivers, ca. 250 m. Vouchers: Mason in EAH 15404, Luke 1577 (EA).

***Dorstenia foetida* (Forssk.) Schweinf.** Habit: Herb. Habitat: Rock outcrops and open places in deciduous, and succulent bushland, ca. 100 m. Voucher: Faden 74/1017 (EA).

***Dorstenia goetzei* Engl.** Habit: Herb. Habitat: Forest in wet shaded places and often among rocks, ca. 200 m. Vouchers: Luke WRQ & Robertson SA 1893, Faden 71/790, B Adams 82 (EA): Near Threatened.

***Dorstenia hildebrandtii* Engl.** Habit: Herb. Habitat: Open forest to woodland, bushland, and succulent thickets, ca. 0–330 m. Vouchers: Drummond & Hemsley 4254, Luke WRQ & Robertson SA 1464 (EA).

***Dorstenia kameruniana* Engl.** Habit: Shrub. Habitat: Undergrowth of evergreen forests, often near streams, ca. 231 m. Vouchers: SAJIT–005528, Ngumbau V & Mwadime N V112 (EA, HIB), Dale in FD 3545, Drummond & Hemsley 1154, Faden et al. 70/947, Robertson SA 5083 (EA).

**Dorstenia tayloriana Rendle var. tayloriana** Habit: Herb. Habitat: Lowland evergreen forest, up to 600 m. Vouchers: Ngumbau V & Mwadime N V0312 & V0475 (EA, HIB), WE Taylori, Dale IR 3827 (EA): Vulnerable.

**Dorstenia tayloriana var. laikipiensis (Rendle) Hijman** Habit: Herb. Habitat: Evergreen forest and coastal forests, ca. 240 m. Vouchers: SAJIT–005530, Ngumbau V & Mwadime N V0108 (EA, HIB), Gardner in FD 1440, Drummond & Hemsley 3959, Faden 74/1271, Faden RB 70/427, Malombe I, Mwandime N & Saidi 1663, Luke PA & WRQ 5980 (EA).

***Dorstenia warneckei* Engl.** Habit: Herb. Habitat: Forest in shady and sometimes wet places, 200–750 m. Vouchers: Glover et al. 1155, Luke WRQ & Robertson SA 471, Luke WRQ 1644 (EA): Near Threatened.

***Ficus bubu* Warb.** Habit: Tree. Habitat: Forest, riverine, lakesides, and other ground water forest, ca. 100 m. Vouchers: Dale 2014, Luke WRQ & Robertson SA 2788 (EA).

***Ficus craterostoma* Warb. ex Mildbr. & Burret** Habit: Tree. Habitat: Along the margins of evergreen forest, ca. 36 m. Voucher: SAJIT–006190 (EA, HIB).

***Ficus exasperata* Vahl** Habit: Tree. Habitat: Forest, often at edges, in rocky places, and along rivers, ca. 200 m. Vouchers: Ngumbau V & Mwadime N V0403 (EA, HIB), Gillett 18636, BR Adams 102, Robertson SA 4239 (EA).

***Ficus faulkneriana* C.C. Berg** Habit: Tree. Habitat: Coastal bushland and wooded grassland, 0–450 m. Voucher: Magogo & Glover 51 (EA): Vulnerable.

***Ficus ingens* (Miq.) Miq.** Habit: Tree. Habitat: Wooded savannah, sometimes in rocky places. Voucher: Robertson SA 5216 (EA).

**Ficus lingua subsp. depauperata (Sim) C.C. Berg** Habit: Tree. Habitat: Forest, coastal bushland, and coral outcrops, 0–50 m. Vouchers: Gillet & Kibuwa 19904, Perdue & Kibuwa 10103, Greenway & Rawlins 9346 (EA).

***Ficus lutea* Vahl** Habit: Tree. Habitat: Deciduous woodland, wooded grassland, and forest. Voucher: Robertson SA & Luke WRQ 5145 (EA).

***Ficus natalensis* Hochst.** Habit: Tree. Habitat: Riverine and coastal forests or swamp forest, ca. 200 m. Vouchers: Luke WRQ & Robertson SA 190, Robertson SA 5068 (EA).

**Ficus ottoniifolia subsp. ulugurensis (Mildbr. & Burret) C.C. Berg** Habit: Tree. Habitat: Rainforest, riverine, and coastal bushland, ca. 100 m. Vouchers: Verdcourt 3949, BR Adams 116, Luke WRQ 880 (EA).

***Ficus polita* Vahl** Habit: Tree. Habitat: Evergreen forests, bushland, semi-deciduous forest, and coastal rainforest. Voucher: Luke WRQ & PA sr.

***Ficus recurvata* De Wild.** Habit: Tree. Habitat: Lowland forest, riverine, swampy forest, and flood plains, 0–550 m. Vouchers: Jeffery 497, Magogo FC & Glover PE 69 (EA).

**Ficus sansibarica Warb. subsp. sansibarica** Habit: Tree. Habitat: Evergreen forest, including drier types, and coastal bushland, ca. 0–250 m. Vouchers: Bally & Smith 14397, Dale 2008, Verdcourt 2409, Luke Q 1428, Gray M & Luke WRQ 290, Mulwa PCW 57 (EA).

**Ficus scassellatii Pamp. subsp. scassellatii** Habit: Tree. Habitat: Rainforest, lakesides, riverine, and ground water forest, ca. 300 m. Vouchers: Polhill & Paulo 807, Saufferer S 896 (EA).

***Ficus stuhlmannii* Warb.** Habit: Tree. Habitat: Wooded grassland, bushland, and often along lakesides, ca. 0–400 m. Vouchers: Ngumbau V & Mwadime N V0539 (EA, HIB), Robertson SA & Luke WRQ 5863, Pakia M 979, Dale in FD 3634 (EA).

***Ficus sur* Forssk.** Habit: Tree. Habitat: Woodland, riverine, and semi-deciduous forest, ca. 150 m. Vouchers: Brenan JPM, Gillett JB 14573, Luke WRQ & Robertson SA 249 (EA).

***Ficus sycomorus* L.** Habit: Tree. Habitat: Forest edges, lakesides, riverine, and rock outcrops, ca. 0–250 m. Vouchers: Greenway & Rawlins 9362, Mulwa PCW 13 (EA).

**Ficus tremula Warb. subsp. tremula** Habit: Tree. Habitat: Lowland dry evergreen forest, woodland, and coastal bushland, 0–600 m. Vouchers: Greenway 9800, Verdcourt 3288, Gillett & Kibuwa 19893 (EA).

***Ficus usambarensis* Warb.** Habit: Tree. Habitat: Woodland, ca. 500 m. Vouchers: Ngumbau V & Mwadime N V0240 (EA, HIB), Mulwa PCW 57 (EA).

***Ficus wakefieldii* Hutch.** Habit: Tree. Habitat: Riverine, lakesides, and wooded grassland, ca. 230 m. Vouchers: Jembe SM & Morimoto Y 47, Robertson SA & Luke WRQ 4469 (EA).

***Maclura africana* (Bureau) Corner** Habit: Tree. Habitat: Bushland and dry evergreen forest, 0–720 m. Vouchers: Bally 8908, Drummond & Hemsley 3922, RM Graham in FD 1953, Luke Q 1444, Wamukoya K7x57a (EA).

***Milicia excelsa* (Welw.) C.C. Berg** Habit: Tree. Habitat: Rainforest, lowland evergreen forest, riverine, and ground water forest, ca. 0–300 m. Vouchers: Dale in FD 1072, CW Elliot 212, Robertson SA 4221 (EA).

***Morus mesozygia* Stapf** Habit: Tree. Habitat: Semi deciduous forests, ca. 20 m. Voucher: Thomas Mwadime in Mrs Robertson SA 7785 (EA).

***Streblus usambarensis* (Engl.) C.C.Berg** Habit: Shrub. Habitat: Forest and often riverine, 30–700 m. Vouchers: SAJIT–006187 & 005534 (EA, HIB), Brenan, Gillett et al. in EAH 14534, Faden, Evans & Msafiri 70/950, Gilbert & May 5341, Luke WRQ & Robertson SA 528 (EA).

***Trilepisium madagascariense* DC.** Habit: Tree. Habitat: Rainforest, wetter evergreen forests, riverine, and ground water forest, ca. 100 m. Voucher: RM Graham in FD 1643 (EA).

**F116. Moringaceae**

1 Genus, 1 Species

***Moringa oleifera* Lam.** Habit: Tree. Habitat: Cultivations, grassland on black clay, and bushland on coral, ca. 15 m. Voucher: Verdcourt B 1102 (EA): Naturalized.

**F117. Myristicaceae**

1 Genus, 1 Species

***Cephalosphaera usambarensis* (Warb.) Warb.** Habit: Tree. Habitat: Rainforest on steep slopes, also lowland streamside forest, ca. 200 m. Vouchers: Luke & Robertson 2704A & 2704B (EA): Vulnerable.

**F118. Myrtaceae**

2 Genera, 7 Species

**Eugenia capensis subsp. multiflora Verdc.** Habit: Shrub. Habitat: Dry evergreen forest, bushland, coastal thicket, and mangrove swamp edges, 0–350 m. Vouchers: Robertson & Luke 5165 & 5640, Sampson 30 (EA).

***Eugenia nigerina* A. Chev.** Habit: Shrub. Habitat: Thicket and river beds, 18–360 m. Vouchers: Drummond & Hemsley 4143, Robertson & Luke 6083 & 5325, Festo L & Luke WRQ 2537 (EA).

***Eugenia tanaensis* Verdc.** Habit: Shrub. Habitat: On rocks at high water mark of river, (5–) 800 m. Vouchers: Drummond & Hemsley 4260, Robertson & Luke 5813 (EA).

***Eugenia verdcourtii* Byng** Habit: Shrub. Habitat: Dry evergreen forest and bushland, coastal thicket, mangrove swamp edges, flood plains, 0–350 m. Vouchers: Ngumbau V & Mwadime N V0317 (EA, HIB), Luke Q 1439, Verdcourt 5283 (EA).

**Syzygium cordatum subsp. shimbaense Verdc.** Habit: Tree. Habitat: Forest edges, grassland, and scattered trees, (100–) 300–450 m. Vouchers: SAJIT–005557 (EA, HIB), RM Graham Q251 in FD 1775, Brenan et al. 14559, Magogo & Glover 104, Luke WRQ & PA 5735 (EA).

***Syzygium cumini* (L.) Skeels** Habit: Tree. Habitat: Coastal bushland and savannahs on coral rock, ca. 200 m. Vouchers: Perdue & Kibuwa 10115, Magogo FC & Glover PE 859 (EA): Cultivated.

***Syzygium guineense* (Willd.) DC. ?subsp. nov**, Habit: Tree. Habitat: Forest. Voucher: Luke WRQ & PA 4511 (EA).

**Syzygium guineense subsp. afromontanum F. White** Habit: Tree. Habitat: Evergreen forest, ca. 100 m. Voucher: Luke WRQ & Robertson SA 2683 (EA).

**F119. Nyctaginaceae**

2 Genera, 6 Species

***Boerhavia coccinea* Mill.** Habit: Herb. Habitat: Disturbed, rocky, and swampy ground, ca. 0–10 m. Vouchers: Tweedie 933, Frazier J 2221 (EA).

***Boerhavia diffusa* L.** Habit: Herb. Habitat: Disturbed ground in cultivation, waste places, and grassland, ca. 65 m. Vouchers: SAJIT–005592 (EA, HIB), Moomaw 1417, Polhill & Paulo 534, Bally 13268 (EA).

***Boerhavia erecta* L.** Habit: Herb. Habitat: Disturbed sandy, bushland, waste ground, cultivation, and on roadsides, ca. 213 m. Vouchers: SA Robertson 3335, Gerhardt K & Steiner M 44, Magogo FC & Glover PE 725 (EA).

***Boerhavia* sp A of FTEA** Habit: Herb. Habitat: Bushland and forest, ca. 10 m. Voucher: Luke Q 5463 (EA): Endemic.

***Pisonia aculeata* L.** Habit: Liana. Habitat: Found along coasts, hedges, rain forests, and open forests, ca. 20 m. Voucher: Mwadime N 9 (EA).

***Pisonia grandis* R. Br.** Habit: Shrub. Habitat: Coral outcrops along the coast, shoreline. Vouchers: Frazier 2251 & 2252 (EA).

**F120. Nymphaeaceae**

1 Genus, 2 Species

***Nymphaea lotus* L.** Habit: Herb. Habitat: Sheltered water, ca. 200 m. Vouchers: Verdcourt 3195, Festo L, Luke Q & P 2678 (EA).

**Nymphaea nouchali var. caerulea (Savigny) Verdc.** Habit: Herb. Habitat: Pools or lake edges, also swamps, seasonal ponds, and waterholes, ca. 0–700 m. Vouchers: Bally 8558, Gillett JB 20426 (EA).

**Nymphaea nouchali var. zanzibariensis (Casp.) Verdc.** Habit: Herb. Habitat: Lowland streams, also ponds on granitic rocks, ca. 0–650 m. Vouchers: SAJIT–005474 & 006231 (EA, HIB), Magogo & Glover 578, Magogo & Glover 155, Hildebrandt 1921 (EA).

**F121. Ochnaceae**

4 Genera, 12 Species

***Brackenridgea zanguebarica* Oliv.** Habit: Shrub. Habitat: Coastal bushland, wooded grassland, *Brachystegia* woodland, and lowland *Julbernadia* evergreen forest, ca. 0–200 m. Vouchers: Ngumbau V & Mwadime N V0499 & V095 (EA, HIB), Greenway 9802, Drummond & Hemsley 1107, Hildebrandt 1966, Reitsma J 225, Robertson SA 5071 (EA).

***Campylospermum reticulatum* (P. Beauv.) Farron** Habit: Shrub. Habitat: Evergreen forest, rainforest, and riverine forest, ca. 300 m. Vouchers: SAJIT–006065 (EA, HIB), Magogo & Glover 898, Luke & Robertson 2736, Robertson 7392 (EA).

***Campylospermum sacleuxii* (Tiegh.) Farron** Habit: Shrub. Habitat: Lowland evergreen forest, riverine forest, ground water forest, and semi-dry forest, 40–750 m. Vouchers: Spjut & Ensor 2728, Magogo & Glover 166, Luke 4542, Luke WRQ et al. 4716 (EA).

***Ochna apetala* Verdc.** Habit: Shrub. Habitat: Lowland rainforest, deciduous forest, and sometimes riverine, 5–200 m. Vouchers: Ngumbau V & Mwadime N V091 (EA, HIB), Robertson & Luke 6348, Luke & Robertson 1674 & 2639 (EA).

***Ochna atropurpurea* DC.** Habit: Shrub. Habitat: Various mixed thicket and bushland, 0–450 m. Vouchers: SAJIT–006041, Ngumbau V & Mwadime N V0276 (EA, HIB), Verdcourt 2347, Drummond & Hemsley 1013, Polhill & Paulo 592, Roberts SA & Luke Q 5584, Luke WRQ & Robertson SA 531 (EA).

***Ochna holstii* Engl.** Habit: Shrub. Habitat: Understorey of evergreen forest and among rocks, ca. 480 m. Vouchers: SAJIT–005985 (EA, HIB), Luke WRQ & Robertson SA 1010 (EA).

***Ochna holtzii* Gilg** Habit: Shrub. Habitat: *Brachystegia* woodland, thicket, and intermediate evergreen rainforest, 15–700 m. Vouchers: Luke & Robertson 2128, Swynnerton 16 & 10 (EA).

**Ochna kirkii subsp. multisetosa Verdc.** Habit: Shrub. Habitat: Semi deciduous forest and groundwater forest, 100–600 m. Vouchers: Drummond & Hemsley 4013, Greenway & Rawlins 9367, Rawlins 222, Luke Q 1411 (EA).

***Ochna macrocalyx* Oliv.** Habit: Shrub. Habitat: Coral rag forest, ca. 10 m. Vouchers: Luke & Mbinda 5847, Robertson SA & Luke Q 1557 (EA).

***Ochna* sp. 17 of KTSL** Habit: Shrub. Habitat: Thickets on dune above high water mark, 1–50 m. Vouchers: Gilbert & Kuchar 5874, Kuchar 13719, MacNaughton 2567, Oxtoby EA 15356 (EA): Endemic.

***Ochna thomasiana* Engl. & Gilg** Habit: Shrub. Habitat: Evergreen thicket, forest on coral rock, and coastal bushland, 0–400 m. Vouchers: SAJIT–005942 & 006244, Ngumbau V & Mwadime N V0323 (EA, HIB), Drummond & Hemsley 4154, Gardner in FD 1428, Robertson & Luke 5317, Magogo FC & Glover PE 12, Robertson SA & Beentje HJ 5631 (EA).

***Sauvagesia erecta* L.** Habit: Herb. Habitat: Grassland, 20–60 m. Vouchers: Nyange M & Luke Q 0548, Luke & Mbinda 5749, L & L 3795 (EA).

**F122. Olacaceae**

2 Genera, 2 Species

***Olax obtusifolia* De Wild.** Habit: Tree. Habit: Lowland forest, ca. 200 m. Vouchers: Luke WRQ & Robertson SA 2771, Luke, Stone & Baer 8244 (EA).

***Strombosiopsis pentamera* Q. Luke, ined.** Habit: Tree, 390 m. Habitat: Forest. Voucher: Luke WRQ & Robertson SA 2725 (EA): Endemic.

**F123. Oleaceae**

2 Genera, 7 Species

**Jasminum fluminense Vell. subsp. fluminense** Habit: Liana. Habitat: Forests, woodlands, bushlands, and grassland, ca. 210 m. Voucher: Wakefield (EA).

***Jasminum meyeri-johannis* Engl.** Habit: Liana. Habitat: Forest and bushland, ca. 690 m. Voucher: Battiscombe 261 (EA).

***Jasminum schimperi* Vatke** Habit: Liana. Habitat: Rainforests, wooded grassland, and bushland, ca. 400 m. Voucher: Luke WRQ 3157 (EA).

***Jasminum stenolobum* Rolfe** Habit: Liana. Habitat: *Brachystegia* woodland often on termite mounds, ca. 183 m. Vouchers: Ngumbau V & Mwadime N V0197 (EA, HIB), Malombe I, Mwadime N & Saidi 1653, Luke WRQ & Robertson SA 2782 (EA).

***Jasminum streptopus* E. Mey.** Habit: Liana. Habitat: Scrub and woodland ca. 100 m. Vouchers: Luke WRQ & Robertson SA 2798, Graham 2147 (EA).

**Olea europaea subsp. cuspidata (Wall. ex G. Don) Cif.** Habit: Tree. Habitat: Bushland, woodland, ca. 80 m. Voucher: Luke WRQ & Robertson SA 2497 (EA).

**Olea woodiana subsp. disjuncta P. S. Green** Habit: Tree. Habitat: Forest, ca. 238 m. Vouchers: Ngumbau V & Mwadime N V0185 (EA, HIB), Luke WRQ & Robertson SA 505 (EA).

**F124. Onagraceae**

1 Genus, 7 Species

***Ludwigia abyssinica* A. Rich.** Habit: Herb. Habitat: In swampy places, ca. 40 m. Vouchers: Luke WRQ & Robertson SA 2756, Nyange M 576 (EA).

**Ludwigia adscendens subsp. diffusa (Forssk.) P. H. Raven** Habit: Herb. Habitat: In wet places, ca. 36 m. Vouchers: SAJIT–006201 (EA, HIB), Sangai GW 15607 (EA).

***Ludwigia erecta* (L.) H. Hara** Habit: Herb. Habitat: Wet areas, ca. 30 m. Voucher: Gray M, PA & Luke Q 431 (EA).

***Ludwigia jussiaeoides* Desr.** Habit: Herb. Habitat: Swampy areas, ca. 25 m. Vouchers: Graham 2099, Joanna in CM 5977, Luke WRQ 15336 (EA).

***Ludwigia perennis* L.** Habit: Herb. Habitat: Swamps, flood plains of lakes, and probably in other wet habitats, ca. 0–30 m. Vouchers: Whyte, Festo L, Luke Q & P 2701 (EA).

**Ludwigia sp. aff. stolonifera (Guill. & Perr.) P. H. Raven** Habit: Herb. Habitat: Forest. Voucher: Luke WRQ & Robertson SA 2777 (EA).

**Ludwigia stenorraphe subsp. macrosepala (Brenan) P. H. Raven** Habit: Herb. Habitat: Forest, ca. 240 m. Voucher: Luke WRQ & Robertson SA 2766 (EA).

**F125. Opiliaceae**

2 Genera, 2 Species

***Opilia amentacea* Roxb.** Habit: Liana. Habitat: Coastal bushland and riverine forest to upland rainforest, ca. 0–300 m. Vouchers: Ngumbau V & Mwadime N V0270, V0426 & V041 (EA, HIB), Kimeu JM, Meso M, Ot 613, Magogo FC & Glover PE 1053, Verdcourt 3609 (EA).

***Pentarhopalopilia umbellulata* (Baill.) Hiepko** Habit: Liana. Habitat: Coastal forest, *Brachystegia* woodland, and coastal bushland, 0–100 m. Vouchers: Ngumbau V & Mwadime N V0498 (EA, HIB), RM Graham in FD1830 & 2301, Gray M & Luke WRQ 341 (EA).

**F126. Orchidaceae**

24 Genera, 61 Species

***Acampe pachyglossa* Rchb.f.** Habit: Herb. Habitat: Epiphytic on trees in deciduous and coastal bushland, 0–800 m. Vouchers: Ngumbau V & Mwadime N V0202 (EA, HIB), Copley 25, Dale in FD 3773, Tweedie 634, Tweedie EM 87 (EA).

***Aerangis hologlottis* (Schltr.) Schltr.** Habit: Herb. Habitat: Epiphytic on twigs and small branches of trees in coastal forests, 0–500 m. Vouchers: Castelino in EAH 18/63, Archer 522, Luke WRQ 1640 (EA).

***Aerangis kirkii* (Rchb.f.) Schltr.** Habit: Herb. Habitat: Coastal bush and riverine forest at low altitudes inland, on small trees, and bushes, 0–450 m. Vouchers: Ngumbau V & Mwadime N V0139 (EA, HIB), Magogo & Glover 955, RM Graham B 442 & FD 2146, Rawlins SP 11259 (EA).

***Aerangis kotschyana* (Rchb.f.) Schltr.** Habit: Herb. Habitat: Wooded grassland, woodland, and sometimes in forest, 0–200 m. Vouchers: WE Taylori, Musyoki BM & Hansen OJ 963, Robertson SA & Luke WRQ 4850 (EA).

***Angraecum cultriforme* Summerh.** Habit: Herb. Habitat: *Brachystegia* woodland, dry evergreen forest, and evergreen bushland, ca. 0–400 m. Vouchers: Boscawen in Moreau 724, Robertson SA & Luke WRQ 2450 (EA).

***Angraecum dives* Rolfe** Habit: Herb. Habitat: Coastal woodland, evergreen forest, scrub, and on exposed coral rocks, 0–50 m. Vouchers: SAJIT–006460 & 005591 (EA, HIB), Irwin 92, Polhill & Paulo 691, Rathbun in EAH 14989, Muchiri J 506 (EA).

**Angraecum eburneum subsp. giryamae (Rendle) Senghas & P.J. Cribb** Habit: Epiphytic or lithophytic herb. Habitat: Thickets on outcropping rocks, on rocks by sea, and on cliff faces, ca. 0–350 m. Vouchers: Drummond & Hemsley 4181, Greenway 4969, Dale in FD 3566, Jeffery GW 699 (EA).

***Angraecum teres* Summerh.** Habit: Epiphytic herb. Habitat: Epiphytic on bole of *Cedrela odorata*, and on small trees by the sea, 0–600 m. Voucher: Luke WRQ & Robertson SA 2661 (EA).

***Angraecum viride* Kraenzl.** Habit: Herb. Habitat: In rainforest, ca. 150 m. Voucher: Luke WRQ 1848 (EA).

***Ansellia africana* Lindl.** Habit: Epiphytic herb. Habitat: Epiphytic or rarely on rocks in open woodland, wooded grassland, and on *Hyphaene* palm trees, ca. 0–381 m. Vouchers: Ngumbau V & Mwadime N V0545 (EA, HIB), Magogo FC & Glover PE 1078, Reitsma J 495, Copley 123 (EA): Vulnerable.

***Bolusiella* sp.** Habit: Epiphyte. Habitat: Forest. Voucher: Luke WRQ 5270 (EA).

***Bonatea rabaiensis* (Rendle) Rolfe** Habit: Herb. Habitat: Lowland dry evergreen forest on sand or evergreen bushland, ca. 0–304 m. Vouchers: Jeffery GW 238, Luke WRQ & PA 4507 (EA).

***Bulbophyllum intertextum* Lindl.** Habit: Herb. Habitat: Epiphytic in rainforest, riverine, and moist forest, ca. 300 m. Voucher: Boscawen in Moreau 99 (EA).

***Bulbophyllum maximum* (Lindl.) Rchb. f.** Habit: Herb. Habitat: Epiphyte in open woodland and riverine forests, ca. 0–365 m. Vouchers: Luke WRQ 1580, Magogo FC & Glover PE 582, Rawlings 763 (EA).

***Bulbophyllum scaberulum* (Rolfe) Bolus** Habit: Herb. Habitat: Epiphyte in riverine and montane forests, ca. 100–250 m. Vouchers: Cunningham van Someren in Moreau 414, Greenway 9807, Luke WRQ & Robertson SA 2621, Robertson SA & Luke WRQ 6013 (EA).

***Calyptrochilum christyanum* (Rchb. f.) Summerh.** Habit: Herb. Habitat: Forest, woodland, and grassland, ca. 300 m. Vouchers: Kirk 12, Malombe I, Mwadime N & Saidi 1669 (EA).

***Cynorkis kirkii* Rolfe** Habit: Herb. Habitat: On rocky seepage slopes, usually in shade, ca. 326 m. Voucher: Luke WRQ & Pakia M 5278 (EA).

***Cyrtorchis arcuata* (Lindl.) Schltr.** Habit: Herb. Habitat: Woodland and forest, ca. 0–800 m. Vouchers: Verdcourt & Bayliss 2413, Jeffery GW 806, Robertson SA & Luke WRQ 2064 (EA).

**Disperis aphylla Kraenzl. subsp. aphylla** Habit: Herb. Habitat: Evergreen forest, ca. 380 m. Vouchers: CG van Someren Sh 8, Magogo FC & Glover PE 981 (EA).

***Eulophia angolensis* (Rchb. f.) Summerh. var. angolensis** Habit: Herb. Habitat: Swampy area, ca. 20 m. Vouchers: Dale IR 3548, Luke WRQ & PA 3798 (EA).

***Eulophia cucullata* (Afzel. ex Sw.) Steud.** Habit: Herb. Habitat: Grassland, wooded grassland, bushland, and woodland, ca. 100–200 m. Vouchers: SAJIT–005520 (EA, HIB), Lucas, Jeffery & Kirrika 246, Hawthorne W 109, Luke WRQ 3155 (EA).

***Eulophia horsfallii* (Bateman) Summerh.** Habit: Herb. Habitat: Riverine, evergreen forest, and along streams, ca. 30 m. Vouchers: Bally PRO & Smith 14360, Luke WRQ & PA 3954 (EA).

***Eulophia livingstoneana* (Rchb. f.) Summerh.** Habit: Herb. Habitat: Grassland or in deciduous woodland of *Brachystegia*, ca. 0–442 m. Vouchers: Magogo & Glover 851, Magogo FC & Glover PE 203 (EA).

***Eulophia petersii* (Rchb. f.) Rchb. f.** Habit: Herb. Habitat: Thickets, and bushland, ca. 0–330 m. Vouchers: SAJIT–006239 (EA, HIB), Drummond & Hemsley 4083 & 4232, Luke WRQ 1081 (EA).

***Eulophia schweinfurthii* Kraenzl.** Habit: Herb. Habitat: In grassland, thicket, bushland, and woodland, ca. 40 m. Vouchers: Kassner 359, Luke WRQ 3097 (EA).

***Eulophia serrata* P. J. Cribb** Habit: Herb. Habitat: *Commiphora*-*Acacia* bushland, ca. 150–200 m. Vouchers: Robertson SA 5198 & 4037B (EA): Endemic.

***Eulophia speciosa* (R. Br.) Bolus** Habit: Herb. Habitat: In grassland, deciduous bushland, and woodland, ca. 0–335 m. Vouchers: Ngumbau V & Mwadime N V0199 (EA, HIB), Verdcourt 1191, Luke WRQ & Robertson SA 1816, Magogo FC & Glover PE 480 (EA).

***Eulophia taitensis* Pfennig & P. J. Cribb** Habit: Herb. Habitat: Thickets, coastal bushland, and woodland. Voucher: RM Graham 388 (EA).

***Habenaria armatissima* Rchb. f.** Habit: Herb. Habitat: Forest, open marshy ground, deciduous thicket, and mixed dry woodland, ca. 20 m. Vouchers: Jeffery 247, Rawlins SP 806 (EA).

***Habenaria boiviniana* Kraenzl. & Schltr.** Habit: Herb. Habitat: At margins of lowland rainforest and coastal evergreen bushland, up to 400 m. Voucher: Drummond & Hemsley 3967 (EA).

***Habenaria kilimanjari* Rchb. f.** Habit: Herb. Habitat: Periodically flooded grassland, often with scattered bushes, ca. 90–152 m. Vouchers: RM Graham in FD 1596, Dale in FD 3547, Jex-Blake in CM 5996, Dale IR 3547 (EA).

***Habenaria plectromaniaca* Rchb.f. & S. Moore** Habit: Herb. Habitat: Marshy grassland or at edge of forests, 30–420 m. Vouchers: RM Graham in FD 1593 & 1934, Adams BR 33, Luke WRQ 3317 (EA).

**Habenaria stylites Rchb. f. & S. Moore subsp. stylites** Habit: Herb. Habitat: Grassland, lowland rainforest, and wooded grassland, ca. 100–335 m. Vouchers: GM 11200, Cunningham-van Someren GR Sh 17 (EA): Endangered.

***Habenaria subarmata* Rchb. f.** Habit: Herb. Habitat: Grassland or open woodland, ca. 5 m. Vouchers: Rawlins in EAH 11258, Festo 2550 (EA).

***Habenaria trilobulata* Schltr.** Habit: Herb. Habitat: Open woodland and lowland dry evergreen forest, ca. 304 m. Vouchers: Ngumbau V & Mwadime N V092 (EA, HIB), Drummond & Hemsley 3809, Tweedie 635 (167), Luke WRQ & Robertson SA 1794, Luke WRQ 3116 (EA).

***Jumellea filicornoides* (De Wild.) Schltr.** Habit: Herb. Habitat: Riverine forest, savannah trees, and on rocks, ca. 600 m. Vouchers: Luke & Robertson 1826, Luke WRQ & Robertson SA 1826 (EA).

***Microcoelia aphylla* (Thouars) Summerh.** Habit: Epiphyte. Habitat: In thickets of *Acacia*-*Commiphora*, in scrub, and riverine forest on *Barringtonia racemosa*, ca. 65 m. Vouchers: SAJIT–006436 (EA, HIB), RM Graham 1637 (EA).

***Microcoelia exilis* Lindl.** Habit: Herb. Habitat: In riverine forest and woodland, ca. 0–800 m. Vouchers: Drummond & Hemsley 3890, Polhill & Paulo 636, Rawlins SP 11268 (EA).

***Microcoelia megalorrhiza* (Rchb. f.) Summerh.** Habit: Herb. Habitat: Woodland and riverine thicket, ca. 100–500 m. Vouchers: Luke WRQ & Robertson SA 1762 & 2022, Robertson SA, Beentje HJ, Luke WRQ & Khayota B268 (EA).

***Microcoelia obovata* Summerh.** Habit: Herb. Habitat: Woodland or dry riverine forest, ca. 0–500 m. Vouchers: Saunders PJR in EAH 11247, Luke WRQ & Robertson SA 2680 & 2018 (EA).

***Microcoelia physophora* (Rchb. f.) Summerh.** Habit: Herb. Habitat: Coastal bushland and woodland, 0–500 m. Vouchers: JB Smart s.n., Luke WRQ 3480 (EA).

***Microcoelia smithii* (Rolfe) Summerh.** Habit: Herb. Habitat: Evergreen forest, on *Euphorbia* sp, 0–500 m. Vouchers: WE Taylor s.n., Beentje HJ 2309 (EA).

***Nervilia bicarinata* (Blume) Schltr.** Habit: Herb. Habitat: Evergreen forest, ca. 7 m. Vouchers: Luke WRQ 2404, Bytebier B 630 (EA).

***Nervilia crociformis* (Zoll. & Moritzi) Seidenf.** Habit: Herb. Habitat: Evergreen forest, grassland, and riverine forest, ca. 150 m. Voucher: Luke WRQ 3129 (EA).

**Nervilia kotschyi (Rchb. f.) Schltr. var. kotschyi** Habit: Herb. Habitat: Scrub and in shady places in deciduous woodland, ca. 0–442 m. Vouchers: Ngumbau V & Mwadime N V037 (EA, HIB), Magogo & Glover 265, Adams BR 28, Luke WRQ 3596 (EA).

***Nervilia petraea* (Afzel. ex Sw.) Summerh.** Habit: Herb. Habitat: *Brachystegia* woodland, grassland, and pine plantations, ca. 381 m. Vouchers: Luke WRQ 3119, Magogo FC & Glover PE 1112 & 560 (EA).

***Oeceoclades maculata* (Lindl.) Lindl.** Habit: Herb. Habitat: Dry and moist habitats including evergreen deciduous forests, ca. 0–250 m. Vouchers: Luke WRQ & PA 4506, Luke WRQ & Robertson SA 6007 (EA).

***Oeceoclades saundersiana* (Rchb. f.) Garay & P. Taylor** Habit: Herb. Habitat: Forest and thickets, ca. 0–280 m. Vouchers: Ngumbau V & Mwadime N V015 (EA, HIB), Forestry Dept., Archr 411, Castelino H347/62, Robertson SA & Luke WRQ 6007, Evans M 15122 (EA).

***Oeceoclades zanzibarica* (Summerh.) Garay & P. Taylor** Habit: Terrestrial or lithophtic herb. Habitat: Thickets, ca. 5 m. Vouchers: Archer PG 728, Mwadime N & Cherise C 229 (EA).

***Platycoryne crocea* Rolfe** Habit: Herb. Habitat: Grassland, 0–360 m. Vouchers: CG van Someren Sh 4, Jeffery 629, Luke WRQ & Pakia M 7460A (EA).

***Polystachya concreta* (Jacq.) Garay & H. R. Sweet** Habit: Herb. Habitat: Rainforest, ca. 0–280 m. Vouchers: SAJIT–005431, Ngumbau V & Mwadime N V013 (EA, HIB), Piers 62/29, Luke WRQ 1597 (EA).

***Polystachya cultriformis* (Thouars) Lindl. ex Spreng.** Habit: Herb. Habitat: Evergreen forest, ca. 335 m. Voucher: Bayliss RDA H93/57/2 (EA).

***Polystachya fischeri* Rchb. f. ex Kraenzl.** Habit: Herb. Habitat: Epiphytic in dry evergreen forest, ca. 310 m. Vouchers: WE Taylor, Pearce TR 616 (EA).

***Polystachya lindblomii* Schltr.** Habit: Herb. Habitat: Sub montane, riverine evergreen forest, and high rainfall woodland, ca. 69 m. Voucher: Miyawa DO 1155 (EA).

***Polystachya modesta* Rchb. f.** Habit: Herb. Habitat: In riverine forest and woodland, ca. 470 m. Vouchers: Bayliss RDA 3, Robertson SA 5806 (EA).

***Polystachya teitensis* P. J. Cribb** Habit: Herb. Habitat: Forest, ca. 700 m. Vouchers: SAJIT–005434 (EA, HIB), Luke WRQ & Robertson SA 2049 (EA): Endangered.

***Rhipidoglossum rutilum* (Rchb. f.) Schltr.** Habit: Herb. Habitat: In forests, ca. 100 m. Voucher: Luke WRQ & Robertson SA sr.

***Solenangis wakefieldii* (Rolfe) P. J. Cribb & J. Stewart** Habit: Herb. Habitat: In coastal and lowland bushland, 0–300 m. Vouchers: SAJIT–005426 (EA, HIB), Wakefield, Archer PG 447 (EA).

***Tridactyle bicaudata* (Lindl.) Schltr.** Habit: Herb. Habitat: In forest and woodland areas, ca. 600 m. Vouchers: Irwin PH 497, Luke WRQ et al. 7083, Luke WRQ & Robertson SA 2035 (EA).

***Vanilla ramosa* Rolfe** Habit: Climber. Habitat: Moist forest, ca. 280 m. Vouchers: Ngumbau V & Mwadime N V010 (EA, HIB), Luke WRQ 3047 (EA).

***Vanilla roscheri* Rchb. f.** Habit: Climber. Habitat: Coastal bushland, coral rock, mangrove swamps, and open evergreen inland scrub, 0–750 m. Vouchers: SAJIT–006445 (EA, HIB), Bally 10462, Verdcourt 3223, Polhill & Paulo 876 (EA).

**F127. Orobanchaceae**

11 Genera, 21 Species

***Alectra orobanchoides* Benth.** Habit: Herb. Habitat: In open woodland, scrub, rocky areas, and cultivated land, ca. 220 m. Voucher: Luke WRQ & Robertson SA 2765 (EA).

***Alectra picta* (Hiern) Hemsl.** Habit: Herb. Habitat: Riverine *Brachystegia* woodland, amongst shrubs, and sedges at edges of river, ca. 30 m. Vouchers: Robertson & Luke 6213, Luke 1332 (EA).

***Alectra sessiliflora* (Vahl) Kuntze** Habit: Herb. Habitat: Grassland and cultivation areas, ca. 400 m. Vouchers: Luke 1332, Drummond & Hemsley 3970 (EA).

***Buchnera hispida* Buch.-Ham. ex D. Don** Habit: Herb. Habitat: Grassland and woodland, ca. 0–100 m. Vouchers: Drummond & Hemsley 3741, Rawlins SP 65 (EA).

***Buchnera leptostachya* Benth.** Habit: Herb. Habitat: In wet grassland, swamps, and coastal bush, 0–500 m. Vouchers: Swynnerton 403, Graham 2115, Festo L, Luke Q & P 2810 (EA).

***Buttonia natalensis* McKen ex Benth.** Habit: Liana. Habitat: Open woodlands, among rocks, and on riverbanks, ca. 30–518 m. Voucher: Robertson SA & Luke WRQ 6061 (EA).

***Cistanche tinctoria* (Forssk.) Deflers** Habit: Herb. Habitat: Dry stony ground or semi desert scrub, ca. 20 m. Vouchers: Verdcourt 1100, Taiti S 542 (EA).

**Cycnium adonense E. Mey. ex Benth. subsp. adonense** Habit: Herb. Habitat: Grassland and grassland woodland, ca. 0–360 m. Vouchers: Drummond & Hemsley 1192, Magogo FC & Glover PE 26, Festo L, Luke Q & P 2651 (EA).

***Cycnium tubulosum* (L. f.) Engl.** Habit: Herb. Habitat: Damp grassland, along marshes, and river banks, ca. 5 m. Voucher: Festo L & Luke Q 2551 (EA).

***Cycnium veronicifolium* (Vatke) Engl.** Habit: Herb. Habitat: Grassland, wooded grassland, and bushland, ca. 0–300 m. Vouchers: CW Elliot in FD 3373, Robertson SA & Luke WRQ 6054 (EA).

***Ghikaea speciosa* (Rendle) Diels** Habit: Herb. Habitat: Dry grassland and *Acacia*-*Commiphora* scrub, ca. 350 m. Voucher: van Someren 878 (EA).

***Harveya kenyensis* Hepper** Habit: Herb. Habitat: Dry forests, 30–450 m. Voucher: Robertson SA & Luke WRQ 6065 (EA).

***Harveya obtusifolia* (Benth.) Vatke** Habit: Herb. Habitat: Thickets, bushland, fields, and orchards, ca. 350 m. Vouchers: Drummond & Hemsley 3865, Jeffery GW 268 (EA).

***Micrargeria filiformis* (Schumach. & Thonn.) Hutch. & Dalziel** Habit: Herb. Habitat: Swamps, damp and wet areas in grassland, and seepages in woodland, ca. 0–20 m. Vouchers: Greenway 4963, Joy Adamson in Bally B 6141, Luke WRQ 15342 (EA).

***Nesogenes africanus* G. Taylor** Habit: Herb. Habitat: In shade of bushes on limestone rock, ca. 10 m. Voucher: Robertson SA 5805 (EA).

***Pseudosopubia hildebrandtii* Engl.** Habit: Herb. Habitat: Grassland, grassland with trees, rocky, river, and swamp margins, ca. 0–100 m. Vouchers: Drummond & Hemsley 3748, Rawlins SP 801 (EA).

***Striga asiatica* (L.) Kuntze** Habit: Herb. Habitat: Semi-parasitic on roots of wild grasses and cultivated maize, ca. 0–365 m. Vouchers: SAJIT–006051, Ngumbau V & Mwadime N V0341 (EA, HIB), Drummond & Hemsley 3839, Burstyn P 75/77, Magogo FC & Glover PE 1089 (EA).

***Striga forbesii* Benth.** Habit: Herb. Habitat: Low lying grassy places, ca. 10–70 m. Vouchers: Gilbert & Kibuwa 19932, Festo L, Luke Q & P 2711, Luke WRQ 3489 (EA).

***Striga gesnerioides* (Willd.) Vatke** Habit: Herb. Habitat: In permanent grassland parasitizing grass roots, ca. 20 m. Voucher: Polhill & Paulo 579 (EA).

***Striga latericea* Vatke** Habit: Herb. Habitat: Seasonally wet places among grass, ca. 100 m. Vouchers: Drummond & Hemsley 4247, Archer PG 445, Magogo FC & Glover PE 1050 (EA).

***Striga pubiflora* Klotzsch** Habit: Herb. Habitat: Woodland among grass, in salt marshes, and on roots of grasses, ca. 100–304 m. Vouchers: Hildebrandt 1907, Moomaw 953, Magogo & Glover 849, Thomas M 23 (EA).

**F128. Oxalidaceae**

2 Genera, 4 Species

***Biophytum umbraculum* Welw.** Habit: Herb. Habitat: *Brachystegia* woodland, open grassland, and marsh edges, ca. 0–381 m. Vouchers: Ngumbau V & Mwadime N V0346 (EA, HIB), Jeffery 342, Magogo FC & Glover PE 951, Rawlins SP 66 (EA).

***Oxalis barrelieri* L.** Habit: Herb. Habitat: Weed of roadsides and cultivated areas, ca. 100 m. Voucher: Robertson SA 3389 (EA): Naturalized.

***Oxalis corniculata* L.** Habit: Herb. Habitat: Arable land and waste places, 0–396 m. Vouchers: Magogo FC & Glover PE 667 & 496 (EA): Naturalized.

***Oxalis latifolia* Kunth** Habit: Herb. Habitat: Forests and forest edges, arable fields, and waste places. Voucher: Jeffery GW 527 (EA).

**F129. Pandanaceae**

1 Genus, 2 Species

***Pandanus kirkii* Rendle** Habit: Tree. Habitat: Sandy beaches and on coral near the sea, 0–10 m. Vouchers: Greenway 9642, St. John 26594, Beentje 3737 (EA).

***Pandanus rabaiensis* Rendle.** Habit: Tree. Habitat: Riverine forest or along streams in evergreen forest, 1–100 m. Vouchers: Agnew 9742, St. John 26596, Dominic U 16189, Luke WRQ & Robertson SA 2827 (EA).

**F130. Papaveraceae**

1 Genus, 1 Species

***Argemone mexicana* L.** Habit: Herb. Habitat: Open ground or along the roads, ca. 0–200 m. Voucher: Robertson SA 3774 (EA): Naturalized.

**F131. Passifloraceae**

7 Genera, 19 Species

**Adenia aculeata (Oliv.) Engl. subsp. aculeata** Habit: Liana. Habitat: Dry forests, ca. 20 m. Voucher: Gilbert MG & Kuchar P 5870 (EA).

**Adenia aculeata subsp. manganiana (Chiov.) W.J.de Wilde** Habit: Liana. Habitat: Coastal scrub, 0–100 m. Voucher: Bally 5951 (EA).

***Adenia angulosa* G.W. Hu & Q.F. Wang** Habit: Liana. Habitat: Coastal moist forest, forest margin, ca. 350 m. Vouchers: SAJIT–005956, 006061, 006074, 006078, 005975 & 006156, Ngumbau V & Mwadime N V0171 (EA, HIB).

**Adenia globosa Engl. subsp. globosa** Habit: Liana. Habitat: Deciduous and dry evergreen bushland, often in rocky places, ca. 100 m. Vouchers: Drummond & Hemsley 4053, Ndakala J 363, Graham RM 863 (EA).

***Adenia cissampeloides* (Planch. ex Hook.) Harms** Habit: Liana. Habitat: Forest and bushland of various types, ca. 0–442 m. Vouchers: SAJIT–005959, 006083 & 006209, Ngumbau V & Mwadime N V0268 (EA, HIB), RM Graham A 494 in FD1873, Robertson SA & Luke WRQ 5626 (EA).

***Adenia keramanthus* Harms** Habit: Herb. Habitat: Deciduous woodland, bushland, dry evergreen, and coastal bushland, ca. 0–330 m. Vouchers: Drummond & Hemsley 4233, Luke WRQ & Robertson SA 1551 (EA).

***Adenia kirkii* (Mast.) Engl.** Habit: Liana. Habitat: Lowland evergreen forest and grassland, 0–700 m. Vouchers: SAJIT–006429, Ngumbau V & Mwadime N V0441 & V005 (EA, HIB), Rawlins 891, Jeffery K 569, Rawlins 292, Robertson SA & Luke WRQ 5375 (EA).

***Adenia lindiensis* Harms** Habit: Liana. Habitat: Edges of evergreen forest and bushland, 0–304 m. Vouchers: VG van Someren 87, Robertson SA & Luke WRQ 5170 (EA).

**Adenia lobata subsp. rumicifolia (Engl. & Harms) Lye** Habit: Liana. Habitat: In moister places, in forest edges, semi swamp forest, riverine forest, and thicket, 0–320 m. Vouchers: SAJIT–006008, Ngumbau V & Mwadime N V0522 (EA, HIB), VG van Someren 97, Mwadime N 29, Luke WRQ 3107, Luke WRQ 1599 (EA).

***Adenia venenata* Forssk.** Habit: Liana. Habitat: Wooded grassland, deciduous woodland, and bushland, ca. 20 m. Vouchers: Rawlins 109, Rawlins SP 372 (EA).

***Basananthe hanningtoniana* (Mast.) W.J.de Wilde** Habit: Climber. Habitat: Wide variety of open habitats and deciduous bushland, ca. (0–) 400 m. Vouchers: Rawlins SP 340, Magogo FC & Glover PE 852 (EA).

***Basananthe lanceolata* (Engl.) W. J.de Wilde** Habit: Climber. Habitat: Grassland, bushland, and disturbed places, 0–100 m. Vouchers: Graham RM 2022, Kuchar P 11753, Drummond & Hemsley 3856 (EA).

***Basananthe zanzibarica* (Mast.) W.J.de Wilde** Habit: Climber. Habitat: Lowland dry evergreen forest and coastal bushland, 0–450 m. Vouchers: SAJIT–006039, Ngumbau V & Mwadime N V070 (EA, HIB), Drummond & Hemsley 3956, Napper 1373, Verdcourt 3924, Festo L, Luke Q & P 2716, Magogo FC & Glover PE 438 (EA).

***Loewia tanaensis* Urb.** Habit: Shrub. Habitat: Woodland, ca. 280 m. Voucher: Mwadime N & Luke WRQ 2436 (EA).

***Passiflora foetida* L.** Habit: Climber. Habitat: Thickets, ca. 10–15 m. Vouchers: Luke WRQ 3059 & 3069 (EA): Naturalized.

***Schlechterina mitostemmatoides* Harms** Habit: Liana. Habitat: Lowland dry evergreen, riverine forest, and coastal bushland, 0–700 m. Vouchers: SAJIT–005998 & 006430, Ngumbau V & Mwadime N V0213 (EA, HIB), Magogo & Glover 287, Dale in FD 3643, Rawlins in EAH 11269, Muchiri J 451 (EA).

***Turnera thomasii* (Urb.) Story** Habit: Shrub. Habitat: Dry sandy places, ca. 25 m. Vouchers: Mwadime N & Luke WRQ 2435, F Thomas 47 (EA): Endemic.

**Tricliceras brevicaule (Urb.) R. Fern. var. brevicaule** Habit: Herb. Habitat: Wooded grassland, ca. 200 m. Vouchers: Luke WRQ et al. 3623 & 3839 (EA).

***Tricliceras xylorhizum* Verdc.** Habit: Herb. Habitat: *Acacia*-*Commiphora* bushland, ca. 230 m. Voucher: Luke & Robertson 2516 (EA).

**F132. Pedaliaceae**

4 Genera, 6 Species

***Dicerocaryum zanguebarium* (Lour.) Merr.** Habit: Herb. Habitat: Coastal dune, 0–500 m. Vouchers: SAJIT–006250 (EA, HIB), Luke Q 6143 (EA).

***Pedalium murex* L.** Habit: Herb. Habitat: On limestone in short grass near the coast, old sisal plantations, 0–440 m. Vouchers: SAJIT–004678 (EA, HIB), Napier 3265 (EA).

***Sesamothamnus busseanus* Engl.** Habit: Shrub. Habitat: *Acacia*-*Commiphora* scrub, ca. 300 m. Voucher: Bally 4375 (EA).

***Sesamum angolense* Welw.** Habit: Herb. Habitat: Disturbed grassland and roadsides, ca. 215 m. Voucher: SAJIT–005939 (EA, HIB).

***Sesamum angustifolium* (Oliv.) Engl.** Habit: Herb. Habitat: In cultivated, waste areas, native gardens, roadsides, and short grassland, ca. (15–) 600 m. Voucher: Battiscombe 258 (EA).

***Sesamum indicum* L.** Habit: Herb. Habitat: Grassland and roadsides, ca. 10 m. Voucher: Jeffery K 24 (EA).

**F133. Peraceae**

1 Genus, 1 Species

**Clutia abyssinica var. usambarica Pax & K. Hoffm.** Habit: Shrub. Habitat: Dry evergreen forest, ca. 300 m. Voucher: Cunningham-van Someren GR Sh 84 (EA).

**F134. Phyllanthaceae**

10 Genera, 30 Species

***Antidesma membranaceum* Müll. Arg.** Habit: Shrub. Habitat: Rainforest, drier evergreen forest, thickets, and wooded grassland, ca. 10–410 m. Vouchers: Gillett 18669, Wakefield (EA).

***Antidesma venosum* E. Mey. ex Tul.** Habit: Shrub. Habitat: In riverine vegetation, woodland, and wooded grassland, ca. 0–237 m. Vouchers: SAJIT–005949, 005499 & 005560, Ngumbau V & Mwadime N V0173 (EA, HIB), Luke WRQ & PA 238 (EA).

***Antidesma vogelianum* Müll. Arg.** Habit: Shrub. Habitat: Rainforest and drier evergreen forest, ca. 0–187 m. Vouchers: Ngumbau V & Mwadime N V089 (EA, HIB), Robertson SA & Luke WRQ 5160 (EA).

***Bridelia atroviridis* Müll. Arg.** Habit: Tree. Habitat: Forest edges, associated bushland, thicket, and rivers, ca. 70–300 m. Vouchers: SAJIT–006011, Ngumbau V & Mwadime N V076 & 0172 (EA, HIB), Drummond & Hemsley 4025, Faden 70/255 (EA).

***Bridelia cathartica* Bertol.** Habit: Shrub. Habitat: Woodland, bushland, and thicket, ca. 0–300 m. Vouchers: SAJIT–006106 & 004660, Ngumbau V & Mwadime N V0191 (EA, HIB), Magogo & Glover 1067, Polhill & Paulo 746, Gillespie 146, Gray M & Luke WRQ 342 (EA).

***Bridelia micrantha* (Hochst.) Baill.** Habit: Tree. Habitat: Evergreen forest, associated bushland, thickets, along rivers, lakes, and swamps, ca. 50–400 m. Vouchers: Magogo & Glover 165, Luke WRQ & Robertson SA 228 (EA).

***Cleistanthus beentjei* Q. Luke, ined.** Habit: Tree. Habitat: Moist forest, 30–500 m. Vouchers: Luke WRQ & Robertson SA 2684 & 1934, Robertson SA & Luke WRQ 6343A (EA).

***Cleistanthus schlechteri* (Pax) Hutch.** Habit: Tree. Habitat: Lowland dry evergreen forest and riverine, 0–760 m. Vouchers: Faden & Faden 77/737, Graham RM 1652 (EA).

***Cyathogyne usambarensis* (Verdc.) J. Léonard** Habit: Shrub. Habitat: Lowland dry evergreen forest and bushland, 200–244 m. Vouchers: SAJIT–004656, Ngumbau V & Mwadime N V0398 (EA, HIB), Faden & Evans 70/797, Lavranos & Newton 12292, Festo L, Luke Q & P 2765, Thomas Mwadime in Mrs SA Robertson 7792, Luke WRQ 3144 (EA).

**Flueggea virosa (Roxb. ex Willd.) Royle subsp. virosa** Habit: Shrub. Habitat: Locally common in wide associations, mainly forest edges, bushland, and thickets, 0–230 m. Vouchers: Musyoki & Hansen 961, Magogo FC & Glover PE 295 (EA).

**Margaritaria discoidea var. nitida (Pax) Radcl.-Sm**. Habit: Tree. Habitat: Dry evergreen forest, deciduous woodland, thickets, and disturbed places, ca. 20–237 m. Vouchers: Ngumbau V & Mwadime N V0186 (EA, HIB), Magogo & Glover 678, Luke Q 1461(EA).

**Margaritaria discoidea var. triplosphaera Radcl.**-Sm. Habit: Tree. Habitat: Dry evergreen forest and disturbed places, 9–300 m. Vouchers: Kassner 146, Drummond & Hemsley 1140 (EA).

**Meineckia fruticans (Pax) G. L. Webster var. fruticans** Habit: Shrub. Habitat: Forest and coastal bushland, 6–200 m. Vouchers: SAJIT–006081, Ngumbau V & Mwadime N V0420 (EA, HIB), Gillett et al. 14645, Faden RB 70/433, Luke Q 1500 (EA).

**Meineckia fruticans var. engleri (Pax) G. L. Webster** Habit: Shrub. Habitat: On lowland wet evergreen and riparian forest, 30–220 m. Vouchers: Faden & Evans 70/735, Verdcourt 1078 (EA).

***Phyllanthus amarus* Schumach. & Thonn.** Habit: Herb. Habitat: Cultivated ground and disturbed places, ca. 2 m. Vouchers: Verdcourt 3908, Tweedie 1245, Sangai GW in EA 15752 (EA).

***Phyllanthus chevalieri* Beille** Habit: Herb. Habitat: On black clay soil, in grassland, flood plain grassland, and *Acacia* woodland, ca. 15–200 m. Vouchers: Polhill & Paulo 509, Luke 4620 (EA).

***Phyllanthus harrisii* Radcl.-Sm.** Habit: Herb. Habitat: Waste ground, swamps, rice fields, dry stream beds, riverine forest, and old cultivations, 0–150 m. Vouchers: SAJIT–005992, Ngumbau V & Mwadime N V0551 (EA, HIB), Tweedie 1041, Rawlins 674, Hooper & Townsend 1198, Magogo FC & Glover PE 363 (EA).

***Phyllanthus kaessneri* Hutch.** Habit: Shrub. Habitat: Coastal bushland, *Brachystegia* woodland, shady evergreen forest, and disturbed places, 0–90 m. Voucher: Polhill & Paulo 774 (EA).

***Phyllanthus leucocalyx* Hutch.** Habit: Herb. Habitat: Open or rocky generally damp places in a variety of associations, disturbed places, ca. 0–407 m. Vouchers: SAJIT–006047 (EA, HIB), Magogo & Glover 245, Gillespie 287, RM Graham 1726 (EA).

**Phyllanthus maderaspatensis L. var. maderaspatensis** Habit: Herb. Habitat: Deciduous woodland, bushland, wooded grassland places, cultivated, and disturbed places, ca. 0–300 m. Vouchers: Magogo & Glover 1057, Festo L & Luke Q 2470 (EA).

***Phyllanthus mittenianus* Hutch.** Habit: Shrub. Habitat: Moist forest, ca. 300 m. Vouchers: SAJIT–005984 (EA, HIB), Luke WRQ & PA 5404 (EA).

***Phyllanthus nummulariifolius* Poir.** Habit: Herb. Habitat: Woodland, wooded grassland, forest edges, extending to upland grassland, and bushland, 0–382 m. Vouchers: SAJIT–006073 (EA, HIB), Mwangangi 1282, Luke WRQ & Robertson SA 2749 (EA).

***Phyllanthus ovalifolius* Forssk.** Habit: Shrub. Habitat: Deciduous woodlands, thickets, and evergreen rainforest, ca. 86 m. Vouchers: Ngumbau V & Mwadime N V0470 (EA, HIB), Luke Q 1564 (EA).

***Phyllanthus pinnatus* (Wight) G.L. Webster** Habit: Shrub. Habitat: Coastal, lowland mixed bushland, wooded grassland, and sometimes riverine, 0–125 m. Voucher: Polhill & Paulo 685 (EA).

***Phyllanthus physocarpus* Müll.Arg.** Habit: Shrub. Habitat: Evergreen forest, ca. 450 m. Vouchers: Magogo & Glover 260, Spjut 4553, Cain in EAH. 13663, Luke WRQ & Robertson SA 503 (EA).

**Phyllanthus reticulatus Poir. var. reticulatus** Habit: Shrub. Habitat: Forest edges, riverine, maritime, edges of swamps, and lakes, 0–213 m. Vouchers: Ngumbau V & Mwadime N V0327 (EA, HIB), Sangai 962 (EA).

**Phyllanthus reticulatus var. glaber (Thwaites) Müll.Arg.** Habit: Shrub. Habitat: Coastal forests, 0–600 m. Vouchers: Bally 5790, Drummond & Hemsley 1022, Verdcourt 2111, Luke WRQ et al. 5412 (EA).

***Phyllanthus somalensis* Hutch.** Habit: Shrub. Habitat: *Acacia*-*Commiphora* bushland, in thickets, temporary pools, and swampy areas, 120–300 (–570) m. Vouchers: Bally 16865, Lucas 60 (EA).

***Phyllanthus suffrutescens* Pax** Habit: Herb. Habitat: Grassland and open bushland often in rocky places, ca. 100 m. Voucher: Gillett 16866 (EA).

***Phyllanthus welwitschianus* Müll. Arg.** Habit: Shrub. Habitat: Coastal bushland, dry forest undergrowth, *Brachystegia* and mixed woodland, ca. 0–224 m. Vouchers: Ngumbau V & Mwadime N V096 (EA, HIB), Maggridge 144, Rawlins 696, Tweedie 1859, Bock K 715 (EA).

***Thecacoris spathulifolia* (Pax) Leandri** Habit: Shrub. Habitat: Dry evergreen forest, wooded grassland, and thickets, 15–700 m. Vouchers: Dale in FD 3560, Polhill & Paulo 772 (EA).

***Wielandia fadenii* (Radcl.-Sm.) Petra Hoffm. & McPherson** Habit: Shrub. Habitat: Lowland evergreen forest, ca. 220 m. Vouchers: Dale in FD 3665, Bally & Smith 14396, Hawthorne 200, Thomas Mwandime in Mrs. SA Robertson 7793, Festo L, Luke Q & P 2761, Faden, R.B, Githui, M. & Evans, A.N. 71/263 (EA).

**F135. Phytolaccaceae**

1 Genus, 1 Species

***Rivina humilis* L.** Habit: Herb. Habitat: A weed of closed forests or forest margins, ca. 3 m. Voucher: Mrs Robertson SA 7692 (EA).

**F136. Picrodendraceae**

2 Genera, 2 Species

***Aristogeitonia monophylla* Airy Shaw** Habit: Tree. Habitat: Forest, ca. 199 m. Vouchers: Ngumbau V & Mwadime N V0406 (EA, HIB), Faden RB, Evans A & Msafiri F 70/949 (EA): Vulnerable.

***Oldfieldia somalensis* (Chiov.) Milne-Redh.** Habit: Tree. Habitat: Lowland dry evergreen and semi deciduous forest, 30–500 m. Vouchers: Perdue & Kibuwa 10005, Greenway & Rawlins 9352, Ross KS 193, Kirika P, Nyamongo D & Sanyanyi S 04/16/2008 (EA).

**F137. Piperaceae**

2 Genera, 2 Species

***Peperomia pellucida* (L.) Kunth** Habit: Herb. Habitat: Seasonally swampy areas in grassland, scattered tree grassland, and evergreen thicket, ca. 0–30 m. Vouchers: Schlieben 12142, RB & AJ Faden 74/1213 (EA).

***Piper betle* L.** Habit: Climber. Habitat: Humid forest, ca. 240 m. Vouchers: Robertson SA 7421, Luke WRQ 1647 (EA): Cultivated.

**F138. Plantaginaceae**

4 Genera, 5 Species

***Bacopa crenata* (Benth.) Hepper** Habit: Herb. Habitat: Grassy swamps and salt marshes, ca. 0–36 m. Vouchers: SAJIT–006198, Ngumbau V & Mwadime N V0552 (EA, HIB), Drummond & Hemsley 4009 & 1186, Magogo & Glover 130, Luke WRQ & Robertson SA 2754 (EA).

***Bacopa floribunda* (R.Br.) Wettst.** Habit: Herb. Habitat: Wet places and rice fields, ca. 2–350 m. Vouchers: Drummond & Hemsley 4010 & 4144, Polhill & Paulo 888, Festo L & Luke Q 2579 (EA).

***Limnophila indica* (L.) Druce** Habit: Herb. Habitat: Marshes, pools, along riversides or forest paths, 0–200 m. Voucher: Festo L & Luke Q 2566 (EA).

***Scoparia dulcis* L.** Habit: Herb. Habitat: In cultivated and waste ground, 0–50 m. Vouchers: Magogo & Glover 1034, Mwachala & Vollesen 57, Kirika P, Mbale M & Mbatha M 779 (EA).

***Stemodia serrata* Benth.** Habit: Herb. Habitat: Moist, grassy places, flood plains, drying out during dry season, ca. 50 m. Voucher: Luke et al. 562 (EA).

**F139. Plumbaginaceae**

1 Genus, 1 Species

***Plumbago stenophylla* Wilmot-Dear** Habit: Herb. Habitat: Coastal forest, ca. 0–30 m. Vouchers: Graham RM 615 & 2103 (EA): Endemic.

**F140. Poaceae**

72 Genera, 207 Species

***Acroceras attenuatum* Renvoize** Habit: Herb. Habitat: In shade, ca. 0–300 m. Vouchers: Bogdan A 4714, Magogo & Glover 772, Rawlins 300, Bogdan 4714 (EA).

***Alloteropsis papillosa* Clayton** Habit: Herb. Habitat: Seasonally wet grassland, particularly on black clay, ca. 0–400 m. Vouchers: Robertson SA 3396, Moomaw JC 1165 (EA).

***Andropogon africanus* Franch.** Habit: Herb. Habitat: Seasonally flooded grassland. Voucher: Allan 189 (EA).

***Andropogon canaliculatus* Schumach.** Habit: Herb. Habitat: Mainly in moist or swampy places, ca. 0–300 m. Voucher: Magogo FC & Glover PE 612 (EA).

***Andropogon chinensis* (Nees) Merr.** Habit: Herb. Habitat: Deciduous bushland and wooded grassland, 0–200 m. Vouchers: Thomas Mwambiri 10, Bogdan A 3316 (EA).

***Andropogon heterantherus* Stapf** Habit: Herb. Habitat: Coastal scrub, 0–100 m. Vouchers: Wilson J 32, Bogdan 3310 (EA).

***Andropogon schirensis* Hochst. ex A. Rich.** Habit: Herb. Habitat: Deciduous bushland and wooded grassland, ca. 100 m. Voucher: Moomaw JC 1217 (EA).

***Aristida adscensionis* L.** Habit: Herb. Habitat: Waste places, ca. 0–150 m. Vouchers: Magogo FC & Glover PE 704, Kirika P 85 (EA).

***Aristida barbicollis* Trin. & Rupr.** Habit: Herb. Habitat: Deciduous bushland and open places, ca. 0–351 m. Vouchers: JM 15, Magogo FC & Glover PE 329 (EA).

***Aristida kelleri* Hack.** Habit: Herb. Habitat: Deciduous bushland, 250–450 m. Voucher: Verdcourt 2101 (EA).

***Aristida mutabilis* Trin. & Rupr.** Habit: Herb. Habitat: *Acacia*-*Commiphora* deciduous bushland and open semi-desert bushland, ca. 100–304 m. Voucher: Magogo FC & Glover PE 521 (EA).

***Aristida sieberiana* Trin. ex Spreng.** Habit: Herb. Habitat: Deciduous bushland, ca. 300 m. Voucher: Pratt in EAH 13812 (EA).

***Aristida stenostachya* Clayton** Habit: Herb. Habitat: Deciduous thicket and bushland, 0–426 m. Vouchers: Magogo FC & Glover PE 692, Muchiri J 475, Bogdan 5322 (EA).

***Axonopus flexuosus* (Peter) Troupin** Habit: Herb. Habitat: Damp or swampy soils, 0–152 m. Vouchers: Luke PA & WRQ 3611, Bogdan 5348 & 3343 (EA).

***Bothriochloa bladhii* (Retz.) S. T. Blake** Habit: Herb. Habitat: Streamside, swamp margins, and cracking clays, ca. 0–75 m. Voucher: Magogo FC & Glover PE 964 (EA).

***Bothriochloa insculpta* (Hochst. ex A. Rich.) A. Camus** Habit: Herb. Habitat: Overgrazed and disturbed places, ca. 50 m. Voucher: Tateoka 3023 (EA).

***Brachiaria brizantha* (A. Rich.) Stapf** Habit: Herb. Habitat: Deciduous woodland, wooded grassland, and upland grassland, ca. 300 m. Vouchers: Taylor 1212, Magogo FC & Glover PE 206 (EA).

***Brachiaria chusqueoides* (Hack.) Clayton** Habit: Herb. Habitat: Coastal bushland and forest in shade, rarely inland, 0–274 m. Vouchers: Luke WRQ & Robertson SA 2193, Magogo & Glover 770, Gillespie 303 (EA).

***Brachiaria deflexa* (Schumach.) C. E. Hubb. ex Robyns** Habit: Herb. Habitat: Deciduous bushland, margins or riverine forest, 0–300 m. Vouchers: Luke PA & WRQ 6138, Magogo FC & Glover PE 814 (EA).

***Brachiaria dictyoneura* (Fig. & De Not.) Stapf** Habit: Herb. Habitat: Wooded grassland and deciduous bushland, ca. 300 m. Vouchers: Moomaw JC 970, Magogo FC & Glover PE 901 (EA).

***Brachiaria distachya* (L.) Stapf** Habit: Herb. Habitat: Weedy places, 0–300 m. Vouchers: Allan 117, Bogdan 3333, Graham MD 13, Luke WRQ & PA 6261 (EA).

***Brachiaria leersioides* (Hochst.) Stapf** Habit: Herb. Habitat: Waysides, old farmland, and weedy places, ca. 0–200 m. Vouchers: Patternson GD 29, Polhill & Paulo 745 (EA).

***Brachiaria leucacrantha* (K. Schum.) Stapf** Habit: Herb. Habitat: Deciduous coastal bushland and disturbed places, ca. 0–250 m. Vouchers: Greenway PJ & Rawlins SP 11337, Magogo FC & Glover PE 714, Drummond & Hemsley 3812 (EA).

***Brachiaria lindiensis* (Pilg.) Clayton** Habit: Herb. Habitat: In shade of open forest, 0–250 m. Voucher: Faden RB, Evans A & Rathbun G 71/690 (EA).

***Brachiaria longiflora* Clayton** Habit: Herb. Habitat: Coastal bushland, 0–30 m. Vouchers: Hacker JB 81, Faden 71/807, Gillespie 124 (EA).

***Brachiaria reptans* (L.) C.A. Gardner & C.E. Hubb.** Habit: Herb. Habitat: Roadsides and weedy places, 0–400 m. Vouchers: Leauthaud C 123, Church 85, Bogdan 5329, Faden 74/116 (EA).

***Brachiaria rugulosa* Stapf** Habit: Herb. Habitat: Streamside, ca. 15 m. Voucher: Bogdan 4698 (EA).

***Brachiaria serrifolia* (Hochst.) Stapf** Habit: Herb. Habitat: Deciduous bushland, ca. 400 m. Voucher: Luke WRQ & Robertson SA 2176 (EA).

***Cenchrus biflorus* Roxb.** Habit: Herb. Habitat: A noxious weed of old farm land and waste places, 0–650 m. Voucher: GR Williams 818 (EA).

***Cenchrus ciliaris* L.** Habit: Herb. Habitat: Deciduous bushland and wooded grassland, ca. 0–350 m. Voucher: Luke PA & WRQ 6133 (EA).

***Cenchrus echinatus* L.** Habit: Herb. Habitat: Roadside and waste places, 0–350 m. Vouchers: Spjut RW 2630, Luke WRQ & PA 6266, Magogo & Glover 1060 & 816, Bally 4704 (EA).

***Cenchrus mitis* Andersson** Habit: Herb. Habitat: Coastal bushland on sandy soils, 0–100 m. Vouchers: Kimeu JM 663, Magogo FC & Glover PE 816, Drummond & Hemsley 3816, Greenaway 10832, Polhill & Paulo 574 (EA).

**Cenchrus pedicellatus subsp. unispiculus (Brunken) Morrone** Habit: Herb. Habitat: Coastal bushland and disturbed sites, ca. 100 m. Voucher: Jeffrey 538 (EA).

**Cenchrus polystachios (L.) Morrone. subsp. polystachios** Habit: Herb. Habitat: Old farmland and disturbed places, ca. 0–303 m. Vouchers: Ngumbau V & Mwadime N V036 (EA, HIB), Pettersson GD 6, Magogo FC & Glover PE 824, Polhill & Paulo 744 (EA).

**Cenchrus polystachios subsp. atrichus (Stapf & C.E. Hubb.) Morrone** Habit: Herb. Habitat: Seasonally flooded grassland on sand or clay, 0–420 m. Vouchers: DKS Grant 878, Rawlins 401 (EA).

***Cenchrus purpureus* (Schumach.) Morrone** Habit: Herb. Habitat: Riverine sites, valley bottoms, and forest margins, ca. 0–300 m. Voucher: Allan 506 (EA).

***Chloris barbata* Sw.** Habit: Herb. Habitat: Waste places or disturbed ground usually near the coast, 0–400 m. Vouchers: Davis RM 1256, Luke WRQ & PA 6263, Napier 6379, Leah & Bayliss 10294 (EA).

***Chloris gayana* Kunth** Habit: Herb. Habitat: Open grassland, ca. 120 m. Voucher: Magogo FC & Glover PE sr.

***Chloris mossambicensis* K. Schum.** Habit: Herb. Habitat: Grassland with scattered trees or bushland, 200–381 m. Vouchers: Patterson GD 24, Magogo FC & Glover PE 823, Drummond & Hemsley 4189, Bogdan 5429 (EA).

***Chloris pycnothrix* Trin.** Habit: Herb. Habitat: Grassland, deciduous woodland, and disturbed ground, ca. 200 m. Voucher: Magogo FC & Glover PE 240 (EA).

***Chloris virgata* Sw.** Habit: Herb. Habitat: Scattered tree grassland, bushland, and disturbed habitats, ca. 10 m. Voucher: Rawlins 682 (EA).

***Chloris woodii* Renvoize** Habit: Herb. Habitat: Deciduous bushland, ca. 270 m. Voucher: Wood D 1363 (EA).

***Chrysopogon plumulosus* Hochst.** Habit: Herb. Habitat: Deciduous bushland and sub-desert grassland, 0–350 m. Voucher: Graham A4 (EA).

***Cleistachne sorghoides* Benth.** Habit: Herb. Habitat: On fallow land following cultivation, ca. 0–50 m. Vouchers: Allan 96, Moggridge 455, Bogdan 2549 (EA).

***Coelorachis lepidura* Stapf** Habit: Herb. Habitat: Swampy grassland, 0–300 m. Voucher: Allan 188 (EA).

***Coix lacryma-jobi* L.** Habit: Herb. Habitat: Wet places in grassland, ca. 90 m. Voucher: Luke WRQ 3305 (EA).

***Ctenium concinnum* Nees** Habit: Herb. Habitat: Open grassy places in deciduous bushland, 250 m. Vouchers: Moomaw JC 1156, Bogdan A 3905 (EA).

***Cymbopogon caesius* (Hook. & Arn.) Stapf** Habit: Herb. Habitat: Wooded grassland, deciduous bushland, and open grassland, 0–322 m. Vouchers: Magogo FC & Glover PE 557, Drummond & Hemsley 1073 (EA).

***Cymbopogon commutatus* (Steud.) Stapf** Habit: Herb. Habitat: Deciduous bushland and sub-desert grassland, ca. 100 m. Voucher: Praet van (EA).

***Cynodon dactylon* (L.) Pers.** Habit: Herb. Habitat: Roadsides, old farmland, and weedy, 0–410 m. Voucher: Bogdan 2531 (EA).

**Cynodon nlemfuensis Vanderyst var. nlemfuensis** Habit: Herb. Habitat: Open places in deciduous bushland or forest, ca. 300 m. Voucher: Luke WRQ & PA 6258 (EA).

**Cynodon nlemfuensis var. robustus Clayton & Harlan** Habit: Herb. Habitat: Clearings in forest, deciduous bushland, cattle paddocks, and old cultivation, ca. 300 m. Voucher: Verdcourt 3944 (EA).

***Cyrtococcum trigonum* (Retz.) A. Camus** Habit: Herb. Habitat: In forest shade, 0–400 m. Vouchers: Luke WRQ & PA 8196, Drummond & Hemsley 1193, Bogdan 4713, Greenway & Rawlins 9368 (EA).

***Dactyloctenium aegyptium* (L.) Willd.** Habit: Herb. Habitat: Grassland, open woodland, roadsides, and waste ground, ca. 10 m. Voucher: Magogo FC & Glover PE 1081 (EA).

***Dactyloctenium aristatum* Link** Habit: Herb. Habitat: A seashore grass on sand and exposed coral outcrops, sea level. Vouchers: Leauthaud C 39, Greenway 9263, Rawlins 121, Gillespie 238 (EA).

***Dactyloctenium australe* Steud.** Habit: Herb. Habitat: Bushland and grassland, ca. 0–800 m. Voucher: Bogdan A 5430 (EA).

***Dactyloctenium ctenoides* (Steud.) Bosser** Habit: Herb. Habitat: Sand dunes, coral outcrops on or near the seashore, sea level. Vouchers: Allan 30, Echlin 7, Bogdan 2532 (EA).

***Dactyloctenium geminatum* Hack.** Habit: Herb. Habitat: Coastal bushland, 0–170 m. Vouchers: Luke Q 6139, Bogdan 2597, Kassner 451, Gillespie 160 (EA).

***Dactyloctenium giganteum* B.S. Fisher & Schweick.** Habit: Herb. Habitat: Roadsides, old cultivations, and other disturbed sites, ca. 200 m. Voucher: Sampson 21 (EA).

***Dactyloctenium pilosum* Stapf** Habit: Herb. Habitat: On rocks by the sea, sea level. Vouchers: Bogdan 4704, Harker 41a (EA).

***Dactyloctenium scindicum* Boiss.** Habit: Herb. Habitat: Dry grassland and open bushland, ca. 20 m. Voucher: Luke WRQ & PA 6119 (EA).

***Daknopholis boivinii* (A. Camus) Clayton** Habit: Herb. Habitat: Coastal bushland, near the seashore. Voucher: Hacker 127 (EA).

***Dichanthium annulatum* (Forssk.) Stapf** Habit: Herb. Habitat: Dry open places subject to grazing or disturbances, 0–800 m. Voucher: Hitchcock 25163 (EA).

***Dichanthium foveolatum* (Delile) Roberty** Habit: Herb. Habitat: Deciduous bushland or sub-desert grassland, 0–800 m. Voucher: Heady HF 1358 (EA).

***Digitaria abyssinica* (Hochst. ex A. Rich.) Stapf** Habit: Herb. Habitat: Cultivated areas, ca. 20 m. Vouchers: Polhill & Paulo 532, Luke WRQ & PA 5937 (EA).

***Digitaria argyrotricha* (Andersson) Chiov.** Habit: Herb. Habitat: Seashore and coastal bushland, 0–365 m. Vouchers: Kuchar P 13588, Luke WRQ & PA 6262, Drummond & Hemsley 3821, Greenway 10819, Bogdan 5698 (EA).

***Digitaria aridicola* Napper** Habit: Herb. Habitat: Dry open deciduous bushland, 200–600 m. Voucher: Luke WRQ & Robertson SA 2187 (EA).

***Digitaria ciliaris* (Retz.) Koeler** Habit: Herb. Habitat: Roadsides and weedy places, 0–200 m. Voucher: Hacker JB 142 (EA).

***Digitaria diagonalis* (Nees) Stapf** Habit: Herb. Habitat: Open grassy places in a wide range of habitats from waterlogged, 0–200 m. Voucher: Magogo FC & Glover PE 233 (EA).

***Digitaria gymnotheca* Clayton** Habit: Herb. Habitat: In shade of trees, bushes or rocks on sandy soils, ca. 0–30 m. Voucher: Bogdan A 2589 (EA).

***Digitaria longiflora* (Retz.) Pers.** Habit: Herb. Habitat: Open places in deciduous bushland, disturbed soils, old farmlands, and path sides, ca. 200 m. Voucher: Drummond & Hemsley 4198 (EA).

***Digitaria milanjiana* (Rendle) Stapf** Habit: Herb. Habitat: Recorded from a wide range of habitats, ca. 10 m. Vouchers: Luke Q 6135, Magogo FC & Glover PE 296, Drummond & Hemsley 3825, Makin 298 (EA).

***Digitaria nuda* Schumach.** Habit: Herb. Habitat: Open weedy places, ca. 300 m. Vouchers: Church 87, Drummond & Hemsley 1079, Allan 182 (EA).

***Digitaria pennata* (Hochst.) T. Cooke** Habit: Herb. Habitat: Dry open deciduous bushland, ca. 100 m. Voucher: Praet van U.N.D.P/F.A.O (EA).

***Digitaria perrottetii* (Kunth) Stapf** Habit: Herb. Habitat: Deciduous bushland, particularly on old farmland, and disturbed places, 0–100 m. Voucher: Bogdan 4718 (EA).

***Digitaria velutina* (Forssk.) P. Beauv.** Habit: Herb. Habitat: A weed of path sides, farmland, and open places, 0–228 m. Voucher: Ngumbau V & Mwadime N V054 (EA, HIB).

***Dignathia gracilis* Stapf** Habit: Herb. Habitat: Coastal bushland, 0–15 m. Vouchers: Luke Q 6155, Greenway 10841, Polhill & Paulo 650, Greenway 9497 (EA).

***Dignathia hirtella* Stapf** Habit: Herb. Habitat: Deciduous bushland, 300–800 m. Voucher: Ndakala J Gang P 279 (EA).

***Diheteropogon amplectens* (Nees) Clayton** Habit: Herb. Habitat: Deciduous bushland, coastal bushland, and wooded grassland, ca. 0–320 m. Voucher: Magogo FC & Glover PE 57 (EA).

**Dinebra retroflexa var. condensata S.M. Phillips** Habit: Herb. Habitat: Grassland and open woodland, ca. 300 m. Voucher: Moomaw 1380 (EA).

***Echinochloa colona* (L.) Link** Habit: Herb. Habitat: A weedy species of muddy or swampy places, ca. 0–85 m. Vouchers: Luke WRQ & PA 6269, Nyange M 507 (EA).

***Echinochloa haploclada* (Stapf) Stapf** Habit: Herb. Habitat: Stream banks and dry river beds, ca. 5–85 m. Vouchers: Leauthaud C 5, Luke WRQ & PA 6259 (EA).

***Echinochloa stagnina* (Retz.) P. Beauv.** Habit: Herb. Habitat: Swamps and standing water, ca. 5 m. Voucher: Leauthaud C 8 (EA).

***Eleusine indica* (L.) Gaertn.** Habit: Herb. Habitat: Roadsides, waste ground, and cultivated land, 0–213 m. Vouchers: Luke WRQ & PA 6256, Leauthaud C 18 (EA).

***Eleusine semisterilis* S.M. Phillips** Habit: Herb. Habitat: Dry open ground, ca. 300 m. Voucher: Allan 507 (EA): Endemic.

***Enteropogon barbatus* C.E. Hubb.** Habit: Herb. Habitat: Not precisely recorded, ca. 150 m. Vouchers: VG van Someren in Herb, Amani 9863 & Bogdan 4506 (EA).

***Enteropogon macrostachyus* (Hochst. ex A. Rich.) Munro ex Benth.** Habit: Herb. Habitat: Disturbed places, particularly in *Acacia*-*Commiphora* bushland, ca. 150–400 m. Vouchers: Luke Q & Kabuye CHS 5121, Magogo FC & Glover PE 888 (EA).

***Enteropogon rupestris* (J.A. Schmidt) A. Chev.** Habit: Herb. Habitat: Scattered tree grassland, ca. 300 m. Voucher: Moomaw JC 1378 (EA).

***Enteropogon sechellensis* (Benth.) T. Durand & Schinz** Habit: Herb. Habitat: Coastal bushland, sea level. Vouchers: Drummond & Hemsley 3915, Greenway 10835, 9441 (EA).

***Eragrostiella bifaria* (Vahl) Bor** Habit: Herb. Habitat: Semi-desert grassland or dry bushland, and often among rocks, ca. 300 m. Voucher: Sheldrick D 103 (EA).

***Eragrostis aethiopica* Chiov.** Habit: Herb. Habitat: Disturbed ground and weedy places, ca. 100 m. Voucher: Mwadime N & Luke WRQ 2635 (EA).

***Eragrostis unioloides* (Retz.) Nees ex Steud.** Habit: Herb. Habitat: Path sides and cultivated land, ca. 300 m. Voucher: Bally 8936 (EA).

***Eragrostis ambleia* Clayton** Habit: Herb. Habitat: Deciduous bushland, 200–500 m. Voucher: Hacker 161b (EA).

***Eragrostis cilianensis* (All.) Vignolo ex Janch.** Habit: Herb. Habitat: Path sides, farmland, and overgrazed places, ca. 100 m. Voucher: Polhill & Paulo 906 (EA).

***Eragrostis ciliaris* (L.) R.Br.** Habit: Herb. Habitat: Farmland, clearings, and overgrazed, ca. 0–70 m. Vouchers: Leauthaud C 37, Magogo FC & Glover PE 820, Bogdan 2618 (EA).

***Eragrostis cylindriflora* Hochst.** Habit: Herb. Habitat: Overgrazed places and dry sandy riverbeds, ca. 90 m. Voucher: Greenway & Kanuri 12872 (EA).

***Eragrostis exasperata* Peter** Habit: Herb. Habitat: Moist grassland, streamside, and seasonally flooded places, ca. 300 m. Voucher: Heady 1375 (EA).

***Eragrostis homomalla* Nees** Habit: Herb. Habitat: Moist soils bordering depressions, ca. 200 m. Voucher: Estes 29 (EA).

***Eragrostis inamoena* K. Schum.** Habit: Herb. Habitat: Floodplain grassland and swampy grassland, ca. 300 m. Voucher: Moomaw JC 1158 (EA).

***Eragrostis lappula* Nees** Habit: Herb. Habitat: *Brachystegia* woodland, ca. 350 m. Voucher: JA Allan 108A (EA).

***Eragrostis perbella* K. Schum.** Habit: Herb. Habitat: Coastal bushland, 0–450 m. Vouchers: Bogdan A 3320, Moomaw 1159, Magogo & Glover 847, Linton 194 (EA).

***Eragrostis pilosa* (L.) P. Beauv.** Habit: Herb. Habitat: Roadsides, old farmland, and weedy places, ca. 300 m. Voucher: Hitchcock 25153 (EA).

***Eragrostis racemosa* (Thunb.) Steud.** Habit: Herb. Habitat: Moist vegetation types, ca. 300 m. Voucher: Magogo FC & Glover PE 229 (EA).

***Eragrostis sennii* Chiov.** Habit: Herb. Habitat: Roadside, sea level. Voucher: Pearsson in EAH 15302 (EA).

***Eragrostis superba* Peyr.** Habit: Herb. Habitat: Deciduous bushland or wooded grassland, often in disturbed places, 0–410 m. Voucher: Luke WRQ & PA 6270 (EA).

***Eragrostis* sp. A of FTEA** Habit: Herb. Habitat: Bushland. Voucher: Greenway 9259 (EA): Endemic.

***Eragrostis tenuifolia* (A. Rich.) Steud.** Habit: Herb. Habitat: Forest, ca. 50 m. Voucher: A Bogdan 4717 (EA).

***Eriochloa fatmensis* (Hochst. & Steud.) Clayton** Habit: Herb. Habitat: Swampy places and damp depressions, 0–300 m. Vouchers: RB & AJ Faden 74/1163, Magogo FC & Glover PE 696 (EA).

***Eriochloa meyeriana* (Nees) Pilg.** Habit: Herb. Habitat: Swampy places and streamside, ca. 0–300 m. Voucher: Luke WRQ & PA 6265 (EA).

***Eriochloa parvispiculata* C.E. Hubb.** Habit: Herb. Habitat: Damp places in coastal bushland and near cultivation, 0–300 m. Vouchers: Leauthaud C 34, Luke WRQ & PA 6264, Allan 37, Bogdan 3308, Polhill & Paulo 502 (EA).

***Eriochloa stapfiana* Clayton** Habit: Herb. Habitat: Swampy places and streamside, 0–800 m. Voucher: Polhill & Paulo 831 (EA).

***Eustachys paspaloides* (Vahl) Lanza & Mattei** Habit: Herb. Habitat: Deciduous bushland and dry grassland, 300 m. Voucher: Magogo FC & Glover PE 372 (EA).

***Hackelochloa granularis* (L.) Kuntze** Habit: Herb. Habitat: Disturbed soils around habitations and old farmland, 0–123 m. Vouchers: Luke WRQ 3301, Drummond & Hemsley 1090, Bogdan 3906 (EA).

***Halopyrum mucronatum* (L.) Stapf** Habit: Herb. Habitat: Coastal sand dunes, sea level. Vouchers: Luke Q 6113, Polhill & Paulo 766, Greenway & Rawlins 8913 (EA).

***Heteropogon contortus* (L.) P. Beauv. ex Roem. & Schult.** Habit: Herb. Habitat: Deciduous bushland and wooded grassland, 0–20 m. Vouchers: Magogo FC & Glover PE 399, Drummond & Hemsley 4196 (EA).

***Heteropogon melanocarpus* (Elliott) Benth.** Habit: Herb. Habitat: Abandoned cultivation, bushland and *Brachystegia* wooded grassland, ca. 80 m. Vouchers: Drummond & Hemsley 3949, Bogdan 2552 & 2614 (EA).

***Holcolemma inaequale* Clayton** Habit: Herb. Habitat: Coastal bushland, 10–200 m. Vouchers: Polhill & Paulo 564 & 619 (EA).

***Hylebates chlorochloe* (K. Schum.) Napper** Habit: Herb. Habitat: Shady places in forest or bushland, 50–300 m. Vouchers: Ngumbau V & Mwadime N V023 (EA, HIB), Magogo FC & Glover PE 944 & 641, Bogdan 3909 (EA).

***Hyparrhenia filipendula* (Hochst.) Stapf** Habit: Herb. Habitat: Wide range of soil and vegetation types, particularly those subject to disturbances, ca. 200 m. Vouchers: Hacker JB 140, Magogo FC & Ester R 1212A (EA).

***Hyparrhenia poecilotricha* (Hack.) Stapf** Habit: Herb. Habitat: Deciduous bushland, 0–304 m. Vouchers: Drummond RB & Hemsley JH 1081, Ross K 102 (EA).

***Hyparrhenia rufa* (Nees) Stapf** Habit: Herb. Habitat: Deciduous bushland and wooded grassland, ca. 100 m. Vouchers: Magogo FC & Glover PE 658, Verdcourt 1868 (EA).

***Hyperthelia dissoluta* (Nees ex Steud.) Clayton** Habit: Herb. Habitat: Deciduous bushland and wooded grassland, ca. 0–326 m. Vouchers: Magogo FC & Glover PE 63, Mwadime N & Gaya H 71 (EA).

***Imperata cylindrica* (L.) Raeusch.** Habit: Herb. Habitat: Cultivated areas, ca. 0–381 m. Vouchers: Kimeu JM Meso M & Ot 606, Magogo FC & Glover PE 381, Drummond & Hemsley 1031 (EA).

***Ischaemum rugosum* Salisb.** Habit: Herb. Habitat: Damp soils, pond margins, and rice fields, 0–50 m. Voucher: Greenway & Rawlins 9482 (EA).

***Leersia hexandra* Sw.** Habit: Herb. Habitat: In shallow water, ca. 40 m. Vouchers: Leauthaud C 7, Nyange M 339 (EA).

***Leptaspis zeylanica* Nees ex Steud.** Habit: Herb. Habitat: Ground layer in forest, ca. 400 m. Voucher: Drummond RB & Hemsley JH 1127 (EA).

***Leptochloa chinensis* (L.) Nees** Habit: Herb. Habitat: Aquatic or semi-aquatic, in damp hollows and in shallow water at pond margins, 0–400 m. Voucher: Sampson 63 (EA).

***Leptochloa fusca* (L.) Kunth** Habit: Herb. Habitat: Shallow margins of lakes and rivers, 0–100 m. Voucher: Bogdan 2605 (EA).

***Leptochloa obtusiflora* Hochst.** Habit: Herb. Habitat: Deciduous bushland and grassland, ca. 0–300 m. Vouchers: Magogo FC & Glover PE 871, Moomaw 1160 (EA).

***Leptochloa panicea* (Retz.) Ohwi** Habit: Herb. Habitat: Deciduous bushland, wooded grassland, plantations, roadsides, and waste places, 0–300 m. Vouchers: Magogo FC & Glover PE 1051, Bogdan 5431 & 3305, Rawlins 446 (EA).

***Leptochloa uniflora* Hochst. ex A. Rich.** Habit: Herb. Habitat: Wooded grassland and deciduous bushland, 0–100 m. Vouchers: Magogo FC & Glover PE 368, Verdcourt 1913, Bogdan 3910, Gillespie 195 (EA).

***Leptothrium senegalense* (Kunth) Clayton** Habit: Herb. Habitat: Deciduous bushland and sub-desert grassland, ca. 5 m. Voucher: Luke Q 6117(EA).

***Lepturus radicans* (Steud.) A. Camus** Habit: Herb. Habitat: Sandy in grassland, 0–300 m. Vouchers: Luke Q 6118, JA Allan 94, Napper 1665, Bogdan 4711 (EA).

***Lepturus repens* (J.R. Forst.) R.Br.** Habit: Herb. Habitat: Coastal sands above high-water mark. Vouchers: JA Allan 97, Bogdan 3642, Longridge 24 (EA).

***Megastachya mucronata* (Poir.) P. Beauv.** Habit: Herb. Habitat: Forest shade, ca. 0–400 m. Vouchers: SAJIT–005516, Ngumbau V & Mwadime N V0103 (EA, HIB), Kirika P, Nyamongo D & Sanyanyi S 04/13/2008, Robertson SA 3394, Drummond & Hemsley 3835, Bogdan 3912, Greenway & Rawlins 9347 (EA).

**Melinis repens (Willd.) Zizka subsp. repens** Habit: Herb. Habitat: Disturbed areas, ca. 43 m. Vouchers: Magogo FC & Glover PE 717, Luke WRQ 16133 (EA).

***Olyra latifolia* L.** Habit: Herb. Habitat: Edges of clearings and path sides in forests, ca. 300 m. Vouchers: Ngumbau V & Mwadime N V0164 (EA, HIB), Verdcourt B 3930, Lucas, Jeffery & Kirika 229, JA Allan 501 (EA).

***Oplismenus burmannii* (Retz.) P. Beauv.** Habit: Herb. Habitat: In shade of forest or bushland, 0–300 m. Vouchers: Brathys Expenditure 67, Drummond & Hemsley 3998, Hacker 5, Bogdan 4716 (EA).

***Oplismenus compositus* (L.) P. Beauv.** Habit: Herb. Habitat: Forest shade, ca. 200 m. Voucher: Magogo FC & Glover PE 940 (EA).

***Oplismenus hirtellus* (L.) P. Beauv.** Habit: Herb. Habitat: Forest shade, ca. 0–246 m. Vouchers: Luke WRQ et al. 6181, Magogo & Glover 963 (EA).

***Oropetium minimum* (Hochst.) Pilg.** Habit: Herb. Habitat: Dry grassland and open deciduous bushland, ca. 780 m. Vouchers: Legesse A 119, Pratt DJ 699 & 402 (EA).

***Oryza eichingeri* Peter** Habit: Herb. Habitat: forest, ca. 300 m. Vouchers: Luke WRQ & Robertson SA 2295, Magogo FC & Glover PE 769 (EA).

***Oryza longistaminata* A. Chev. & Roehr.** Habit: Herb. Habitat: Swamp and flood plain grassland, ca. 0–100 m. Vouchers: Leauthaud C 23, JA Allan 111, Sampson 58 (EA).

***Oryza punctata* Kotschy ex Steud.** Habit: Herb. Habitat: Swampy soils by stream banks and pond margins, 0–280 m. Vouchers: SAJIT–006225, Ngumbau V & Mwadime N V0258 (EA, HIB), Leauthaud C 33, Luke WRQ 3602, Taylor 1211, Polhill & Paulo 484, Bogdan 5428 (EA).

***Panicum atrosanguineum* Hochst. ex A. Rich.** Habit: Herb. Habitat: Disturbed places and old farmland in deciduous bushland, ca. 100 m. Voucher: Magogo & Glover 187 (EA).

***Panicum brevifolium* L.** Habit: Herb. Habitat: Forest shade, ca. 15 m. Vouchers: SAJIT–006216 (EA, HIB), Magogo FC & Glover PE 114, Bogdan 3341, Drummond & Hemsley 1128 (EA).

***Panicum deustum* Thunb.** Habit: Herb. Habitat: Forest, deciduous bushland, and grassland, ca. 0–70 m. Vouchers: Magogo FC & Glover PE 281, Allan 165 (EA).

***Panicum genuflexum* Stapf** Habit: Herb. Habitat: Wooded grassland or deciduous bushland, ca. 0–100 m. Vouchers: Drummond & Hemsley 3814, Bogdan 3300, Gillespie 193, Muchiri J 550 (EA).

***Panicum hippothrix* K. Schum. ex Engl.** Habit: Herb. Habitat: Deciduous bushland and margins of cultivation, ca. 0–80 m. Vouchers: Rawlins SP 399, Drummond & Hemsley 3823, Bogdan 3307, Moomaw 994, Heady 1378 (EA).

***Panicum hirtum* Lam.** Habit: Herb. Habitat: Deciduous bushland or wooded grassland, ca. 30 m. Voucher: Gilllespie 295 (EA).

***Panicum infestum* Andersson** Habit: Herb. Habitat: Grassland, bushland or deciduous woodland, ca. 0–266 m. Vouchers: Ngumbau V & Mwadime N V047 (EA, HIB), Luke Q 6121, Magogo FC & Glover PE 828 (EA).

***Panicum laticomum* Nees** Habit: Herb. Habitat: Forest shade, 0–800 m. Vouchers: Ngumbau V & Mwadime N V053 (EA, HIB), Robertson SA & Luke WRQ 5496, Magogo FC & Glover PE 457, Verdcourt 1874 & 1080 (EA).

**Urochloa maxima (Jacq.) R. D. Webster subsp. maxima** Habit: Herb. Habitat: Woodland, deciduous bushland, roadsides, and river banks, ca. 0–381 m. Vouchers: Ngumbau V & Mwadime N V102 (EA, HIB), Luke PA & WRQ 6114, Magogo FC & Glover PE 395 (EA).

***Panicum parvifolium* Lam.** Habit: Herb. Habitat: Swamps, ca. 0–100 m. Vouchers: Luke WRQ et al. 4332, Bogdan 5346 (EA).

***Panicum peteri* Pilg.** Habit: Herb. Habitat: Forests and *Brachystegia* woodland, 100–800 m. Voucher: Ngumbau V & Mwadime N V007 (EA, HIB).

***Panicum pinifolium* Chiov.** Habit: Herb. Habitat: Coastal bushland and sand dunes, sea level. Vouchers: Greenway & Rawlins 9286, Rawlins & Oseni (EA).

***Panicum pleianthum* Peter** Habit: Herb. Habitat: Forest shade, 50–550 m. Vouchers: Ngumbau V & Mwadime N V011 (EA, HIB), Robertson SA 5124, Robertson SA 3395A, Magogo & Glover 236, Bogdan 3913, Verdcourt 3916 (EA).

***Panicum stoloniferum* Poir.** Habit: Herb. Habitat: Bush or forest shade or sandy soils, ca. 0–300 m. Vouchers: Magogo FC & Glover PE 332, Drummond & Hemsley 3826 (EA).

***Panicum trichoides* Sw.** Habit: Herb. Habitat: Forest shade, 0–900 m. Vouchers: Moggridge 448, Graham 45 (EA).

***Paspalum glumaceum* Clayton** Habit: Herb. Habitat: Damp places and forest margins, ca. 0–100 m. Vouchers: Magogo FC & Glover PE 664, Allan 110 & 5 (EA).

***Paspalum scrobiculatum* L.** Habit: Herb. Habitat: Damp places and forest margins, ca. 50 m. Vouchers: Magogo FC & Glover PE 324 & 106 (EA).

***Paspalum vaginatum* Sw.** Habit: Herb. Habitat: Coastal salt marshes, but sometimes also in inland marshes, 0–100 m. Vouchers: Allan 114, Bogdan 3314 (EA).

***Perotis hildebrandtii* Mez** Habit: Herb. Habitat: Open places, roadsides, and wasteland, ca. 0–400 m. Vouchers: Magogo FC & Glover PE 369, Greenway 10834 (EA).

***Perotis patens* Gand.** Habit: Herb. Habitat: Path sides and weedy places, ca. 300 m. Vouchers: Drummond & Hemsley 1099, Magogo FC & Glover PE 109 (EA).

**Phragmites australis (Cav.) Trin. ex Steud. subsp. australis** Habit: Herb. Habitat: Shallow water of streams, rivers lakes and in swampy places, 0–300 m. Vouchers: Moomaw 961, Battiscombe 266, Kimeu JM, Meso M & Otieno V 616 (EA).

***Rhytachne rottboellioides* Desv. ex Ham.** Habit: Herb. Habitat: In swamps and seasonally wet grassland, 0–300 m. Voucher: Moomaw JC 1155 (EA).

***Rottboellia cochinchinensis* (Lour.) Clayton** Habit: Herb. Habitat: Disturbed places, 0–110 m. Voucher: Magogo FC & Glover PE 1135 (EA).

**Saccharum spontaneum subsp. aegyptiacum (Willd.) Hack.** Habit: Herb. Habitat: Damp soils, often fringing rivers and lakes, ca. 100 m. Vouchers: Hacker JB 93, DC Edwards 53 (EA).

***Sacciolepis curvata* (L.) Chase** Habit: Herb. Habitat: Damp shady places subject to disturbances, ca. 0–100 m. Vouchers: Kirika P, Muthoka P & Mbale M 750, Magogo FC & Glover PE 672, Bogdan 3309, Greenway 10836, Polhill & Paulo 620 (EA).

***Schizachyrium brevifolium* (Sw.) Buse** Habit: Herb. Habitat: Damp or shady places, ca. 200 m. Vouchers: Bogdan 5351, 2611 & 4700 (EA).

***Schizachyrium exile* (Hochst.) Pilg.** Habit: Herb. Habitat: Bushland and woodland, ca. 600 m. Voucher: Allan 75 (EA).

***Schizachyrium rupestre* (K. Schum.) Stapf** Habit: Herb. Habitat: Moist places in coastal bushland, ca. 100 m. Voucher: Allan 75 (EA).

***Schizachyrium sanguineum* (Retz.) Alston** Habit: Herb. Habitat: Bushland and woodland, ca. 0–365 m. Vouchers: Festo L & Luke Q 2614, Magogo FC & Glover PE 511 (EA).

***Schoenefeldia transiens* (Pilg.) Chiov.** Habit: Herb. Habitat: Wooded grassland, deciduous bushland, and dry grassland, 5–15 m. Vouchers: Polhill & Paulo 823, RB & AJ Faden74/1167 (EA).

***Setaria incrassata* (Hochst.) Hack.** Habit: Herb. Habitat: Swamps to stony hillsides or margins of upland evergreen forest, ca. 0–457 m. Vouchers: Patterson G 27, Ross K 17 (EA).

***Setaria sulcata* Raddi** Habit: Herb. Habitat: Shady places and around forest, ca. 200 m. Vouchers: Ngumbau V & Mwadime N V034 (EA, HIB), Magogo FC & Glover PE 393 & 787, Allan 500 (EA).

***Setaria obtusifolia* (Delile) Morrone** Habit: Herb. Habitat: Marshy soils or shallow water, ca. 0–25 m. Vouchers: Leauthaud C 24, Allan 166 (EA).

***Setaria pumila* (Poir.) Roem. & Schult.** Habit: Herb. Habitat: Path sides, bare patches, and grazing area, ca. 0–300 m. Voucher: Magogo FC & Glover PE 1085 (EA).

***Setaria punctata* (Burm.f.) Veldkamp** Habit: Herb. Habitat: On wet soils or in water, 0–400 m. Voucher: Magogo FC & Glover PE 115 (EA).

***Setaria sphacelata* (Schumach.) Stapf & C.E. Hubb. ex Moss** Habit: Herb. Habitat: Deciduous bushland or wooded grassland, swamps, and riversides, ca. 20 m. Vouchers: Magogo FC & Glover PE 23, Moomaw JC 1254 (EA).

**Sorghum bicolor subsp. verticilliflorum (Steud.) de Wet ex Wiersema & J. Dahlb.** Habit: Herb. Habitat: Swampy soils, streamside, disturbed places, and old farmland, 0–60 m. Vouchers: Greenway 8962, Moomaw JC 962 (EA).

***Sorghum versicolor* Andersson** Habit: Herb. Habitat: Bushland, deciduous bushland, and wooded grassland, 0–400 m. Vouchers: Petterson GD 28, Kirika P, Mbale M & Mbatha M 767, Drummond & Hemsley 3775, Polhill & Paulo 711 (EA).

***Sporobolus congoensis* Franch.** Habit: Herb. Habitat: Wooded grassland, ca. 304 m. Vouchers: Magogo FC & Glover PE 550, Moomaw 1269 (EA).

***Sporobolus coromandelianus* (Retz.) Kunth** Habit: Herb. Habitat: Rare open places, 0–100 m. Voucher: Hitchcock 25158 (EA).

***Sporobolus ioclados* (Trin.) Nees** Habit: Herb. Habitat: Saline grasslands, seashore sands, and sandy patches in mangrove swamps, ca. 0–100 m. Voucher: Polhill & Paulo 687 (EA).

***Sporobolus microprotus* Stapf** Habit: Herb. Habitat: Deciduous bushland, 0–100 m. Voucher: Bogdan 3331A (EA).

***Sporobolus pyramidalis* P. Beauv.** Habit: Herb. Habitat: Disturbed areas, ca. 0–266 m. Vouchers: Ngumbau V & Mwadime N V051 (EA, HIB), Luke WRQ & PA 6268 (EA).

***Sporobolus spicatus* (Vahl) Kunth** Habit: Herb. Habitat: Grassland and open bushland, ca. 0–100 m. Voucher: Bogdan A 4707 (EA).

***Sporobolus stolzii* Mez** Habit: Herb. Habitat: Roadsides and waste places in deciduous bushland, ca. 0–80 m. Vouchers: JA Allan 81, Drummond & Hemsley 3799, Boyle B 127 (EA).

***Sporobolus subglobosus* A. Chev.** Habit: Herb. Habitat: Coastal bushland, up to 300 m. Voucher: Moomaw JC 1096 (EA).

***Sporobolus tenuissimus* (Mart. ex Schrank) Kuntze** Habit: Herb. Habitat: Roadsides and waste places, ca. 0–200 m. Vouchers: JA Allan 8, Burtt Davy 2695, Bogdan 2542, Moomaw JC 1278 (EA).

***Sporobolus virginicus* (L.) Kunth** Habit: Herb. Habitat: Sandy seashores, 0–300 m. Vouchers: SAJIT–006248 (EA, HIB), Bogdan 2632, Gillespie 110, Greenway & Rawlins 8911, Luke Q 6112 (EA).

***Stenotaphrum dimidiatum* (L.) Brongn.** Habit: Herb. Habitat: Seashore, usually in shade, 0–10 m. Vouchers: Ngumbau V & Mwadime N V0512 (EA, HIB), Drummond & Hemsley 3996, Bogdan 3645, Greenway & Rawlins 9465 (EA).

***Tetrapogon roxburghiana* (Schult.) P. M. Peterson** Habit: Herb. Habitat: Wooded grassland, bushland, and disturbed habitats, ca. 30 m. Vouchers: Hacker JB 111, Luke WRQ & PA 6260, Drummond & Hemsley 3849 (EA).

***Tetrapogon bidentatus* Pilg.** Habit: Herb. Habitat: Deciduous bushland or grassland, ca. 30–65 m. Vouchers: TPR 282, RB & AJ Faden 74/1049 (EA).

***Tetrapogon cenchriformis* (A. Rich.) Clayton** Habit: Herb. Habitat: Deciduous bushland or grassland, ca. 50 m. Voucher: J Makin 14667 (EA).

***Tetrapogon tenellus* (J. Koenig ex Roxb.) Chiov.** Habit: Herb. Habitat: Deciduous bushland or grassland, ca. 0–65 m. Vouchers: Leauthaud C 6, Magogo FC & Glover PE 881, Drummond & Hemsley 4127 (EA).

***Themeda triandra* Forssk.** Habit: Herb. Habitat: Deciduous bushland, ca. 0–381 m. Vouchers: Robertson SA & Luke WRQ 5113, Magogo FC & Glover PE 827 (EA).

***Trachypogon spicatus* (L.f.) Kuntze** Habit: Herb. Habitat: Deciduous bushland or wooded grassland, ca. 0–370 m. Voucher: Magogo FC & Glover PE 330 (EA).

***Tragus berteronianus* Schult.** Habit: Herb. Habitat: Overgrazed places, ca. 0–100 m. Voucher: Magogo FC & Glover PE 932 (EA).

***Tragus heptaneuron* Clayton** Habit: Herb. Habitat: Deciduous bushland, 0–800 m. Vouchers: Bogdan A 5333, Polhill & Paulo 515 (EA).

***Tragus racemosus* (L.) All.** Habit: Herb. Habitat: Deciduous bushland, ca. 200 m. Vouchers: Amani 9868A, VGL van Someren (EA).

***Trichoneura mollis* (Kunth) Ekman** Habit: Herb. Habitat: Dry bushland, 100–230 m. Vouchers: Koss 114, Makin (EA).

***Urochloa mosambicensis* (Hack.) Dandy** Habit: Herb. Habitat: Wooded grassland, deciduous bushland, and disturbed sites, ca. 0–100 m. Voucher: Allan 79 (EA).

***Urochloa panicoides* P. Beauv.** Habit: Herb. Habitat: Old farmland, overgrazed, and deciduous bushland, ca. 0–170 m. Voucher: Leauthaud C 36 (EA).

***Urochloa rudis* Stapf** Habit: Herb. Habitat: Coastal bushland, 0–30 m. Vouchers: Allan 151 & 173, Polhill & Paulo 589 (EA).

***Urochloa sclerochlaena* Chiov.** Habit: Herb. Habitat: Coastal and semi-desert bushland, ca. 200 m. Voucher: Thairu 125 (EA).

***Urochloa setigera* (Retz.) Stapf** Habit: Herb. Habitat: Lowland and riverine forest, 0–900 m. Voucher: Greenway & Rawlins 9365 (EA).

***Urochloa trichopus* (Hochst.) Stapf** Habit: Herb. Habitat: Wooded grassland and coastal bushland, 0–100 m. Vouchers: Drummond & Hemsley 3841, Bogdan 2529, Gillespie 10, Luke Q 6140 (EA).

***Vossia cuspidata* (Roxb.) Griff.** Habit: Herb. Habitat: In or close to water, often floating, 0–200 m. Voucher: Leauthaud C 12 (EA).

**F141. Polygalaceae**

3 Genera, 17 Species

***Carpolobia goetzei* Gürke** Habit: Shrub. Habitat: Moist or dry forest, riverine forest, ca. 0–210 m. Vouchers: SAJIT–006453, Ngumbau V & Mwadime N V0389 (EA, HIB), Drummond & Hemsley 1135, Adams 93, Festo L, Luke Q & P 2759, Magogo FC & Glover PE 269 (EA).

***Polygala amboniensis* Gürke** Habit: Herb. Habitat: Bushland, secondary grassland, and coastal dunes, 0–100 m. Vouchers: SAJIT–006098 (EA, HIB), Polhill & Paulo 594, Luke & Saidi 6250, Luke WRQ 3337 & 2796 (EA).

***Polygala arenaria* Willd.** Habit: Herb. Habitat: Grassland, scattered tree grassland, ruderal sites, ca. 0–200 m. Vouchers: Makin 425, Luke 3572, Luke WRQ 2453A(EA).

***Polygala conosperma* Bojer** Habit: Herb. Habitat: Wooded grassland, 0–10 m. Vouchers: Blake 2278, Luke 3323 (EA).

***Polygala erioptera* DC.** Habit: Herb. Habitat: Grassland, sandy, and muddy roadside, 30 m. Voucher: Klotzli F et al. 601 (EA).

***Polygala fischeri* Gürke** Habit: Herb. Habitat: Various habitats, ca. 0–300 m. Vouchers: Loveridge JP 13, Drummond & Hemsley 1074 (EA).

***Polygala irregularis* Boiss.** Habit: Herb. Habitat: *Acacia*-*Commiphora* bushland on dunes or white sand, 0–500 m. Vouchers: Luke 5441, Luke PA & WRQ 5441 (EA).

***Polygala kilimandjarica* Chodat** Habit: Herb. Habitat: *Acacia*-*Commiphora* bushland, secondary bushland, ca. 0–300 m. Vouchers: Luke 3494, Festo L & Luke Q 2658, Luke WRQ 3156 (EA).

***Polygala meonantha* Chodat** Habit: Herb. Habitat: Scattered tree grassland, *Acacia* bushland, woodland, 0–700 m. Voucher: Harvey, Mwachala & Vollesen 59 (EA).

***Polygala paniculata* L.** Habit: Herb. Habitat: Cultivated land, grassland, path and roadsides, ca. 350 m. Voucher: Magogo & Estes 1231 (EA).

***Polygala petitiana* A. Rich.** Habit: Herb. Habitat: *Brachystegia*-*Julbernadia* woodland, grassland, and cultivated ground, ca. 15 m. Vouchers: Robertson 3313, Luke PA & WRQ 5974, Luke 10344K (EA).

***Polygala sadebeckiana* Gürke** Habit: Herb. Habitat: Riverine forest, bushland, and forest margin, ca. 10–200 m. Voucher: Magogo & Glover 996 (EA).

***Polygala sansisbarensis* Gürke** Habit: Herb. Habitat: Wet places and seasonally wet grassland, 0–10 m. Vouchers: Tweedie 3181, Luke & Gray 4054, 2^nd^ Herbarium Technique Course 075, Gillett JB 20433 (EA).

***Polygala senensis* Klotzsch** Habit: Herb. Habitat: Scattered tree grassland, 0–500 m. Voucher: Gillet 16535 (EA).

***Polygala sphenoptera* Fresen.** Habit: Herb. Habitat: Evergreen bushland, wooded grassland, ca. 0–227 m. Vouchers: SAJIT–005941, Ngumbau V & Mwadime N V0277 (EA, HIB), Drummond & Hemsley 1074, Festo L, Luke Q & P 2636 (EA).

***Polygala stenopetala* Klotzsch** Habit: Herb. Habitat: *Brachystegia*-*Julbernadia* woodland, grassland, and cultivated ground, 0–280 m. Vouchers: SAJIT–005937, Ngumbau V & Mwadime N V0248 (EA, HIB), Verdcourt 3911, Magogo & Glover 394 (EA).

***Securidaca longipedunculata* Fresen.** Habit: Shrub. Habitat: Wooded and bushed grassland, ca. 0–300 m. Vouchers: SAJIT–005480 (EA, HIB), Greenway 9006, Magogo FC & Glover PE 169 (EA).

**F142. Polygonaceae**

3 Genera, 7 Species

***Antigonon leptopus* Hook. & Arn.** Habit: Climber. Habitat: Thickets, rainforest margins, and coastal sand dunes, 0–600 m. Vouchers: Kimeu JM 538, Starzenska 6 (EA).

***Oxygonum atriplicifolium* (Meisn.) Martelli** Habit: Herb. Habitat: Hedgerows, cultivated, and waste ground. Vouchers: Bogdan 3298, Verdcourt 1087, Tweedie 942, Verdcourt B 2112 (EA).

***Oxygonum sagittatum* R.A. Graham** Habit: Herb. Habitat: Sand ground, disturbed area, grassland, ca. 150 m. Voucher: Kokwaro JO 3571 (EA).

***Oxygonum salicifolium* Dammer** Habit: Herb. Habitat: Grasslands and disturbed ground, 0–156 m. Vouchers: SAJIT–004676, Ngumbau V & Mwadime N V0433 (EA, HIB), Kassner 262, RM Graham 2138, Simpson BL 259 (EA).

***Oxygonum stuhlmannii* Dammer** Habit: Herb. Habitat: Roadsides, ca. 88 m. Vouchers: SAJIT–006452 (EA, HIB), Reitsma J 419 (EA).

***Persicaria decipiens* (R.Br.) K.L. Wilson** Habit: Herb. Habitat: Waste land and roadsides, ca. 0–213 m. Vouchers: Luke WRQ 3599, Simpson BL 259, Magogo FC & Glover PE 921 (EA).

**Persicaria senegalensis f. albotomentosa (R.A. Graham) K.L. Wilson** Habit: Herb. Habitat: In damp places, lakes and rivers, ca. 60 m. Vouchers: Perdue RE & Kibuwa SP 10 & 159, Luke PA & WRQ 3826 (EA).

**F143. Pontederiaceae**

1 Genus, 1 Species

***Monochoria africana* (Solms) N.E.Br.** Habit: Herb. Habitat: Margins of rice fields and cleared mangrove forest, 0–100 m. Voucher: Greenway & Rawlins 9483 (EA).

**F144. Portulacaceae**

1 Genus, 11 Species

***Portulaca ciferrii* Chiov.** Habit: Herb. Habitat: Sand patches on coral rag pavement, ca. 5 m. Voucher: Greenway & Rawlins 9420 (EA).

***Portulaca coralloides* S.M. Phillips** Habit: Herb. Habitat: Deciduous bushland on coral and between coastal dunes, near sea level. Vouchers: Greenway & Rawlins 9423, Rawlins 94 (EA): Endemic.

***Portulaca fascicularis* Peter** Habit: Herb. Habitat: Coastal deciduous bushland, 0–450 m. Voucher: Faden et al. 70/939 (EA).

***Portulaca foliosa* Ker Gawl.** Habit: Herb. Habitat: Seasonally dry river beds or sandbanks in rivers, ca. 20 m. Voucher: Katz SS 75/2/11 (EA).

***Portulaca greenwayi* M.G. Gilbert** Habit: Herb. Habitat: Deciduous bushland, 50–150 m. Voucher: Ochung & Koech 86 (EA).

**Portulaca kermesina N.E.Br. var. kermesina** Habit: Herb. Habitat: Bushland, ca. 0–25 m. Vouchers: Rawlins SP 94 & 932, Graham RM 1623 (EA).

**Portulaca kermesina var. lutea (Poelln.) S.M. Phillips** Habit: Herb. Habitat: Grassland, wooded grassland, ca. 30 m. Voucher: Luke et al. TPR 253 (EA).

***Portulaca oblonga* Peter** Habit: Herb. Habitat: Open bushland and disturbed weedy places, ca. 0–65 m. Vouchers: Polhill & Paulo 640, RB & AJ Faden 74/1037 (EA).

***Portulaca oleracea* L.** Habit: Herb. Habitat: Seasonally flooded grassland or sandy areas, ca. 0–20 m. Vouchers: Kassner 463, Tweedie 935, Robertson SA 3755 (EA).

***Portulaca peteri* Poelln.** Habit: Herb. Habitat: Deciduous bushland and thicket, ca. 30 m. Voucher: Luke et al. TPR401 (EA).

***Portulaca quadrifida* L.** Habit: Herb. Habitat: Open disturbed grounds, ca. 50 m. Vouchers: Robertson SA 3756 & 3455 (EA).

***Portulaca wightiana* Wall. ex Wight & Arn.** Habit: Herb. Habitat: Rock outcrops, 0–100 m. Voucher: RB & AJ Faden 72/93 (EA).

**F145. Primulaceae**

1 Genus, 1 Species

***Myrsine melanophloeos* (L.) R. Br.** Habit: Tree. Habitat: Upland forest, riverine, swamp forest, open woodland, and thickets, ca. 100 m. Voucher: Sangai GW 1125 (EA).

**F146. Putranjivaceae**

1 Genus, 4 Species

**Drypetes natalensis var. leiogyna Brenan** Habit: Tree. Habitat: Lowland dry evergreen, semi-deciduous forest, and riverine forest, 15–380 m. Vouchers: SAJIT–005442 & 006095 (EA, HIB), Magogo FC & Glover PE 286 (EA): Vulnerable.

***Drypetes parvifolia* (Müll.Arg.) Pax & K. Hoffm.** Habit: Tree. Habitat: Evergreen semi-deciduous coastal and riverine forest, 0–450 m. Vouchers: SAJIT–005539, 005550, 005935 & 006192 (EA, HIB), Verdcourt 1893, RB & AJ Faden 71/773 (EA).

***Drypetes reticulata* Pax** Habit: Tree. Habitat: Evergreen forest and thicket, 0–500 m. Vouchers: Greenway 9621, Simpson 28 (EA).

**Drypetes usambarica (Pax) Hutch. var. usambarica** Habit: Tree. Habitat: Rainforest, 400 m. Voucher: Polhill R & Robertson 4836 (EA).

**Drypetes usambarica var. mrimae Radcl.-Sm.** Habit: Tree. Habitat: Lowland evergreen forest, 100–250 m. Vouchers: Luke WRQ & Robertson SA 523, Robertson SA & Luke Q 5842, RB & AJ Faden 77/677 (EA).

**Drypetes usambarica var. trichogyna Radcl.-Sm.** Habit: Tree. Habitat: Lowland to sub-montane moist forest. Voucher: Luke WRQ & Robertson SA 250 (EA): Vulnerable.

**F147. Ranunculaceae**

1 Genus, 1 Species

***Clematis sigensis* Engl.** Habit: Climber. Habitat: Forest edges and bushland, ca. 350–500 m. Vouchers: Luke WRQ 1625, Gillett JB 18715 (EA).

**F148. Rhamnaceae**

5 Genera, 9 Species

***Berchemia discolor* (Klotzsch) Hemsl.** Habit: Tree. Habitat: Grassland and open woodland, ca. 5 m. Voucher: Mrs Robertson SA 7743 (EA).

***Colubrina asiatica* (L.) Brongn.** Habit: Shrub. Habitat: Just above high-tide level. Vouchers: SAJIT–005596 (EA, HIB), Verdcourt 3963 & 1073, Ossent 239, Luke Q 5686 (EA).

***Lasiodiscus mildbraedii* Engl.** Habit: Tree. Habitat: Dry evergreen rainforest, dry forest, and woodland, ca. 20 m. Voucher: Thomas Mwadime in Mrs Robertson SA 7782 (EA).

**Lasiodiscus pervillei subsp. ferrugineus (Verdc.) Figueiredo** Habit: Shrub. Habitat: Lowland evergreen forest, ca. 100 m. Vouchers: Verdcourt 1181, Greenway & Rawlins 8954, Dale in FD 8834, Rawlins SP 241, Luke Q 1555 (EA).

***Scutia myrtina* (Burm.f.) Kurz** Habit: Shrub. Habitat: Forest margins, bushland, thicket, and wooded grassland, ca. 0–370 m. Vouchers: SAJIT–005573 (EA, HIB), Polhill & Paulo 497, Robertson SA 4377 (EA).

***Ziziphus mauritiana* Lam.** Habit: Tree. Habitat: Disturbed areas, ca. 30 m. Vouchers: SAJIT–005438 & 005439 (EA, HIB), RM Graham 245, Bally PRO 215 (EA).

***Ziziphus mucronata* Willd.** Habit: Tree. Habitat: Open scrubland, woodland, forest margins, and riverine vegetation, ca. 5 m. Vouchers: Mrs Robertson SA 7717, Magogo FC & Glover PE 887 (EA).

***Ziziphus pubescens* Oliv.** Habit: Tree. Habitat: Coastal forest, sand forest, and riverine fringes, ca. 0–300 m. Voucher: Verdcourt B 2410 (EA).

***Ziziphus robertsoniana* Beentje** Habit: Tree: Habitat: Moist semi-deciduous forest on coral rag, 10–160 m. Vouchers: Robertson SA 3645, 4274, 4310, 4889, 4675, 5097 & 6601, Mrima-Dzombo Expedition 170 (EA): Endangered.

**F149. Rhizophoraceae**

4 Genera, 5 Species

***Bruguiera gymnorhiza* (L.) Lam.** Habit: Tree. Habitat: Intertidal mud-flats and estuaries, up to 10 m. Vouchers: SAJIT–005584 (EA, HIB), Greenway PJ & Rawlins SP 8871 (EA).

***Cassipourea celastroides* Alston** Habit: Shrub. Habitat: On rocky hillsides, ca. 290 m. Vouchers: Kuchar P 13438, Robertson SA 5197, Luke WRQ & Robertson SA 283 (EA).

***Cassipourea euryoides* Alston** Habit: Tree. Habitat: Coastal forest, scrub or rarely in secondary bushland, 80–400 m. Vouchers: SAJIT–006012 & 006045, Ngumbau V & Mwadime N V0144 (EA, HIB), RM Graham 1627, Drummond & Hemsley 3964, Dale 3549, Gilbert MG & Kuchar P 5832, Greenway PJ 8934 (EA).

***Ceriops tagal* (Perr.) C.B. Rob.** Habit: Tree. Habitat: Intertidal mud flats and estuaries, sea level. Vouchers: MacNaughton 129 in Forestry Dept. 2715, Dale IR 1072 (EA).

***Rhizophora mucronata* Lam.** Habit: Tree. Habitat: Intertidal mud-flats of shores and estuaries, sea level. Vouchers: SAJIT–006247 (EA, HIB), Jeffery K136, Ross KS 203 (EA).

**F150. Rubiaceae**

63 Genera, 137 Species

***Afrocanthium kilifiense* (Bridson) Lantz** Habit: Shrub. Habitat: Dry lowland forest or *Brachystegia* woodland, 30–130 m. Vouchers: Ngumbau V & Mwadime N V0154 (EA, HIB), Luke Q 1516, Luke WRQ & Robertson SA 2789, RM Graham in FD 1711, Musyoki & Hansen 1012, Rawlins 856 (EA): Vulnerable.

***Afrocanthium peteri* (Bridson) Lantz** Habit: Shrub. Habitat: Forest or riverine thicket, 0–450 m. Voucher: Luke et al. TPR 659 (EA).

***Afrocanthium pseudoverticillatum* (S. Moore) Lantz** Habit: Shrub. Habitat: Bushland, ca. 0–297 m. Vouchers: SAJIT–006037, Ngumbau V & Mwadime N V0141 (EA, HIB), Festo L, Luke Q & P 2786 & 2601, Luke WRQ & Robertson SA 2790, Kassner 383, Faden 71/713, Robertson & Luke 5616 (EA).

***Agathisanthemum bojeri* Klotzsch** Habit: Herb. Habitat: Woodland, grassland, and riverine forest, 25–50 m. Vouchers: SAJIT–006050 & 005473 (EA, HIB), Luke WRQ & Robertson SA 2757 (EA).

***Aidia abeidii* S.E. Dawson & Gereau** Habit: Tree. Habitat: Forest, 8–360 m. Vouchers: Luke Q 2948, 8317, Festo L, Luke Q & P 2762 (EA).

***Breonadia salicina* (Vahl) Hepper & J.R.I. Wood** Habit: Tree. Habitat: Gallery forest by rivers, 0–100 m. Vouchers: Sampson 8, Luke WRQ 3597 (EA).

***Bullockia mombazensis* (Baill.) Razafim., Lantz & B. Bremer** Habit: Shrub. Habitat: Coastal bushland, wooded grassland, and evergreen forest, ca. 256 m. Vouchers: SAJIT–004642, Ngumbau V & Mwadime N V0137 (EA, HIB), Spjut RW 4579, Gillet & Kibuwa 20023, Gillespie 374, Luke Q 1437 (EA).

***Bullockia setiflora* (Hiern) Razafim., Lantz & B. Bremer** Habit: Shrub. Habitat: Woodland, riverine forests, 60–750 m. Vouchers: SAJIT–006125 (EA, HIB), Luke WRQ & Robertson SA 2808, Faden 77/675, Polhill & Robertson 4842 (EA).

***Calycosiphonia spathicalyx* (K. Schum.) Robbr.** Habit: Shrub. Habitat: Forest, ca. 250–400 m. Vouchers: Magogo & Glover 1100, Luke WRQ 8330 (EA).

**Canthium glaucum Hiern subsp. glaucum** Habit: Shrub. Habitat: Coastal forest, 10–225 m. Vouchers: Ngumbau V & Mwadime N V0463 (EA, HIB), Gisau SOK 22, RM Graham in FD 2352, Langridge 30, Luke WRQ 3159, Graham RM 1327 (EA).

***Canthium mrimaense* (Verdc.) Lantz** Habit: Shrub. Habitat: Evergreen forest, moist semi-deciduous forest, 10–200 m. Vouchers: Ngumbau V & Mwadime N V0500 (EA, HIB), Robertson 7, Luke 5897, Robertson et al. in MDE 14, Robertson & Luke 5868, Festo L & Luke Q 2599, Luke WRQ & PA 4386 (EA): Endemic.

***Catunaregam nilotica* (Stapf) Tirveng.** Habit: Shrub. Habitat: Riverine, thicket edges, woodland, coastal bushland, and scattered tree grassland, ca. 300 m. Vouchers: Greenway 9660, Spjut 3953, J Adamson 287, Robertson SA 3530 (EA).

***Catunaregam spinosa* (Thunb.) Tirveng.** Habit: Shrub. Habitat: Lowland evergreen forest, 70–290 m. Vouchers: SAJIT–006018, Ngumbau V & Mwadime N V0176 (EA, HIB), Verdcourt 1859, Faden 70/245, Gillett & Kibuwa 19930, Festo L, Luke Q & P 2756 (EA).

**Chassalia umbraticola Vatke subsp. umbraticola** Habit: Shrub. Habitat: Moist forest, 0–450 (–800) m. Vouchers: SAJIT–004655 & 005563, Ngumbau V & Mwadime N V077 & V0210 (EA, HIB), Luke Q 1518, Luke WRQ 8321, Napper 1377, Verdcourt 3921 (EA).

**Chassalia umbraticola subsp. geophila Verdc.** Habit: Shrub. Habitat: Moist forest, ca. 250 m. Voucher: Mrs SA Robertson & Luke Q 4511 (EA).

***Cladoceras subcapitatum* (K. Schum. & K. Krause) Bremek.** Habit: Shrub. Habitat: Evergreen forest and bushland, ca. 0–200 m. Vouchers: Joanna in CM 5952, Luke WRQ & Robertson SA 2648 (EA).

***Coffea pseudozanguebariae* Bridson** Habit: Shrub. Habitat: Forest and coastal bushland, 0–800 m. Vouchers: Ngumbau V & Mwadime N V0495 (EA, HIB), Kibuwa 1225, Magogo & Glover 421, Adams 105, Goodrich J 17167, Luke WRQ 3479 (EA): Near Threatened.

***Coffea rhamnifolia* (Chiov.) Bridson** Habit: Shrub. Habitat: *Acacia*-*Commiphora* bushland, ca. 95 m. Vouchers: Makin in EAH 13041, Gillett 16505 (EA).

**Coffea sessiliflora Bridson subsp. sessiliflora** Habit: Shrub. Habitat: Forest, 0–450 m. Vouchers: Magogo & Glover 209, Dale in FD 3817, Rawlins 216, Festo L, Luke Q & P 2669, Luke WRQ 3478, Katende AB 1769 (EA).

**Coptosperma graveolens (S. Moore) Degreef var. graveolens** Habit: Shrub. Habitat: Forest, ca. 40 m. Vouchers: Luke Q 1417, Ross KS 126 (EA).

***Coptosperma littorale* (Hiern) Degreef** Habit: Shrub. Habitat: Coastal bushland or foreshore, 0–105 m. Voucher: RM Graham in FD 2209 (EA).

***Coptosperma supra-axillare* (Hemsl.) Degreef** Habit: Shrub. Habitat: Forest and also inner borders of mangrove swamps, 0–400 m. Vouchers: Ngumbau V & Mwadime N V094 & V0153 (EA, HIB), Magogo & Glover 590/B, RM Graham in FD 2129, Rawlins 855, Festo L, Luke Q & P 2743, Robertson SA & Luke WRQ 6008 (EA).

***Cordylostigma longifolium* (Klotzsch) Groeninckx & Dessein** Habit: Herb. Habitat: Deciduous woodland, ca. 15–400 m. Vouchers: Polhill & Paulo 603, Magogo & Glover 826, Whyte (EA).

***Cordylostigma obtusilobum* (Hiern) Groeninckx & Dessein** Habit: Herb. Habitat: Coarse grassland, 0–270 m. Vouchers: Napier in CM 6291, Hildebrandt 1968, Jeffery 250, Magogo FC & Glover PE 995 (EA).

***Cordylostigma prolixipes* (S. Moore) Groeninckx & Dessein** Habit: Herb. Habitat: Rocky places, bushland, and grassland, ca. 120 m. Voucher: RM Graham in FD 1720 (EA).

***Cordylostigma virgatum* (Willd.) Groeninckx & Dessein** Habit: Herb. Habitat: Coastal bushland, deciduous woodland, and rice fields, 0–600 m. Vouchers: Verdcourt 3286, Rawlins 614 & 650, Magogo FC & Glover PE 473 (EA).

**Cremaspora triflora subsp. confluens (K. Schum.) Verdc.** Habit: Shrub. Habitat: Evergreen forest, fringing forest, and bush thicket, 0–700 m. Vouchers: SAJIT–006087, Ngumbau V & Mwadime N V074 (EA, HIB), Dale in FD 3559, RM Graham in FD 2072, Rawlins 280, Mutanga JG, Kamau 7, Magogo FC & Glover PE 76 (EA).

***Crossopteryx febrifuga* (Afzel. ex G. Don) Benth.** Habit: Tree. Habitat: Deciduous woodland and wooded grassland, ca. 0–400 m. Vouchers: SAJIT–005482 & 005973, Ngumbau V & Mwadime N V0138 (EA, HIB), Drummond & Hemsley 1209, RM Graham in FD 1696, Donald in FD 2372, Luke WRQ 8323 (EA).

***Didymosalpinx norae* (Swynn.) Keay** Habit: Shrub. Habitat: Evergreen forest, secondary forest, forest edges, 190–810 m. Vouchers: SAJIT–006031, Ngumbau V & Mwadime N V0310 (EA, HIB), Verdcourt 1854, Magogo & Glover 225 & 857, Luke WRQ 878 (EA).

***Diodella sarmentosa* (Sw.) Bacigalupo & Cabral ex Borhidi** Habit: Herb. Habitat: Evergreen forest, 0–280 m. Vouchers: SAJIT–005989, Ngumbau V & Mwadime N V0260 (EA, HIB), Drummond RB & Hemsley JH 1205 (EA).

**Diodia aulacosperma K. Schum. var. aulacosperma** Habit: Herb. Habitat: Grassland and bushland, 0–30 m. Vouchers: Davis 91, Tweedie 3163, Verdcourt 2115, Luke Q 5647 (EA).

**Diodia aulacosperma var. angustata Verdc.** Habit: Herb. Habitat: Grassland and bushland, 0–5 m. Vouchers: Napier 3321 in CM 6276, Rawlins 779, Gillespie 28, Festo L, Luke Q & P 2779 (EA).

**Empogona ovalifolia (Hiern) Tosh & Robbr. var. ovalifolia** Habit: Shrub. Habitat: Coastal evergreen or mixed formations, 0–80(–150) m. Vouchers: SAJIT–005572, Ngumbau V & Mwadime N V0136 (EA, HIB), Gillett 21047, Ng’weno 10, Moggridge 333, Luke Q 1432 (EA).

**Empogona ovalifolia var. glabrata (Oliv.) Tosh & Robbr.** Habit: Shrub. Habitat: Dry thickets, wooded grassland, and evergreen forest, ca. 400 m. Vouchers: Brenan, Gillett, Kanuri & Chomba 14620, Tweedie 3192 (EA).

**Empogona ovalifolia var. taylorii (S. Moore) Tosh & Robbr.** Habit: Shrub. Habitat: Thicket, dry forest, and bushland, ca. 0–300 m. Vouchers: RM Graham in FD 2172, WE Taylor, Robertson SA, Luke WRQ & Awimbo J 5237 (EA).

**Eumachia abrupta (Hiern) Delprete & J.H. Kirkbr. var. abrupta** Habit: Shrub. Habitat: Evergreen forest, *Combretum*, *Acacia* and *Brachystegia* woodland, ca. 30–200 m. Vouchers: SAJIT–005565, Ngumbau V & Mwadime N V0351 (EA, HIB), Magogo & Glover 375, Kassner 393, Bally 8850, Graham MD 2339 (EA).

**Eumachia abrupta var. parvifolia (Verdc.) C.M. Taylor** Habit: Shrub. Habitat: Presumably evergreen forest, ca. 50 m. Vouchers: Wakefield, Swynnerton 257 (K29), RM Graham in FD 2339 (EA).

**Feretia apodanthera subsp. keniensis Bridson** Habit: Shrub. Habitat: Coastal forest and bushland, 0–30 m. Vouchers: Gillett 18651, Bally 12195, MacNaughtan 9 (EA).

***Galiniera saxifraga* (Hochst.) Bridson** Habit: Tree. Habitat: Forest, ca. 2 m. Voucher: SAJIT–006239 (EA, HIB).

***Gardenia fiorii* Chiov.** Habit: Shrub. Habitat: *Commiphora*-*Acacia* open scrub, 60–750 m. Vouchers: Gillett 16501, Makin in EAH 13049 (EA).

***Gardenia posoquerioides* S. Moore** Habit: Shrub. Habitat: Evergreen forest, *Brachystegia* woodland, ca. 250–400 m. Vouchers: SAJIT–006124 (EA, HIB), Drummond & Hemsley 3875, Magogo & Glover 939, van Someren 313, Luke WRQ 1649 (EA).

**Gardenia ternifolia subsp. jovis-tonantis (Welw.) Verdc.** Habit: Tree. Habitat: Grassland, bushland, *Brachystegia* and *Acacia* woodland, 0–2100 m. Voucher: Bally & Smith 14346 (EA).

***Gardenia transvenulosa* Verdc.** Habit: Shrub. Habitat: Dry lowland evergreen forest, woodland, and bushland, 10–450 (–700) m. Vouchers: Greenway 9809, RB & AJ Faden 71/748, Moggridge 135, Musyoki BM & Hansen OJ 997 (EA).

**Gardenia volkensii K. Schum. var. volkensii** Habit: Tree. Habitat: Thicket and dry woodland, ca. 0–160 m. Vouchers: Polhill & Paulo 642, Graham RM 1584 (EA).

**Geophila obvallata subsp. ioides (K. Schum.) Verdc.** Habit: Herb. Habitat: Coastal evergreen forest and lowland rainforest, ca. 80–350 m. Vouchers: Drummond & Hemsley 3892, Verdcourt 3920, Magogo & Glover 1018, Luke WRQ 1631 (EA).

***Geophila repens* (L.) I.M. Johnst.** Habit: Herb. Habitat: Evergreen forest floors, 80–300 m. Vouchers: SAJIT–006148 (EA, HIB), Verdcourt 3939A, Drummond & Hemsley 3801, Napper 1379, Magogo FC & Glover PE 806 (EA).

***Guettarda speciosa* L.** Habit: Tree. Habitat: On sand and coral, sea level. Vouchers: Bally 8906, Napier in CM 3268, Verdcourt 1060 (EA).

**Heinsenia diervilleoides K. Schum. subsp. diervilleoides** Habit: Tree. Habitat: Rainforest and moist evergreen forest, ca. 375 m. Vouchers: SAJIT–006069, Ngumbau V & Mwadime N V056 (EA, HIB), Magogo & Glover 309, Luke WRQ & Robertson SA 2734 (EA).

**Heinsia crinita subsp. parviflora (K. Schum. & K. Krause) Verdc.** Habit: Shrub. Habitat: Bushland and degraded coastal *Brachystegia*, 15–660 m. Vouchers: SAJIT–006454, Ngumbau V & Mwadime N V0165 (EA, HIB), Magogo & Glover 865, Hildebrandt 1983, Polhill & Paulo 840 (EA).

***Heinsia zanzibarica* (Bojer) Verdc.** Habit: Shrub. Habitat: Forest edges, 90–480 (–700) m. Vouchers: Ngumbau V & Mwadime N V064 & 0177 (EA, HIB), Drummond & Hemsley 1110, Battiscombe 72, Magogo & Glover 1065, Luke WRQ 8322 (EA).

***Hymenodictyon parvifolium* Oliv.** Habit: Tree. Habitat: Mixed bushland and thicket, ca. 0–200 m. Vouchers: Drummond & Hemsley 4230, Robertson SA 4234 (EA).

***Ixora narcissodora* K. Schum.** Habit: Shrub. Habitat: Riverine forest, thickets, and seashore, ca. 240–400 m. Vouchers: SAJIT–006040, Ngumbau V & Mwadime N V0313 & 014 (EA, HIB), Drummond & Hemsley 1153, Magogo & Glover 78, Faden 72/63, Luke WRQ 8324 (EA).

***Keetia gueinzii* (Sond.) Bridson** Habit: Liana. Habitat: Forest, woodland, often on swampy ground, ca. 90–300 m. Vouchers: Ngumbau V & Mwadime N V026 (EA, HIB), Drummond & Hemsley 1138, Magogo FC & Glover PE 571 (EA).

***Keetia lukei* Bridson** Habit: Shrub. Habitat: Mixed forest, 40–400 m. Vouchers: Luke 3080, 3397 & 3302, Luke & Robertson 2634 (EA).

***Keetia venosa* (Oliv.) Bridson** Habit: Liana. Habitat: Forest edges and scrub, ca. 275 m. Vouchers: SAJIT–005500, 005506 & 006035, Ngumbau V & Mwadime N V0174 (EA, HIB), Spjut 4582, Gillet 18704, Gardner 1435, Magogo FC & Glover PE 66 (EA).

**Keetia zanzibarica (Klotzsch) Bridson subsp. zanzibarica** Habit: Shrub. Habitat: Coastal bushland, thickets, and forest edges, 0–500 m. Vouchers: SAJIT–005465, Ngumbau V & Mwadime N V081 (EA, HIB), Bally & Smith 14351, Jeffery K104, Hooper & Townsend 1210, Luke WRQ & Robertson SA 223 (EA).

**Kohautia caespitosa subsp. amaniensis (K. Krause) Govaerts** Habit: Herb. Habitat: Bushland, ca. 420 m. Vouchers: Magogo FC & Glover PE 666, GM Mungai & SM Rucina 223/84 (EA).

***Kraussia kirkii* (Hook.f.) Bullock** Habit: Shrub. Habitat: Forest and coastal bushland, 0–480 m. Vouchers: Dale in FD 3668, J Adamson 309 in Bally 5815, Power, Festo L, Luke Q & P 2680 (EA).

***Kraussia speciosa* Bullock** Habit: Shrub. Habitat: Forest, ca. 10–600 m. Vouchers: Magogo & Glover 435, Greenway & Rawlins 9348, Rawlins 326, Robertson SA & Luke WRQ 5532 (EA).

***Lamprothamnus zanguebaricus* Hiern** Habit: Shrub. Habitat: Wooded grassland, coastal bushland, thicket, and woodland, 0–300 m. Vouchers: Ngumbau V & Mwadime N V0451 (EA, HIB), Polhill & Paulo 908, Jex-Blake in Bally 5704, Polhill & Paulo 695, Kokwaro JO 3984 (EA).

***Leptactina platyphylla* (Hiern) Wernham** Habit: Shrub. Habitat: Evergreen forest, woodland, and secondary bushland, 45–400 m. Vouchers: Ngumbau V & Mwadime N V0146 (EA, HIB), Robertson SA 4671 (EA).

**Meyna tetraphylla subsp. comorensis (Robyns) Verdc.** Habit: Shrub. Habitat: Coastal bush on coral, dry or moist evergreen forest, 0–200 m. Voucher: Gillett & Kibuwa 19843 (EA).

***Mitracarpus hirtus* (L.) DC.** Habit: Herb. Habitat: Waste places, roadsides, ca. 418 m. Vouchers: Ngumbau V & Mwadime N V0126 (EA, HIB), Luke WRQ & PA 5692 (EA).

***Mitriostigma greenwayi* Bridson** Habit: Shrub. Habitat: Coastal forest, on coral rock, 0–150 m. Vouchers: SAJIT–005581, Ngumbau V & Mwadime N V0473 (EA, HIB), Brenan, Gillett et al. 14669, Hawthorne 247, Faden et al. 77/531 (EA): Endemic.

***Multidentia sclerocarpa* (K. Schum.) Bridson** Habit: Tree. Habitat: Forest, 100–550 m. Vouchers: SAJIT–006058, Ngumbau V & Mwadime N V0367 (EA, HIB), Luke & Robertson 530, Luke PA & WRQ 4327 (EA): Endangered.

**Mussaenda monticola K. Krause var. monticola** Habit: Shrub. Habitat: Evergreen forest, 45–820 m. Vouchers: SAJIT–006080 (EA, HIB), Magogo & Glover 1040, Spjut & Ensor 2739, Faden 74/1264, Luke WRQ & Robertson SA 1915, Luke WRQ et al. 4723 (EA).

***Nichallea soyauxii* (Hiern) Bridson** Habit: Tree. Habitat: Forest edges or coastal bushland, 0–350 m. Vouchers: Magogo & Glover 799, Gillett & Kibuwa 19857, Dale in FD 3670, Omondi W & Obunyali C 308 (EA).

**Oldenlandia affinis subsp. fugax (Vatke) Verdc.** Habit: Herb. Habitat: Grassland, swampy ground, bushland, and evergreen forest margins, ca. 0–240 m. Vouchers: Ngumbau V & Mwadime N V049 (EA, HIB), Drummond & Hemsley 1076, GM Jeffery 30, Gillespie 364, Magogo FC & Glover PE 1030 (EA).

**Oldenlandia corymbosa L. var. corymbosa** Habit: Herb. Habitat: Grassland and bushland, ca. 228 m. Voucher: Ngumbau V & Mwadime N V0120 (EA, HIB).

***Oldenlandia cryptocarpa* Chiov.** Habit: Herb. Habitat: Coastal bushland, ca. 15 m. Voucher: Polhill & Paulo 601 (EA).

**Oldenlandia fastigiata Bremek. var. fastigiata** Habit: Herb. Habitat: Grassland, thickets, and open *Acacia* woodland, ca. 0–200 m. Vouchers: Verdcourt 1870, Luke WRQ & PA 5723 (EA).

**Oldenlandia fastigiata var. pseudopentodon Verdc.** Habit: Herb. Habitat: Open damp grassland, ca. 15 m. Voucher: Gillett & Kibuwa 19900 (EA).

**Oldenlandia fastigiata var. somala (Chiov. ex Bremek.) Verdc.** Habit: Herb. Habitat: Grassland areas in *Acacia* bushland, ca. 60 m. Vouchers: Thairu 77, Thomas 23 (EA).

**Oldenlandia goreensis var. trichocaula Bremek.** Habit: Herb. Habitat: Edges of swamps, 0–375 m. Vouchers: Ngumbau V & Mwadime N V0550 (EA, HIB), Drummond & Hemsley 1162, Magogo & Glover 378, Luke Q 5624 (EA).

**Oldenlandia herbacea (L.) Roxb. var. herbacea** Habit: Herb. Habitat: Grassland, thicket, and bushland, ca. 0–300 m. Vouchers: Polhill & Paulo 485, Thulin M 301(EA).

***Oldenlandia ichthyoderma* Cufod.** Habit: Herb. Habitat: *Acacia*-*Commiphora* open bushland, 100 m. Voucher: Gillett 16518 (EA).

***Oldenlandia johnstonii* (Oliv.) K. Schum. ex Engl.** Habit: Herb. Habitat: Dry evergreen forest and wooded grassland, ca. 350 m. Voucher: Luke WRQ & Robertson SA 2702 (EA).

**Oldenlandia lancifolia var. scabridula Bremek.** Habit: Herb. Habitat: In moist or aquatic habitats, ca. 24 m. Vouchers: Ngumbau V & Mwadime N V0547 (EA, HIB), Gerhardt K & Steiner M 162, Luke WRQ & Pakia M 7459 (EA).

**Oldenlandia richardsonioides (K. Schum.) Verdc. var. richardsonioides** Habit: Herb. Habitat: On consolidated dunes, ca. 6 m. Vouchers: Gillespie 254, Luke Q 6127, Greenway & Rawlins 9289, Rawlins 107 (EA).

**Oldenlandia rosulata var. littoralis Verdc.** Habit: Herb. Habitat: Forest, ca. 50 m. Voucher: Symes 133 (EA).

**Oxyanthus goetzei subsp. keniensis Bridson** Habit: Shrub. Habitat: Bushland, forest, ca. 15 m. Vouchers: SAJIT–005542 (EA, HIB), JM Reitsma 200, RB & AJ Faden 77/662, Robertson 4882, Gray 106, Luke 5272 (EA).

**Oxyanthus pyriformis subsp. longitubus Bridson** Habit: Tree. Habitat: Forest, 240–450 m. Vouchers: SAJIT–006059 & 006151 (EA, HIB), Drummond & Hemsley 3961, Magogo & Glover 811, Magogo & Glover 812, Herbarium Techniques Course II–099 (EA).

***Oxyanthus zanguebaricus* (Hiern) Bridson** Habit: Shrub. Habitat: Forest, 60–240 m. Vouchers: Ngumbau V & Mwadime N V0214 (EA, HIB), Gisau SOK 25, Rawlins 225 & 259, Luke WRQ et al. 3329 (EA).

***Paracephaelis trichantha* (Baker) De Block** Habit: Shrub. Habitat: In coastal bushland, besides rivers, ca. 20 m. Vouchers: Luke Q 1436, Faden 74/1104, Marquis P, Festo L, Luke Q & P 2787 (EA).

**Pavetta crebrifolia Hiern var. crebrifolia** Habit: Shrub. Habitat: Coastal scrub or forest, ca. 0–304 m. Vouchers: Verdcourt 1904, Polhill & Paulo 801, Festo L, Luke Q & P 2655, Spjut RW 4584 (EA).

**Pavetta crebrifolia var. pubescens Bridson** Habit: Shrub. Habitat: Forest edge, ca. 30 m. Vouchers: Gillespie 327, Robertson SA & Luke WRQ 5606 (EA).

***Pavetta linearifolia* Bremek.** Habit: Shrub. Habitat: Coastal bushland or besides rivers, 50–100 m. Vouchers: Polhill & Paulo 821, Leroy 1039, Adamson 308 in Bally 5999, Luke, WRQ & Robertson SA 2508 (EA): Vulnerable.

**Pavetta sansibarica subsp. trichosphaera (Bremek.) Bridson** Habit: Shrub. Habitat: Bushland and forest, ca. 380 m. Vouchers: Luke WRQ et al. 4720, Luke WRQ 8326 & 4315 (EA).

**Pavetta sepium var. merkeri (K. Krause) Bridson** Habit: Shrub. Habitat: Forest, ca. 183 m. Vouchers: Ngumbau V & Mwadime N V0203 (EA, HIB), Hawthorne 186, Luke 5280 (EA).

**Pavetta sphaerobotrys subsp. tanaica (Bremek.) Bridson** Habit: Shrub. Habitat: Riverine forest, 30–350 m. Vouchers: Hamewood 8, Kibuwa 2500, Thairu 45 (EA).

**Pavetta stenosepala K. Schum. subsp. stenosepala** Habit: Shrub. Habitat: Evergreen forest, thickets, and bushland, 0–340 m. Vouchers: SAJIT–005978 & 005502, Ngumbau V & Mwadime N V105 (EA, HIB), Drummond & Hemsley 1207, RM Graham in FD 2149, Rawlins 425, Luke WRQ & Robertson SA 517, Luke WRQ & Robertson SA 225 (EA).

***Pavetta tarennoides* S. Moore** Habit: Shrub. Habitat: Undergrowth of coastal forest, 300–440 m. Vouchers: Brown 736, Magogo & Glover 40, Christensen 188, Luke WRQ & Robertson SA 2719 (EA): Vulnerable, Endemic.

***Pavetta uniflora* Bremek.** Habit: Shrub. Habitat: Coastal woodland or coastal bushland, 0–150 m. Vouchers: Bally 16739, RM Graham in FD 2136, Polhill & Paulo 859, Graham RM 1856 (EA).

***Pentas zanzibarica* (Klotzsch) Vatke** Habit: Herb. Habitat: Open grassland and forest edges, 0–600 m. Vouchers: Ngumbau V & Mwadime N V0198 (EA, HIB), Verdcourt 1867, Luke WRQ 8319 (EA).

**Pentodon pentandrus var. minor Bremek.** Habit: Herb. Habitat: Swamp lakes and river margins, ca. 0–50 m. Vouchers: SAJIT–005522 & 006226 (EA, HIB), Verdcourt 1887, Polhill & Paulo 890, Polhill & Paulo 538, Kirika P, Muthoka P & Mbale M 746, Luke WRQ & PA 5732 (EA).

**Polysphaeria lanceolata Hiern subsp. lanceolata** Habit: Shrub. Habitat: Riverine forest, ca. 15 m. Voucher: Faden et al. 77/485 (EA).

**Polysphaeria multiflora Hiern subsp. multiflora** Habit: Shrub. Habitat: Margins of shrubby thickets, mangrove swamps, forest, and woodland, ca. 0–200 m. Vouchers: Drummond & Hemsley 3913, Kassner 362, Bally 13051(EA).

**Polysphaeria multiflora subsp. pubescens Verdc.** Habit: Shrub. Habitat: Riverine forest and bushland, ca. 60 m. Vouchers: Homewood 32, Makin 14 (EA).

***Polysphaeria parvifolia* Hiern Habit**: Shrub. Habitat: Dry evergreen forest, woodland, coastal bushland, and scrub, 0–500 m. Vouchers: SAJIT–005540, 005495 & 005446 (EA, HIB), Drummond & Hemsley 1109, Wakefield, RM Graham in FD 1522, Robertson SA 4229 (EA).

**Psychotria amboniana K. Schum. var. amboniana** Habit: Shrub. Habitat: Coastal forest, thickets, scrub, and grassland with scattered trees, 0–300 m. Vouchers: SAJIT–006043, Ngumbau V & Mwadime N V0132 (EA, HIB), Lucas, Jeffery & Kirrika 243, Wakefield, Linder 2660, Rawlins 426, Robertson SA 4231, Luke Q 1451 (EA).

**Psychotria amboniana var. velutina (E.M.A. Petit) Verdc.** Habit: Shrub. Habitat: Coastal bushland, 0–160 m. Vouchers: RM Graham in FD 2337, Gillett & Kibuwa 19928, Rawlins 427 (EA).

**Psychotria capensis subsp. riparia (K. Schum. & K. Krause) Verdc.** Habit: Shrub. Habitat: Evergreen bushland, coastal bushland, and wooded grassland, ca. 20 m. Vouchers: Ngumbau V & Mwadime N V0309 (EA, HIB), Mwadime N 39, RM Graham in FD 2185 (EA).

**Psychotria capensis var. puberula (E.M.A. Petit) Verdc.** Habit: Shrub. Habitat: Coastal forest and thicket, ca. 80 m. Vouchers: Drummond & Hemsley 3951, Magogo & Glover 324 (EA).

***Psychotria crassipetala* E.M.A. Petit** Habit: Shrub. Habitat: Evergreen forest, ca. 30 m. Voucher: Dale in FD 3831 (EA).

***Psychotria faucicola* K. Schum.** Habit: Shrub. Habitat: Rainforest and forest edges, 15–750 m. Vouchers: SAJIT–006188, Ngumbau V & Mwadime N V0353 (EA, HIB), Napper 1375, Faden 70/242, Magogo & Glover 972, Faden RB 677A, Luke WRQ 868 (EA).

**Psychotria holtzii (K. Schum.) E.M.A. Petit var. holtzii** Habit: Shrub. Habitat: Evergreen forest, 0–360 (450) m. Vouchers: Verdcourt 1884, Drummond & Hemsley 3895 & 1190, Verdcourt 3915 (EA).

***Psychotria lauracea* (K. Schum.) E.M.A. Petit** Habit: Shrub. Habitat: Various types of evergreen forest, 0–268 m. Vouchers: SAJIT–005508 & 005983, Ngumbau V & Mwadime N V038 (EA, HIB), Drummond & Hemsley 1180, Luke WRQ & Robertson SA 245 (EA).

***Psychotria leucopoda* E.M.A. Petit** Habit: Shrub. Habitat: Rainforest, lowland fringing forest, and coastal evergreen forest, 45–300 m. Vouchers: SAJIT–006010, Ngumbau V & Mwadime N V0166 (EA, HIB), VG van Someren 7180, CF Elliot 202, Wakefield, Luke PA & WRQ 6295, Luke WRQ 897 (EA).

***Psychotria punctata* Vatke** Habit: Shrub. Habitat: Coastal bushland, savannah, and forest, mostly on sand or coral rag, 0–45 (–280) m. Vouchers: SAJIT–005576, 006233, 005600 & 006238 (EA, HIB), Napier 3252 in CM 6282, Tweedie 1045, Greenway & Rawlins 8907 (EA).

**Psychotria schliebenii var. parvipaniculata E.M.A. Petit** Habit: Shrub. Habitat: Evergreen forest, ca. 60 m. Voucher: Dale in FD 3570 (EA).

**Psychotria schliebenii var. sessilipaniculata E.M.A. Petit** Habit: Shrub. Habitat: Evergreen forest, ca. 0–442 m Vouchers: RM Graham in FD 2031, Magogo & Glover 14, Magogo & Glover 259 (EA).

**Psychotria tanganyicensis Verdc. subsp. tanganyicensis** Habit: Shrub. Habitat: Rainforest, riverine forest, 300–1530 m. Vouchers: SAJIT–006160 (EA, HIB), Luke WRQ 1847, Drummond & Hemsley 1194, Magogo & Glover 440, Moomaw 1140 (EA).

***Psydrax faulknerae* Bridson** Habit: Tree. Habitat: Coastal bush, thicket or *Brachystegia* woodland, 0–725 m. Vouchers: Ngumbau V & Mwadime N V0267 & V093 (EA, HIB), Magogo & Glover 726, RM Graham in FD 1823, Moggridge 332, Luke WRQ & Robertson SA 1543 (EA).

***Psydrax kaessneri* (S. Moore) Bridson** Habit: Shrub. Habitat: Riverine thickets and forest edges, (20–) 90–300 m. Vouchers: Gilllett & Kibuwa 19927, Medley 414, Luke & Robertson 1532, Festo L, Luke Q & P 2672 (EA).

***Psydrax polhillii* Bridson** Habit: Shrub. Habitat: Thickets and *Brachystegia* woodland, 15–350 m. Vouchers: Drummond & Hemsley 4163, Faden & Evans 71/708, Luke WRQ 9466 (EA).

***Psydrax recurvifolia* (Bullock) Bridson** Habit: Shrub. Habitat: Forest or at swamp edge, 0–10 m. Vouchers: RM Graham in FD 1751, Moggridge 534, SA Robertson 4652, Robertson SA & Luke WRQ 5993 (EA).

***Psydrax robertsoniae* Bridson** Habit: Shrub. Habitat: Thicket and forest, ca. 5 m. Vouchers: Moggridge 334, Hawthorne 273, SA Robertson 6152, Luke WRQ 3142 (EA): Endemic.

***Psydrax schimperiana* (A. Rich.) Bridson** Habit: Tree. Habitat: Forest, thicket, and bushland, ca. 15 m. Vouchers: RM Graham in FD 1995, Moomaw JC 1077 (EA).

***Psydrax* sp. A of FTEA** Habit: Shrub. Habitat: Sand dune thicket. Voucher: Luke & Robertson 1368 (EA): Endemic.

***Pyrostria bibracteata* (Baker) Cavaco** Habit: Tree. Habitat: Bushland or forest edges, 0–400 m. Vouchers: Ngumbau V & Mwadime N V0133 (EA, HIB), Magogo & Glover 294, Perdue & Kibuwa 10022, Gillett 20346, Festo L Luke Q 2523 (EA).

***Pyrostria phyllanthoidea* (Baill.) Bridson** Habit: Shrub. Habitat: *Acacia*-*Commiphora* bushland, ca. 20 m. Vouchers: Drummond & Hemsley 4170, Luke Q 1435 (EA).

***Rhodopentas bussei* (K. Krause) Kårehed & B. Bremer** Habit: Shrub. Habitat: Grassland, bushland, and woodland dry evergreen forest, ca. 0–400 m. Vouchers: Ngumbau V & Mwadime N V0217 & V088 (EA, HIB), Drummond & Hemsley 3813, Graham in FD 2065, Napier 3361 in CM 6286, Luke Q 1483 (EA).

***Rhodopentas parvifolia* (Hiern) Kårehed & B. Bremer** Habit: Shrub. Habitat: Dry bushland, 0–300 m. Vouchers: Robertson & Luke 1778, V de Meester-Manger Cats 243, Luke WRQ et al. 5418 (EA).

***Richardia scabra* L.** Habit: Herb. Habitat: Grassland, ca. 36 m. Voucher: SAJIT–006169 (EA, HIB).

***Rothmannia macrosiphon* (K. Schum. ex Engl.) Bridson** Habit: Tree. Habitat: Forest, 60–450 m. Vouchers: SAJIT–005928, Ngumbau V & Mwadime N V069 (EA, HIB), Drummond & Hemsley 3938 & 3875, RM Graham in FD1984, Luke WRQ & Robertson SA 312 & 235 (EA): Vulnerable.

***Rothmannia ravae* (Chiov.) Bridson** Habit: Tree. Habitat: In thicket or sometimes forest, 45–600 m. Vouchers: Dale K 206A, Gillespie 205, Festo L, Luke Q & P 2774, Luke WRQ 2932 (EA).

***Rutidea fuscescens* Hiern** Habit: Liana. Habitat: Evergreen forest, ca. 370 m. Voucher: Luke WRQ 895 (EA).

**Rytigynia celastroides (Baill.) Verdc. var. celastroides** Habit: Shrub. Habitat: Woodland, open forest, relict thickets, and lowland rainforest, 0–750 m. Vouchers: SAJIT–005467, 005468 & 005469, Ngumbau V & Mwadime N V0134 (EA, HIB), Verdcourt 1902, RM Graham BB383 in FD 1750, RM Graham N824 in FD 2336, Luke WRQ & Robertson SA 2795 (EA).

***Rytigynia decussata* (K. Schum.) Robyns** Habit: Shrub. Habitat: *Acacia*-*Combretum* and *Brachystegia* woodlands, thickets, and open grassland, ca. 75–300 m. Vouchers: Ngumbau V & Mwadime N V0131 (EA, HIB), Robertson & Luke 5183 (EA).

***Rytigynia parvifolia* Verdc.** Habit: Shrub. Habitat: Bushland and scattered tree grassland, 30–180 m. Vouchers: Rawlins 726, Greenway 9489, Luke Q 1450 (EA).

***Rytigynia* sp. I of FTEA** Habit: Shrub. Habitat: Coastal riverine forest, ca. 6 m. Vouchers: Homewood 62 & 64 (EA): Endemic.

***Rytigynia* sp. L of FTEA** Habit: Shrub. Habitat: Moist lowland forest, ca. 30 m. Voucher: Robertson & Luke 6309 (EA): Endemic.

***Spermacoce filituba* (K. Schum.) Verdc.** Habit: Herb. Habitat: Grassland with scattered trees, sea shore bushland on sand, and coconut groves, 0–600 m. Vouchers: SAJIT–006052 (EA, HIB), RM Graham in AD 1629, Polhill & Paulo 550, Whyte, Luke Q 5670, Magogo FC & Glover PE 715, Tiede 9 & 11 (EA).

***Spermacoce laevis* Lam.** Habit: Herb. Habitat: Grassland, along roadsides, and in rice field, ca. 0–418 m. Vouchers: Ngumbau V & Mwadime N V0127 (EA, HIB), Magogo FC & Glover PE 661 (EA).

***Spermacoce pusilla* Wall.** Habit: Herb. Habitat: Seasonally damp grassland, bushland, ca. 120 m. Voucher: McKeag in EAH 9180 (EA).

***Spermacoce* sp. B of FTEA** Habit: Herb. Habitat: Evergreen forest, ca. 225 m. Voucher: Bally 8546 (EA): Endemic.

***Tarenna drummondii* Bridson** Habit: Tree. Habitat: Forest or open woodland, 100–460 m. Vouchers: SAJIT–006072 & 006149, Ngumbau V & Mwadime N V0163 (EA, HIB), Drummond & Hemsley 3876, Magogo & Glover 492, Faden et al. 77/411, Luke WRQ & Kahumbu P 4546F (EA): Vulnerable.

**Tarenna pavettoides subsp. friesiorum (K. Krause) Bridson** Habit: Tree. Habitat: Forest. Voucher: Cunningham-van Someren GR Sh 245 (EA).

**Triainolepis africana subsp. hildebrandtii (Vatke) Verdc.** Habit: Shrub. Habitat: Mostly in coastal bushland near high-tide mark, 0 (–150) m. Vouchers: SAJIT–006107 & 005450 (EA, HIB), Verdcourt 3961, Ossent 242, Tweedie 2377 (EA).

**Tricalysia bridsoniana Robbr. var. bridsoniana** Habit: Shrub. Habitat: *Cynometra* forest, below 300 m. Vouchers: Spjut & Ensor 2789, Brenan, Gillett, Kanuri & Chomba 14685, Pdwa 26, Brenan JPM 14685, Luke WRQ 9463 (EA): Endemic.

***Tricalysia congesta* (Oliv.) Hiern** Habit: Shrub. Habitat: Sclerophyllous thicket, fire-protected woodland, and riverine forest, ca. 400 m. Voucher: Verdcourt 5289 (EA).

***Tricalysia microphylla* Hiern** Habit: Shrub. Habitat: Forest, ca. 300 m. Vouchers: Ngumbau V & Mwadime N V0314 (EA, HIB), Faden 77/716, Magogo & Glover 941, Gillett & Gachathi 77/413, Robertson SA & Luke, WRQ 5480, Luke WRQ 9457 (EA).

***Tricalysia pallens* Hiern** Habit: Shrub. Habitat: Evergreen forests, forest margins, and thickets, ca. 229 m. Vouchers: SAJIT–005443, Ngumbau V & Mwadime N V0160 (EA, HIB), Drummond & Hemsley 1197, Luke WRQ 867 (EA).

**Uncaria africana G. Don subsp. africana** Habit: Liana. Habitat: Secondary forest, ca. 18 m. Voucher: Luke WRQ & Robertson SA 2752 (EA).

**Vangueria infausta subsp. rotundata (Robyns) Verdc.** Habit: Tree. Habitat: Grassland with forest patches, ca. 10–200 m. Vouchers: Ngumbau V & Mwadime N V0200 (EA, HIB), Magogo FC & Glover PE 181, Luke Q 1511 (EA).

***Vangueria loranthifolia* K. Schum.** Habit: Shrub. Habitat: *Cynometra* forest, thicket, woodland, and bushland, 0–600 m. Vouchers: SAJIT–005974, Ngumbau V & Mwadime N V0234 & 0460 (EA, HIB), Napier 3305 in C.M.6281, Spjut & Ensor 2671, Polhill & Paulo 652, Gillett JB 20431, Luke WRQ 9464 (EA).

***Vangueria pallidiflora* (Bullock) Lantz** Habit: Shrub. Habitat: Evergreen forest, thickets, 0–600 m. Vouchers: SAJIT–006057 (EA, HIB), Drummond & Hemsley 3940, Magogo & Hemsley 441, Spjut 2731, Festo L, Luke Q & P 2657, Luke WRQ & Kahumbu P 4546D (EA): Vulnerable.

**Vangueria randii subsp. acuminata Verdc.** Habit: Shrub. Habitat: Evergreen forest, (0–) 30–300 m. Vouchers: SAJIT–005536 (EA, HIB), Magogo & Glover 437, Gillett 21053, Gillett & Kibuwa 19895 (EA).

***Vangueriopsis shimbaensis* A. P. Davis & Q. Luke** Habit: Tree. Habitat: Lowland forest, 360 m. Vouchers: Luke & Lehman 10894, Luke WQR 8316 (EA): Critically Endangered, Endemic.

**F151. Rutaceae**

5Genera, 17 Species

***Clausena anisata* (Willd.) Hook.f. ex Benth.** Habit: Tree. Habitat: Evergreen and fringing forest, 0–400 m. Vouchers: SAJIT–006044, Ngumbau V & Mwadime N V0170 (EA, HIB), Luke WRQ & Robertson SA 533 (EA).

***Vepris* (*Diphasia* sp. A of FTEA)** Habit: Tree. Habitat: Moist forest, ca. 5 m. Voucher: SAJIT–006094 (EA, HIB), Luke WRQ 3035 (EA): Endemic.

***Harrisonia abyssinica* Oliv.** Habit: Shrub. Habitat: Dry evergreen forest, forest edges, and clearings, 35–120 m. Vouchers: SAJIT–005459 & 005458 (EA, HIB), Magogo FC & Glover PE 21, Burstyn P 89 (EA).

***Toddalia asiatica* (L.) Lam.** Habit: Liana. Habitat: Forest edges, bushland, and wooded grassland, ca. 1–400 m. Vouchers: SAJIT–005452, Ngumbau V & Mwadime N V0237 (EA, HIB), Kokwaro 1983, Magogo FC & Glover PE 257 (EA).

***Vepris amaniensis* (Engl.) Mziray** Habit: Shrub. Habitat: Rainforest or forest-edge thickets, ca. 360 m. Vouchers: Magogo & Glover 1102, Lavranos J & Newton 12290, Malombe I, Mwadime N & Saidi 1652 (EA).

***Vepris eugeniifolia* (Engl.) I. Verd.** Habit: Shrub. Habitat: Coastal forest and bushland on coral rock, 0–750 m. Vouchers: Ngumbau V & Mwadime N V0299 (EA, HIB), Drummond & Hemsley 4063, Polhill & Paulo 610, Magogo FC & Glover PE 693 (EA).

**Vepris glomerata var. glabra Kokwaro** Habit: Shrub. Habitat: Wooded grassland and deciduous bushland, ca. 30 m. Voucher: Dale in FD 3853 (EA).

***Vepris lanceolata* (Lam.) G. Don** Habit: Tree. Habitat: Coastal evergreen thicket or on loose sandy soil along the beaches, 0–30 m. Voucher: Schultka K 149 (EA).

***Vepris robertsoniae* Q. Luke, ined.** Habit: Tree. Habitat: Lowland coastal forest, ca. 35 m. Vouchers: Luke Q 1539, Luke WRQ & Robertson SA 2806 (EA).

***Vepris sansibarensis* (Engl.) Mziray** Habit: Shrub. Habitat: Coastal thicket and forest, 1–760 m. Vouchers: Dale in FD 3661, Moomaw 1674, Rawlins 762, Muchiri J 440, Luke Q 1560, Luke WRQ 3135 (EA): Vulnerable.

***Vepris simplicifolia* (Engl.) Mziray** Habit: Tree. Habitat: Dry forest, riverine thicket or woodland, ca. 15 m. Vouchers: Ngumbau V & Mwadime N V0560 (EA, HIB), Karin Gerhardt & Mariette Steiner 263 (EA).

**Vepris sp. nr. stolzii I. Verd.** Habit: Tree. Habitat: Lowland and upland forest, ca. 100 m. Voucher: Luke WRQ & PA 4501 (EA).

***Vepris trichocarpa* (Engl.) Mziray** Habit: Tree. Habitat: Coastal and upland forests, 1–2300 m. Vouchers: SAJIT–004657 (EA, HIB), Greenway 10826, Luke WRQ & Mbinda J 5969 (EA).

***Zanthoxylum chalybeum* Engl.** Habit: Tree. Habitat: Dry bushland and wooded grassland, ca. 3 m. Vouchers: SAJIT–006447, Ngumbau V & Mwadime N V0158 (EA, HIB), Gilbert MG, Kuchar P 5873 (EA).

***Zanthoxylum holtzianum* (Engl.) P.G. Waterman** Habit: Tree. Habitat: Coastal forest, bushland, sometimes on coral, 1–230 m. Vouchers: Ngumbau V & Mwadime N V0535 (EA, HIB), Drummond & Hemsley 4006, Dale in FD 3567, Rawlins in EAH 11279, Robertson SA 3775, Festo L, Luke Q & P 2785 (EA).

***Zanthoxylum paracanthum* (Mildbr.) J. O. Kokwaro** Habit: Tree. Habitat: Evergreen forest, bushland, and riverine, ca. 140 m. Vouchers: Ngumbau V & Mwadime N V0534 (EA, HIB), Faden 234, Luke WRQ & Robertson SA 2801 (EA).

**F152. Salicaceae**

10 Genera, 15 Species

***Bivinia jalbertii* Tul.** Habit: Tree. Habitat: Open forest and coastal bushland, up to 200 m. Vouchers: Ngumbau V & Mwadime N V0246 (EA, HIB), Rawlins SP 321, Luke WRQ & Robertson SA 510 (EA): Near Threatened.

***Casearia gladiiformis* Mast.** Habit: Tree. Habitat: Dry evergreen, riverine, secondary forest, coastal woodland, and bushland, ca. 600 m. Vouchers: Luke WRQ & Robertson SA 1926, Luke WRQ & Robertson SA 511 (EA).

***Dovyalis abyssinica* (A. Rich.) Warb.** Habit: Shrub. Habitat: Dry evergreen forest, and open wooded grassland, ca. 15 m. Voucher: Gerhardt K & Steiner M 42 (EA).

***Dovyalis hispidula* Wild** Habit: Shrub. Habitat: Riverine thickets and *Brachystegia* woodland, ca. 150 m. Voucher: Rawlins SP 240 (EA).

***Dovyalis keniensis* E. V. Williams** Habit: Shrub. Habitat: In *Afzelia*-*Mimusops* forest, ca. 142 m. Vouchers: SAJIT–006004 (EA, HIB), Trump EC 109, Luke WRQ 3150 (EA).

***Dovyalis macrocalyx* (Oliv.) Warb.** Habit: Tree. Habitat: Rainforest, dry evergreen, riverine forest, bushland, and wooded grassland, ca. 0–400 m. Vouchers: SAJIT–006004 (EA, HIB), Sangai GW 15650, Luke WRQ 3160 (EA).

***Flacourtia indica* (Burm.f.) Merr.** Habit: Tree. Habitat: Woodland, wooded grassland and bushland, ca. 0–350 m. Vouchers: SAJIT–005606 & 006433 (EA, HIB), Festo L & Luke Q 2291, Greenway & Rawlins 8877 (EA).

***Homalium abdessammadii* Asch. & Schweinf.** Habit: Tree. Habitat: Forest or forest edge, ca. 200 m. Vouchers: Luke WRQ & PA 4582, Greenway PJ & Rawlins SP 9427 (EA).

***Homalium longistylum* Mast.** Habit: Tree. Habitat: Forest, forest edge, and riverine forest, 600–800 m. Voucher: Luke WRQ & Robertson SA 2281 (EA).

***Ludia mauritiana* J.F. Gmel.** Habit: Tree. Habitat: Dry evergreen forest and coastal bushland, ca. 0–300 m. Vouchers: SAJIT–005587 & 005441 (EA, HIB), Dale IR 3864, Greenway 9627 (EA).

***Oncoba spinosa* Forssk.** Habit: Tree. Habitat: Forest edges, riverine forest, bushland, and *Brachystegia* woodland, ca. 300 m. Voucher: Festo L & Luke Q 2535 (EA).

***Populus ilicifolia* (Engl.) Rouleau** Habit: Tree. Habitat: Riverine forest, mudflats, and sandbanks, ca. 30 m. Voucher: Gillett & Kibuwa 19912/A (EA).

***Scolopia rhamniphylla* Gilg** Habit: Shrub. Habitat: Rainforest or dry evergreen forest, ca. 300 m. Vouchers: Robertson SA 5457, Festo L & Luke Q 2529, Dale in FD 3866, Verdcourt 1896 (EA).

***Scolopia zeyheri* (Nees) Harv.** Habit: Tree. Habitat: Dry evergreen forest, riverine forest, and bushland, ca. 0–20 m. Vouchers: Luke WRQ & Robertson SA 1035, Luke 10769 (EA).

**Trimeria grandifolia (Hochst.) Warb. subsp. tropica (Burkill) Sleumer** Habit: Tree. Habitat: Dry evergreen or riverine forest, ca. 297 m. Vouchers: SAJIT–005494 & 006006, Ngumbau V & Mwadime N V0142 (EA, HIB), Luke WRQ & Robertson SA 507 (EA).

**F153. Salvadoraceae**

3 Genera, 4 Species

***Azima tetracantha* Lam.** Habit: Shrub. Habitat: Scrub, seasonal rivers, and coastal bushland, ca. 30 m. Voucher: Verdcourt 1179 (EA).

***Dobera glabra* (Forssk.) Poir.** Habit: Tree. Habitat: Dry forest, ca. 30–40 m. Vouchers: Kimberly Medley 386, Robertson SA 7734 (EA).

***Dobera loranthifolia* (Warb.) Harms** Habit: Tree. Habitat: In scrub and wooded grassland, 0–810 m. Voucher: Luke WRQ & PA sr.

**Salvadora persica L. var. persica** Habit: Shrub. Habitat: On saline, sandy or loamy soils, along large seasonal rivers, ca. 0–150 m. Vouchers: SAJIT–005906 & 006451 (EA, HIB), Jeffery 476 (EA).

**Salvadora persica var. cyclophylla (Chiov.) Cuf.** Habit: Shrub. Habitat: In scrub, wooded grassland, just above high tide level, ca. 0–50 m. Vouchers: Unknown collector in CM 242, Verdcourt 3599, Birch 62/170B, Gillespie 65, Boll K 16031(EA).

**F154. Santalaceae**

2 Genera, 2 Species

***Thesium subaphyllum* Engl.** Habit: Herb. Habitat: Grassland and on rocks, ca. 60–380 m. Vouchers: Faden, Evans & Mahasi 70/821, Luke WRQ 3312 (EA).

***Viscum triflorum* DC.** Habit: Herb. Habitat: Evergreen forest, 0–150 m. Voucher: Wiens 4538 (EA).

**F155. Sapindaceae**

20 Genera, 25 Species

***Allophylus chirindensis* Baker f.** Habit: Tree. Habitat: Evergreen forest, 350–850 m. Vouchers: Magogo & Glover 1098, Luke WRQ et al. 4715 (EA).

**Allophylus pervillei Blume f. pervillei** Habit: Shrub. Habitat: Fringing forest, forest clumps, ground water forest, and coastal *Brachystegia*, 0–550 m. Vouchers: SAJIT–006193 & 006144 (EA, HIB), SA Robertson 3502, RM Graham in FD 2126, Rawlins 275, Mutangah JG & Kamau P 14, Robertson SA 3502 (EA).

**Allophylus pervillei f. trifoliolatus Radlk.** Habit: Shrub. Habitat: Coastal forest bushland and thicket, 0–400 m. Vouchers: Drummond & Hemsley 3952, Bally 4685, Rawlins 349 (EA).

**Allophylus rubifolius var. alnifolius (Baker) Friis & Vollesen** Habit: Shrub. Habitat: Evergreen thicket and dry forest edges, 0–700 m. Vouchers: Polhill & Paulo 576, Ross KS 118 (EA).

**Allophylus rubifolius var. dasystachys (Gilg) Verdc.** Habit: Tree. Habitat: Dry evergreen forest, open woodland, bushland, and cultivations, 50–150 m. Vouchers: Luke Q 5669, Saufferer S 864 (EA).

***Aporrhiza paniculata* Radlk.** Habit: Tree. Habitat: Riverine forest, and ground water forest, ca. 20 m. Vouchers: Luke WRQ & Robertson SA 2762, Magogo & Glover 615, 753, F Hughes (EA).

***Blighia unijugata* Baker** Habit: Tree. Habitat: Evergreen forest, riverine or forest margins, ca. 0–20 m. Vouchers: Brenan et al. 14629, Mwadime N 55, Shimba Hills Survey Unit 75 (EA).

***Camptolepis ramiflora* (Taub.) Radlk.** Habit: Tree. Habitat: *Trichilia*, *Garcinia*, *Hyphaene* forest, 10–100 m. Vouchers: SA Robertson & Luke 5476, SA Robertson & Baer 7229, Mwadime N 49 (EA).

***Cardiospermum microcarpum* Kunth** Habit: Climber. Habitat: Coastal grassland, ca. 86 m. Vouchers: Ngumbau V & Mwadime N V0479 (EA, HIB), Kassner 339 (EA).

***Chytranthus obliquinervis* Radlk. ex Engl.** Habit: Tree. Habitat: Lowland evergreen forest, 5–400 m. Vouchers: SAJIT–006086 (EA, HIB), Verdcourt 3955, Drummond & Hemsley 1120, Luke Q 1515 (EA): Vulnerable.

***Chytranthus prieurianus* Baill.** Habit: Shrub. Habitat: Evergreen forest or forest on coral rag, 0–600 m. Vouchers: Drummond & Hemsley 3926, SA Robertson & Luke 4911, SA Robertson & Baer 7191 (EA).

***Deinbollia borbonica* Scheff.** Habit: Shrub. Habitat: Riverine *Acacia* thorn bush, evergreen thicket, and low evergreen forest, 0–210 m. Vouchers: Ngumbau V & Mwadime N V0395 (EA, HIB), Polhill & Paulo 861, Omondi W & Obunyali C 305 (EA).

**Dodonaea viscosa (L.) Jacq. subsp. viscosa** Habit: Shrub. Habitat: Coastal bushland, on sand dunes, and coral rocks, 0–30 (–75) m. Vouchers: SAJIT–005593 & 006258 (EA, HIB), Strange 74, RM Graham, Harry van der Hagen & Simpson 88 (EA).

**Dodonaea viscosa subsp. angustifolia (L.f.) J.G. West** Habit: Shrub. Habitat: Margins of evergreen forest, ca. 100 m. Vouchers: Gillett JB 20317, Robertson SA 7660 (EA).

***Glenniea africana* (Radlk.) Leenh.** Habit: Tree. Habitat: Wet evergreen forest, riparian forest, and bushland, ca. 30 m. Vouchers: Battiscombe 57, Luke WRQ 2929A (EA).

***Haplocoelopsis africana* F.G. Davies** Habit: Tree. Habitat: Lowland wet, dry forest, and thickets, 40–700 m. Vouchers: Dale IR 3820, Mrima-Dzombo Exped. 142, Luke & SA Robertson 1433 & 247 (EA).

**Haplocoelum foliolosum subsp. mombasense (Bullock) Verdc.** Habit: Tree. Habitat: Hillside grassland, *Acacia*-*Combretum* woodland, seashore bushland, 0–300 m. Vouchers: RB & AJ Faden 77/722, SA Robertson & Luke 5719, SA Robertson 6435 (EA).

***Haplocoelum inoploeum* Radlk.** Habit: Tree. Habitat: Coastal forest edges, semi-evergreen bushland, and thicket on coral rag, 0–600 m. Vouchers: Greenway 9622, SA Robertson 3866, Polhill & Paulo 671, Luke Q 1438, Sangai GW in EAH 15763 (EA).

**Lecaniodiscus fraxinifolius subsp. scassellatii (Chiov.) Friis** Habit: Tree. Habitat: Coastal forest, bushland, and riverine forest, 0–150 m. Vouchers: Verdcourt 1177, Greenway & Rawlins 9479 (EA).

**Lecaniodiscus fraxinifolius subsp. vaughanii (Dunkley) Friis** Habit: Tree. Habitat: Forest, ca. 199 m. Vouchers: Ngumbau V & Mwadime N V0423 (EA, HIB).

***Lepisanthes senegalensis* (Poir.) Leenh.** Habit: Tree. Habitat: Evergreen lowland and sub montane forest, 0–100 m. Voucher: Rawlins 353 (EA).

**Macphersonia gracilis var. hildebrandtii (O. Hoffm.) Capuron** Habit: Tree. Habitat: Coastal bushland, thicket, and forest edges, 0–100 m. Vouchers: Wamukoya x41, Luke & SA Robertson 265 (EA).

***Majidea zanguebarica* J. Kirk ex Oliv.** Habit: Tree. Habitat: Dry evergreen forest fringes, riverine, and coastal bushland, 0–300 m. Vouchers: SAJIT–005577 (EA, HIB), Gillett & Kibuwa 19874, Polhill & Paulo 803, Verdcourt 2128 (EA).

***Pancovia golungensis* (Hiern) Exell & Mendonça** Habit: Tree. Habitat: Moist evergreen lowland forest and riverine forest, 0–500 m. Vouchers: Ngumbau V & Mwadime N V0388 (EA, HIB), Drummond & Hemsley 1130, Dale in FD 3875, SA Robertson & Luke 5478, Mwadime N 53, Luke WRQ & Robertson SA 2824 (EA).

***Pancovia* sp**. Habit: Tree. Habitat: Forest. Voucher: Luke WRQ & Robertson SA 2803 (EA).

***Paullinia pinnata* L.** Habit: Liana. Habitat: Forest margins, gallery forest, moist thicket, and scrub, 0–1600 m. Vouchers: SAJIT–006019, Ngumbau V & Mwadime N V0262 & 080 (EA, HIB), Magogo & Glover 284 (EA).

***Sapindus trifoliatus* L.** Habit: Tree. Habitat: Probably not naturalized anywhere, about 0–200 m. Voucher: van Someren 316 (EA): Cultivated.

**Stadmania oppositifolia Lam. subsp. oppositifolia** Habit: Tree. Habitat: Dry evergreen forest and coastal bushland on coral rag, 0–5 m. Vouchers: RM Graham in FH 2232, Gillett & SA Robertson 24025 & 24028, AJ Faden 77/387, Mwadime N 48 (EA).

***Zanha golungensis* Hiern** Habit: Tree. Habitat: Deciduous woodland and rainforest, ca. 300 m. Vouchers: RB & AJ Faden 72/71, Luke WRQ & Robertson SA 2732 (EA).

**F156. Sapotaceae**

8 Genera, 20 Species

***Chrysophyllum viridifolium* J.M. Wood & Franks** Habit: Tree. Habitat: Evergreen lowland forests, low to moderate elevation. Voucher: Robertson SA & Luke WRQ 5208 (EA).

***Inhambanella henriquezii* (Engl. & Warb.) Dubard** Habit: Tree. Habitat: Lowland rainforest and groundwater forest, 10–300 m. Vouchers: Verdcourt 3936B, Rawlins in EAH 25/58, Greenway & Rawlins 8957, Mwadime N 60, Luke Q 1513, Luke WRQ & Baer S 8242 (EA).

***Manilkara discolor* (Sond.) J.H. Hemsl.** Habit: Tree. Habitat: Lowland and upland dry evergreen forest, ca. 20 m. Voucher: Mwadime N 50 (EA).

***Manilkara mochisia* (Baker) Dubard** Habit: Tree. Habitat: Deciduous bushland and thickets, 0–300 m. Vouchers: Drummond & Hemsley 4217, Dale in FD 3663, Polhill & Paulo 677 (EA).

***Manilkara sansibarensis* (Engl.) Dubard** Habit: Tree. Habitat: Lowland rainforest and lowland dry evergreen forest, 0–300 m. Vouchers: Ngumbau V & Mwadime N V0487 (EA, HIB), Suleman in FD 877, RM Graham 809 in FD 2313, RM Graham 695 in FD 2161, Luke WRQ 3126 (EA).

***Manilkara sulcata* (Engl.) Dubard** Habit: Tree. Habitat: Lowland dry evergreen forest, coastal woodlands, evergreen bushlands, and thickets, 0–600 m. Vouchers: Drummond & Hemsley 4231, RM Graham in FD 2145, Kuchar P 13430, Shimba Hills Survey Unit 107 (EA).

**Manilkara sp. cf. discolor (Sond.) J.H. Hemsl.** Habit: Tree. Habitat: Forest. Voucher: Luke WRQ 3139A (EA).

***Mimusops aedificatoria* Mildbr.** Habit: Tree. Habitat: Lowland rainforest, riverine forest, and groundwater forest, ca. 200 m. Vouchers: Templer 3, Dale in FD 3869, Luke WRQ & Robertson SA 538 (EA).

***Mimusops riparia* Engl.** Habit: Tree. Habitat: Riverine, ca. 120 m. Voucher: PJ Greenway 4473 (EA): Vulnerable

***Mimusops obtusifolia* Lam.** Habit: Tree. Habitat: Coastal evergreen bushland or dry evergreen forest, 1–400 m. Vouchers: SAJIT–006235, 005574 & 005374 (EA, HIB), CW Elliot in FD 1499, Battiscombe 232, Greenway & Rawlins 9481, Kimeu, J.M. 524 (EA).

***Mimusops somalensis* Chiov.** Habit: Tree. Habitat: Coastal woodlands and evergreen bushlands, 150–300 m. Vouchers: Bally 8557, Luke WRQ & Robertson SA 4631, Luke WRQ & Robertson SA 211 (EA).

**Pouteria alnifolia (Baker) Roberty var. alnifolia** Habit: Tree. Habitat: Lowland rainforest, groundwater forest, and riverine forest, 0–600 m. Vouchers: Thomas Mwadime in Mrs SA Robertson 7791, Luke WRQ & Robertson SA 242B, Drummond & Hemsley 4635, Verdcourt 1881 (EA).

***Pouteria pseudoracemosa* (J.H. Hemsl.) L.Gaut.** Habit: Tree. Habitat: Old coral rag, forest fragment, ca. 20 m. Voucher: Thomas Mwadime in Mrs SA Robertson 7791 (EA): Vulnerable.

**Sideroxylon inerme subsp. diospyroides (Baker) J.H. Hemsl.** Habit: Tree. Habitat: Thickets on the sea shore, 0–900 m. Vouchers: SAJIT–006240 & 006241(EA, HIB), Napier in CM 6411 & in CM 6272, J Adamson 263 in Bally 5956, Greenway & Rawlins 8874 (EA).

***Synsepalum brevipes* (Baker) T.D.Penn.** Habit: Tree. Habitat: Lowland rainforest and riverine forest, ca. 0–300 m. Vouchers: SAJIT–005930 (EA, HIB), Drummond & Hemsley 4032, RM Graham in FD 2050, Luke Q 1404, Luke WRQ 1623 (EA).

***Synsepalum kaessneri* (Engl.) T.D.Penn.** Habit: Tree. Habitat: Lowland rainforest and riverine forest, less than 300 m. Vouchers: Kassner 398, Robertson SA 6563, Pakia M 915 (EA).

***Synsepalum msolo* (Engl.) T.D.Penn.** Habit: Tree. Habitat: Lowland rainforest, ca. 30 m. Voucher: Sampson 51 (EA).

**Synsepalum sp. cf. subcordatum De Wild.** Habit: Tree. Habitat: Moist forest, 240–400 m. Vouchers: Luke WRQ 1846, Moomaw 1056, Faden et al. 69/487 (EA).

***Synsepalum subverticillatum* (E.A. Bruce) T.D.Penn.** Habit: Shrub. Habitat: Lowland rainforest, 0–100 m. Vouchers: Ngumbau V & Mwadime N V0483 (EA, HIB), Dale in FD 3583, Dale 1138 in CM 11481, Greenway & Rawlins 9431, Festo L, Luke Q & P 2760, Luke WRQ & Robertson SA 2804, Abdallah G 3344 (EA).

***Vitellariopsis kirkii* (Baker) Dubard** Habit: Shrub. Habitat: Lowland dry evergreen forest and coastal evergreen bushlands, 0–300 (–360) m. Vouchers: MacNaughton 85 in FD 2620, Davies RM 1257, Robertson SA & Luke WRQ 6034 (EA).

**F157. Scrophulariaceae**

1 Genus, 1 Species

***Anticharis senegalensis* (Walp.) Bhandari** Habit: Herb. Habitat: In semi desert, an ephemeral, ca. 200 m. Voucher: D Wood 1350 (EA).

**F158. Simaroubaceae**

1 Genus, 1 Species

***Quassia undulata* (Guill. & Perr.) D.Dietr.** Habit: Tree. Habitat: Thickets, open grassland, wooded grassland, montane forest, riverine, and semi-swamp forests, (0–) 150 m. Vouchers: Drummond & Hemsley 1173A, Faden & Faden 72/70, Faden RB 70/835 (EA).

**F159. Smilacaceae**

1 Genus, 1 Species

***Smilax anceps* Willd.** Habit: Liana. Habitat: Forest edges, clearings, secondary associations of bushland, thicket, commonly along rivers, lakesides or marshes, ca. 0–400 m. Vouchers: SAJIT–005970, Ngumbau V & Mwadime N V0295 (EA, HIB), Magogo & Glover 1063, Robertson SA & Luke WRQ 5181 (EA).

**F160. Solanaceae**

8 Genera, 18 Species

***Capsicum annuum* L.** Habit: Herb. Habitat: Secondary vegetation, forests edge, riverine thicket, and roadsides, ca. 150 m. Vouchers: Magogo FC & Glover PE 564, MacNaughton 127 (EA).

***Datura metel* L.** Habit: Herb. Habitat: Roadside weed or ruderal side, ca. 0–100 m. Vouchers: Graham RM 1935, Napier 6299, Thomas 154, Kassner 280 (EA): Naturalized.

***Lycium shawii* Roem. & Schult.** Habit: Shrub. Habitat: Rocky outcrops, montane scrub, riverine thickets, woodland, and bushland, ca. 15 m. Voucher: Luke et al. TPR 405 (EA).

***Lycopersicon esculentum* Mill.** Habit: Herb. Habitat: Cultivated. Voucher: Waaijenberg H 25 (EA): Cultivated.

***Nicandra physalodes* (L.) Gaertn.** Habit: Herb. Habitat: Waste places and near dwellings, ca. 130 m. Voucher: Fukuoka N 267 (EA): Cultivated.

***Physalis angulata* L.** Habit: Herb. Habitat: Damp areas, weed of cultivated crops, and ruderal of waste places, ca. 90 m. Vouchers: SAJIT–006115, Ngumbau V & Mwadime N V0291 (EA, HIB), Luke WRQ & PA 5724, Luke 1338 (EA): Naturalized.

***Physalis minima* L.** Habit: Herb. Habitat: Field edges, waste ground near houses, and roadsides. Vouchers: Brathay 1982, Expedition 40 (EA): Naturalized.

***Solanum americanum* Mill.** Habit: Herb. Habitat: Common weed of cultivation, grassland, open bush, forest clearings, shrubland, and upland rainforest, 0–200 m. Vouchers: Robertson 3454, Polhill R & Paulo S 828 (EA).

***Solanum campylacanthum* Hochst. ex A. Rich.** Habit: Shrub. Habitat: Ubiquitous weed of roadsides, abandoned cultivation, wooded grassland, bushland, dunes, and forest edges, ca. 5 m. Vouchers: Gillespie 24, Vorontsova MS 107 (EA).

***Solanum dasyphyllum* Schumach. & Thonn.** Habit: Herb. Habitat: Forest, savannah, grassland, or wasteland, ca. 30 m. Vouchers: Ngumbau V & Mwadime N V0265 (EA, HIB), Robertson SA & Luke WRQ 5177, Luke Q 1399 (EA).

***Solanum goetzei* Dammer** Habit: Herb. Habitat: Dry to moist forest, riverine forest, coastal thicket, woodland, wooded grassland, and bushland, ca. 0–750 m. Vouchers: Polhill 4818, Polhill & Paulo 750, BR Adams 76 (EA).

***Solanum malindiense* Voronts.** Habit: Shrub. Habitat: Coastal bush, dunes and sand, often on coral, 0–50 m. Vouchers: Faden & Faden 74/1072, Luke & Luke 10326, Polhill & Paulo 709, Vorontsova MS 115 (EA).

***Solanum pampaninii* Chiov.** Habit: Shrub. Habitat: Coastal sand dunes and shores, 0–9 m. Vouchers: Gillespie 120, Rawlins 25 & 156 (EA).

**Solanum sp. cf. monotanthum Damer** Habit: Herb. Habitat: Shore. Voucher: Robertson 3776 (EA).

***Solanum stipitatostellatum* Dammer** Habit: Shrub. Habitat: Forest understorey, open forest, forest edges or disturbed ground, ca. 300 m. Voucher: Verdcourt 3931 (EA).

***Solanum usaramense* Dammer** Habit: Shrub. Habitat: Coastal bushland, thickets, disturbed places, 0–500 m. Voucher: Gillett & Kibuwa 19892 (EA).

***Solanum zanzibarense* Vatke** Habit: Shrub. Habitat: Moist or dry forest, forest edges, and rocky outcrops, 0–700 m. Vouchers: Ngumbau V & Mwadime N V0527 (EA, HIB), Polhill & Paulo 850, Magogo FC & Glover PE 185 (EA).

***Withania somnifera* (L.) Dunal** Habit: Herb. Habitat: Disturbed soil, along roadsides, in cultivated land, ca. 5 m. Voucher: Luke PA & WRQ 6145 (EA).

**F161. Sphenocleaceae**

1 Genus, 1 Species

***Sphenoclea zeylanica* Gaertn.** Habit: Herb. Habitat: Forest, up to 350 m. Vouchers: SAJIT–006200 (EA, HIB), Luke WRQ & PA 4575 (EA).

**F162. Surianaceae**

1 Genus, 1 Species

***Suriana maritima* L.** Habit: Shrub. Habitat: Seashore just above the high-water mark, the beach crest, sea-level. Vouchers: Drummond & Hemsley 1044, Luke 3019, Kimeu JM 656 (EA).

**F163. Talinaceae**

1 Genus, 3 Species

***Talinum paniculatum* (Jacq.) Gaertn.** Habit: Herb. Habitat: Moist or wet fields, thickets. Voucher: Someren HD van 7172 (EA): Naturalized.

***Talinum portulacifolium* (Forssk.) Asch. ex Schweinf.** Habit: Herb. Habitat: Grassland and thicket, ca. 0–300 m. Vouchers: RB & AJ Faden 74/1195, Parker I 517 (EA).

***Talinum tenuissimum* Dinter** Habit: Herb. Habitat: Disturbed places in bushland, 50–600 m. Vouchers: Ngumbau V & Mwadime N V0340 (EA, HIB), Robertson 3484, Luke 3066, Luke WRQ et al. 6195 (EA).

**F164. Tamaricaceae**

1 Genus, 1 Species

***Tamarix senegalensis* DC.** Habit: Shrub. Habitat: Sandy desert and sea shore, ca. 100 m. Voucher: Johansson S, Gachathi N & Mulatya J 99 (EA).

**F165. Thymelaeaceae**

2 Genera, 3 Species

***Gnidia latifolia* (Oliv.) Gilg** Habit: Shrub. Habitat: Evergreen bushland and wooded grassland, ca. 50–304 m. Vouchers: Drummond & Hemsley 4040, Festo L & Luke Q 2555 (EA).

***Synaptolepis alternifolia* Oliv.** Habit: Liana. Habitat: Woodland and wooded grassland, 30–420 m. Vouchers: Luke WRQ 3099, Luke TPR794 (EA).

***Synaptolepis kirkii* Oliv.** Habit: Liana. Habitat: Lowland dry evergreen forest, *Brachystegia* woodland, coastal and secondary bushland or thicket, 0–450 m. Vouchers: SAJIT–006143 (EA, HIB), RM Graham 1805, Polhill & Paulo 776, Verdcourt 1931, Robertson SA 5070, Simpson BL 326 (EA).

**F166. Typhaceae**

1 Genus, 1 Species

***Typha domingensis* Pers.** Habit: Herb. Habitat: Swamps, dams, lakes, and rivers, ca. 0–250 m. Vouchers: SAJIT–005914 (EA, HIB), Hildebrandt 1229, Luke Q 5643 (EA).

**F167. Urticaceae**

4 Genera, 6 Species

***Laportea interrupta* (L.) Chew** Habit: Herb. Habitat: Lowland rainforest, often along roads, riverine forest, and moist places in wooded grassland, 0–550 m. Voucher: Gillespie 406 (EA).

***Laportea lanceolata* (Engl.) Chew** Habit: Herb. Habitat: Lowland rainforest and moist woodland, 50–400 m. Vouchers: SAJIT–006025 & 006153 (EA, HIB), Verdcourt 1940, BR Adams 27, Luke Q 1467 (EA).

***Pilea holstii* Engl.** Habit: Herb. Habitat: In lowland rainforest, ca. 200 m. Vouchers: Gilbert et al. 4963, BR Adams 85, Luke WRQ 2928 (EA).

***Pouzolzia fadenii* Friis & Jellis** Habit: Herb. Habitat: Lowland rainforest, ca. 0–220 m. Vouchers: Verdcourt 1858, Faden 71/792 & 74/1092, Luke WRQ & Robertson SA 1915 (EA).

***Urera sansibarica* Engl.** Habit: Liana. Habitat: Lowland rainforest, often on limestone, and climbing over rocks, 5–600 m. Vouchers: Ngumbau V & Mwadime N V057 (EA, HIB), Magogo & Glover 613, Verdcourt 3939C, Musyoki & Hansen 954, Mwadime N 41, Saufferer S 1590 (EA).

***Urera trinervis* (Hochst.) Friis & Immelman** Habit: Liana. Habitat: Along margins and in clearings of lowland evergreen forest, ca. 300 m. Vouchers: SAJIT–006191 (EA, HIB), Mwadime N 32 (EA).

**F168. Vahliaceae**

1 Genus, 2 Species

***Vahlia dichotoma* (Murray) Kuntze** Habit: Herb. Habitat: Grassland or woodland, ca. 10–1370 m. Vouchers: Thulin 303, Polhill & Paulo 590 (EA).

***Vahlia digyna* (Retz.) Kuntze** Habit: Herb. Habitat: Wet and heavy soils or on saline flats, ca. 140 m. Vouchers: Makin, RB & AJ Faden 74/ 990 (EA).

**F169. Verbenaceae**

4 Genera, 5 Species

***Lantana camara* L.** Habit: Shrub. Habitat: Roadsides or degraded lands, ca. 100 m. Voucher: Magogo FC & Glover PE 434 (EA): Naturalized.

**Lantana viburnoides (Forssk.) Vahl subsp. viburnoides** Habit: Shrub. Habitat: Woodland and bushland, ca. 40 m. Voucher: Magogo FC & Glover PE 283 (EA).

***Lippia carviodora* Meikle** Habit: Shrub. Habitat: *Acacia* and *Commiphora* bushland, 0–700 m. Voucher: Jeffrey H21/49 (EA).

***Phyla nodiflora* (L.) Greene** Habit: Herb. Habitat: Grassy area, ca. 23 m. Voucher: Kirika P, Muthoka P & Mbale M 754 (EA).

***Stachytarpheta urticifolia* (Salisb.) Sims** Habit: Shrub. Habitat: Roadside weed, grassland, bushland, and forest, ca. 0–100 m. Vouchers: Bock in EAH 15433, Church 73, Magogo & Glover 892 (EA): Naturalized.

**F170. Violaceae**

2 Genera, 10 Species

***Afrohybanthus enneaspermus* (L.) Flicker** Habit: Herb. Habitat: Ploughed lands, riverbanks, forest margins, and disturbed area, ca. 0–335 m. Vouchers: Adams BR 53, Magogo FC & Glover PE 730, Drummond & Hemsley 4042, Luke Q 1459, RM Graham in FD 1944, Magogo & Estes 1185, Jeffrey 298, Graham RM 1983 & 205 (EA).

**Rinorea angustifolia subsp. ardisiiflora (Oliv.) Grey-Wilson** Habit: Tree. Habitat: Evergreen forest, ca. 100–300 m. Vouchers: Ngumbau V & Mwadime N V0383 (EA, HIB), Hawthorne 327, Luke PA & WRQ Luke WRQ 4321 (EA).

**Rinorea angustifolia subsp. albersii (Engl.) Grey-Wilson** Habit: Tree. Habitat: Evergreen forest, ca. 0–420 m. Voucher: Robertson SA, Luke Q & Kh 297 (EA).

***Rinorea arborea* (Thouars) Baill.** Habit: Tree. Habitat: Lowland evergreen forest, 0–850 m. Vouchers: SAJIT–006076, Ngumbau V & Mwadime N V0404 (EA, HIB), Polhill & Paulo 798, Verdcourt 1077, Mohamed Abdulla in FD 3846, Gathii S 127, Luke WRQ 875 (EA).

***Rinorea elliptica* (Oliv.) Kuntze** Habit: Tree. Habitat: Forest, 50–600 m. Vouchers: Bally 2043, Mohamed Abdullah in FD 3349, Rawlins 70, Festo L, Luke Q & P 2666 (EA).

***Rinorea ferruginea* Engl.** Habit: Shrub. Habitat: Evergreen Forest, ca. 150–400 m. Vouchers: Magogo & Glover 405, Drummond & Hemsley 1104 & 3963, Luke WRQ & Robertson SA 2747 (EA).

**Rinorea ilicifolia (Welw. ex Oliv.) Kuntze var. ilicifolia** Habit: Shrub. Habitat: Lowland and sub montane evergreen forest, 0–500 m. Vouchers: SAJIT–005533, Ngumbau V & Mwadime N V0401 (EA, HIB), Mwadime N 6, Festo L & Luke Q 2538, Gillet 21069, Drummond & Hemsley 4262, Greenway & Rawlins 8950 (EA).

***Rinorea* sp. nov**. Habit: Tree. Habitat: Forest, ca. 300 m. Voucher: Luke WRQ et al. 6184 (EA).

**Rinorea sp. nr. beniensis Engl.** Habit: Tree. Habitat: Forest, ca. 420 m. Vouchers: Luke WRQ & Robertson SA 2758, Mrima/Dzombo Expedition 297 (EA).

**Rinorea squamosa subsp. kaessneri (Engl.) Grey-Wilson** Habit: Tree. Habitat: Evergreen lowland forest, 30–450 m. Vouchers: SAJIT–006033 (EA, HIB), Kassner 310, Drummond &Hemsley 3858, Mohamed Abdullah in FD 3346, Festo L, Luke Q & P 2686 (EA).

***Rinorea subintegrifolia* (P. Beauv.) Kuntze** Habit: Shrub. Habitat: Evergreen forest, ca. 50 m. Voucher: Luke WRQ, PA, Parkia M & Kahumbu P 4736 (EA).

**F171. Vitaceae**

5 Genera, 31 Species

***Ampelocissus africana* (Lour.) Merr.** Habit: Liana. Habitat: Seasonally swampy grassland, coastal and deciduous thicket, ca. 0–183 m. Vouchers: SAJIT–005453, Ngumbau V & Mwadime N V0204 (EA, HIB), Drummond & Hemsley 1024, Kirika P, Mbale M & Mbatha M 768 (EA).

**Ampelocissus obtusata subsp. kirkiana (Planch.) Wild & R.B. Drumm.** Habit: Liana. Habitat: Woodland, bushland, thicket, and riverine vegetation, ca. 60–243 m. Vouchers: Ngumbau V & Mwadime N V025, Luke & Robertson 1685 & 1760, Luke WRQ & Robertson SA 2746 (EA).

***Cayratia gracilis* (Guill. & Perr.) Suess.** Habit: Climber. Habitat: Forest, 15–120 m. Vouchers: L& L 6009, Kimeu JM KEFRI 457 (EA).

***Cayratia ibuensis* (Hook.f.) Suess.** Habit: Climber. Habitat: Edge of small forest patches, derived thickets, cleared land, and papyrus swamps, ca. 100 m. Voucher: Mutangah & Muasya 296 (EA).

***Cissus adeyana* Masinde & L.E. Newton** Habit: Climber. Habitat: Scattered scrub on rocky ground, ca. 300 m. Voucher: Mungai et al. 247 (EA).

***Cissus albiporcata* Masinde & L.E. Newton** Habit: Climber. Habitat: Bushland in rocky areas, ca. 50 m. Vouchers: Kassner 504, RM Graham in FD 1559 (EA).

***Cissus aphylla* Chiov.** Habit: Climber. Habitat: Open bushland to dense mixed thicket of *Acacia*, *Commiphora*, *Terminalia*, *Grewia*, etc., 100–750 m. Voucher: Mutang’ah 63 (EA).

***Cissus aphyllantha* Gilg** Habit: Climber. Habitat: Bushland, 240–360 m. Voucher: Luke WRQ & Robertson SA 52 (EA).

**Cissus aralioides subsp. orientalis Verdc.** Habit: Climber. Habitat: Coastal and riverine evergreen forest, 60–210 m. Vouchers: Ngumbau V & Mwadime N V0380 (EA, HIB), Verdcourt 3935B, Moggridge 170, SA Robertson 6727 (EA).

***Cissus integrifolia* (Baker) Planch.** Habit: Climber. Habitat: Evergreen forest, woodland, bushland, and grassland, (5–) 10–300 m. Vouchers: Ngumbau V & Mwadime N V0215 (EA, HIB), Verdcourt 1906, RM Graham D434, Rawlins in EAH 11276, Kimeu JM 533, Magogo FC & Glover PE 197 (EA).

***Cissus phymatocarpa* Masinde & L.E. Newton** Habit: Climber. Habitat: Coastal bush, thickets on coral rock, and forest edges, ca. 0–225 m. Vouchers: Magogo & Glover 997, Masinde 326, Smith SS 12 (EA).

***Cissus quinquangularis* Chiov.** Habit: Climber. Habitat: Forest, woodland, grassland with scattered trees, 0–300 m. Vouchers: Ngumbau V & Mwadime N V002 (EA, HIB), Bally 5789, Brenan et al. 14660, Verdcourt 3602, Jeffery GW 137, Masinde PS 331 (EA).

**Cissus rotundifolia Vahl var. rotundifolia** Habit: Climber. Habitat: *Commiphora*-*Acacia* scrub, dry forest and forest edges, 0–304 m. Vouchers: Bally & Smith 14366, Magogo FC & Glover PE 676, Frazier J 903 (EA).

**Cissus rotundifolia var. ferrugineo-pubescens Verdc.** Habit: Climber. Habitat: Bushland, 300–365 m. Vouchers: Verdcourt 2351, Kuchar 7345 (EA, HIB).

***Cissus sciaphila* Gilg** Habit: Climber. Habitat: Lowland forest, riverine forest fringes, and woodland, 0–450 m. Vouchers: Adams 101, Robertson & Luke 5394 (EA).

***Cissus sylvicola* Masinde & L.E. Newton** Habit: Climber. Habitat: Evergreen forest, also forest on rocky hills, coral limestone, and thicket, 5–500 m. Vouchers: SAJIT–006013 (EA, HIB), Verdcourt 2406, Sangai in EAH 15735, Masinde 355, Luke Q 1522, Masinde PS 357 (EA).

***Cyphostemma adenocaule* (Steud. ex A. Rich.) Desc. ex Wild & R.B. Drumm. subsp. adenocaule** Habit: Climber. Habitat: Fringing forest, clearings in evergreen forest, bushland, thicket, grassland, ca. 0–300 m. Vouchers: SAJIT–005476 (EA, HIB), Polhill & Paulo 733, Magogo FC & Glover PE 74 (EA).

***Cyphostemma buchananii* (Planch.) Desc. ex Wild & R.B. Drumm.** Habit: Climber. Habitat: Coastal thicket on old coral reefs, bushland, wooded grassland, and woodland, ca. 10 m. Vouchers: SA Robertson 3548, Magogo & Glover 945, Haller 25, fide Wild & Drummond (EA).

**Cyphostemma cyphopetalum var. nodiglandulosum (T.C.E.Fr.) Verdc.** Habit: Climber. Habitat: Forest, grassland margins, grassland with scattered trees and derived waste places. Voucher: Nash LT 47 (EA).

***Cyphostemma duparquettii* (Planch.) Desc.** Habit: Climber. Habitat: Lowland forest, 10–600 m. Vouchers: Ngumbau V & Mwadime N V0194 (EA, HIB), Verdcourt 1907, Robertson SA 6842 (EA).

**Cyphostemma dysocarpum subsp. pwani Verdc.** Habit: Climber. Habitat: Bushland near shore and on open rocky sites, 0–several m. Vouchers: Tweedie 944, de Meester 48, SA Robertson 6710, Rawlins SP 1 (EA).

***Cyphostemma engleri* (Gilg) Desc.** Habit: Climber. Habitat: Low *Commiphora*-*Acacia*-*Combretum*-*Terminalia*-*Boscia* bushland, (20–) 350–600 m. Vouchers: Drummond & Hemsley 4140, Kassner 502, Vercourt 5303 (EA).

***Cyphostemma hildebrandtii* (Gilg) Desc. ex Wild & R.B. Drumm.** Habit: Climber. Habitat: Grassland, scrub, and thicket, ca. 15–200 m. Vouchers: Ngumbau V & Mwadime N V0544 (EA, HIB), Drummond & Hemsley 1086, RM Graham A 549 in FD 1933, Luke Q 1540 (EA).

***Cyphostemma kirkianum* (Planch.) Desc. ex Wild & R.B. Drumm. subsp. kirkianum** Habit: Climber. Habitat: Rainforest or lowland evergreen forest on coral, ca. 0–600 m. Vouchers: Brenan et al. 14507, Robertson & Luke 5887, SA Robertson 5915 (EA).

***Cyphostemma pachyanthum* (Gilg & M. Brandt) Desc.** Habit: Climber. Habitat: Rainforest, ca. 200 m. Vouchers: Luke & Robertson 1896, Luke WRQ & Robertson SA 2254 (EA).

***Cyphostemma* sp. G** Habit: Climber. Habitat: Coastal bushland. Voucher: Luke & Robertson 1284 (EA).

***Cyphostemma* sp. I of FTEA** Habit: Climber. Habitat: Moist forest, ca. 304 m. Vouchers: Spjut RW & Ensor PD 2682, Robertson SA & Luke WRQ 5148 (EA).

***Cyphostemma* sp. L** Habit: Climber. Habitat: Forest, ca. 70 m. Voucher: Luke & Robertson 1691 (EA).

***Cyphostemma ternatum* (Forssk.) Desc.** Habit: Climber. Habitat: Open bushland and grassland, 30–230 m. Voucher: Luke & Robertson 1186 (EA).

***Cyphostemma zimmermannii* Verdc.** Habit: Climber. Habitat: Streamside in rainforest, also *Brachystegia* patches, ca. 330 m. Vouchers: SAJIT–006060, Ngumbau V & Mwadime N V028 & 0253 (EA, HIB), GG van Someren Sh 71, Magogo & Glover 74, Hawthorne 208, Luke WRQ & PA, Kahumbu & Lehman 4688, Luke WRQ & Robertson SA 2711 (EA).

***Rhoicissus revoilii* Planch.** Habit: Liana. Habitat: Grassland, bushland, and woodland, ca. 0–410 m. Vouchers: SAJIT–005424 (EA, HIB), RM Graham 496 in FD1871, Katz SS 75/63, Luke WRQ & Robertson SA 2786D (EA).

***Rhoicissus tridentata* (L.f.) Wild & R.B. Drumm.** Habit: Liana. Habitat: Often associated with rocky places, ca. 240 m. Vouchers: Luke WRQ & Robertson SA 2786C & 543 (EA).

**F172. Ximeniaceae**

1 Genus, 2 species

***Ximenia americana* L.** Habit: Shrub. Habitat: Wooded grassland, deciduous, and coastal bushland, ca. 50 m. Vouchers: SAJIT–004652 (EA, HIB), Moggridge 215, Simpson BL 296 (EA).

**Ximenia caffra var. natalensis Sond.** Habit: Shrub. Habitat: Dry woodland, bushland, and wooded grassland, ca. 10 m. Vouchers: Drummond & Hemsley 4167, Mwadime N & Luke WRQ 546 & 743, Robertson SA 4317 (EA).

**F173. Xyridaceae**

1 Genus, 3 Species

**Xyris anceps Lam. var. anceps** Habit: Herb. Habitat: Perennial swamps and pools near the coast, 0–100 m. Vouchers: Ngumbau V & Mwadime N V0548 (EA, HIB), SA Robertson 3509 (EA).

**Xyris anceps var. minima (Steud.) Lock** Habit: Herb. Habitat: Seasonal pools, marshes, and rice fields, ca. 0–200 m. Vouchers: Luke & SA Robertson 2713, Magogo & Glover 354 (EA).

***Xyris parvula* Malme** Habit: Herb. Habitat: Sandy seasonally wet places near the sea, 0–10 m. Voucher: RM Graham 2113 (EA).

**F174. Zingiberaceae**

2 Genera, 6 Species

***Aframomum amaniense* Loes.** Habit: Herb. Habitat: Moist forest undergrowth, ca. 304 m. Voucher: Gardner FD 1461 (EA).

***Aframomum angustifolium* (Sonn.) K. Schum.** Habit: Herb. Habitat: Forest undergrowth, forest margins, and gallery forest, ca. 300 m. Voucher: Drummond RB & Hemsley JH 1177 (EA).

***Aframomum orientale* Lock** Habit: Herb. Habitat: Coastal forest and abandoned cultivation, 100–400 m. Voucher: Magogo FC & Glover PE 263 (EA).

***Aframomum alboviolaceum* (Ridl.) K. Schum.** Habit: Herb. Habitat: Moist savannas, often in *Brachystegia* woodland, ca. 375 m. Voucher: Padwa 446 (EA).

***Siphonochilus brachystemon* (K. Schum.) B.L. Burtt** Habit: Herb. Habitat: Drier evergreen forests, ca. 50–300 m. Vouchers: SAJIT–005531 (EA, HIB), Luke WRQ 3137 (EA).

***Siphonochilus kirkii* (Hook.f.) B.L. Burtt** Habit: Herb. Habitat: Woodland, ca. 450 m. Vouchers: Ngumbau V & Mwadime N V0207 (EA, HIB), Magogo FC & Glover PE 919 (EA).

**F175. Zosteraceae**

1 Genus, 1 Species

***Zostera capensis* Setch.** Habit: Herb. Habitat: In shore sandbanks or mudflats in fairly shallow water, 0–6 m. Vouchers: Greenway & Rawlins 8902, Isaac 105 & 98, fide Isaac 1968, Isaac A109 (EA): Vulnerable.

**F176. Zygophyllaceae**

3 Genera, 8 Species

***Balanites aegyptiaca* (L.) Delile** Habit: Tree. Habitat: Wooded and scattered tree grassland or deciduous bushland. Voucher: Perdue RE & Kibuwa SP 10096 (EA).

**Balanites maughamii subsp. acuta Sands** Habit: Tree. Habitat: Coastal forest, riverine thicket, and groundwater forest, ca. 0–400 m. Vouchers: Ngumbau V & Mwadime N V0459 & 0355 (EA, HIB), Gilbert 6038 (EA).

***Balanites pedicellaris* Mildbr. & Schltr.** Habit: Tree. Habitat: Bushland, woodland or grassland, ca. 0–450 m. Vouchers: Ngumbau V & Mwadime N V0449 (EA, HIB), Robertson SA & Luke WRQ 6058 (EA).

***Balanites rotundifolia* (Tiegh.) Blatt.** Habit: Tree. Habitat: Bushland and grassland, 0–800 m. Vouchers: Luke Q 1536, Luke & Roberson 1536 (EA).

***Tetraena simplex* (L.) Beier & Thulin** Habit: Herb. Habitat: Open places in deciduous bushland, 0–600 m. Vouchers: RB & AJ Faden 74/783, Luke WRQ 5462 (EA).

***Tribulus cistoides* L.** Habit: Herb. Habitat: Sand and coral along coast, also open places inland, 0–800 m. Vouchers: Verdcourt 3616, Lucas GLl, Jeffrey C & Kirika 265 (EA).

**Tribulus parvispinus C. Presl var. parvispinus** Habit: Herb. Habitat: Open disturbed area, ca. 2 m. Voucher: Mwadime N & Chesire C 259 (EA).

***Tribulus terrestris* L.** Habit: Herb. Habitat: Bushland, tree grassland, ca. 40–420 m. Vouchers: TPR 739, Mugai GM & Rucina SM 491/84 (EA).
